# AuCl_3_-Catalyzed Hemiacetal Activation for the Stereoselective Synthesis of 2-Deoxy Trehalose Derivatives

**DOI:** 10.1021/acs.orglett.2c02530

**Published:** 2022-08-22

**Authors:** Robin Jeanneret, Carlo Walz, Maarten van Meerbeek, Sarah Coppock, M. Carmen Galan

**Affiliations:** School of Chemistry, University of Bristol, Cantock’s Close, Bristol BS8 1TS, United Kingdom

## Abstract

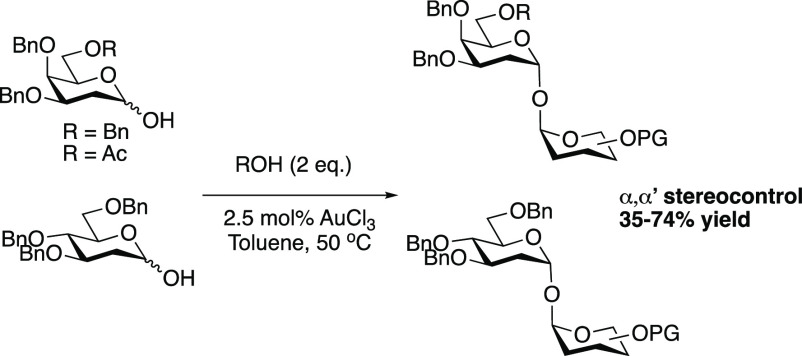

A new practical, catalytic, and highly stereoselective method for directly accessing 1,1-α,α′-linked 2-deoxy trehalose analogues via AuCl_3_-catalyzed dehydrative glycosylation using hemiacetal glycosyl donors and acceptors is described. The method relies on the chemoselective Brønsted acid-type activation of tribenzylated 2-deoxy hemiacetals in the presence of other less reactive hemiacetals.

Accessing structurally defined carbohydrates is essential to probe the complex biological roles that carbohydrates play.^1,2^ Thus, the development of novel, efficient, and practical strategies for the stereoselective formation of glycosidic linkages, to add to the existing toolkit of carbohydrate chemistry, is still needed to push the boundaries of glycobiology research.^3−6^

Trehalose is a symmetrical disaccharide composed of two 1,1-α,α′-linked glucose subunits. Trehalose monomycolate (TMM) and dimycolate (TDM), bearing one and two 6-*O-*mycolyl substituents, respectively, are produced in all mycobacterial species and have been shown to be crucial components of the outer layer of the cell wall of *Mycobacterium tuberculosis* (*Mtb*). These glycolipids play essential roles in *Mtb* cell wall biosynthesis and in the viability and virulence of the pathogen.^2,7,8^ Targeting trehalose uptake and subsequent metabolism has garnered attention in recent years as an attractive route for the development of novel therapeutics and diagnostic agents.^9−14^ Previous elegant studies reported the synthesis of a series of symmetrical and unsymmetrical trehalose mimetics, including amino, azido, fluoro, iodo, 2-deoxy, and phosphate functionalities, as well as a fluorescein-functionalized analogue that was shown to label *Mtb*.^10^ Subsequently, a range of differently functionalized trehalose analogues with fluorescent dyes^12,15,16^ and biorthogonal handles,^17^ including azides,^18,19^ alkynes,^11,20^ and photoactivatable diazirines,^21^ have been shown to be metabolically incorporated into the mycomembrane of live mycobacteria.

Different strategies exist by which unsymmetrical, functionalized trehalose derivatives can be accessed.^22,23^ Enzymatic methods have been successfully applied to the synthesis of a range of trehalose analogues.^24,25^ Alternatively, chemical synthesis involving either the desymmetrization of natural trehalose or chemical glycosylation of two separate building blocks is also possible. The former often requires long regioselective protection/deprotection and functional group interconversion sequences with the desymmetrisation step often being low-yielding.^26^ On the contrary, chemical glycosylation can be used for the construction of the 1,1-α,α′-linkage, bringing together a glycosyl donor and a hemiacetal acceptor; however, unlike enzymatic syntheses, the chemical synthesis of unsymmetrical trehalose derivatives is often more problematic due to the potential for the formation of up to four diastereomers, unwanted dimerization of the reactive components, and the formation of side products, decreasing the efficiency of the overall synthesis. Alternative methods for the stereoselective synthesis of unsymmetrical α,α′-linked trehalose derivatives using intramolecular aglycone delivery have been described.^27,28^ Moreover, the synthesis of ketoside-type analogues of trehalose via Lewis acid-catalyzed activation of exoglycals and ketoside hemiacetals has also been reported.^29^

Our group is interested in the development of expedient and efficient catalytic methods for the synthesis of 2-deoxy glycosides, which are prominent components of a number of natural products.^4^ A number of glycosylation protocols exist for the stereoselective formation of 2-deoxy linkages,^4^ but few examples of 2-deoxy trehalose analogues have been reported. For instance, symmetrical 2-deoxy trehalose derivatives via debenzylation deiodination of a 2,2′-diiodo derivative prepared by dehydrative dimerization of the benzylated 2-iodo hemiacetal have been described.^30^ McGarrigle et al. reported the organocatalytic synthesis of symmetrical and unsymmetrical 2-deoxy trehalose derivatives via activation of galactal **1**; unfortunately, the products were formed as a mixture of anomers.^31^ Herein, we report the development of a new, practical, and stereoselective method for accessing 1,1-α,α′-linked 2-deoxy trehalose derivatives via AuCl_3_-catalyzed dehydrative glycosylation using hemiacetal glycosyl donors and acceptors.

During our previous work on the synthesis of 2-deoxy glycosides via the Au(I)/Ag(I)-catalyzed activation of glycals, we found that activation of **1** using Lewis acidic AuCl_3_ formed an inseparable mixture of products, including 2,3-unsaturated Ferrier products (Scheme 1, top).^32^ As part of our ongoing work on the development of catalytic glycosylation methods, AuCl_3_ was further investigated as a catalyst for the activation of 2-deoxy hemiacetals **2a**(33) and **3a**(34) as an alternative starting material to the 1,2-unsaturated glycals.

**Scheme 1:**
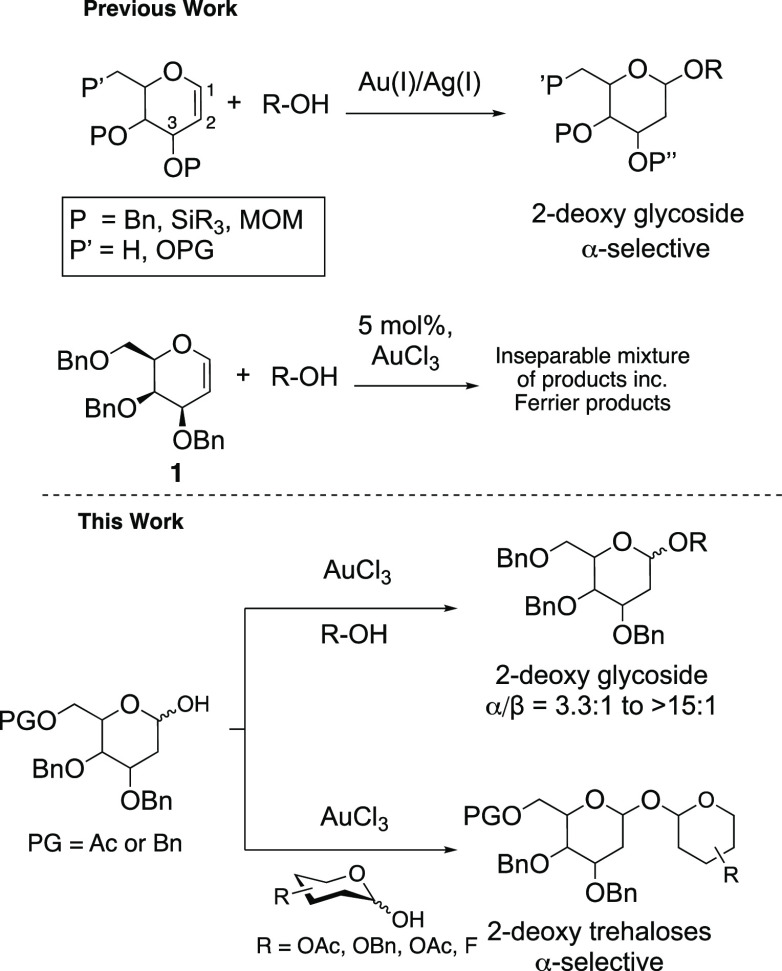
AuCl_3_-Catalyzed Activation of 2-Deoxy Hemiacetals

Gold catalysis has been widely applied to carbohydrate synthesis.^35^ For instance, AuCl_3_ has been reported for the catalytic activation of acetylated glycals to give 2,3-unsaturated Ferrier products,^36^ thioglycosides,^37^ trichloroacetimidates,^38^ and alkynyl donors.^39,40^ AuBr_3_ has also been reported for the activation of methyl glycosides.^41^ Moreover, a number of methods for the activation of glycosyl hemiacetals using Lewis and Brønsted acids have been reported for the activation of glycosyl hemiacetals,^4,5^ including gold chloride in combination with allyl trimethylsilane to generate C-glycosides.^42^ However, to the best of our knowledge, the application of AuCl_3_ for the direct activation of hemiacetals to access *O*-glycosides has not been reported to date.

In our initial studies, we found 1 mol % AuCl_3_ in EtOAc at 50 °C could catalyze the glycosylation of tribenzylated 2-deoxy galactosyl hemiacetal **2a** with **4a** to give the corresponding 2-deoxy galactoside **5a** in 70% yield with an α:β ratio of 12:1 (Table 1, entry 1). Following these encouraging results, hemiacetals **2a–c** and **3a** were reacted with a range of primary nucleophiles using between 1 and 3 mol % AuCl_3_ in either EtOAc or toluene to form the corresponding 2-deoxy glycosides in 59–84% yields. In all cases, the α-anomer was favored (α:β = 3.3:1 to >15:1) (Table 1, entries 1–9). See the Supporting Information for full details and solvent and temperature optimization screening.^43^ Lower yields (10–25%) of the desired 2-deoxy glycoside products were observed with less reactive secondary alcohols **4h** and **4i** (Table 1, entries 10–12). This was proposed to be a result of competitive dimerization of the donor, even when an excess of the alcohol was used. When 4-thiocresol (**4j**) and propargyl alcohol (**4k**) were used as acceptors, no reaction occurred, which can be attributed to coordination of the gold catalyst to the acceptor (Table 1, entries 13 and 14, respectively). In the case of benzylidene-protected **4l** (Table 1, entry 15), removal of the benzylidene group was observed as evidenced by NMR, which suggests the presence of a catalytic acid and/or moisture in the reaction. Finally, no reaction was observed with less reactive triacetylated 2-deoxy hemiacetals **2b**(34) and **3b** and 2-fluorogalactoside **2c**(33) glycosyl donors under the optimized reaction conditions in toluene using primary acceptor **4a** or **4c** after 24 h (entries 16–18).

**Table 1:**
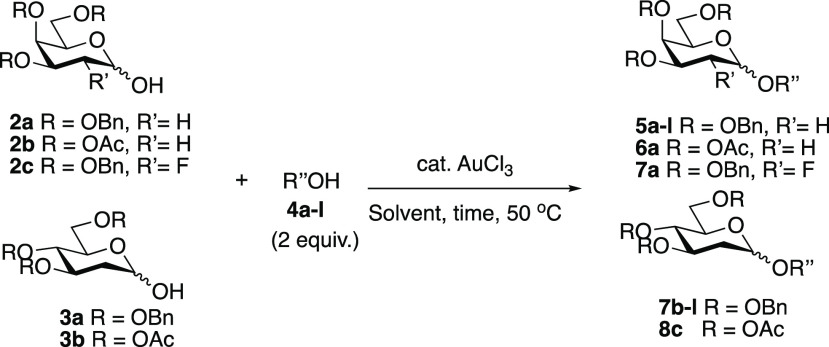

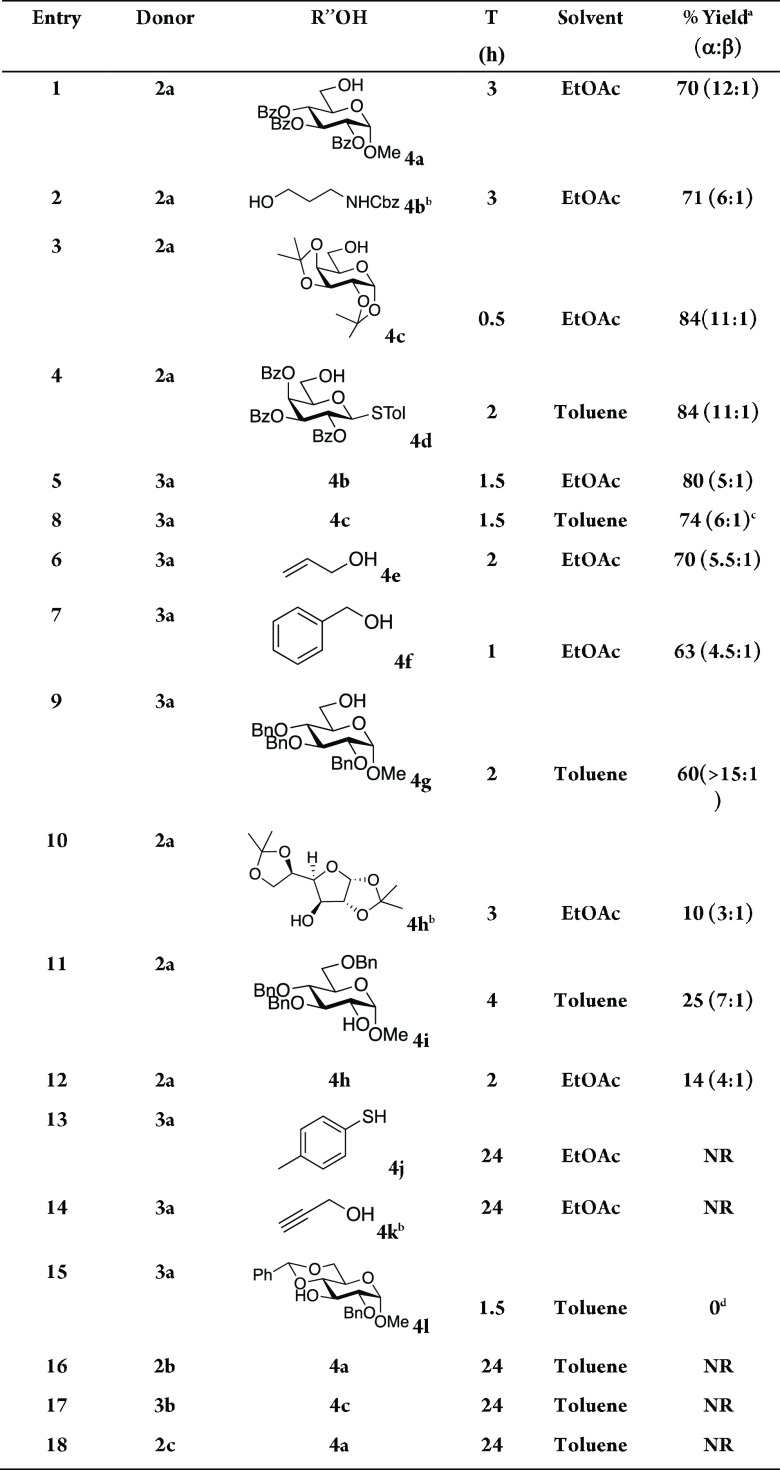
Glycosylation Reactions with Glycosyl Donors **2a–c**, **3a**, and **3b**

a As determined by H NMR.

b With 1.5 equiv.

c With 55% unreacted **4c** recovered.

d Benzylidene acetal cleavage occurred. NR = no reaction.

Generally, glycosylation reactions are conducted under strictly anhydrous conditions to minimize unwanted hydrolysis of the glycosyl donor. In our case, performing the AuCl_3_-catalyzed reactions under an inert atmosphere or using anhydrous solvents did not have an effect on the reaction yield or time, demonstrating that the process is compatible with the use of “wet” solvents and can be performed under air.

Dimerization of the hemiacetal donors was detected when secondary alcohols were used as acceptors in the Au(III)-catalyzed reactions, which suggested the reaction condition could be amenable to the direct synthesis of 2,2′-deoxy trehalose mimetics. To that end, hemiacetal donors **2a** or **3a** were treated with 1 mol % AuCl_3_ in toluene in the absence of any alternative OH nucleophile. Pleasingly, dimers **10** and **11** were isolated in 55% yields. In both cases, only the α,α′-linked products were observed (Scheme 2). Reaction of 6-deoxy fucose hemiacetal **9**(44) under the same conditions led to the formation of **12** in 60% yield (only the α,α′-linked). When less reactive donors **2b**, **2c**, and **3b** were employed, no reaction was observed, suggesting the substrates are unreactive toward glycosylation and dimerization under the mild conditions.^45^

**Scheme 2:**
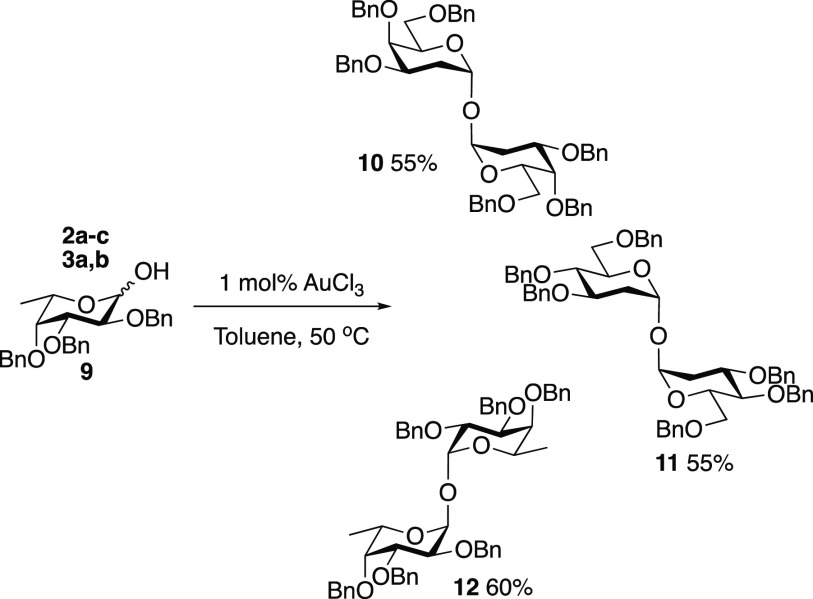
Dimerization Reactions of Deoxy Hemiacetals **2a–c**, **3a**, **3b**, and **8**

The difference in reactivity of the functionalized hemiacetals under our reaction conditions paved the way for the investigation of the selective activation of more reactive 2-deoxy hemiacetals (e.g., **2a** and **3a**) as a method for providing access to unsymmetrical trehalose derivatives. To this end, differently protected hemiacetal acceptors **13a–c**, **14**, and **2c** were reacted with **2a**, **3a**, and **2d** using 2.5 mol % AuCl_3_ in toluene at 50 °C, and the desired unsymmetrical products **15a–g**, **16**, and **17a–c** were isolated in 32–76% yields and exclusively as α,α′-linked products (Table 2).^46^ Hemiacetal **2d** protected with an acetate group at *O*-6 was also synthesized and successfully glycosylated with acceptor **13a** to form disaccharide **16** in 54% yield (entry 4).^47^

**Table 2:**
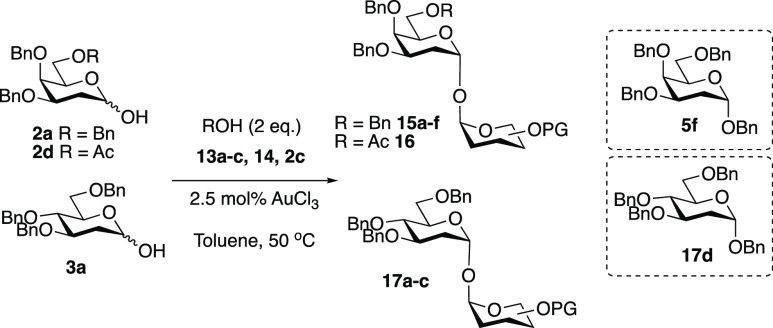

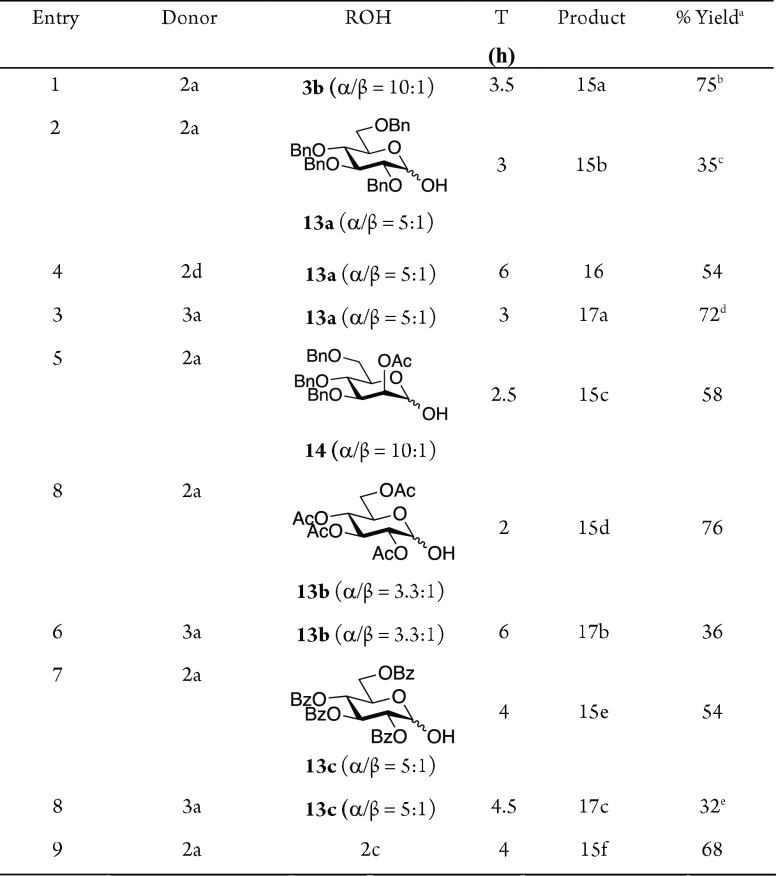
Synthesis of Unsymmetrical 2-Deoxy Trehalose Derivatives

a As determined by H NMR.

b With **15h** (4%).

c With **15h** (13%).

d With **17d** (4%).

e With **17d** (43%).

One of the advantages of this chemoselective strategy is the ability to perform orthogonal late-stage functionalizations. To exemplify this, the divergent synthesis of 6-azido derivatives **21** and **25** from common disaccharide **17c** was carried out (Scheme 3). Selective deprotection of the benzyl or benzoyl protecting groups could be performed using palladium-mediated hydrogenolysis in 95% yield or LiOH-mediated ester hydrolysis (98% yield), respectively. In each case, a tosyl group was selectively installed at the more reactive *O*-6 hydroxyl group. In the case of **19**, NaN_3_ treatment gave 6-azido disaccharide **20**. Following ester hydrolysis, 6-azido trehalose derivative **21** was accessed with the azido group installed on the 2-deoxyglucose unit. Due to the instability of azido groups to common reductive methods, 6-tosyl disaccharide **23** was hydrogenated in the presence of palladium to form **24**, and the 6-tosyl group was then converted into an azido group to give **25**, whereby the glucose unit bears the 6-azido group.

**Scheme 3:**
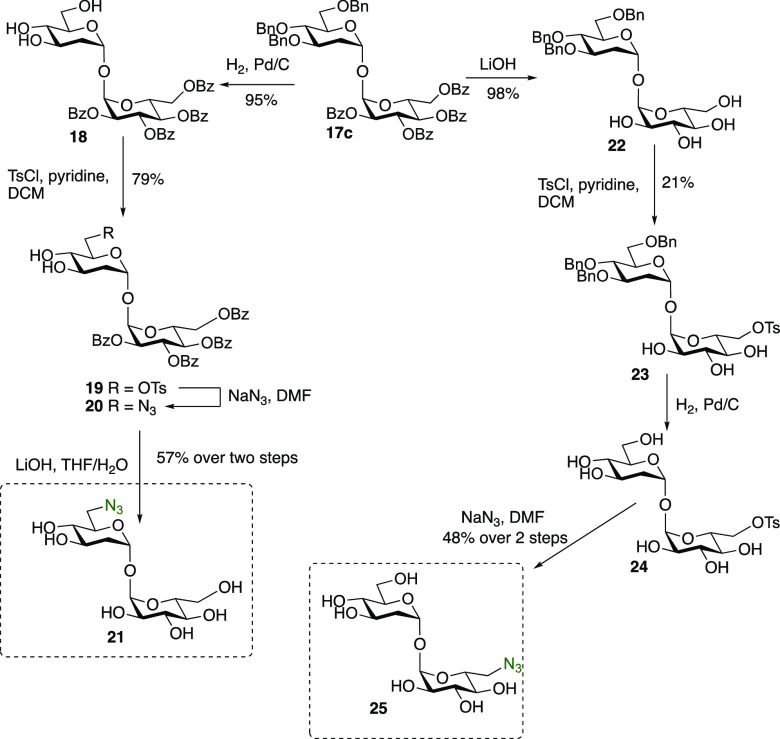
Synthesis of 6-Azido and 6′-Azido 2-Deoxy Trehalose Derivatives **21** and **25**

Mechanistically, it was initially postulated that AuCl_3_ could act as a Lewis acid, coordinating to the hydroxyl group of the 2-deoxy hemiacetal to promote the formation of a transient oxocarbenium ion that can react with the less reactive hemiacetal acceptor. However, we found addition of organic or inorganic bases (DIPEA or K_2_CO_3_) stopped the reaction (Scheme S1), indicating a Brønsted acid-type mechanism might be plausible.^48^ It was also found that dimerization of 2-deoxy hemiacetal donor **2a** also occurred upon treatment with HCl, albeit in lower yields (Table S6). However, formation of unsymmetrical trehalose derivative **15e** using benzoylated hemiacetal **2a** and acceptor **13c** was not observed using HCl (Scheme S2). A number of different activation conditions were also tested for this reaction, but lower yields and/or less clean reaction profiles were observed compared to those with the use of AuCl_3_ (Table S7). ^1^H NMR spectroscopy studies in *d*_8_-toluene with equimolar mixtures of AuCl_3_ and hemiacetal acceptor **13c** did not indicate any interaction or reaction between the gold catalyst and the nucleophile (Figure S1). Although it cannot be entirely ruled out as a reactive intermediate, 2-deoxy glycosyl chlorides were not observed at any point by NMR spectroscopy. Moreover, a 4:1 α,α′/α,β′-anomeric mixture of **15b**(31) was subjected to the reaction conditions using 2.5 mol % AuCl_3_ to investigate whether the α,α′ selectivity was the result of *in situ* anomerization. An increase in the proportion of the α,β′ diastereomer as well as the formation of small amounts of hydrolyzed hemiacetal **13a** was observed (Table S5). These results indicate the α,α′ selectivity of the reaction is not due to anomerization and highlights the importance of not leaving the reactions for longer than necessary.

In summary, we have developed a new practical and catalytic method for the synthesis of 2-deoxy trehalose derivatives via Au(III) chemoselective activation of tribenzylated 2-deoxy hemiacetals in the presence of other less reactive hemiacetals. Due to the catalytic nature of the activation system, the glycosylation reactions could be performed under non-anhydrous conditions. Despite starting with a mixture of anomers for both the donor and the acceptor, only the α,α′-linked products were generated. The protecting group pattern of the acceptors could be varied, and this allows for the orthogonal modification of functionality at a later stage. The versatility of this approach was highlighted via the synthesis of 6- and 6′-azido-functionalized 2-deoxy trehalose analogues, which are useful tools for studying the biosynthetic pathway of *Mtb*.

## Supporting Information Available

Experimental procedures, full characterization data and copies of NMR data (PDF)
