# Supplementary material for: AuCl3-Catalyzed Hemiacetal Activation for the Stereoselective Synthesis of 2-Deoxy Trehalose Derivatives
Source: Org Lett. 2022 Aug 22;24(34):6304–9. doi: 10.1021/acs.orglett.2c02530 (PMC9442795; doi:10.1021/acs.orglett.2c02530)

Supplementary Information

$AuCl_3$-catalysed hemiacetal activation for the stereoselective synthesis of 2-deoxy trehalose derivatives

Robin Jeanneret, Carlo Walz,‖ Maarten van Meerbeek,‖ Sarah Coppock, M. Carmen Galan*

School of Chemistry, University of Bristol, Cantock's Close, Bristol BS8 1TS, United Kingdom

General experimental 2
Synthesis of 2-deoxy hemiacetals 2a-d and 3a-b 3
List of glycosyl acceptors used for the synthesis of 2-deoxy glycosides (Table 1) 8
General procedure A - $AuCl_3$ catalysed glycosylation reactions 10
Reaction optimisation/screens 10
  Table S1 – Solvent screen using 10 mol% $AuCl_3$ 11
  Table S3 – Effect of temperature 12
  Table S4 – Solvent screen at 50 °C 12
Synthesis of 2-deoxy glycosides (Table 1) 13
Synthesis of deoxy-trehalose dimers 10, 11 and 12 25
Synthesis of OEt derivatives S11 and S12 28
List of hemiacetal acceptors used for the synthesis of 2-deoxy trehalose derivatives 30
Synthesis of unsymmetrical trehalose derivatives (Table 2) 30
Synthesis of 6-azido and 6' azido 2-deoxy trehalose derivatives 21 and 25 40
Control Reactions and Mechanistic Studies 47
  Attempted $AuCl_3$ catalysed anomerisation of 15b 47
  Table S5 – Results of anomerisation of 15b 47
  Experimental procedure for attempted $AuCl_3$ catalysed anomerisation 48
  NMR experiments 49
  Control reactions using base 50
  The attempted synthesis of 2-deoxy trehalose derivatives using alternative activation systems 50
  Table S6 – Synthesis of Dimer 10 using HCl 51
  Scheme S2 – Attempted formation of asymmetric trehalose 15e using HCl 51
  Table S7 – Asymmetric trehalose formation using alternative activation systems 52
References 53
NMR spectra 56

General experimental

All reactions were performed using reagent grade 'wet' solvent under air unless otherwise stated. Reactions requiring anhydrous conditions were performed under nitrogen; glassware and needles were either flame dried immediately prior to use or placed in an oven (150 °C) for at least 2 hours and allowed to cool either in a desiccator or under reduced pressure; liquid reagents, solutions or solvents were added *via* syringe through rubber septa; solid reagents were added *via* Schlenk type adapters. Compositions of solvent mixtures are quoted as ratios of volumes unless otherwise stated. Organic solutions were dried by adding anhydrous $MgSO_4$ and concentrated by rotary evaporation under reduced pressure using both a Büchi rotary evaporator at a pressure of either 15 mmHg (diaphragm pump) or 0.1 mmHg (oil pump), as appropriate, and a high vacuum line at room temperature. Reactions were monitored by TLC on Kieselgel 60 F254 (Merck). Detection was by examination under UV light (254 nm) and cerium molybate (Hanesian's stain) or $KMnO_4$. Flash column chromatography was performed using silica gel [Merck, 230−400 mesh (40−63 μm)] using the flash technique.[1] Amberlite® and Dowex® resins were purchased from Sigma- Aldrich. Sephadex™ resins were purchased from GE. Reactions requiring heating were carried out on a Drysyn heating block.

^{1}H NMR and ^{13}C NMR spectra were measured in the solvent stated at 400 or 500 MHz. Chemical shifts are quoted in parts per million from residual solvent peak ($CDCl_3$: ^{1}H - 7.26 ppm and ^{13}C - 77.16 ppm) and coupling constants (*J*) given in Hertz. ^{13}C shifts are given to 1 d.p. unless 2 d.p. is required to distinguish peaks. Multiplicities are abbreviated as: bs (broad), s (singlet), d (doublet), t (triplet), q (quartet), m (multiplet) or combinations thereof. Coupling constants (*J* values) are quoted to the nearest 0.1 Hz and are given as observed i.e. not made equal for coupling protons. Where a signal in the ^{1}H or ^{13}C NMR cannot be fully assigned as much information as possible is given for assignment of the signal. If ^{13}C signals overlap (as determined by HSQC or HMQC) then the signal assignments are included in the same bracket (e.g. C-2 and C-6) or (2 x CH_2Ph). Where ^{1}H NMR data for a mixture of anomers has been measured, separate ^{1}H assignments are given. Where signals in the ^{13}C NMR spectrum are not assignable due to spectral overlap, the multiplicity of the carbon is given (e.g. CH). Structural assignments were made with additional information from gCOSY, gHSQC, and gHMBC experiments. Mass spectrometry was carried out by the University of Bristol Mass Spectrometry Service on a micrOTOF II (ESI) spectrometer, with the HRMS mode incorporating a lock-in mass injected midway through the run (sodium formate).

Synthesis of 2-deoxy hemiacetals 2a-d and 3a-b

3,4,6-Tri-O-benzyl-2-deoxy-α/β-D-galactopyranose 2a

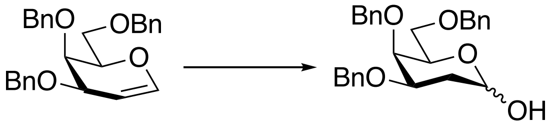

Concentrated 36% HCl (0.4 mL) was added to a solution of tri-*O*-benzyl galactal **1** (3.0 g, 7.2 mmol) in a mixture of THF and H_2O (60 mL, 9:1). The reaction was stirred at RT for 24 hr**.** Solid $NaHCO_3$ was added until a pH of 7 was reached, followed by extraction using CH_2Cl_2 (50 mL), dried, filtered and concentrated *in vacuo*. Recrystallisation by dissolving in Et_2O and slowly adding hexane gave the *title compound* **2a** as thin white needles (2.58 g, 5.9 mmol, 82%, α/β = 5:1); R_f = 0.29 (3:2 Hexane/EtOAc); MS (ES^+) found *m/z* 457.5 $[M+Na]^+$.

NMR data for α-anomer: ^{1}H NMR (400 MHz, Chloroform-*d*) δ 7.38–7.29 (15 H, m, Ar*H*), 5.48 (1 H, br t, *J* = 2.5 Hz, H-1), 4.95 (1 H, d, *J* = 11.7 Hz, C*H*$_2$Ph), 4.66–4.62 (3 H, m, 3 x C*H*$_2$Ph), 4.53 (1 H, d, *J* = 12.0 Hz, C*H*$_2$Ph), 4.46 (1 H, d, *J* = 12.0 Hz, C*H*$_2$Ph), 4.17–4.14 (1 H, m, H-5), 4.01 (1 H, ddd, *J* = 12.0, 4.5, 2.5 Hz, H-3), 3.89 (1 H, br s, H-4), 3.61 (1 H, dd, *J* = 9.5, 6.7 Hz, H-6a), 3.50 (1 H, dd, *J* = 9.5, 5.8 Hz, H-6b), 2.73 (1 H, t, *J* 3.5 Hz, O*H*), 2.28–2.20 (1 H, m, H-2a), 2.06–2.01 (1 H, m, H-2b); ^{13}C NMR (101 MHz, $CDCl_3$) δ 138.8 (Ar C), 138.5 (Ar C), 138.0 (Ar C), 128.42 (Ar CH), 128.38 (Ar CH), 128.3 (Ar CH), 128.2 (Ar CH), 127.9 (Ar CH), 127.7 (Ar CH), 127.6 (Ar CH), 127.3 (Ar CH), 92.7 (C-1), 74.3 (C-3), 74.2 (*C*H$_2$Ph), 73.5 (*C*H$_2$Ph), 73.2 (C-4), 70.5 (*C*H$_2$Ph), 70.13 (C-5), 70.10 (C-6), 31.0 (C-2).

NMR data for β-anomer: ^{1}H NMR (400 MHz, Chloroform-*d*) δ 7.38 – 7.29 (15 H, m, Ar*H*), 4.95 (1 H, d, *J* = 11.7 Hz, C*H*$_2$Ph), 4.73 (1 H, ddd, *J* = 9.6, 7.7, 2.2 Hz, H-1), 4.66–4.62 (3 H, m, 3 x C*H*$_2$Ph), 4.52 (1 H, d, *J* = 11.9 Hz, C*H*$_2$Ph), 4.46 (1 H, d, *J* = 12.0 Hz, C*H*$_2$Ph), 3.84–3.83 (1 H, m, H-4), 3.66 (1 H, dd, *J* = 9.0, 6.2 Hz, H-6a), 3.63–3.54 (3 H, m, H-3, H-5, H-6), 3.26 (1 H, d, *J* = 7.6 Hz, O*H*), 2.19–2.14 (1 H, m, H-2a), 2.06–2.01 (1 H, m, H-2b); ^{13}C NMR (101 MHz, $CDCl_3$) δ 138.7 (Ar C), 137.9 (Ar C), 136.5 (Ar C), 128.5 (Ar CH), 128.3 (Ar CH), 128.2 (Ar CH), 127.9 (Ar CH), 127.8 (Ar CH), 127.7 (Ar CH), 127.3 (Ar CH), 94.8 (C-1), 77.2 (C-3), 74.3 (*C*H$_2$Ph), 73.6 (*C*H$_2$Ph), 71.7 (C-4), 70.3 (*C*H$_2$Ph), 70.11 (C-5), 69.3 (C-6), 34.5 (C-2).

Data was in agreement with previously reported synthesis.[2, 3]

3,4,6-Tri-*O*-acetyl-2-deoxy-α/β-D-galactopyranose 2b

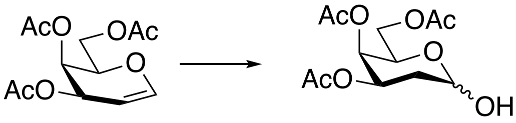

S1

Prepared according to the procedure reported by Gilmour *et. al*[6] Tri-*O*-acetyl-D-galactal **S1** (4.5 g, 16.5 mmol) was dissolved in CH_3CN (130 mL) and Amberlite IR-120 (4.5 g, pre-washed with MeCN x 3), LiBr (4.5 g, 51.8 mmol) and H_2O (4.5 mL) were subsequently added. The reaction mixture was stirred at room temperature for 1 hour. The reaction was then filtered. A mixture of EtOAc and sat. aq. $NaHCO_3$ were added, the organic layer was extracted, dried (Na_2SO_4), filtered and concentrated *in vacuo*. The crude product was purified to flash chromatography (3:2 Hexane/EtOAc) to give the *title compound* **2b** (3.54 g, 12.2 mmol, 74%, α/β = 3:1) as a pale yellow oil; $R_f = 0.27$ (1:1 Hexane/EtOAc); MS (ES^+) found *m/z* 313.1 $[M+Na]^+$; HRMS (ESI) m/z: Calcd $C_{12}H_{18}O_8Na$ $[M+Na]^+$ 313.0899; Found 313.0902.

NMR data for α-anomer:^1H NMR (400 MHz, Chloroform-*d*) δ 5.52 (1H, t, J = 2.7 Hz, H-1), 5.41–5.37 (2 H, m, H-3, H-4), 4.43 (1 H, t, J = 6.6 Hz, H-5), 4.18–4.1 (2 H, m, 2 x H-6), 2.89 (1 H, dd, J = 3.0, 2.3 Hz, O*H*), 2.15 (3 H, s, C(O)C*H*3), 2.11–2.08 (1 H, m, H-2), 2.08 (3 H, s, C(O)C*H*3), 2.01 (3 H, s, C(O)C*H*3), 1.97–1.91 (1 H, m, H-2); ^{13}C NMR (101 MHz, $CDCl_3$) δ 170.6 (C=O), 170.3 (C=O), 170.1 (C=O), 92.3 (C-1), 68.80 (C-4 or C-5), 68.79 (C-4 or C-5), 65.8 (C-3), 62.6 (C-6), 30.1 (C-2), 20.9 (C(O)*C*H3), 20.8 (C(O)*C*H3), 20.7 (C(O)*C*H3).

NMR data for β-anomer: ^1H NMR (400 MHz, Chloroform-*d*) δ 5.28 (1 H, d, J = 3.1 Hz, H-4), 5.03 (1 H, ddd, J = 12.6, 4.9, 3.2 Hz, H-3), 4.94 (1 H, ddd, J = 9.7, 6.3, 2.3 Hz, H-1), 4.18–4.1 (2 H, m, 2 x H-6), 3.88 (1 H, td, J = 6.5, 1.1 Hz, H-5), 3.44 (1 H, d, J = 6.3 Hz, O*H*), 2.17 (3 H, s, C(O)C*H*3), 2.11–2.08 (1 H, m, H-2), 2.08 (3 H, s, C(O)C*H*3), 2.03 (3 H, s, C(O)C*H*3), 1.97–1.91 (1 H, m, H-2); ^{13}C NMR (101 MHz, $CDCl_3$) δ 170.6 (C=O), 170.3 (C=O), 170.1 (C=O), 94.3 (C-1), 71.3 (C-5), 68.3 (C-3), 65.3 (C-4), 62.2 (C-6), 33.2 (C-2), 20.8 (C(O)*C*H3), 20.73 (C(O)*C*H3), 20.69 (C(O)*C*H3).

Data in agreement with previously reported synthesis. [3]

3,4,6-Tri-*O*-benzyl-2-deoxy-2-fluoro-α/β-D-galactopyranose 2c

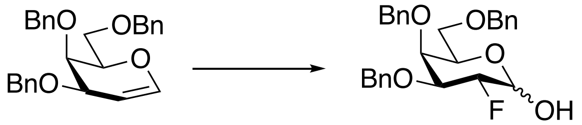

Prepared according to the procedure reported by Gilmour and co-workers.[6]
To a solution of **1** (2.5 g, 6.0 mmol) in acetone (30 mL) was added water (5.5 mL) and Selectfluor™ (2.6 g, 7.34 mmol). After the reaction mixture was stirred at room temperature for 5 days it was concentrated *in vacuo*, diluted with CH_2Cl_2/sat. aq. $NaHCO_3$ (200mL/50mL) and extracted with CH_2Cl_2 three times. The crude product was purified by silica gel flash column chromatography (2 columns: first column 2:1 Hexane/EtOAc, second column 4:1 Hexane/EtOAc) to give the title compound as a pale yellow oil (1.20 g, 2.65 mmol, 44%, α/β = 2.5:1); R_f= 0.18 (2:1 Hexane/EtOAc) and 0.14 (2:1 Hexane/EtOAc);

NMR data for α-anomer: ^{1}H NMR (400 MHz, Chloroform-*d*) δ 7.42–7.28 15H, m, Ar*H*), 5.47 (1 H, t, *J* = 3.5 Hz, H-1), 4.98 (1 H, ddd, *J* = 50.4, 9.7, 3.9 Hz, H-2), 4.95 (1 H, d, *J* = 11.4, C*H*$_2$Ph), 4.84 (1 H, d, *J* = 12.0, C*H*$_2$Ph), 4.72 (1 H, d, *J* = 12.0, C*H*$_2$Ph), 4.63–4.49 (2 H, m, 2 x C*H*$_2$Ph), 4.45 (1 H, d, *J* = 12.0, C*H*$_2$Ph), 4.21 (1 H, t, *J* = 6.3 Hz, H-5), 4.06 (1 H, td, *J* = 10.2, 2.8 Hz, H-3), 3.97 (1 H, t, *J* = 3.2 Hz, H-4), 3.65–3.54 (1 H, m, H-6), 3.46 (1 H, dd, *J* = 9.4, 5.9 Hz,H-6), 3.31 (1 H, br s, O*H*); ^{13}C NMR (101 MHz, Chloroform-*d*) δ 138.3 (Ar C), 138.2 (Ar C), 137.7 (Ar C), 128.5 (Ar CH), 128.4 (Ar CH), 128.33 (Ar CH), 128.29 (Ar CH), 128.0 (Ar CH), 127.74 (Ar CH), 127.70 (Ar CH), 127.5 (Ar CH), 91.0 (d, *J* = 21.7 Hz, C-1), 89.6 (d, *J* = 186.4 Hz, C-2), 76.7 (d, *J* = 14.1 Hz, C-3), 75.6 (d, *J* = 8.5 Hz, C-4), 74.8 (*C*H$_2$Ph), 73.5 (*C*H$_2$Ph), 73.0 (d, *J* = 26.5, *C*H$_2$Ph), 69.6 (C-5), 69.0 (C-6); ^{19}F NMR (376 MHz, Chloroform-*d*) δ -206.8.

NMR data β-anomer: ^{1}H NMR (400 MHz, Chloroform-*d*) δ7.42–7.28 (15 H, m, Ar*H*), 4.95 (1 H, d, *J* = 11.4, C*H*$_2$Ph), 4.81 (1 H, d, *J* = 12.0, C*H*$_2$Ph), 4.77–4.63 (2 H, m, H-1, H-2), 4.63–4.49 (3 H, m, 3 x C*H*$_2$Ph), 4.44 (1 H, d, *J* = 11.8, C*H*$_2$Ph), 3.94 (1 H, t, *J* = 3.2 Hz, H-4), 3.71 (1 H, d, *J* = 6.9 Hz, O*H*), 3.65–3.54 (4 H, m, H-3, H-5, 2 x H-6); ^{13}C NMR (101 MHz, Chloroform-*d*) δ 138.0 (Ar C), 137.9 (Ar C), 137.6 (Ar C), 128.5 (Ar CH), 128.0 (Ar CH), 127.9 (Ar CH), 127.84 (Ar CH), 127.82 (Ar CH), 127.77 (Ar CH), 127.6 (Ar CH), 95.2 (d, *J* = 24.3 Hz, C-1), 93.3 (d, *J* = 183.2 Hz, C-2), 80.1 (d, *J* = 15.9 Hz, C-3), 74.9 (*C*H$_2$Ph), 74.4 (d, *J* = 8.8 Hz, C-4), 73.9 (C-5), 73.6 (*C*H$_2$Ph), 73.0 (d, *J* = 26.5, *C*H$_2$Ph), 68.5 (C-6), ^{19}F NMR (376 MHz, Chloroform-*d*) δ -204.7.

Data in agreement with previously reported synthesis.[3, 4]

6-O-Acetyl-3,4-di-O-benzyl-2-deoxy-α/β-D-galactopyranose 2d and 3,4-Di-O-benzyl-2-deoxy-α/β-D-galactopyranose S3

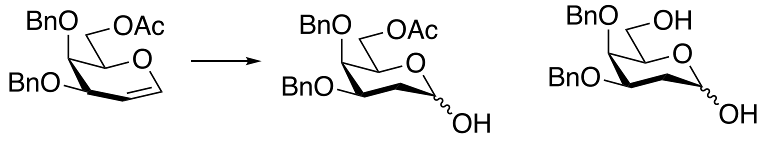

S2 **2d** **S3**

To a solution of **S2**[5] (1.95 g, 5.05 mmol) in THF/H_2O (40 mL, 9:1 *v:v*) was added aq. HCl (36%, 0.25 mL). The reaction was stirred for 22h, upon which another portion of aq. HCl (36%, 0.25 mL) was added and the reaction stirred for a further 18 h. Solid $NaHCO_3$ was added to neutralise (pH ~7) and the reaction mixture was concentrated *in vacuo.* CH_2Cl_2 was added, the mixture filtered and the filtrate purified by silica gel flash column chromatography (1:1 Hex/EtOAc) to give the title compound **2d** (1.0 g, 2.59 mmol, 52%, α/β = ~3:1) plus diol **S3** (121 mg, 0.35 mmol, 7%, α/β = ~2.5:1).

Data for α-**2d**: R_f= 0.24 (Hexane/EtOAc 1:1); MS (ES^+) found *m/z* 409.2 $[M+Na]^+$, HRMS (ESI) m/z: Calcd $C_{22}H_{26}O_6Na$ $[M+Na]^+$ 409.1622; Found 409.1628. ^1H NMR (400 MHz, Chloroform-*d*) δ 7.41–7.32 (10 H, m, Ar*H*), 5.49 (1 H, br s, H-1), 5.00 (1 H, dd, *J* = 11.6 Hz, C*H*$_2$Ph), 4.71–4.63 (3 H, m, 3 x C*H*$_2$Ph), 4.24–4.12 (3 H, m, H-5, 2 x H-6), 4.03 (1 H, ddd, *J* = 12.0, 4.5, 2.4 Hz, H-3), 3.86 (1 H, br s, H-4), 2.22 (1 H, ddd, *J* = 12.3, 3.2, 2.0 Hz, H-2_{eq}), 2.07–2.02 (1 H, m, H-2_{ax}), 2.01 (3 H, d, C(O)C*H*$_3$); ^{13}C NMR (101 MHz, Chloroform-*d*) δ 171.3 (C=Oβ), 170.8 (C=Oα), 138.4 (2 x Ar C), 128.5 (Ar CH), 128.4 (Ar CH), 128.3 (Ar CH), 127.74 (Ar CH), 127.65 (Ar CH), 127.3 (Ar CH), 92.6 (C-1), 74.2 (C-3), 74.1 (*C*H$_2$Ph), 72.8 (C-4), 70.6 (*C*H$_2$Ph), 69.2 (C-5), 64.3 (C-6), 63.9 (C-6), 30.9 (C-2), 20.8 (C(O)*C*H$_3$); Data for β-**2d**: ^1H NMR (400 MHz, Chloroform-*d*) δ 7.41–7.32 (10 H, m, Ar*H*), 4.99 (1 H, d, *J* = 11.5 Hz, C*H*$_2$Ph), 4.71–4.63 (4 H, m, H-1, 3 x C*H*$_2$Ph), 4.24–4.12 (3 H, m, 2 x H-6), 3.80–3.76 (1 H, m, H-4), 3.63–3.56 (2 H, m, H-3, H-5), 2.22–2.15 (1 H, m, H-2), 2.07–2.02 (1 H, m, H-2), 2.02 (3 H, s, C(O)C*H*$_3$); ^{13}C NMR (101 MHz, Chloroform-*d*) 138.3 (Ar C), 138.1 (Ar C), 128.5 (Ar CH), 127.81 (Ar CH), 127.78 (Ar CH), 127.4 (Ar CH), 94.8 (C-1), 77.1 (C-5), 74.2 (*C*H$_2$Ph), 73.0 (C-3), 71.5 (C-4), 70. 5 (*C*H$_2$Ph), 63.9 (C-6), 34.4 (C-2), 20.9 (C(O)*C*H$_3$).

Data for α-**S3**: R_f= 0.07 (Hexane/EtOAc 2:1); MS (ES^+) found *m/z* 367.5 $[M+Na]^+$, HRMS (ESI) m/z: Calcd $C_{20}H_{24}O_5Na$ $[M+Na]^+$ 367.1521; Found 367.1510, $[M+Na]^+$ requires; ^1H NMR (400 MHz, Chloroform-*d*) δ 7.39–7.31 (10 H, m, Ar*H*), 5.44 (1 H, d, *J* = 2.9 Hz, H-1), 4.95 (1 H, d, *J* = 11.7 Hz, C*H*$_2$Ph), 4.67–4.60 (3 H, m, 3 x C*H*$_2$Ph), 4.01–3.97 (2 H, m, H-3, H-5), 3.88–3.79 (2 H, m, H-4, H-6a), 3.46 (1 H, dd, *J* = 11.5, 3.8 Hz, H-6b), 2.19 (1 H, dt, 12.2, 6.2 Hz, H-2_{eq}), 2.04–1.99 (1 H, m, H-2); ^{13}C NMR (101 MHz, Chloroform-*d*) δ 138.5 (Ar C), 138.4 (Ar C), 128.51 (Ar CH), 128.47 (Ar CH), 128.4 (Ar CH), 127.8 (Ar CH), 127.6 (Ar CH), 127.3 (Ar CH), 92.4 (C-1), 74.5 (CH), 74.0 (*C*H$_2$Ph), 73.3 (C-4), 71.4 (CH), 70.6 (*C*H$_2$Ph), 63.4 (C-6), 31.1 (C-2); Data for β-**S3**: ^1H NMR (400 MHz, Chloroform-*d*) δ 7.39–7.31 (10 H, m, Ar*H*), 4.93 (1 H, d, *J* = 11.7 Hz, C*H*$_2$Ph), 4.67–4.60 (4 H, m, H-1, 3 x C*H*$_2$Ph), 3.88–3.79 (1 H, m, H-6a), 3.69 (1 H, t, *J* = 1.2 Hz, H-4), 3.53 (1 H, ddd, *J* = 12.0, 4.4, 2.6 Hz, H-3), 3.49–3.43 (1 H, m, H-6b), 3.35 (1 H, ddd, 7.7, 4.0, 0.9 Hz, H-5), 2.22–2.11 (1 H, m, H-2), 2.04–1.99 (1 H, m, H-2); ^{13}C NMR (101 MHz, Chloroform-*d*) δ 138.2 (Ar C), 138.1 (Ar C), 128.5 (Ar CH), 127.9 (Ar CH), 127.8 (Ar CH), 127.6 (Ar CH), 127.4 (Ar CH),

94.7 (C-1), 77.3 (C-3), 75.7 (C-5), 74.1 (CH_2Ph), 71.9 (C-4), 70.5 (CH_2Ph), 62.7 (C-6), 34.3 (C-2).

3,4,6-Tri-O-benzyl-2-deoxy-α/β-D-glucopyranose 3a

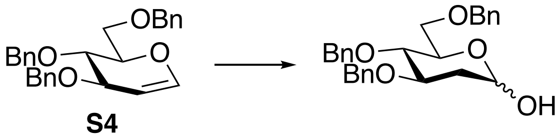

Concentrated 36% HCl (1.5 mL) was added to a solution of tri-*O*-benzyl glucal **S4** (9.0 g, 21.6 mmol) in a mixture of THF and H_2O (200 mL, 9:1). The reaction was stirred at RT for 24 hr and a further portion of concentrated 36% HCl (1.5 mL) was added**.** After a further 24 h solid $NaHCO_3$ was added until a pH of 7 was reached. The reaction mixture was concentrated to remove the THF, H_2O was added and the resultant white solid was filtered. Purification by flash column chromatography (6:1 to 1:1 Hexane/EtOAc) was necessary to removal trace starting material to give title compound **3a** as a white powder (7.16 g, 16.5 mmol, 76%, α/β = 2.5:1); R_f = 0.29 (3:2 Hexane/EtOAc); MS (ES^+) found *m/z* 457.2 $[M+Na]^+$.

NMR data for α-anomer: ^{1}H NMR (400 MHz, Chloroform-*d*) δ 7.40–7.22 (15 H, m, Ar*H*), 5.43 (1 H, br t, *J* = 2.5 Hz, H-1), 4.95 (1 H, d, *J* = 11.0 Hz, CH_2Ph), 4.70–4.54 (5 H, m, 5 x CH_2Ph), 4.12–4.06 (2 H, m, H-3, H-5), 3.72–3.70 (2 H, m, 2 x H-6), 3.54 (1 H, dd, *J* = 9.8, 9.0 Hz, H-4), 3.26 (1 H, dd, *J* = 2.9, 2.3 Hz, O*H*), 2.32 (1 H, ddd, *J* = 13.0, 5.0, 1.3 Hz, H-2_{eq}), 1.72 (1 H, *J* = 13.0, 11.3, 3.6, 2.0 Hz, H-2_{ax}); ^{13}C NMR (101 MHz, $CDCl_3$) δ 138.7 (Ar C), 138.5 (Ar C), 138.0 (Ar C), 128.41 (Ar CH), 128.39 (Ar CH), 128.3 (Ar CH), 128.0 (Ar CH), 127.9 (Ar CH), 127.71 (Ar CH), 127.68 (Ar CH), 92.1 (C-1), 78.7 (C-4), 77.1 (C-3 or C-5), 74.9 (CH_2Ph), 73.6 (CH_2Ph), 71.8 (CH_2Ph), 70.7 (C-3 or C-5), 69.4 (C-6), 35.6 (C-2).

NMR data for β-anomer: ^{1}H NMR (400 MHz, Chloroform-*d*) δ 7.40–7.22 (15 H, m, Ar*H*), 4.93 (1 H, d, *J* = 10.9 Hz, CH_2Ph), 4.70–4.54 (6 H, m, H-1, 5 x CH_2Ph), 3.98 (1 H, d, *J* = 6.3 Hz, O*H*), 3.76 (1 H, dd, *J* = 10.4, 1.7 Hz, H-6a), 3.72–3.70 (1 H, m, H-6b), 3.66–3.61 (1 H, m, H-3), 3.51–3.49 (2 H, m, H-4, H-5), 2.37–2.32 (1 H, m, H-2_{eq}), 1.61 (1 H, *J* = 12.5, 11.6, 9.7 Hz, H-2_{ax}); ^{13}C NMR (101 MHz, $CDCl_3$) δ 138.5 (Ar C), 138.32 (Ar C), 138.30 (Ar C), 128.5 (Ar CH), 128.4 (Ar CH), 128.0 (Ar CH), 127.7 (Ar CH), 127.62 (Ar CH), 127.59 (Ar CH), 94.2 (C-1), 79.3 (C-3), 77.9 (C-4 or C-5), 74.8 (C-4 or C-5), 73.5 (CH_2Ph), 71.5 (CH_2Ph), 69.3 (C-6), 37.9 (C-2).

Data was in agreement with previously reported synthesis.[6]

3,4,6-Tri-*O*-acetyl-2-deoxy-α/β-D-glucopyranose 3b

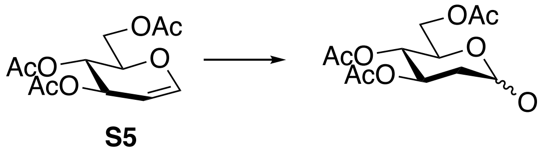

Prepared according to the procedure reported by Bucher and Gilmour.[6] Tri-*O*-acetyl-D-glucal **S5** (5 g, 18.4 mmol) was dissolved in CH_3CN (60 mL) and Amberlite IR-120 (5

g, pre-washed with MeCN x 3), LiBr (5 g, 57.5 mmol) and H_2O (1.5 mL) were subsequently added. The reaction mixture was stirred at room temperature for 1 hour. The reaction was then filtered. The filtrate was neutralised by the addition of Et_3N and concentrated *in vacuo.* A mixture of CH_2Cl_2 and water was added, the organic layer was extracted, washed with ice cold 1M HCl, sat. aq. $NaHCO_3$, dried (Na_2SO_4), filtered and concentrated *in vacuo*. The crude product was purified to flash chromatography (3:2 to 2:3 hexane/EtOAc) followed by dissolution in the min volume of Et_2O and the addition of hexane. The resultant suspension was filtered to give the *title compound* **3b** (3.3 g, 12 mmol, 63%, α/β = 10:1) as small white needles; R_f = 0.18 (3:2 Hexane/EtOAc); MS (ES^+) found *m/z* 313.1 $[M+Na]^+$; HRMS (ESI) m/z: Calcd $C_{12}H_{18}O_8Na$ $[M+Na]^+$ 313.089; Found 313.0890.

NMR data for α-anomer: ^1H NMR (400 MHz, Chloroform-*d*) δ 5.43 (1 H, app t, J = 3.0 Hz, H-1), 5.38 (1 H, ddd, J = 11.7, 9.6, 5.4 Hz, H-3), 5.01 (1 H, t, J = 9.7 Hz, H-4), 4.27 (1 H, dd, J = 11.9, 4.5 Hz, H-6), 4.22 (1 H, ddd, J = 10.1, 4.8, 1.9 Hz, H-5), 4.09 (1 H, dd, J = 12.0, 2.0 Hz, H-6), 2.75 (1 H, dd, J = 3.3, 2.2 Hz, O*H*), 2.28 (1 H, ddd, J = 13.0, 5.3, 1.4 Hz, H-2_{eq}), 2.09 (3 H, s, C(O)CH_3), 2.05 (3 H, s, C(O)CH_3), 2.02 (3 H, s, C(O)CH_3), 1.82 (1 H, dddd, J = 13.1, 11.6, 3.6, 2.2 Hz, H-2_{ax}); ^{13}C NMR (101 MHz, $CDCl_3$) δ 170.8 (C=O), 170.2 (C=O), 169.9 (C=O), 91.7 (C-1), 69.4 (C-4), 68.7 (C-3), 68.1 (C-5), 62.5 (C-6), 35.1 (C-2), 21.0 (*C*H_3), 20.8 (*C*H_3), 20.8 (*C*H_3).

Selected signals for β-anomer: ^1H NMR (400 MHz, Chloroform-*d*) δ 2.44 (1 H, ddd, J = 12.7, 4.9, 2.1 Hz, H-2_{eq}), 1.77–1.68 (1 H, m, H-2_{ax}).

Data in agreement with previously reported synthesis.[6]

List of glycosyl acceptors used for the synthesis of 2-deoxy glycosides (Table 1)

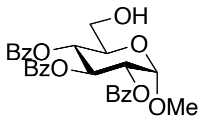

4a

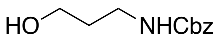

4b

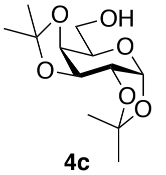

4c

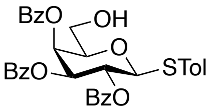

4d

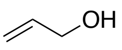

4e

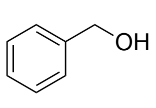

4f

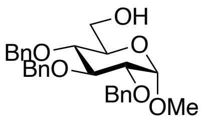

4g

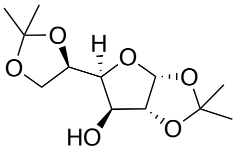

4h

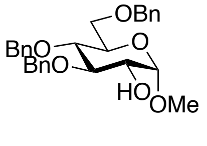

4i

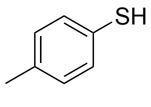

4j

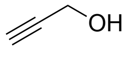

4k

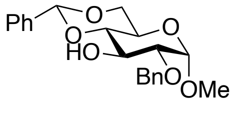

4l

4b, **4c**, **4e**, **4f**, **4h**, **4j**, **4k** were commercially available.

The preparation of acceptors **4a**, **4g** and **4l** has previously been described by Galan *et al.*[5]
Acceptor **4i** was prepared according to the procedure reported by Lecourt *et al.*[7]

2,3,4-Tri-*O*-benzoyl-1-*p*-methylphenylthio-β-D-galactopyranose 4d

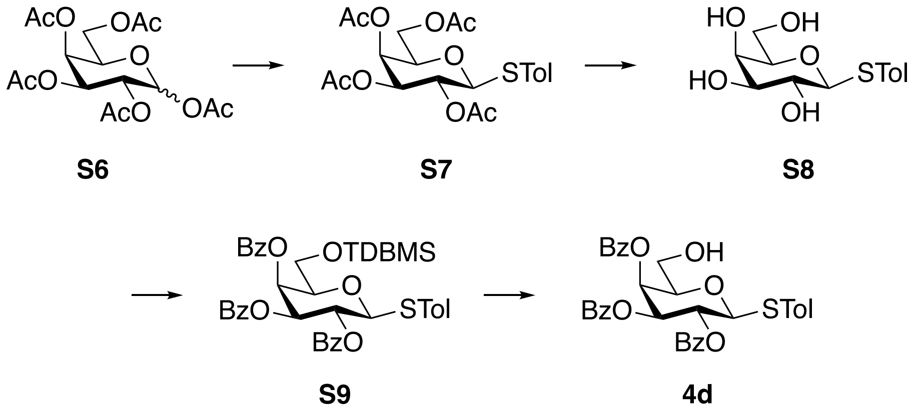

Tetraol **S8** was prepared according to the procedure reported by Varga-Berenguel *et al.*[8] TBDMS intermediate **S9** and acceptor **4d** were prepared according to the procedure reported by Zhang *et al.*[9]

Step 1: $BF_3.Et_2O$ (36.4 mL, 0.295 mmol) was added to a solution of per-OAc galactose **S6** (100 g, 0.256 mol) and 4-thiocresol (35 g, 0.282 mol) in anhydrous CH_2Cl_2 (500 mL) at 0 °C under N_2. The stirring reaction mixture was allowed to warm to room temperature and after 26 h the reaction mixture was slowly poured in sat. aq. $NaHCO_3$. When effervescence had ceased I_2 was added until the red colour persisted. After stirring for 20 minutes solid $Na_2S_2O_3$ was added portionwise until the dark-red colour disappeared. The organic layer was separated, dried, filtered and concentrated *in vacuo*. The resultant crude solid was stirred with hexane and the resultant precipitate filtered to give tetra-acetate **S7** (90.7 g, 0.200 mol, 78%). Data in agreement with previously reported synthesis.[10]

Step 2: To a suspension of **S7** (90.7 g, 0.200 mol) in MeOH (200 mL) was added NaOH (1.6 g, 40 mmol). After stirred for 30 minutes at room temperature the reaction was neutralised by the addition of Amberlite IR120 acidic resin. The resin was removed by filtration and the filtrate concentrated to give tetraol **S8** (54.4 g, 0.190 mmol, 95%).

Steps 3 and 4: To a solution of **S8** (5 g, 17.5 mmol) in pyridine (50 mL) was added TBDMSCl (7.25 mL, 21.0 mmol). The reaction was stirred at RT for 17 h upon which the reaction was judged complete by TLC whereupon BzCl (8.1 mL, 69.9 mmol) was added dropwise. The reaction was stirred for 4 h, then diluted with EtOAc (250 mL), and sat. aq. $NaHCO_3$ (60 mL) added. The organic layer was separated, washed with 1 M aq. HCl (3 x 30 mL), dried, filtered and concentrated *in vacuo*. The crude product was purified by silica gel flash column chromatography (9:1 Hexane/EtOAc) to give the fully protected intermediate **S9** which was used in the next step without further purification. To 6-*O*-TBDMS protected galactose **S9** was added TBAF (61 mL, 1 M in THF) followed by the addition glacial acetic acid until a pH of 6 was reached. The reaction was left to stir for 2 h and EtOAc (100 mL) and sat. aq. NH_4Cl (50 mL) were

added. The organic layer was seprated, washed with sat. aq. NaCl (100 mL), dried, filtered and concentrated *in vacuo*. The crude product was purified by silica gel flash column chromatography (1:1 Hexane/EtOAc) to give the *title compound* **4d** (2.8 g, 9.8 mmol, 56% over three steps). Data in agreement with previously reported synthesis.[10]

General procedure A - $AuCl_3$ catalysed glycosylation reactions

Hemiacetal donor and acceptor were combined in a vial/RBF and dissolved in either EtOAc or toluene (non-anhydrous solvents used). $AuCl_3$ was added, the vial/RBF sealed and the reaction was then heated to 50 °C. When the reaction was deemed complete by TLC the reaction mixture was quenched via the addition of a sat. aq. $NaHCO_3$ and sat. aq. $Na_2S_2O_3$ and the diluted with EtOAc. The organic layers was separated, dried ($MgSO_4$), filtered and concentrated *in vacuo*. The resulting crude products were purified using flash chromatography.

Reaction optimisation/screens

All reactions were performed according to general procedure A.
Ethyl acetate was initially identified and used as the reaction solvent (Tables S1 and S2). When chloroform was used complete conversion to the anomeric benzyl derivative **5f** was observed (Table S1, Entry 5). Crude ^{1}H NMR was compared to data available for **5f** the synthesis of which we have previously reported. [11] [12] [13] [14]

It was found that although higher temperatures did improve the reaction yields there was not a dramatic difference between 50 °C and 70 °C (Table S3). Following the discovery that EtOAc could lead to by-product formation (Scheme 3) a further solvent screen was carried out (Table S4). Toluene and *tert*-butyl methyl ether gave the highest yields. Due to the lower boiling point of TBME toluene was selected as the reaction solvent from then on.

Table S1 – Solvent screen using 10 mol% $AuCl_3$

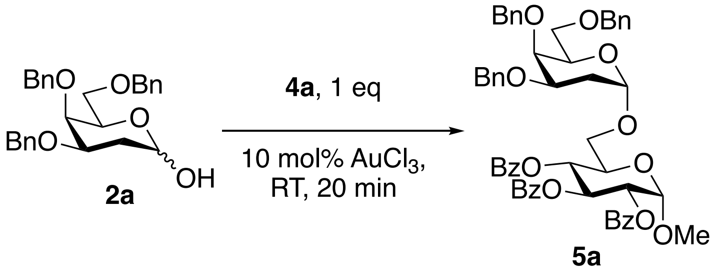

Entry	Solvent	Yield **5a**
1	Acetone	46
2	**EtOAc**	**64**
3	DCM	44
4	Et_2O	29
5	$CHCl_3$	0**

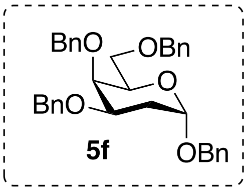

*Calculated from crude ^{1}H NMR spectrum
Complete conversion to anomeric benzyl derivative **5f

Table S2 – Solvent screen using 1 mol% $AuCl_3$

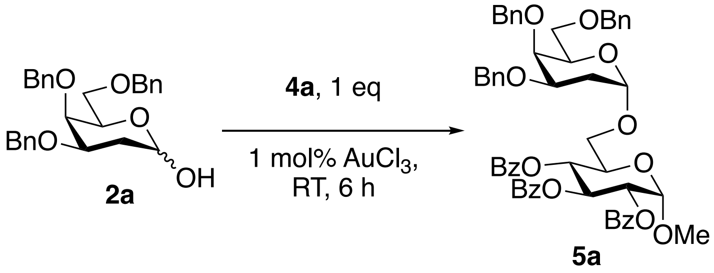

Entry	Solvent	Yield **5a***
1	Acetone	0
2	**EtOAc**	**59**
3	DCM	0
4	Et_2O	13

*Calculated from crude ^{1}H NMR spectrum

Table S3 – Effect of temperature

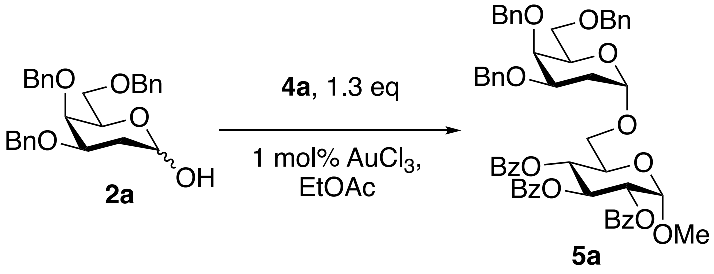

Entry	Temperature	Time (h)	Yield **5a***
1	50 °C	1	74
2	50 °C	18	78
3	70 °C	2	58
4	70 °C	4	73
5	70 °C	24	83

*Calculated from crude ^{1}H NMR spectrum

Table S4 – Solvent screen at 50 °C

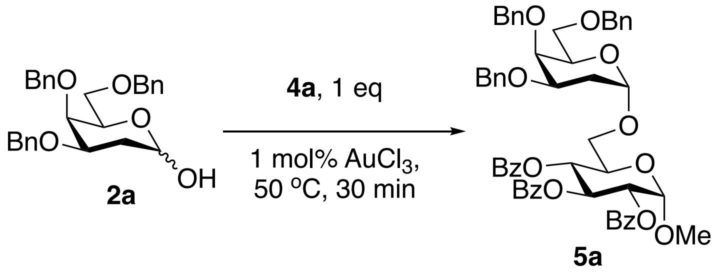

Entry	Solvent	Yield **5a***	Entry	Solvent	Yield **5a***
1	EtOAc	63	6	**TBME**	**72**
2	Acetone	53	7	THF	63
3	**PhCl**	**69**	8	**Toluene**	**72**
4	MeCN	61	9	1,4-dioxane	50
5	1,2-DCE	65	10	$MeNO_2$	No reaction

*Calculated from crude ^{1}H NMR spectrum

Synthesis of 2-deoxy glycosides (Table 1)

Methyl 2,3,4-tri-*O*-benzyl-6-*O*-(3,4,6-tri-*O*-benzyl-α-D-2-deoxy-galactopyranosyl)-α-D-glucopyranoside 5a

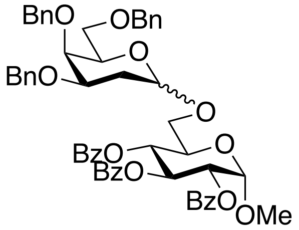

Prepared according to general procedure A from donor **2a** (26 mg, 0.060 mmol), acceptor **4a** (60 mg, 0.119 mmol), $AuCl_3$ (0.3 mg, 0.001 mmol) and EtOAc (0.4 mL). After 3 h, and following work up and purification via silica gel flash column chromatography (3:1 Hexane/EtOAc) gave *the title compound* **5a** as a yellow oil (39 mg, 0.042 mmol, 70%, α/β = 12:1); R_f = 0.33 (3:1 Hexane/EtOAc); MS (ES$^+$) found m/z = 945.6 $[M+Na]^+$.

NMR data for α-anomer: ^{1}H NMR (400 MHz; $CDCl_3$): δ 8.02 (2 H, d, J = 7.3 Hz, Ar*H*), 7.96 (2 H, d, J = 7.3 Hz, Ar*H*), 7.91 (2 H, d, J = 7.3 Hz, Ar*H*), 7.54–7.21 (24 H, m, Ar H), 6.16 (1 H, t, J = 9.9 Hz, H-3_{Glc}), 5.68 (1 H, t, J = 9.9 Hz, H-4_{Glc}), 5.32 (1 H, dd, J = 10.3, 3.6 Hz, H-2_{Glc}), 5.23 (1 H, d, J = 3.6 Hz, H-1_{Glc}), 5.03 (1 H, d, J = 3.0 Hz, H-1_{2DGal}), 4.93 (1 H, d, J = 11.6 Hz, CH_2Ph), 4.65–4.59 (3 H, m, 3 x CH_2Ph), 4.36 (1 H, d, J = 11.9 Hz, CH_2Ph), 4.28 (1 H, d, J = 11.9 Hz, CH_2Ph), 4.23 (1 H, dt, J = 10.2, 3.7 Hz, H-5_{Glc}), 3.98 (1 H, ddd, J = 11.8, 4.1, 2.1 Hz, H-3_{2DGal}), 3.92–3.85 (3 H, m, H-4_{2DGal}, H-5_{2DGal}, H-6_{Glc}), 3.62 (1 H, dd, J = 11.1, 2.6 Hz, H-6_{Glc}), 3.52–3.48 (2 H, m, 2 x H-6_{2DGal}), 3.43 (3 H, s, OCH_3), 2.22 (1 H, td, J = 12.4, 3.5 Hz, H-$2_{2DGal\ ax}$), 2.01 (1 H, dd, J = 12.8, 4.5 Hz, H-$2_{2DGal\ eq.}$); ^{13}C NMR (101 MHz; $CDCl_3$): δ 165.9 (C=O), 165.8 (C=O), 165.3 (C=O), 138.9 (Ar C), 138.6 (Ar C), 138.2 (Ar C), 133.4 (Ar C), 133.3 (Ar C), 133.0 (Ar C), 130.0 (Ar *C*H), 129.9 (Ar *C*H), 129.8 (Ar *C*H), 129.7 (Ar *C*H), 129.3 (Ar *C*H), 129.2 (Ar CH), 129.1 (Ar CH), 128.41 (Ar *C*H), 128.38 (Ar *C*H), 128.36 (Ar *C*H), 128.3 (Ar *C*H), 128.2 (Ar *C*H), 128.1 (Ar *C*H), 127.52 (Ar *C*H), 127.47 (Ar *C*H), 127.44 (Ar *C*H), 127.43 (Ar *C*H), 98.1 (C-1_{2DGal}), 97.0 (C-1_{Glc}), 74.5 (C-3_{2DGal}), 74.3 (*C*H_2Ph), 73.2 (*C*H_2Ph), 73.1 (C-4_{2DGal}), 72.1 (C-2_{Glc}), 70.7 (C-3_{Glc}), 70.4 (*C*H_2Ph), 69.9 (C-5_{2DGal}), 69.5 (C-4_{Glc}), 69.4 (C-6_{2DGal}), 68.2 (C-5_{Glc}), 65.8 (C-6_{Glc}), 55.5 (O*C*H_3), 30.9 (C-2_{2DGal}).

Data in agreement with previously reported synthesis. [15, 16]

3,4,6-Tri-*O*-benzyl-1-(3-carboxybenzyl-amino-1-propanol)-2-deoxy-D-galactopyranose 5b

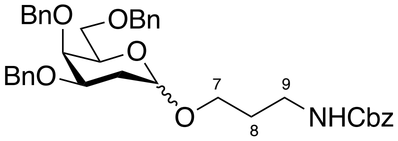

Prepared according to general procedure A from donor **2a** (26 mg, 0.06 mmol), benzyl (3-hydroxypropyl)carbamate **4b** (19 mg, 0.090 mmol) and $AuCl_3$ (0.2 mg, 0.0007 mmol) and EtOAc (0.4 mL). After 3 hours, and following work up and purification via silica gel flash column chromatography (7:1 to 3:1 Hexane/EtOAc) gave *the title compound* **5b** as a colourless oil (26.8 mg, 0.042 mmol, 71%, α/β = 6:1); R_f= 0.32 (5:1 Hexane/EtOAc); MS (ES$^+$) found m/z = 648.3 [M+Na]$^+$, HRMS (ESI) m/z: Calcd $C_{38}H_{43}NO_7Na$ [M+Na]$^+$ 648.29232; Found 648.2919.

NMR data for α-anomer: ^{1}H NMR (400 MHz; $CDCl_3$): *δ* 7.37–7.26 (20 H, m, Ar H), 5.42 (1 H, br. s, N*H*), 5.11 (2 H, s, CH_2Ph), 4.99 (1 H, d, *J* = 2.9 Hz, H-1), 4.94 (1 H, d, *J* = 11.7 Hz, CH_2Ph), 4.64–4.53 (4 H, m, 4 x CH_2Ph), 4.44 (1 H, d, *J* = CH_2Ph), 3.93–3.75 (4 H, m, H-3, H-4, H-5, 1 x H-7a), 3.59 (1 H, dd, *J* = 9.5, 6.7 Hz, H-6a), 3.51–3.46 (2 H, m, H-7b, H-6b), 3.42–3.22 (2 H, m, 2 x H-9), 2.25 (1 H, td, *J* = 12.3, 12.3, 3.7 Hz, H-2_{ax}), 1.98 (1 H, *J* = 12.6, 4.3 Hz, H-2_{eq}), 1.80 (2 H, dt, *J* = 11.6, 5.7 Hz, 2 x H-8); ^{13}C NMR (101 MHz; $CDCl_3$): *δ* 156.4 (C=O), 138.8 (Ar C), 138.5 (Ar C), 138.0 (Ar C), 136.7 (Ar C), 128.5 (Ar *C*H), 128.41 (Ar *C*H), 124.39 (Ar *C*H), 128.3 (Ar *C*H), 128.23 (Ar *C*H), 128.16 (Ar *C*H), 128.1 (Ar *C*H), 127.9 (Ar *C*H), 127.7 (Ar *C*H), 127.6 (Ar *C*H), 127.5 (Ar *C*H), 127.3 (Ar *C*H), 97.9 (C-1), 75.0 (C-3), 74.2 (*C*H_2Ph), 73.4 (*C*H_2Ph), 73,1 (C-5), 70.6 (*C*H_2Ph), 70.4 (C-4), 70.0 (C-7), 66.6 (*C*H_2Ph), 65.1 (C-3), 38.7 (C-9), 31.1 (C-2), 29.4 (C-8).

Selected signals for β-anomer: ^{1}H NMR (400 MHz; $CDCl_3$): *δ* 4.17 (2 H, t, *J* = 6.2 Hz, C-7a), 2.09 (2 H, m, 2 x H-2), 1.87 (2 H, t, *J* = 6.4 Hz, 2 x H-8); ^{13}C NMR (101 MHz; $CDCl_3$): *δ* 100.6 (C-1), 61.8 (C-7), 38.4 (C-9), 31.1 (C-2), 29.7 (C-8).

6-*O*-(2-deoxy-3,4,6-tri-O-benzyl-α-D-galactopyranosyl)-1,2,3,4-di-*O*-isopropylidene-α-D-galactopyranoside 5c

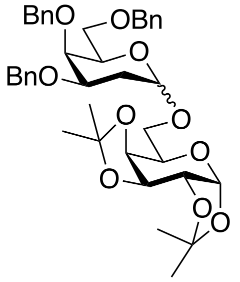

Prepared according to general procedure A from donor **2a** (43 mg, 0.1 mmol), acceptor **4c** (52 mg, 0.2 mmol, 2 eq.) $AuCl_3$ (0.3 mg, 0.001 mmol), and EtOAc (1 mL). After 0.5 hours and following work up and purification via silica gel flash column chromatography (5:1 then 1:1 Hexane/EtOAc) gave *the title compound* **5c** as a colourless oil (91 mg, 84%, α/β = 11:1) plus acceptor **4c** (26 mg, 50% of amount used); R_f= 0.57 (3:1 Hexane/EtOAc); MS (ES^+) found m/z = 699.0 $[M+Na]^+$;

NMR data for α-anomer: ^{1}H NMR (400 MHz; $CDCl_3$): δ 7.34–7.23 (15 H, m, Ar*H*), 5.52 (1 H, d, J = 5.0 Hz, H-1_{Gal}), 5.03 (1 H, d, J = 3.1 Hz, H-1_{2DGal}), 4.92 (1 H, d, J = 11.6 Hz, CH_2Ph), 4.62 (1 H, d, J = 11.5 Hz, CH_2Ph), 4.58 (2 H, s, CH_2Ph, H-3_{Gal}), 4.49 (1 H, d, J = 12 Hz, CH_2Ph), 4.42 (1 H, d, J = 12 Hz, CH_2Ph), 4.31 (1 H, dd, J = 5.0, 2.3 Hz, H-2_{Gal}), 4.21 (1 H, dd, J = 7.9, 1.4 Hz, H-4_{Gal}), 3.98–3.93 (4 H, m, H-3_{2DGal}, H-4_{2DGal}, H-5_{2DGal}, H-2_{Gal}), 3.77–3.72 (1 H, m, H-$6a_{2DGal}$), 3.68–3.66 (1 H, m, H-$6b_{2DGal}$), 3.64–3.60 (1 H, m, H-$6a_{Gal}$), 3.56–3.53 (1 H, m, H-$6a_{Gal}$), 2.20 (1 H, td, J = 12.0, 4.0 Hz, H-$2_{2DGal\ ax}$), 2.02 (1 H, dd, J = 12.8, 4.1 Hz, H-$2_{2DGal\ eq}$), 1.51 (3 H, s, OCH_3), 1.42 (3 H, s, OCH_3), 1.33 (6 H, s, 2 x OCH_3); ^{13}C NMR (101 MHz; $CDCl_3$): δ 139.0 (Ar C), 138.6 (Ar C), 138.2 (Ar C), 128.4 (Ar *C*H), 128.23 (Ar *C*H), 128.19 (Ar *C*H), 127.8 (Ar *C*H), 127.6 (Ar *C*H) 127.5 (Ar *C*H), 127.3 (Ar *C*H), 109.3 ($O_2C(CH_3)_2$), 108.5 ($O_2C(CH_3)_2$), 97.6 (C-1_{2DGal}), 96.4 (C-1_{Gal}), 74.7 (CH), 74.3 (*C*H_2Ph), 73.4 (*C*H_2Ph), 72.9 (CH), 71.1 (C-4), 70.71 (CH), 70.65 (CH), 70.4(*C*H_2Ph), 69.9 (CH), 69.2 (C-6_{Gal}), 65.9 (CH), 65.6 (C-6_{2DGal}), 31.2 (C-2_{2DGal}), 26.1 (*C*H_3), 26.0 (*C*H_3), 25.0 (*C*H_3), 24.6 (*C*H_3).

The α/β ratio was determined based on the integrations of peaks at 5.57 (1 H, d, J = 5.2 Hz, H-1_{Gal} β) and 5.52 (H-1_{Gal} α).

Data in agreement with previously reported synthesis.[15, 16]

1-Methyl-phenyl 2,3,4-tri-*O*-benzyl-6-*O*-(3,4,6-tri-*O*-benzyl-α-D-2-deoxy-galactopyranosyl)-α-D-galactopyranoside 5d

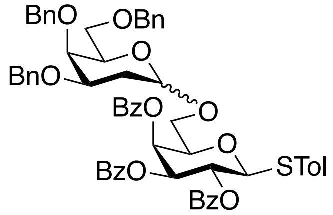

Prepared according to general procedure A from donor **2a** (43 mg, 0.1 mmol), acceptor **4d** (120 mg, 0.2 mmol) $AuCl_3$ (0.9 mg, 0.003 mmol), and toluene (1 mL). After 2 hours and following work up and purification via silica gel flash column chromatography (4:1 then 1:1 Hexane/EtOAc) gave *the title compound* **5d** as a colourless oil (85 mg, 84%, α/β = 11:1) plus acceptor **4d** (59 mg, 49% of amount used); R_f = 0.26 (2:1 Hexane/EtOAc); MS (ES^+) found *m/z* 1032.4 $[M+NH_4]^+$, HRMS (ESI) m/z: Calcd $C_{61}H_{62}O_{12}NS$ $[M+NH_4]^+$ 1032.3987; Found 1032.4012.

NMR data for α-anomer: ^{1}H NMR (400 MHz; $CDCl_3$): δ 8.05–8.00 (2 H, m, Ar*H*), 7.98–7.95 (2 H, m, Ar*H*), 7.92–7.88 (2 H, m, Ar*H*), 7.83–7.79 (2 H, m, Ar*H*), 7.65–7.51 (4 H, m, Ar*H*), 7.48–7.19 (24 H, m, Ar*H*), 6.00 (1 H, dd, *J* = 3.2, 0.8 Hz, H-4_{Gal}), 5.72 (1 H, t, *J* = 9.9 Hz, H-2_{Gal}), 5.61 (1 H, dd, *J* = 10.0, 3.2 Hz, H-3_{Gal}), 4.97 (1 H, d, *J* = 11.7 Hz, CH_2Ar), 4.92–4.89 (2 H, m, H-1_{Gal}, H-1_{Gal2D}), 4.66–4.62 (3 H, m, 3 x CH_2Ar), 4.57 (1 H, d, *J* = 11.9 Hz, CH_2Ar), 4.50 (1 H, d, *J* = 11.8 Hz, CH_2Ar), 4.23 (1 H, t, *J* = 6.9 Hz, H-5_{Gal}), 4.00 (1 H, t, *J* = 6.9 Hz, H-5_{Gal2D}), 3.97–3.91 (3 H, m, H-3_{Gal2D}, H-4_{Gal2D}, H-6_{Gal}), 3.67–3.59 (3 H, m, H-6_{Gal}, 2 x H-6_{Gal2D}), 2.43 (3 H, s, ArCH_3), 2.19 (1 H, td, *J* = 12.4, 3.3 Hz, H-$2_{Gal2D\ ax}$), 2.04 (1 H, dd, *J* = 12.8, 4.5 Hz, H-$2_{Gal2D\ eq}$); ^{13}C NMR (101 MHz; $CDCl_3$): δ 165.6 (C=O), 165.3 (C=O), 165.1 (C=O), 138.9 (Ar C), 138.6 (Ar C), 138.5 (Ar C), 138.2 (Ar C), 134.7 (Ar CH), 133.4 (Ar CH), 133.3 (Ar CH), 133.2 (Ar CH), 130.0 (Ar CH), 129.83 (Ar CH), 129.78 (Ar CH), 129.6 (Ar CH), 129.5 (Ar CH), 129.5 (Ar C), 129.2 (Ar C), 129.0 (Ar C), 128.5 (Ar CH), 128.44 (Ar CH), 128.42 (Ar CH), 128.27 (Ar CH), 128.25 (Ar CH), 128.22 (Ar CH), 127.9 (Ar CH), 127.5 (Ar CH), 127.4 (Ar CH), 99.1 (C-1_{Gal2D}), 85.6 (C-1_{Gal}), 75.8 (C-5_{Gal}), 74.7 (C-3_{Gal2D} or C-4_{Gal2D}), 74.3 (*C*H_2Ar), 73.5 (*C*H_2Ar), 73.3 (C-3_{Gal}), 73.1 (C-3_{Gal2D} or C-4_{Gal2D}), 70.5 (*C*H_2Ar), 70.4 (C-5_{Gal2D}), 69.8 (C-6_{Gal2D}), 68.4 (C-4_{Gal}), 68.1 (C-2_{Gal}), 65.7 (C-6_{Gal}), 30.8 (C-2_{Gal2D}), 21.4 (Ar*C*H_3).

Selected signals for β-anomer: ^{1}H NMR (400 MHz; $CDCl_3$): 5.93 (1 H, dd, *J* = 3.4, 0.6 Hz, H-4_{Gal}), 5.03 (1 H, *J* = 10.0 Hz, H-1_{Gal2D}), 2.41 (3 H, s, ArCH_3); ^{13}C NMR (101 MHz; $CDCl_3$): δ 100.9 (C-1_{Gal2D}), 85.6 (C-1_{Gal}), 30.8 (C-2_{Gal2D}).

3-*O*-(2-deoxy-3,4,6-tri-*O*-benzyl-2-deoxy-α-D-galactopyranoside)-1,2:5,6-di-*O*-isopropy-lidene-α-D-glucofuranoside 5h

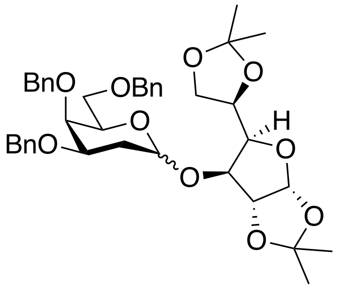

Prepared according to general procedure A from donor **2a** (26 mg, 0.06 mmol), acceptor **4h** (23 mg, 0.09 mmol, 1.5 eq.), $AuCl_3$ (0.2 mg, 0.00067 mmol, 1 mol%) and EtOAc (0.4 mL). After 3 hours, and following work up and purification via silica gel flash column chromatography (7:1 to 5:1 Hexane/EtOAc) gave *the title compound* **5h** as a colourless oil (4 mg, 0.006 mmol, 10%, α/β = 3:1); R_f = 0.56 (2:1 Hexane/EtOAc); MS (ES^+) found m/z = 699.4 $[M+Na]^+$.

NMR data for α-anomer: ^1H NMR (400 MHz; $CDCl_3$): δ 7.36–7.26 (15 H, m, Ar*H*), 5.83 (1 H, d, *J* = 3.6 Hz, H-1_{Glc}), 5.25 (1 H, d, *J* = 3.1 Hz, H-1_{2DGal}), 4.94 (1 H, d, *J* = 11.6 Hz, CH_2Ph), 4.64–4.49 (4 H, m, 4 x CH_2Ph), 4.44 (1 H, d, *J* = 11.8 Hz, CH_2Ph), 4.23 (1 H, d, *J* = 2.8 Hz, H-3_{Glc}), 4.20–4.15 (1 H, m, H-5_{2DGal}), 4.11–4.07 (2 H, m, H-4_{Glc}, H-6_{2DGal}), 4.01–3.85 (4 H, m, H-3_{2DGal}, H-4_{2DGal} H-5_{Glc}, H-6_{2DGal}), 3.63 (1 H, dd, *J* = 9.5, 6.2 Hz, H-6_{Glc}), 3.57 (1 H, dd, *J* = 9.5, 6.2 Hz, H-6_{Glc}), 2.24 (1 H, td, *J* = 12.4, 3.7 Hz, H-$2_{2DGal\ ax}$), 2.00 (1 H, dd, *J* = 12.7, 4.5 Hz, H-$2_{2DGal\ eq}$), 1.48 (3 H, s, CH_3), 1.40 (3 H, s, CH_3), 1.33 (3 H, s, CH_3), 1.21 (3 H, s, CH_3); ^{13}C NMR (101 MHz; $CDCl_3$): δ 138.7 (Ar C), 138.3 (Ar C), 138.0 (Ar C), 128.44 (Ar *C*H), 128.40 (Ar *C*H), 128.24 (Ar *C*H), 128.10 (Ar *C*H), 127.8 (Ar *C*H), 127.63 (Ar *C*H), 127.60 (Ar *C*H), 127.57 (Ar *C*H), 127.3 (Ar *C*H), 111.8 (*C*($CH_3)_2$), 109.1 (*C*($CH_3)_2$), 105.3 (C-1_{Glc}), 99.5 (C-1_{2DGal}), 83.5 (C-2_{Glc}), 81.3 (C-4_{Glc}), 80.9 (C-3_{Glc}), 74.4 (C-3_{2DGal}), 74.3 (*C*H_2Ph), 73.6 (*C*H_2Ph), 73.1 (C-4_{2DGal} or C-5_{Glc}), 72.6 (C-5_{2DGal}), 71.0 (C-4_{2DGal} or C-5_{Glc}), 70.5 (*C*H_2Ph), 70.0 (C-6_{Glc}), 67.6 (C-6_{2DGal}), 31.0 (C-2_{2DGal}), 26.9 (*C*H_3), 26.8 (*C*H_3), 26.1 (*C*H_3), 25.4 (*C*H_3).

The α/β ratio was determined based on the integrations of peaks at 5.99 (1 H, d, *J* = 3.7 Hz, H-1_{Glc} β) and 5.83 (H-1_{Glc} α).

Data in agreement with previously reported synthesis.[17]

Methyl 3,4,6-tri-*O*-benzyl-2-*O*-(3,4,6-tri-*O*-benzyl-α-D-2-deoxy-galactopyranosyl)-α-D-glucopyranoside 5i

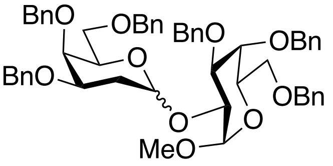

Prepared according to general procedure A from donor **2a** (43 mg, 0.1 mmol), acceptor **4i** (93 mg, 0.2 mmol, 2 eq.), $AuCl_3$ (0.3 mg, 0.001 mmol) and toluene (1 mL). After 4 hours, and following work up and purification via silica gel flash column chromatography (Note: only 75% of the crude material by weight was columned) (7:3 Hexane/EtOAc) gave *the title compound* **5i** as a colourless oil (22 mg, 0.025 mmol, 25%, α/β = 7:1) plus recovered acceptor **4i** (58 mg, 62% of amount used); MS (ES^+) found m/z = 903.4 [M+Na].

NMR data for α-anomer: ^{1}H NMR (400 MHz; $CDCl_3$): δ 7.37–7.11 (39 H, m, Ar*H*), 5.19 (1 H, d, J = 2.9 Hz, H-1_{2DGal}), 4.97 (1 H, d, J = 1.8 Hz, H-1_{Glc}), 4.92 (1 H, d, J = 11.6 Hz, C*H*$_2$Ph), 4.81–4.78 (3 H, m, 3 x C*H*$_2$Ph), 4.67–4.38 (9 H, m , 9 x C*H*$_2$Ph), 4.13 (1 H, t, J = 6.6 Hz, H-5_{Glc}), 3.43–3.85 (3 H, m, H-2_{Glc}, H-3_{2DGal}, H-4_{2DGal}), 3.79–3.66 (4 H, m, H-4_{Glc}, H-5_{2DGal}, 2 x H-6_{2DGal}), 3.65–3.58 (2 H, m, H-3_{Glc}, H-6_{Glc}), 3.55 (1 H, dd ,J = 9.4, 7.1 Hz, H-6_{Glc}), 3.44 (3 H, s, C*H*$_3$), 2.28 (1 H, td, J = 12.4, 3.5 Hz, H-$2_{2DGal\ ax}$), 2.10 (1 H, dd, J = 12.6, 4.4 Hz, H-$2_{2DGal\ eq}$); ^{13}C NMR (101 MHz; $CDCl_3$): δ 138.9 (Ar C), 138.8 (Ar C), 138.44 (Ar C), 138.38 (Ar C), 138.2 (Ar C), 138.0 (Ar C), 128.4 (Ar CH), 128.34 (Ar CH), 128.29 (Ar CH), 128.2 (Ar CH), 128.0 (Ar CH), 127.9 (Ar CH), 127.70 (Ar CH), 127.66 (Ar CH), 127.61 (Ar CH), 127.56 (Ar CH), 127.45 (Ar CH), 127.38 (Ar CH), 127.33 (Ar CH), 96.4 (C-1_{Glc}), 94.0 (C-1_{2DGal}), 81.2 (CH), 77.9 (C-3_{Glc}), 75.6 (*C*H$_2$Ph), 75.0 (*C*H$_2$Ph), 74.4 (*C*H$_2$Ph), 74.3 (2 x CH), 73.0 (*C*H$_2$Ph), 72.9 (CH), 70.3 (*C*H$_2$Ph), 70.2 (CH), 69.6 (C-5_{Glc}), 69.1 (C-6), 68.6 (C-6), 55.0 (O*C*H$_3$), 30.9 (C-2_{2DGal});

Selected peaks for β-anomer: ^{1}H NMR (400 MHz; $CDCl_3$): 2.25–2.18 (1 H, m, H-$2_{2DGal\ ax}$), 1.95 (1 H, dd, J = 13.0, 4.7 Hz, H-$2_{2DGal\ eq}$). The α/β ratio was determined from integration of the H-2 peaks.

Data in agreement with previously reported synthesis.[18]

3-(*N*-benzoyloxycarbonyl)aminopropyl-3,4,6-tri-*O*-benzyl-2-deoxy-D-glucoopyranoside 7b

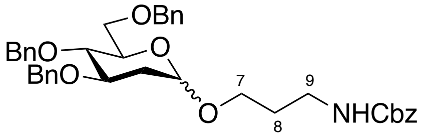

Prepared according to general procedure A from donor **3a** (43 mg, 0.1 mmol), acceptor **4b** (42 mg, 0.2 mmol) $AuCl_3$ (0.75 mg, 0.0025 mmol), EtOAc (1 mL). After 1.5 h, and following work up and purification via silica gel flash column chromatography (2:1 Hexane/EtOAc) gave *the title compound* **7b** as colourless oil (50 mg, 0.08 mmol, 80%, α/β = 5:1). R_f = 0.62 (3:2 Hexane/EtOAc); MS (ES^+) found m/z = 648.3 $[M+Na]^+$, HRMS (ESI) m/z: Calcd $C_{38}H_{43}NO_7Na$ $[M+Na]^+$ 648.2923; Found 648.2919.

NMR data for α-anomer: ^1H NMR (400 MHz, $CDCl_3$) δ 7.36–7.17 (m, 20H, Ar*H*), 5.30 1 H, br s, N*H*), 5.16–5.14 (2 H, m, 2 x C*H*$_2$Ph), 4.94 (1 H, d, *J* = 2.7 Hz, H-1), 4.89 (1 H, d, *J* = 10.9 Hz, C*H*$_2$Ph), 4.71–4.51 (5 H, m, 5 x C*H*$_2$Ph), 3.97 (1 H, ddd, *J* = 11.4, 8.7, 5.0 Hz, H-3), 3.80–3.64 (4 H, m, H-5, 2 x H-6, H-7b), 3.54 (1 H, dd, *J* = 9.3, 9.1 Hz, H-4), 3.47–3.23 (3 H, m, H-7a, 2 x H-9), 2.28 (1 H, dd, *J* = 13.11, 4.9 Hz, $H\text{-}2_{eq}$), 1.83–1.70 (3 H, 2 x H-8, $H\text{-}2_{ax}$); ^{13}C NMR (101 MHz, $CDCl_3$) δ 156.4 (*C*=O), 138.96, 138.6 (Ar C), 138.4 (Ar C), 138.0 (Ar C), 136.6 (Ar C), 128.5 (Ar CH), 128.37 (Ar CH), 128.35 (Ar CH), 128.3 (Ar CH), 128.1 (Ar CH), 128.01 (Ar CH), 127.97 (Ar CH), 127.9 (Ar CH), 127.7 (Ar CH), 127.6 (Ar CH), 127.5 (Ar CH), 97.4 (C-1), 78.3 (C-4), 77.7 (C-3), 75.0 (*C*H$_2$Ph), 73.4 (*C*H$_2$Ph), 71.8 (*C*H$_2$Ph), 71.1 (C-5), 68.9 (C-6), 65.6 (*C*H$_2$Ph), 65.1 (C-7), 38.7 (C-9), 35.4 (C-2), 29.4 (C-8).

Selected peaks for β-anomer: ^1H NMR (400 MHz, $CDCl_3$) δ 2.37–2.32 (1 H, m, $H\text{-}2_{2DGal\ ax}$), 1.64 (1 H, dd, *J* = 12.2, 2.1 Hz, $H\text{-}2_{2DGal\ eq}$); ^{13}C NMR (101 MHz, $CDCl_3$) δ 99.8 (C-1), 79.3 (CH), 78.1 (CH), 77.2 (CH), 71.4 (*C*H$_2$Ph), 29.4 (C-8).

6-*O*-(3,4,6-tri-*O*-benzyl-2-deoxy-α-D-glucopyranosyl)-1,2,3,4-di-*O*-isopropylidene-α-D-galactopyranoside 7c

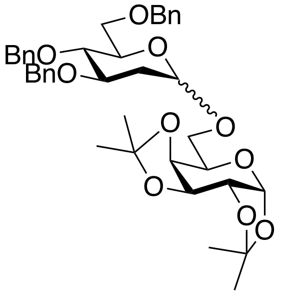

Prepared according to general procedure A from donor **3a** (43 mg, 0.1 mmol), acceptor **4c** (52 mg, 0.2 mmol, 2 eq.), $AuCl_3$ (0.75 mg, 0.0025 mmol), toluene (1 mL). After 1.5 hours, and following work up and purification via silica gel flash column chromatography (5:1 Hexane/EtOAc) gave *the title compound* **6c** as a colourless oil (50 mg, 0.074 mmol, 74%, α/β = 6:1) plus recovered acceptor **7c** (21 mg, 40% of amount used); MS (ES^+) found *m/z* 699.3 [M+Na].

NMR data for α-anomer: ^{1}H NMR (400 MHz, Chloroform-*d*) δ 7.39–7.20 (15 H, m, Ar*H*), 5.54 (1 H, d, *J* = 5.1 Hz, H-1_{Glc}), 5.05 (1 H, d, *J* = 2.6 Hz, H-1_{2DGlc}), 4.91 (1 H, *J* = 10.8 Hz, CH_2Ph), 4.72–4.61 (4 H, m, H-3_{Glc}, 3 x CH_2Ph), 4.59–4.52 (2 H, m, 2 x CH_2Ph), 4.34 (1 H, dd, *J* = 5.0, 2.4 Hz, H-2_{Glc}), 4.25 (1 H, dd, *J* = 7.9, 1.9 Hz, H-4_{Glc}), 4.06–3.96 (2 H, m,),4.03 (1 H, ddd, *J* = 9.1, 6.7, 4.7 Hz, H-3_{2DGlc}), 3.99–3.96 (1 H, m, H-5_{Glc}), 3.85–3.75 (3 H, m, H-5_{2DGlc}, H-$6a_{2DGlc}$, H-$6a_{Glc}$), 3.72–3.66 (3 H, m, H-4_{2DGlc}, H-$6b_{2DGlc}$, H-$6b_{Glc}$), 2.36 (1 H, ddd, *J* = 12.6, 5.1, 1.2 Hz, H-$2_{2DGlc\ eq}$), 1.76 (1 H, ddd, *J* = 13.0, 11.5, 3.7 Hz, H-$2_{2DGlc\ ax}$), 1.55 (3 H, m, CH_3), 1.47 (3 H, m, CH_3), 1.37 (3 H, m, CH_3), 1.36 (3 H, m, CH_3); ^{13}C NMR (101 MHz, Chloroform-*d*) δ 138.8 (Ar C), 138.6 (Ar C), 138.3 (Ar C), 138.2 (Ar C), 128.4 (Ar CH), 128.33 (Ar CH), 128.31 (Ar CH), 127.95 (Ar CH), 127.91 (Ar CH), 127.60 (Ar CH), 127.59 (Ar CH), 127.56 (Ar CH), 127.49 (Ar CH), 109.3 (*C*(CH_3)$_2$), 108.5 (*C*(CH_3)$_2$), 97.3 (C-1_{2DGlc}), 96.3 (C-1_{Glc}), 78.2 (C-4_{2DGlc}), 77.6 (C-3_{2DGlc}), 75.0 (*C*H_2Ph), 73.4 (*C*H_2Ph), 71.8 (*C*H_2Ph), 71.0 (C-4_{Glc} and C-5_{2DGlc}), 70.7 (C-2_{Glc} and C-3_{Glc}), 68.8 (C-6_{Glc}), 65.7 (C-5), 65.4 (C-6_{2DGlc}), 35.4 (C-2_{2DGlc}), 26.2 (*C*(CH_3)), 26.0 (*C*(CH_3)), 24,9 (*C*(CH_3)), 24,6 (*C*(CH_3)).

Selected peaks for β-anomer: ^{1}H NMR (400 MHz, Chloroform-*d*) δ 5.58 (1 H, d, *J* = 5.0 Hz, H-1_{Glc}), 44.59–4.52 (1H, m, H-1_{2DGlc}), 10.9, 3.4 Hz, H-6_{2DGlc}), 3.56 (1 H, t, *J* = 9.1 Hz, H-4_{Glc}), 3.43 (1 H, ddd, *J* = 9.7, 4.2, 2.5 Hz, H-5_{Glc}), 2.49 (1 H, ddd, *J* = 12.6, 4.9, 1.9 Hz, H-$2_{2DGlc\ eq}$), 1.70–1.65 (4 H, m, H-$2_{2DGlc\ ax}$, CH_3), 1.57 (3 H, s, CH_3), 1.46 (3 H, s, CH_3), 1.35 (3 H, m, CH_3); ^{13}C NMR (101 MHz, Chloroform-*d*) δ 100.4 (C-1_{2DGlc}), 96.4 (C-1_{Glc}).

Data in agreement with previously reported synthesis. [19, 20]

1-*O*-Allyl-3,4,6-tri-*O*-benzyl-2-deoxy-D-glucopyranoside 7e

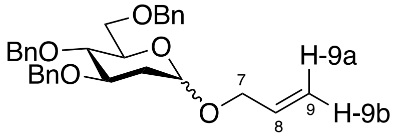

Prepared according to general procedure A from donor **3a** (43 mg, 0.1 mmol), acceptor **4e** (14 µL, 0.2 mmol), $AuCl_3$ (0.75 mg, 00.0025 mmol), EtOAc (0.5 mL). After 2 hours and following work up and purification via silica gel flash column chromatography (9:1 to 2:3 Hexane/EtOAc) gave *the title compound* **7e** as a colourless oil (33 mg, 0.07 mmol, 70%, α/β = 3.3:1); R_f = 0.94 (1:1 Hexane/EtOAc); MS (ES^+) found *m/z* 497.5 $[M+Na]^+$.

NMR data for α-anomer: ^{1}H NMR (400 MHz, Chloroform-*d*) δ 7.41–7.21 (15 H, m, Ar*H*), 5.93 (1 H, dddd, 17.2, 10.4, 6.1, 5.2 Hz, H-8), 5.30 (1 H, dq, *J* = 17.2, 1.7, H-9a), 5.20 (1 H, dq, *J* = 10.4, 1.5 Hz, H-9b), 5.05 (1 H, dd, *J* = 3.4, 1.1 Hz, H-1), 4.93 (1 H, d, *J* = 10.9 Hz, CH_2Ph), 4.74–4.50 (5 H, m, 5 x CH_2Ph), 4.17 (1 H, ddt, *J* = 13.0, 5.1, 1.5 Hz, H-7a), 4.06 (1 H, ddd, *J* = 11.5, 8.7, 5.1 Hz, H-3), 3.98 (1 H, ddt, *J* = 13.0, 6.1, 1.4 Hz, 1H, H-7b), 3.84–3.64 (4 H, m, H-4, H-5, 2 x H-6), 2.35 (ddd, *J* = 13.0, 5.1, 1.3 Hz, 1H, H-2_{eq}), 1.78 (1 H, m, *J* = 13.0, 11.5, 3.6 Hz, H-2_{ax}); ^{13}C NMR (101 MHz, $CDCl_3$) δ 138.8 (Ar C), 138.6 Ar C), 138.2 (Ar C), 134.2 (C-8), 128.4 (Ar CH), 128.34 (Ar CH), 128.33 (Ar CH), 127.93 (Ar CH), 127.87 (Ar CH), 127.62 (Ar CH), 127.58 (Ar CH), 127.5 (Ar CH), 117.1 (C-9), 96.80 (C-1), 78.3 (C-4), 77.7 (C-3), 75.0 (CH_2Ph), 73.5 (CH_2Ph), 71.8 (CH_2Ph), 70.9 (C-5), 68.9 (C-6), 67.7 (C-7), 35.5 (C-2).

NMR data for β-anomer: ^{1}H NMR (400 MHz, Chloroform-*d*) δ 7.41–7.21 (15 H, m, Ar*H*), 6.02–5.96 (1H, m, H-8), 5.33 (1 H, dq, *J* = 17.2, 1.6 Hz, H-9a), 5.23 (1 H, dq, *J* =11.0, 1.5 Hz, H-9b), 4.94 (1 H, d, *J* = 10.9 Hz, CH_2Ph), 4.74–4.50 (5 H, m, 5 x CH_2Ph), 4.55–4.51 (1 H, m, H-1), 4.42 (1 H,ddt, *J* = 12.8, 5.1, 1.5 Hz, H-7a), 4.12–4.03 (1 H, m, H-7b), 3.84–3.64 (3 H, m, H-3, 2 x H-6), 3.56 (1 H, t, *J* = 9.1 Hz, H-4), 3.46 (1 H, ddd, *J* = 9.6, 4.8, 2.1 Hz, H-5), 2.42–2.37 (1 H, m, H-2_{eq}), 1.73 (1 H, ddd, *J* = 12.5, 11.8, 9.8 Hz, H-2_{ax}); ^{13}C NMR (101 MHz, $CDCl_3$) δ 138.4 (Ar C), 138.3 (Ar C), 134.2 (C-8α), 128.4 (Ar CH), 128.0 (Ar CH), 127.8 (Ar CH), 127.71 (Ar CH), 127.68 (Ar CH), 127.67 (Ar CH), 117.4 (C-9), 98.9 (C-1), 79.5 (C-3), 78.2 (C-4), 75.2 (C-5β), 75.0 (CH_2Ph), 73.5 (CH_2Ph), 71.4 (CH_2Ph), 69.7 (C-7), 69.4 (C-6), 36.7 (C-2).

Data in agreement with previously reported synthesis.[19, 20]

1,2,3,6-tetra-*O*-benzyl-D-α/β-D-glucopyranoside 7f

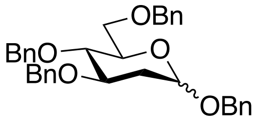

Prepared according to general procedure A from donor **3a** (50 mg, 0.12 mmol), benzyl alcohol **4f** (25 µL, 0.24 mmol), $AuCl_3$ (0.9 mg, 2.5 mol%) and EtOAc (0.6 mL). After heating for 1 h, and following work up and column chromatography (9:1 to 7:3 Hexane/EtOAc) gave *the title compound* **7f** as a colourless oil (40 mg, 0.08 mmol, 63%, α/β = 4.5:1*). R_f =
0.86 (3:2 Hexane/EtOAc); MS (ES^+) found *m/z* 547.6 $[M+Na]^+$.

NMR data for α-anomer: ^{1}H NMR (400 MHz, Chloroform-*d*) δ 7.40–7.31 (18 H, m, Ar*H*), 7.23 (2 H, dd, *J* = 7.6, 1.9 Hz, Ar*H*), 5.11 (1 H, d, *J* = 2.5 Hz, H-1), 4.95 (1 H, d, *J* = 10.8 Hz, CH_2Ph), 4.74–4.69 (4 H, m, 4 x CH_2Ph), 4.58 (2 H, d, *J* = 11.7 Hz, CH_2Ph), 4.50 (1 H, d, *J* = 11.9 Hz, CH_2Ph), 4.10 (1 H, ddd, *J* = 11.5, 8.8, 5.0 Hz, H-3), 3.89 (1 H, ddd, *J* = 9.7, 3.9, 1.8 Hz, H-5), 3.84 (1 H, dd, *J* = 10.4, 4.0 Hz, H-6), 3.72 (1 H, *J* = 10.4, 1.9 Hz, H-6), 3.69 (1 H, t, *J* = 9.3 Hz, H-4), 2.38 (1 H, ddd, *J* = 13.0, 5.1, 1.2 Hz, H-2_{eq}), 1.81 (1 H, ddd, *J* = 13.0, 11.5, 3.7 Hz, H-2_{eq}); ^{13}C NMR (101 MHz, Chloroform-*d*) δ 138.7 (Ar C), 138.5 (Ar C), 138.2 (Ar C), 137.7 (Ar C), 128.39 (Ar CH), 128.37 (Ar CH), 128.35 (Ar CH), 128.0 (Ar CH), 127.94 (Ar CH), 127.89 (Ar CH), 127.7 (Ar CH), 127.6 (Ar CH), 127.5 (Ar CH), 96.8 (C-1), 78.4 (C-4), 77.8 (C-3), 75.0 (*C*H_2Ph), 73.5 (*C*H_2Ph), 71.9 (*C*H_2Ph), 71.0 (C-5), 68.94 (C-6), 68.89 (*C*H_2Ph), 35.5 (C-2).

*Plus 3% 2,3-unsaturated ferrier product.

Data in agreement with previous synthesis.[22, 21]

Methyl 2,3,4-tri-*O*-benzyl-6-*O*-(3,4,6-tri-*O*-benzyl-2-deoxy-α-D-gluco-hexopyranosyl)-α-D-glucopyranoside 7g

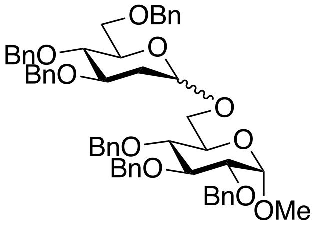

Prepared according to general procedure A from donor **3a** (43 mg, 0.1 mmol), acceptor **4g** (93 mg, 0.2 mmol), $AuCl_3$ (0.75 mg, 0.0025 mmol) and toluene (1 mL). After 2 hours and following work up and purification via silica gel flash column chromatography (3:1 Hexane/EtOAc) gave *the title compound* **7g** as colourless oil (53 mg, 0.06 mmol, 60%, α/β = >15:1) plus recovered acceptor **4g** (53 mg, 57% of amount used); R_f = 0.56 (3:2 Hexane/EtOAc); MS (ES^+) found *m/z* 898.5 $[M+NH_4]^+$.

NMR data for α-anomer: ^{1}H NMR (400 MHz, Chloroform-*d*) δ 7.44–7.20 (30 H, m, Ar*H*), 5.06 (1 H, br s, H-1_{2DGlc}), 5.05 (1 H, d, *J* = 10.9 Hz, C*H*$_2$Ph), 4.98 (1 H, d, *J* = 11.2 Hz, C*H*$_2$Ph), 4.94 (1 H, dd, *J* = 11.0 Hz, C*H*$_2$Ph), 4.88–4.84 (2 H, m, 2 x C*H*$_2$Ph), 4.76–4.60 (7 H, m, H-1_{Glc}, 6 x C*H*$_2$Ph), 4.55 (1 H, dd, *J* = 11.0 Hz, C*H*$_2$Ph), 4.47 (1 H, dd, *J* = 12.1 Hz, C*H*$_2$Ph), 4.06 (1 H, t, *J* = 9.3 Hz, H-3_{Glc}), 4.02–3.97 (1 H, m, H-3_{2DGlc}), 3.88 (1 H, dd, *J* = 11.3, 4.4 Hz, H-$6a_{2DGlc}$), 3.80 (1 H, ddd, *J* = 10.0, 4.3, 1.5 Hz, H-5_{2DGlc}), 3.74 (1 H, ddd, *J* = 9.9, 3.3, 1.8 Hz, H-5_{Glc}), 3.69–3.54 (3 H, m, H-4_{Glc}, H-$6b_{2DGlc}$, H-$6a_{Glc}$), 3.60–3.54 (3 H, m, H-2_{Glc}, H-4_{2DGlc}, H-$6b_{Glc}$), 3.41 (3 H, s, C*H*$_3$), 2.36 (1 H, ddd, *J* = 13.0, 5.0, 1.1 Hz, 1H, H-$2_{2DGlc\ eq}$), 1.675 (1 H, ddd, *J* = 12.9, 11.6 Hz, 3.5 Hz, H-$2_{2DGlc\ ax}$); ^{13}C NMR (101 MHz, Chloroform-*d*) δ 128.48 (Ar CH), 128.42 (Ar CH), 128.37 (Ar CH), 128.34 (Ar CH), 128.30 (Ar CH), 128.2 (Ar CH), 128.1 (Ar CH), 128.0 (Ar CH), 127.92 (Ar CH), 127.87 (Ar CH), 127.78 (Ar CH), 127.74 (Ar CH), 127.65 (Ar CH), 127.57 (Ar CH), 127.54 (Ar CH), 127.4 (Ar CH), 97.9 (C-1_{Glc}), 97.8 (C-1_{2DGlc}), 82.2 (C-3_{Glc}), 80.0 (C-4_{2DGlc}), 78.1 (C-4_{Glc}), 77.8 (C-2_{Glc}), 77.2 (C-3_{2DGlc}), 75.8 (*C*H$_2$Ph), 74.9 (*C*H$_2$Ph), 74.8 (*C*H$_2$Ph), 73.4 (*C*H$_2$Ph), 73.3 (*C*H$_2$Ph), 71.7 (*C*H$_2$Ph), 70.9 (C-5_{Glc}), 69.8 (C-5_{2DGlc}), 68.7 (C-6_{Glc}), 65.7 (C-6_{2DGlc}), 55.1 (*OCH*$_3$), 35.3 (C-2_{2DGlc}).

Data in agreement with previously reported synthesis.[22]

3-*O*-(2-deoxy-3,4,6-tri-*O*-benzyl-α-D-2-deoxy-glucopyranoside)-1,2:5,6-di-*O*-isopropy-lidene-α/β-D-glucofuranoside 7h

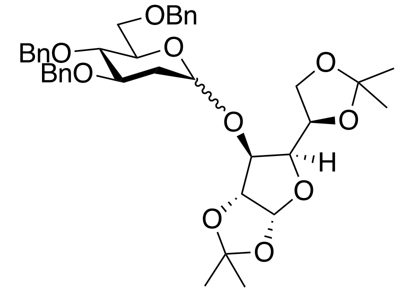

Prepared according to general procedure A from donor **3a** (43 mg, 0.1 mmol), acceptor **4h** (52 mg, 0.2 mmol, 2 eq.) $AuCl_3$ (0.75 mg, 0.0025 mmol, 2.5 mol%), EtOAc (0.5 mL). After heating for 2 hours, and following work up and column chromatography (9:1 to 1:1 Hexane/EtOAc) gave title compound **7h** as colourless oil (13 mg, 0.192 mmol, 19%, α/β = 4:1); R_f = 0.48 (1:1 Hexane/EtOAc); MS (ES$^+$) found *m/z* 699.3 [M + Na]$^+$.

NMR data for α-anomer: ^{1}H NMR (400 MHz, Chloroform-*d*) δ 7.37–7.19 (15 H, m, Ar*H*), 5.84 (1 H, d, *J* = 3.7, H-1_{Glc}), 5.26 (1 H, d, *J* = 3.6, H-1_{2DGlc}), 4.92 (1 H, d, *J* = 10.7 Hz, CH_2Ph), 4.71–4.64 (4 H, H-2_{Glc}, 3 x CH_2Ph), 4.60–4.52 (2 H, m, *2 x* CH_2Ph), 4.29 (1 H, d, *J* = 2.8 Hz, H-3_{Glc}), 4.21–4.08 (3 H, m, H-4_{Glc}, H-5_{Glc}, H-$6a_{2DGlc}$), 4.01–3.93 (2 H, m, H-$6b_{2DGlc}$, H-3_{2DGlc}), 3.84–3.50 (3 H, m, H-5_{2DGlc}, 2 x H-6_{Glc}), 3.62 (1 H, t, *J* = 9.2 Hz, H-4_{2DGlc}), 2.30 (1 H, ddd, *J* = 13.1, 5.1, 1.3 Hz, H-$2_{2DGlc\ eq}$), 1.75 (1 H, dd, *J* = 13.0, 11.5, 3.6 Hz, H-$2_{2DGlc\ ax}$), 1.50 (3 H, s, CH_3), 1.43 (3 H, s, CH_3), 1.25 (3 H, s, CH_3), 1.26 (3 H, s, CH_3); ^{13}C NMR (101 MHz, $CDCl_3$) δ 138.5 (Ar C), 138.3 (Ar C), 138.0 (Ar C), 128.40 (Ar CH), 128.37 (Ar CH), 128.0 (Ar CH), 127.9 (Ar CH), 128.74 (Ar CH), 112.00 (C(CH$_3$)$_2$), 109.3 (C(CH$_3$)$_2$), 105.3 (C-1_{Glc}), 98.7 (C-1_{2DGlc}), 83.7 (C-2_{Glc}), 81.3 (C-5_{Glc}), 80.3 (C-3_{Glc}), 78.1 (C-4_{2DGlc}), 77.2 (C-3_{2DGlc}), 75.1 (*C*H$_2$Ph), 73.5 (*C*H$_2$Ph), 72.5 (C-4_{Glc}), 71.9 (*C*H$_2$Ph), 71.7 (C-5_{2DGlc}), 69.0 (C-6_{Glc}), 67.7 (C-6_{2DGlc}), 35.2 (C-2_{2DGlc}), 26.8 (2 x *C*H$_3$), 26.1 (*C*H$_3$), 25.4 (*C*H$_3$).

The α/β ratio was determined based on the integrations of peaks at 6.000 (1 H, d, *J* = 3.7 Hz, H-1_{Glc} β) and 5.84 (H-1_{Glc} α).

Data in agreement with previously reported synthesis.[23]

Synthesis of deoxy-trehalose dimers 10, 11 and 12

2,3,4-Tri-*O*-benzyl-α/β-L-fucopyranose 9

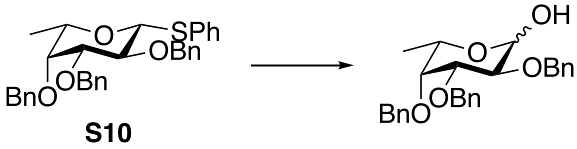

To a solution of **S10**[24] (750 mg, 1.42 mmol) in Acetone/H_2O (9 mL, 9:1 *v:v*) was added *N*-bromosuccinimide (910 mg, 5.11 mmol). The reaction was stirred at room temperature for 2 hours at which point solid $NaHCO_3$ (~1 g) was added and the reaction mixture was concentrated and the reaction mixture was concentrated *in vacuo*. Purification of the crude product by silica gel flash column chromatography (4:1 Hexane/EtOAc) gave the *title product* **9** (548 mg, 1.26 mmol, 89%, α/β = 2:1) as a white solid. Data was in agreement with previously reported synthesis.[25]

3,4,6-Tri-*O*-benzyl-2-deoxy-α-D-galactopyranosyl 3',4',6'-tri-*O*-benzyl-2-deoxy-α- D-galactopyranoside 10

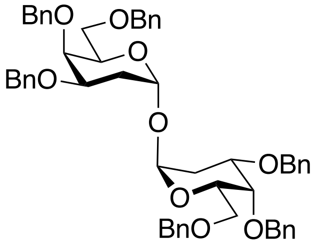

Prepared according to general procedure A from hemiacetal **2a** (43 mg, 0.10 mmol), $AuCl_3$ (0.3 mg, 0.001 mmol) and toluene (0.5 mL). After 3 hours and following work up and purification via silica gel flash column chromatography (8:1 Hexane/EtOAc) gave the *title compound* **10** as a colourless oil (23 mg, 0.027 mmol, 55%); R_f = 0.44 (2:1 Hexane/EtOAc); MS (ES^+) found *m/z* 873.4 $[M+Na]^+$; ^1H NMR (400 MHz, Chloroform-*d*) δ 7.38–7.29 (15 H, m, Ar*H*), 5.28 (1 H, d, *J* = 3.0 Hz, H-1), 4.97 (1 H, d, *J* = 11.6 Hz, C*H*$_2$Ph), 4.67–4.58 (3 H, m, 3 x C*H*$_2$Ph), 4.52 (1 H, d, *J* = 11.7 Hz, C*H*$_2$Ph), 4.45 (1 H, d, *J* = 11.7 Hz, C*H*$_2$Ph), 3.97 (1 H, br s, H-4), 3.91–3.85 (2 H, m, H-3, H-5), 3.66 (1 H, dd, *J* = 9.3, 7.3 Hz, H-6a), 3.57 (1 H, *J* = 9.2, 5.7 Hz, H-6b), 2.28 (1 H, td, *J* = 12.3, 3.6 Hz, H-2_{ax}), 1.89 (1 H, td, *J* = 12.6, 4.5 Hz, H-2_{eq}); ^{13}C NMR (101 MHz, Chloroform-*d*) δ 138.9 (Ar C), 138.5 (Ar C), 138.0 (Ar C), 128.4 (Ar CH), 128.2 (Ar CH), 128.1 (Ar CH), 127.8 (Ar CH), 127.7 (Ar CH), 127.6 (Ar CH), 127.5 (Ar CH), 127.4 (Ar CH), 93.4 (C-1), 74.4 (C-3 or C-5), 74.3 (*C*H$_2$Ph), 73.6 (*C*H$_2$Ph), 73.0 (C-4), 70.5 (C-3 or C-5), 70.3 (*C*H$_2$Ph), 69.3 (C-6), 30.9 (C-2).

Data in agreement with previously reported synthesis.[26]

3,4,6-Tri-*O*-benzyl-2-deoxy-α-D-glucopyranosyl 3',4',6'-tri-*O*-benzyl-2-deoxy-α-D-glucopyranoside 11

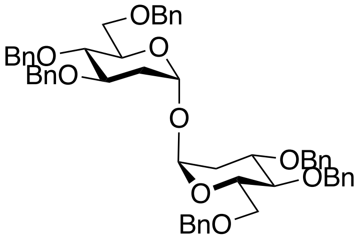

Prepared according to general procedure A from hemiacetal **3a** (215 mg, 0.5 mmol), $AuCl_3$ (1.5 mg, 0.005 mmol) and toluene (2.5 mL). After 4 hours and following work up and purification via silica gel flash column chromatography (8:1 then 4:1 Hexane/EtOAc) gave the *title compound* **11** as a colourless oil (23 mg, 0.027 mmol, 55%); R_f = 0.46 (2:1 Hexane/EtOAc); MS (ES^+) found *m/z* 868.4 $[M+NH_4]^+$; ^{1}H NMR (400 MHz, Chloroform-*d*) δ 7.38–7.28 (30 H, m, Ar*H*), 5.29 (2 H, d, *J* = 2.4 Hz, H-1), 4.95 (2 H, d, *J* = 10.7 Hz, CH_2Ph), 4.71–4.64 (6 H, m, 6 x CH_2Ph), 4.60–4.55 (4 H, m, 4 x CH_2Ph), 3.99 (2 H, ddd, *J* = 11.4, 8.7, 5.0 Hz, H-3), 3.83–3.76 (4 H, m, H-5, H-6a), 3.71–3.65 (4 H, m, H-4, H-6b), 2.19 (2 H, ddd, *J* = 13.0, 5.0, 1.2 Hz, H-2_{eq}), 1.77 (2 H, ddd, *J* = 13.0, 11.5, 3.6 Hz, H-2_{ax}); ^{13}C NMR (101 MHz, Chloroform-*d*) δ 138.6 (Ar C), 138.4 (Ar C), 138.2 (Ar C), 128.44 (Ar CH), 128.38 (Ar CH), 128.1 (Ar CH),127.8 (Ar CH), 127.7 (Ar CH), 127.64 (Ar CH), 127.63 (Ar CH), 92.8 (C-1), 78.1 (C-4), 77.3 (C-4), 75.2 (CH_2Ph), 73.5 (CH_2Ph), 71.8 (CH_2Ph), 71.5 (C-5), 68.8 (C-6), 35.1 (C-2).

Data in agreement with previously reported synthesis.[27]

2,3,4-Tri-*O*-benzyl-α-L-fucopyranosyl 2,3,4-tri-*O*-benzyl-α-L -fucopyranoside 12

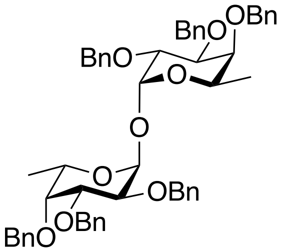

Prepared according to general procedure A from donor **9** (152 mg, 0.35 mmol), toluene (3 mL) and $AuCl_3$ (1.5 mg, 0.005 mmol). After 4 h, and following work-up and purification by silica gel flash column chromatography (6:1 Hexane/EtOAc) to give the *title compound* **12** as a clear oil (90 mg, 0.106 mmol, 60%); R_f = 0.69 (2:1 Hexane/EtOAc); MS (ES$^+$) found *m/z* 850.4 [M+Na]$^+$, HRMS (ESI) m/z: Calcd $C_{54}H_{58}O_9Na$ [M+Na]$^+$ 873.3973; Found 873.3941; ^{1}H NMR (400 MHz, Chloroform-*d*) δ 7.48–7.31 (30 H, m, Ar*H*), 5.29 (2 H, d, *J* = 3.5 Hz, H-1), 5.02 (2 H, d, *J* = 11.6 Hz, C*H*$_2$Ph), 4.89 (2 H, d, *J* = 11.8 Hz, C*H*$_2$Ph), 4.82 (4 H, app d, *J* = 12.4 Hz, 2 x C*H*$_2$Ph), 4.75 (2 H, d, *J* = 12.0 Hz, C*H*$_2$Ph), 4.69 (2 H, d, *J* = 11.6 Hz, C*H*$_2$Ph), 4.28 (2 H, q, *J* = 6.5 Hz, H-5), 4.15 (2 H, ddd, *J* = 10.1, 3.5, 0.9 Hz, H-2), 4.05 (2 H, dd, *J* = 10.2, 2.7 Hz, H-3), 3.72 (2 H, t, *J* = 1.3 Hz, H-4), 1.09 (6 H, d, *J* = 6.5 Hz, 3 x H-6); ^{13}C NMR (101 MHz, Chloroform-*d*) δ 139.0 (Ar C), 138.72 (Ar C), 138.71 (Ar C), 128.4 (Ar CH), 128.3 (Ar CH), 128.2 (Ar CH), 127.6 (Ar CH), 127.5 (Ar CH), 127.4 (Ar CH), 94.0 (C-1), 79.2 (C-3), 77.1 (C-4), 6.3 (C-2), 74.9 (C*H*$_2$Ph), 73.0 (C*H*$_2$Ph), 72.8 (C*H*$_2$Ph), 66.8 (C-5), 16.7 (C-6).

Data in agreement with previously reported synthesis.[28]

Synthesis of OEt derivatives S11 and S12 when EtOAc is used as solvent.

Ethyl 3,4,6-Tri-*O*-acetyl-2-deoxy-α-D-galactopyranose α-S11 and Ethyl 3,4,6-Tri-*O*-acetyl-2-deoxy-β-D-galactopyranose β-S11

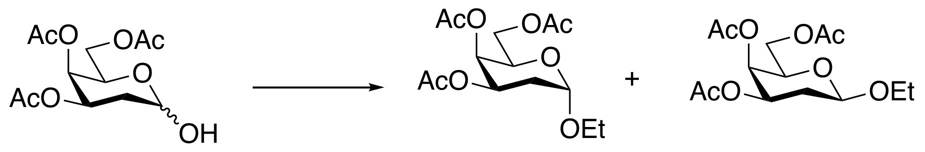

α-**S11** β-**S11**

Prepared according to general procedure A from **2b** (200 mg, 0.690 mmol), $AuCl_3$ (5 mg, 0.017 mmol, 2.5 mol%) and EtOAc (1 mL). After 4 hours, and following work up and purification via silica gel flash column chromatography (Hex/EtOAc 4:1) gave *the title compounds* **α-S11** (69 mg, 0.218 mmol, 32%) and **β-S11** (9 mg, 0.028 mmol, 4%);

Data for **α-S11:** R_f= 0.24 (2:1 Hexane/EtOAc); ^{1}H NMR (400 MHz, Chloroform-*d*) δ 5.35–5.31 (2 H, m, H-3, H-4), 5.04 (dd, *J* = 3.5, 0.7 Hz, H-1), 4.19 (1 H, t, *J* = 6.5 Hz, H-5), 4.12–4.10 (2 H, m, 2 x H-6), 3.72 (1 H, dq, *J* = 9.8, 7.1 Hz, 1 x CH_2CH_3), 3.50 (1 H, dq, *J* = 9.8, 7.1 Hz, 1 x CH_2CH_3), 2.15 (3 H, s, C(O)CH_3), 2.14–2.07 (1 H, m, H-2), 2.07 (3 H, s, C(O)CH_3), 2.00 (3 H, s, C(O)CH_3), 1.91–1.86 (1 H, m, H-2), 1.24 (3 H, t, *J* = 7.1 Hz, CH_2CH_3); ^{13}C NMR (101 MHz, Chloroform-*d*) δ 170.5 (C=O), 170.3 (C=)), 170.0 (C=O), 97.2 (C-1), 66.8 (CH), 66.6 (CH), 66.3 (CH), 63.2 (CH_2CH_3), 62.5 (C-6), 30.3 (C-2), 20.9 (C(O)CH_3), 20.7 (2 x C(O)CH_3), 15.0 (CH_2CH_3); MS (ES^+) found *m/z* 341.1 $[M+Na]^+$, HRMS (ESI) m/z: Calcd $C_{14}H_{22}O_8Na$ $[M+Na]^+$ 341.1207; Found 341.1200.

Data for **β-S11**: R_f= 0.17 (2:1 Hexane/EtOAc); ^{1}H NMR (400 MHz, Chloroform-*d*) δ 5.28 (1 H, dd, *J* = 3.4, 1.1 Hz, H-4), 5.05–4.99 (1 H, m, H-3), 4.61–4.58 (1 H, m, H-1), 4.23–4.14 (2 H, m, 2 x H-6), 4.00 (1 H, dq, *J* = 9.5, 71 Hz, 1 x CH_2CH_3), 3.82 (1 H, td, *J* = 6.7, 1.2 Hz, H-5), 3.60 (1 H, dq, *J* = 9.5, 71 Hz, 1 x CH_2CH_3), 2.15 (3 H, s, C(O)CH_3), 2.07 (3 H, s, C(O)CH_3), 2.03 (3 H, s, C(O)CH_3), 2.01–1.96 (2 H, m, 2 x H-2), 1.28 (3 H, t, *J* = 7.1 Hz, CH_2CH_3); ^{13}C NMR (101 MHz, Chloroform-*d*) δ 170.5 (C=O), 170.4 (C=)), 170.1 (C=O), 99.9 (C-1), 70.9 (C-5), 68.5 (C-3), 65.5 (CH_2CH_3), 65.2 (C-4), 61.8 (C-6), 32.1 (C-2), 20.8 (C(O)CH_3), 20.74 (C(O)CH_3), 20.71 (C(O)CH_3), 15.1 (CH_2CH_3); MS (ES^+) found *m/z* 341.1 $[M+Na]^+$, HRMS (ESI) m/z: Calcd $C_{14}H_{22}O_8Na$ $[M+Na]^+$ 341.1207; Found 341.1203.

Data for both anomers in agreement with previous synthesis.[29]

Ethyl 3,4,6-Tri-*O*-acetyl-2-deoxy-α/β-D-glucopyranose α/β-S12

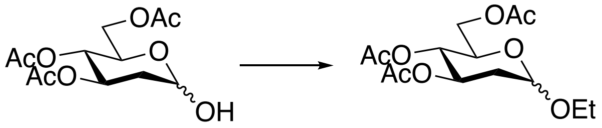

S12

Prepared according to general procedure A from **3b** (200 mg, 0.690 mmol), $AuCl_3$ (5 mg, 0.017 mmol, 2.5 mol%) and EtOAc (1 mL). After 3.5 hours and following work up and purification via silica gel flash column chromatography (Hex/EtOAc 4:1) gave *the title compound* **S12** (107 mg, 0.336 mmol, 49%, α/β = 7.5:1); R_f = 0.35 (Hexane/EtOAc 1:1); MS (ES^+) found *m/z* 341.1 $[M+Na]^+$, HRMS (ESI) m/z: Calcd $C_{14}H_{22}O_8Na$ $[M+Na]^+$ 341.1207; Found 341.1211.

NMR data for α-anomer: ^{1}H NMR (400 MHz, Chloroform-*d*) δ 5.29 (1 H, ddd, *J* = 11.6, 9.4, 5.4 Hz, H-3), 4.95 (1 H, t, *J* = 9.8 Hz, H-4), 4.92 (1 H, d, *J* = 2.8 Hz, H-1), 4.26 (1 H, dd, *J* = 12.2, 4.7 Hz, H-6a), 4.01 (1 H, dd, *J* = 12.2, 2.3 Hz, H-6b), 3.93 (1 H, ddd, *J* = 10.1, 4.6, 2.3 Hz, H-5), 3.65 (1 H, dq, *J* = 9.8, 7.1 Hz, 1 x CH_2CH_3), 3.43 (1 H, dq, *J* = 9.8, 7.1 Hz, 1 x CH_2CH_3), 2.18 (1 H, ddd, *J* = 12.9, 5.4, 1.1 Hz, H-2_{eq}), 2.05 (3 H, s, C(O)CH_3), 2.00 (3 H, s, C(O)CH_3), 1.97 (3 H, s, C(O)CH_3), 1.78 (1 H, ddd, *J* = 12.9, 11.7, 3.7 Hz, H-2_{ax}), 1.18 (3 H, t, *J* = 7.1 Hz, CH_2CH_3); ^{13}C NMR (101 MHz, Chloroform-*d*) δ 170.6 (C=O), 170.1 (C=O), 169.8 (C=O), 96.6 (C-1), 69.5 (C-4), 69.1 (C-3), 68.7 (C-5), 63.1 (CH_2CH_3), 62.4 (C-6), 35.2 (C-2), 20.9 (C(O)CH_3), 20.68 (C(O)CH_3), 20.66 (C(O)CH_3), 14.9 (CH_2CH_3).

NMR data for β-anomer: ^{1}H NMR (400 MHz, Chloroform-*d*) δ 5.02–4.92 (2 H, m, H-3, H-4), 4.54 (1 H, dd, *J* = 9.7, 2.0 Hz, H-1), 4.26 (1 H, dd, *J* = 12.2, 4.9 Hz, H-6a), 4.06 (1 H, dd, *J* = 12.2, 2.5 Hz, H-6b), 3.91–3.85 (1 H, m, 1 x CH_2CH_3), 3.57 (1 H, ddd, *J* = 9.5, 4.9, 2.5 Hz, H-5), 3.52 (1 H, dq, *J* = 9.5, 7.1 Hz, 1 x CH_2CH_3), 2.27 (1 H, ddd, *J* = 12.6, 5.0, 2.0 Hz, H-2_{eq}), 2.03 (3 H, s, C(O)CH_3), 1.99 (3 H, s, C(O)CH_3), 1.98 (3 H, s, C(O)CH_3), 1.74–1.66 (1 H, m, H-2_{ax}), 1.19 (3 H, t, *J* = 7.1 Hz, CH_2CH_3); ^{13}C NMR (101 MHz, Chloroform-*d*) δ 170.7 (C=O), 170.2 (C=O), 169.7 (C=O), 99.3 (C-1), 71.9 (C-5), 70.6 (C-3), 69.1 (C-4), 65.1 (CH_2CH_3), 62.4 (C-6), 36.2 (C-2), 20.8 (C(O)CH_3), 20.7 (C(O)CH_3), 15.0 (CH_2CH_3).

Data in agreement with previously reported synthesis.[30]

List of hemiacetal acceptors used for the synthesis of 2-deoxy trehalose derivatives

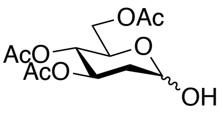

3b α/β = 10:1

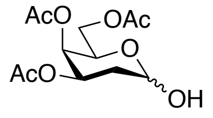

2b α/β = 3:1

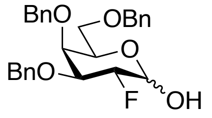

2c α/β = 3:1

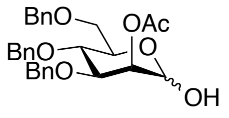

14 α/β = 6:1

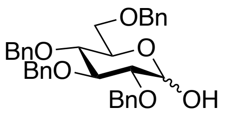

13a α/β = 5:1

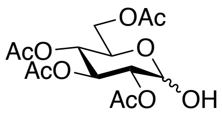

13b α/β = 3.3:1

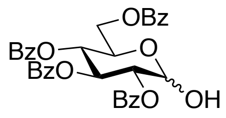

13c α/β = 5:1

13a was commercially available.
14 was prepared in four steps from per-acetylated mannose according to the procedure reported by Rintelamnn *et al.*[31]
13b and **13c** were synthesised from the corresponding per-acylated derivatives (1-*O*-Ac in the case of **13a**, 1-*O*-Bz in the case of **13b)** using dimethylaminopropylamine following using a procedure reported by Anderson *et al.*[32]
Data for **13b**[32] and **13c**[33] in agreement with previously reported syntheses.

Synthesis of unsymmetrical trehalose derivatives (Table 2)

3,4,6-Tri-*O*-benzyl-2-deoxy-α-D-galactopyranosyl 3',4',6'-tri-*O*-acetyl-2-deoxy-α-D-glucopyranoside 15a

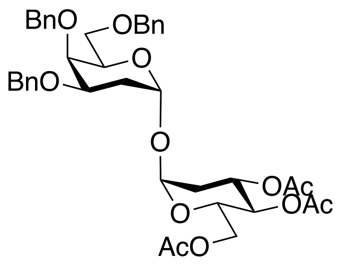

Prepared according to general procedure A from donor **2a** (344 mg, 0.79 mmol), acceptor **3b** (464 mg, 1.60 mmol), $AuCl_3$ (6 mg, 0.02 mmol) and toluene (4 mL). After 3.5 hours, and following work up and purification via silica gel flash column chromatography (4:1 to 3:1 Hex/EtOAc) gave anomeric benzyl **5f** (15 mg, 0.029 mmol, 4%), dimer **7** (6 mg, 0.007 mmol, <1%), *the title compound* **15a** as a clear oil (420 mg, 0.59 mmol, 75%) plus recovered acceptor **11a** (103 mg, 44% of amount used); R_f= 0.14 (Hexane/EtOAc 2:1); MS (ES^+) found *m/z* 724.3 $[M+ NH_4]^+$, HRMS (ESI) m/z: Calcd $C_{39}H_{46}O_{12}Na$ $[M+Na]^+$ 729.2887; Found 729.2893; ^1H NMR (400 MHz, Chloroform-*d*) δ 7.41–7.29 (15 H, m, Ar*H*), 5.36 (1 H, ddd, *J* = 11.7, 9.4, 5.4 Hz, H-3_{Glc2D}), 5.30 (1 H, d, *J* = 2.9 Hz, H-1_{Glc2D}), 5.27 (1 H, d, *J* = 3.1 Hz, H-1_{Gal2D}), 5.27 (1 H, t, *J* = 9.8 Hz, H-4_{Glc2D} 4.96 (1 H, d, *J* = 11.6 Hz, CH_2Ar), 4.71–4.62 (3 H, m, 3 x CH_2Ar), 4.50 (1 H, d, *J* = 11.8 Hz, CH_2Ar), 4.44 (1 H, d, *J* = 11.8 Hz, CH_2Ar), 4.32 (1 H, dd, *J* = 12.2, 4.9 Hz, H-6_{Glc2D}), 4.06 (1 H, dd, *J* = 12.2, 2.3 Hz, H-1_{Glc2D}), 4.02–3.95 (3 H, m, H-3_{Gal2D}, H-4_{Gal2D}, H-5_{Glc2D}), 3.89 (1 H, t, *J* = 6.4 Hz, H-5_{Gal2D}), 3.63–3.54 (2 H, m, 2 x H-6_{Gal2D}), 2.33 (1 H, td, *J* = 12.2, 3.4 Hz, H-$2_{Gal2D\ ax}$), 2.19 (1 H, ddd, *J* = 13.1, 5.4, 1.1 Hz, H-$2_{Glc2D\ eq}$), 2.10 (3 H, s, C(O)CH_3), 2.09 (3 H, s, C(O)CH_3), 2.06 (3 H, s,

$C(O)CH_3$), 1.97–1.86 (2 H, m, H-$2_{Glc2D\ ax}$, H-$2_{Gal2D\ eq}$); ^{13}C NMR (101 MHz, Chloroform-*d*) δ 170.7 (C=O), 170.3 (C=O), 169.8 (C=O), 138.7 (Ar C), 138.3 (Ar C), 138.0 (Ar C), 128.5 (Ar CH), 128.4 (Ar CH), 128.24 (Ar CH), 128.20 3(Ar CH), 127.74 (Ar CH), 127.71 (Ar CH), 127.63 (Ar CH), 127.57 (Ar CH), 127.5 (Ar CH),93.4 (C-1_{Gal2D}), 92.1 (C-1_{Glc2D}), 74.5 (C-3_{Gal2D}), 74.4 (CH_2Ar), 73.5 (CH_2Ar), 72.9 (C-5_{Glc2D} or C-4_{Gal2D}), 70.84 (C-5_{Gal2D}), 70.78 (CH_2Ar), 69.50 (C-4_{Glc2D}), 69.46 (C-6_{Gal2D}), 68.9 (C-3_{Glc2D}), 68.3 (C-5_{Glc2D} or C-4_{Gal2D}), 62.4 (C-6_{Glc2D}), 34.7 (C-2_{Glc2D}), 30.7 (C-2_{Gal2D}), 21.0 ($C(O)CH_3$), 20.7 (2 x $C(O)CH_3$).

3,4,6-Tri-*O*-benzyl-2-deoxy-α-D-galactopyranosyl 2',3',4',6'-tetra-*O*-benzyl-α- D-glucopyranoside 15b

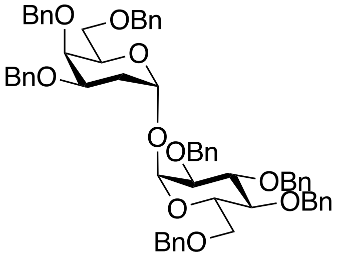

Prepared according to general procedure A from donor **2a** (86 mg, 0.20 mmol), acceptor **13a** (216 mg, 0.40 mmol), $AuCl_3$ (1.5 mg, 0.005 mmol) and toluene (2 mL). After 3 hours and following work up and purification via silica gel flash column chromatography (5:1 Hexane Hex/EtOAc) gave anomeric benzyl **5f** (13 mg, 0.025 mmol, 13%), *the title compound* **15b** as a clear oil (67 mg, 0.07 mmol, 35%) and recovered acceptor **13a** (136 mg, 63% of amount used); R_f= 0.22 (2:1 Hexane/EtOAc); MS (ES^+) found *m/z* 974.5 $[M+NH_4]^+$, HRMS (ESI) m/z: Calcd $C_{61}H_{68}O_{10}N$ $[M+NH_4]^+$ 974.4838; Found 974.4841; ^1H NMR (400 MHz, Chloroform-*d*) δ 7.42–7.21 (35 H, m, Ar*H*), 5.38 (2 H, app d, J = 3.5 Hz, H-1_{2DGal}, H-1_{Glc}), 5.02 (1 H, d, J = 11.0 Hz, CH_2Ph), 5.00 (1 H, d, J = 11.6 Hz, CH_2Ph), 4.90 (1 H, d, J = 10.7 Hz, CH_2Ph), 4.88 (1 H, d, J = 11.0 Hz, CH_2Ph), 4.79 (1 H, d, J = 12.0 Hz, CH_2Ph), 4.70–4.66 (4 H, m,), 4.62 (1 H, d, J = 11.9 Hz, CH_2Ph), 4.56 (1 H, d, J = 10.7 Hz, CH_2Ph), 4.55 (1 H, d, J = 12.2 Hz, CH_2Ph), 4.50 (1 H, d, J = 11.8 Hz, CH_2Ph), 4.42 (1 H, d, J = 11.8 Hz, CH_2Ph), 4.29 (1 H, t, J = 6.6 Hz, H-5_{2DGal}), 4.10 (1 H, ddd, J = 12.1, 4.4, 2.4 Hz, H-3_{2DGal}), 4.04–3.99 (2 H, m, H-3_{Glc}, H-4_{2DGal}), 3.86 (1 H, ddd, J = 9.9, 3.2, 1.8 Hz, H-5_{Glc}), 3.82 (1 H, dd, J = 10.6, 3.5 Hz, H-6_{Glc}), 3.77–3.70 (2 H, m, H-4_{Glc}, H-6_{Glc}), 3.66–3.61 (3 H, m, H-2_{Glc}, 2 x H-6_{2DGal}), 2.36 (td, J = 12.4, 3.6 Hz, H-$2_{2DGal\ ax}$), 1.99 (1 H, dd, J = 12.6, 4.5 H-$2_{2DGal\ eq}$); ^{13}C NMR (101 MHz, Chloroform-*d*) δ 138.93 (Ar C), 138.91 (Ar C), 138.5 (Ar C), 138.22 (Ar C), 138.19 (Ar C), 138.18 (Ar C), 138.0 (Ar C), 128.5 (Ar CH), 128.43 (Ar CH), 128.40 (Ar CH), 128.3 (Ar CH), 128.2 (Ar CH), 128.1 (Ar CH), 128.0 (Ar CH), 128.85 (Ar CH), 128.83 (Ar CH), 128.79 (Ar CH), 128.74 (Ar CH), 128.61 (Ar CH), 128.59 (Ar CH), 128.56 (Ar CH), 128.5 (Ar CH), 93.8 (C-1_{2DGal} or C-1_{Gal}), 92.2 (C-1_{2DGal} or C-1_{Gal}), 81.7 (C-3_{Glc}), 79.3 (C-2_{Glc}), 75.5 (C-4_{Glc}), 75.3 (CH_2Ph), 74.4 (C-3_{2DGal} and CH_2Ph), 73.6 (CH_2Ph), 73.2 (2 x CH_2Ph), 72.5 (C-4_{2DGal}), 70.9 (CH_2Ph), 70.6 (C-5_{Glc}), 70.6 (CH_2Ph), 70.5 (C-5_{2DGal}), 69.7 (C-62D_{Gal}), 68.5 (C-6_{Glc}), 31.0 (C-2_{2DGal}).

3,4,6-Tri-*O*-benzyl-2-deoxy-α-D-galactopyranosyl 3',4',6'-tri-*O*-benzyl-2-*O*-acetyl-α-D-mannopyranoside 15c

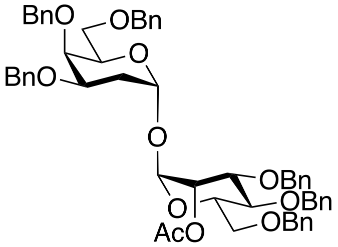

Prepared according to general procedure A from donor **2a** (199 mg, 0.457 mmol), acceptor **14** (450 mg, 0.914 mmol, 2 eq.), $AuCl_3$ (3.5 mg, 0.012 mmol) and toluene (2.5 mL). After 2.5 hours and following work up and purification via silica gel flash column chromatography (5:1 Hexane/EtOAc) gave *the title compound* **15c** as a colourless oil (241 mg, 0.265 mmol, 58%) plus recovered **14** (225 mg, 50% of amount used); R_f = 0.40 (2:1 Hexane/EtOAc); MS (ES^+) found *m/z* 931.4 $[M+Na]^+$, HRMS (ESI) m/z: Calcd $C_{56}H_{60}O_{11}Na$ $[M+Na]^+$ 931.4028; Found 931.4046; ^1H NMR (400 MHz, Chloroform-*d*) δ 7.42–7.25 (30 H, m, ArCH), 5.36–5.34 (2 H, m, H-1_{Gal2D}, H-2_{Man}), 5.21 (1H, d, *J* = 1.8 Hz, H-1_{Man}), 5.00 (1H, d, *J* = 11.5 Hz, CH_2Ph), 4.94 (1H, d, *J* = 10.6 Hz, CH_2Ph), 4.79–4.56 (9 H, m, 9 x CH_2Ph), 4.50 (1 H, d, *J* = 11.7 Hz, CH_2Ph), 4.04 (1 H, br s, H-4_{Gal2D}), 4.00–3.95 (3 H, m, H-3_{Man}, H-4_{Man}, H-5_{Gal2D}), 3.91–3.82 (3 H, m, H-3_{Gal2D}, H-5_{Man}, H-6_{Man}), 3.77 (1 H, dd, *J* = 9.2, H-6_{Man}), 3.71 (1H, dd, *J* = 9.0, 7.6 Hz, , H-6_{Gal2D}), 3.64 (1H, dd, *J* = 9.2, 5.8 Hz, H-6_{Gal2D}), 2.34 (1H, td, *J* = 12.4, 3.7 Hz, H-$2_{2DGal\ ax}$), 2.19 (3H, s, C(O)CH_3), 1.95 (1H, dd, *J* = 12.8, 4.4 Hz, H-$2_{2DGal\ eq}$); ^{13}C NMR (101 MHz, Chloroform-*d*) δ 170.2 (C=O), 138.8 (Ar C), 138.3 (Ar C), 138.2 (Ar C), 138.1 (Ar C), 137.9 (Ar C), 128.45 (Ar CH), 128.41 (Ar CH), 128.38 (Ar CH), 128.35 (ArCH), 128.33 (Ar CH), 128.2 (Ar CH), 128.1 (Ar CH), 128.0 (ArCH), 127.81 (Ar CH), 127.78 (ArCH), 127.77 (Ar CH), 127.74 (ArCH), 127.62 (Ar CH),127.60 (Ar CH), 127.59 (Ar CH), 127.5 (Ar CH), 127.4 (Ar CH), 93.5 (C-1_{2DGal}), 93.2 (C-1_{Man}), 77.9 (CH), 75.4 (CH$_2$Ph). 74.4 (CH$_2$Ph), 74.3 (CH), 74.1 (CH), 73.5 (CH$_2$Ph), 73.4 (CH$_2$Ph), 72.8 (C4_{Gal2D}), 72.1 (CH), 71.9 (CH$_2$Ph), 70.6 (CH), 70.4 (CH$_2$Ph), 69.0 (C-6_{Gal2D}), 68.8 (C-6_{Man}), 68.7 (C-2_{Man}), 30.6 (C2_{2DGal}), 21.09 (C(O)CH$_3$).

3,4,6-Tri-*O*-benzyl-2-deoxy-α-D-galactopyranosyl 2’,3’,4’,6’-tetra-*O*-acetyl-α- D-glucopyranoside 15d

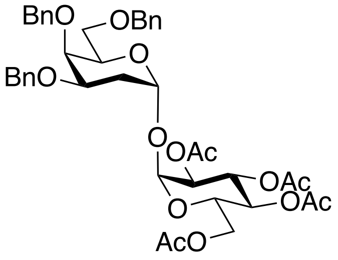

Prepared according to general procedure A from donor **2a** (318 mg, 0.73 mmol), acceptor **13b** (509 mg, 1.46 mmol), $AuCl_3$ (5.5 mg, 0.018 mmol) and toluene (4.5 mL). After 2 hours, and following work up and purification via silica gel flash column chromatography (Hex/EtOAc 4:1) gave anomeric benzyl **5f** (10 mg, 0.02 mmol, 3%), the *title compound* **15d** as a clear oil (424 mg, 0.55 mmol, 76%) plus recovered acceptor **13b** (280 mg, 55% of amount used); R_f= 0.23 (Hexane/EtOAc 1:1); MS (ES^+) found *m/z* 787.3 $[M + Na]^+$, HRMS (ESI) m/z: Calcd $C_{41}H_{48}O_{14}Na$ $[M+Na]^+$ 787.2936; Found 787.2930; ^{1}H NMR (400 MHz, Chloroform-*d*) δ 7.43–7.25 (15 H, Ar*H*), 5.51 (1 H, dd, *J* = 10.2, 9.5 Hz, H-3_{Glc}), 5.35 (1 H, d, *J* = 3.8 Hz, H-1_{Glc}), 5.26 (1 H, d, *J* = 3.1 Hz, H-1_{2DGal}), 5.12 (1 H, dd, *J* = 10.1, 9.5 Hz, H-4_{Glc}), 5.04 (1 H, dd, *J* = 10.3, 3.8 Hz, H-2_{Glc}), 4.95 (1 H, *J* = 12.0 Hz, C*H*$_2$Ph), 4.73 (1 H, *J* = 11.5 Hz, C*H*$_2$Ph), 4.69 (1 H, *J* = 11.6 Hz, C*H*$_2$Ph), 4.63 (1 H, *J* = 11.6 Hz, C*H*$_2$Ph), 4.45 (1 H, *J* = 11.8 Hz, C*H*$_2$Ph), 4.38 (1 H, *J* = 11.8 Hz, C*H*$_2$Ph), 4.30 (1 H, dd, *J* = 12.3, 4.4 Hz, H-6_{Glc}), 4.11 (1 H, dd, *J* = 12.3, 2.3 Hz, H-6_{Glc}), 4.08–4.01 (2 H, m, H-3_{2DGal}, H-5_{Glc}), 4.00 (br s, 1 H, H-4_{2DGal}), 3.96 (1 H, t, *J* = 6.5 Hz, H-5_{2DGal}), 3.59 (1 H, dd, 9.1, 7.3 Hz, H-6_{2DGal}), 3.46 (1 H, dd, *J* = 9.1, 3.5 Hz, H-6_{2DGal}), 2.36 (1 H, td, *J* = 12.4, 3.5, H-$2_{2DGal\ ax}$), 2.11 (3 H, s, C(O)C*H*$_3$), 2.09 (3 H, s, C(O)C*H*$_3$), 2.05 (3 H, s, C(O)C*H*$_3$), 1.98–1.93 (4 H, m, H-2_{2DGal}, C(O)C*H*$_3$); ^{13}C NMR (101 MHz, Chloroform-*d*) 170.6 (C=O), 170.3 (C=O), 169.8 (C=O), 169.5 (C=O), 138.7 (Ar C), 138.2 (Ar C), 137.8 (Ar C), 128.5 (Ar CH), 128.4 (Ar CH), 128.3 (Ar CH), 128.2 (Ar CH), 127.8 (Ar CH), 127.75 (Ar CH), 127.68 (Ar CH), 128.63 (Ar CH), 128.61 (Ar CH), 94.4 (C-1_{2DGal}), 91.7 (C-1_{Glc}), 74.4 (C-3_{2DGal} or C-5_{Glc}), 74.3 (*C*H$_2$Ph), 73.5 (*C*H$_2$Ph), 72.7 (C-4_{2DGal}), 70.93 (C-5_{2DGal}), 70.89 (*C*H$_2$Ph), 70.2 (C-3_{Glc}), 69.9 (C-2_{Glc}), 69.1 (C-6_{2DGal}), 68.6 (C-4_{Glc}), 67.8 (C-3_{2DGal} or C-5_{Glc}), 61.8 (C-6_{Glc}), 30.85 (C-2_{2DGal}), 20.73 (C(O)*C*H$_3$), 20.71 (C(O)*C*H$_3$), 20.6 (C(O)*C*H$_3$), 20.4 (C(O)*C*H$_3$).

3,4,6-Tri-*O*-benzyl-2-deoxy-α-D-galactopyranosyl 2',3',4',6'-tetra-*O*-benzoyl-α- D-glucopyranoside 15e

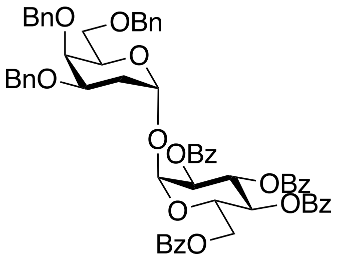

Prepared according to general procedure A from donor **2a** (289 mg, 0.67 mmol), acceptor **13c** (795 mg, 1.33 mmol), $AuCl_3$ (5 mg, 0.017 mmol) and toluene (4 mL). After 4 hours and following work up and purification via silica gel flash column chromatography (Hex/EtOAc 4:1) gave *the title compound* **15e** as a clear oil (367 mg, 0.36 mmol, 54%); R_f = 0.34 (2:1 Hexane/EtOAc); MS (ES$^+$) found *m/z* 1035.4 [M+Na]$^+$, HRMS (ESI) m/z: Calcd $C_{61}H_{56}O_{14}Na$ [M+Na]$^+$ 1035.3568; Found 1035.3530; ^{1}H NMR (400 MHz, Chloroform-*d*) δ 8.10–8.03 (4 H, m, Ar*H*), 8.00–7.95 (4 H, m, Ar*H*), 7.62–7.26 (25 H, m, Ar*H*), 7.13–7.11 (2 H, m, Ar*H*), 6.29 (1 H, t, *J* = 9.9 Hz, H-3$_{Glc}$), 5.80–5.75 (2 H, m, H-1$_{Glc}$, H-4$_{Glc}$), 5.51 (1 H, dd, *J* = 10.3, 3.9 Hz, H-2$_{Glc}$), 5.43 (1 H, d, *J* = 3.0 Hz, H-1$_{Gal2D}$), 4.92 (1 H, d, *J* = 11.5 Hz, C*H*$_2$Ph), 4.76 (2 H, s, 2 x C*H*$_2$Ph), 4.68–4.54 (4 H, m, H-5$_{Glc}$, 2 x H-6$_{Glc}$, C*H*$_2$Ph), 4.19–4.11 (3 H, m, H-3$_{Gal2D}$, 2 x C*H*$_2$Ph), 3.87–3.84 (2 H, m, H-4$_{Gal2D}$, H-5$_{Gal2D}$), 3.45 (1 H, dd, *J* = 8.9, 8.2 Hz, H-6$_{Gal2D}$), 3.24 (1 H, dd, *J* = 9.0, 5.7 Hz, H-6$_{Gal2D}$), 2.42 (1 H, td, *J* = 12.5, 3.5 Hz, H-2$_{Gal2D\ ax}$), 2.14 (1 H, dd, *J* = 12.8, 4.5 Hz, H-2$_{Gal2D\ eq}$); ^{13}C NMR (101 MHz, Chloroform-*d*) δ 166.2 (C=O), 165.9 (C=O), 165.5 (C=O), 165.4 (C=O), 138.8 (Ar C), 138.3 (Ar C), 138.1 (Ar C), 133.53 (Ar CH), 133.48 (Ar CH), 133.3 (Ar CH), 133.2 (Ar CH), 129.9 (Ar CH), 129.8 (Ar CH), 129.73 (Ar CH), 129.70 (Ar CH), 129.6 (Ar C), 129.1 (Ar C), 128.9 (Ar C), 128.85 (Ar C), 128.6 (Ar CH), 128.51 (Ar CH), 128.49 (Ar CH), 128.4 (Ar CH), 128.2 (Ar CH), 128.1 (Ar CH), 127.79 (Ar CH), 127.77 (Ar CH), 127.49 (Ar CH), 127.45 (Ar CH), 94.4 (C-1$_{Glc}$), 91.6 (C-1$_{Gal2D}$), 74.4 (*C*H$_2$Ph), 74.3 (C-3$_{Gal2D}$), 73.0 (*C*H$_2$Ph), 72.9 (C-4$_{Gal2D}$ or C-5$_{Gal2D}$ or C-3$_{Glc}$), 71.4 (C-2$_{Glc}$), 70.9 (*C*H$_2$Ph), 70.49 (C-4$_{Gal2D}$ or C-5$_{Gal2D}$ or C-3$_{Glc}$), 70.45 (C-4$_{Gal2D}$ or C-5$_{Gal2D}$ or C-3$_{Glc}$), 69.7 (C-4$_{Gal}$), 68.41 (C-5$_{Glc}$), 68.39 (C-6$_{Gal2D}$), 63.1 (C-6$_{Glc}$), 30.9 (C-2$_{Gal2D}$).

3,4,6-Tri-*O*-benzyl-2-deoxy-α-D-galactopyranosyl 3',4',6'-tri-*O*-benzyl-2-deoxy-2-fluoro-α-D-galactopyranoside 15f

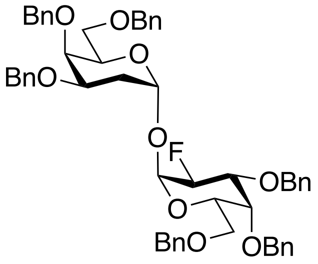

Prepared according to general produce A from donor **2a** (289 mg, 0.666 mmol), acceptor **2c** (603 mg, 1.332 mmol), toluene (4 mL) and $AuCl_3$ (5 mg, 0.17 mmol). After 4 h at 50 °C and following work up and purification by silica gel flash column chromatography (4:1 Hex/EA) to give the *title compound* **15f** (291 mg, 0.45 mmol, 68%). R_f = 0.23 (Hexane/EtOAc 2:1); MS (ES$^+$) found *m/z* 891.4 [M+Na]$^+$, HRMS (ESI) m/z: Calcd $C_{54}H_{57}O_{19}FNa$ [M+Na]$^+$ 891.3879; Found 891.3888; ^{1}H NMR (400 MHz, Chloroform-*d*) δ 7.42–7.22 (30 H, m, Ar*H*), 5.33 (1 H, d, *J* = 3.9 Hz, H-1$_{Gal}$), 5.36 (1 H, d, *J* = 2.9 Hz, H-1$_{Gal2D}$), 4.98 (1 H, ddd, *J* = 50.1, 9.8, 4.0 Hz, H-2$_{Gal}$), 4.96–4.92 (2 H, m, 2 x C*H*$_2$Ph), 4.77 (1 H, d, *J* = 12.0 Hz, C*H*$_2$Ph), 4.68 (1 H, d, *J* = 11.9 Hz, C*H*$_2$Ph), 4.65–4.40 (8 H, m, 8 x C*H*$_2$Ph), 4.09 (1 H, t, *J* = 6.8 Hz, H-5$_{Gal}$), 4.03 (1 H, t, *J* = 3.0 Hz, H-4 $_{Gal}$), 3.98 (1 H, s, H-4$_{Gal2D}$), 3.96–3.89 (3 H, m, H-3$_{Gal}$, H-3$_{Gal2D}$, H-5$_{Gal2D}$), 3.65–3.51 (4 H, m, 2 x H-6$_{Gal2D}$, 2 x H-6$_{Gal}$), 2.29 (1 H, td, 12.4, 3.4 Hz, H-2$_{Gal2D\ ax}$), 1.89 (1 H, dd, *J* = 12.6, 4.5 Hz, H-2$_{Gal2D\ eq}$); ^{13}C NMR (101 MHz, Chloroform-*d*) δ 138.9 (Ar C), 138.43 (Ar C), 138.40 (Ar C), 138.3 (Ar C), 138.2 (Ar C), 137.8 (Ar C), 128.5 (Ar CH), 128.42 (Ar CH), 128.40 (Ar CH), 128.37 (Ar CH), 128.3 (Ar CH), 128.20 (Ar CH), 128.15 (Ar CH), 128.1 (Ar CH), 127.9 (Ar CH), 127.8 (Ar CH), 127.72 (Ar CH), 127.67 (Ar CH), 127.66 (Ar CH), 127.60 (Ar CH), 127.57 (Ar CH), 127.51 (Ar CH), 127.48 (Ar CH), 127.40 (Ar CH), 93.8 (C-1$_{Gal2D}$), 91.9 (d, *J* = 21.9 Hz, C-1$_{Gal}$) 88.8 (d, *J* = 188.1 Hz, C-2$_{Gal}$), 76.7 (d, *J* = 17.0 Hz, C-3$_{Gal}$), 75.4 (d, *J* = 8.3 Hz. C-4$_{Gal}$), 75.0 (*C*H$_2$Ph), 74.4 (*C*H$_2$Ph), 74.3 (C-3$_{Gal2D}$), 73.6 (*C*H$_2$Ar), 73.2 (*C*H$_2$Ph), 72.9 (C-4$_{Gal2D}$), 72.83 (*C*H$_2$Ph), 72.81 (d, *J* = 1.7 Hz, *C*H$_2$Ph), 70.5 (*C*H$_2$Ph), 70.06 (d. *J* = 1.3 Hz, C-5$_{Gal}$), 70.05 (C-5$_{Gal2D}$), 69.1, 68.4 (C-6$_{Gal}$ and C-6$_{Gal2D}$), 30.9 (C-2$_{Gal2D}$); ^{19}F NMR (376 MHz, Chloroform-*d*) δ -207.3 (ddd, *J* = 50.3, 10.1, 4.1 Hz).

6-*O*-Acetyl-3,4-di-*O*-benzyl-2-deoxy-α-D-glucopyranosyl 2',3',4',6'-tetra-*O*-benzyl-α-D-glucopyranoside 16

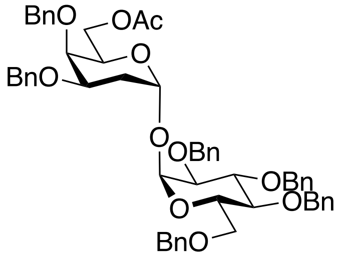

Prepared according to general procedure A from donor **2d** (463 mg, 1.2 mmol), acceptor **13a** (1.30 g, 2.4 mmol), toluene (6 mL), $AuCl_3$ (9 mg, 0.03 mmol). After 6 h and following work up and purification by silica gel flash column chromatography (2 columns; column 1: 4:1 Hexane/EtOAc, column 2 5:1 to 4:1 Hexane/EtOAc) gave the *title compound* **16** as a clear oil (593 mg, 0.65 mmol, 54%); R_f = 0.31 (Hexane/EtOAc 2:1); MS (ES$^+$) found *m/z* 926.4 $[M+NH_4]^+$, HRMS (ESI) m/z: Calcd $C_{56}H_{64}O_{11}N$ $[M+Na]^+$ 926.4474; Found 926.4468; ^{1}H NMR (400 MHz, Chloroform-*d*) δ 7.44–7.30 (28 H, m, Ar*H*), 7.21–7.19 (2 H, m, Ar*H*), 5.35 (1 H, d, *J* = 2.7 Hz, H-1_{2DGal}), 5.34 (1 H, d, *J* = 3.6 Hz, H-1_{Glc}), 4.99 (1 H, d, *J* = 11.6 Hz, CH_2Ph), 4.98 (1 H, d, *J* = 11.0 Hz, CH_2Ph), 4.88 (1 H, d, *J* = 10.6 Hz, CH_2Ph), 4.87 (1 H, d, *J* = 11.0 Hz, CH_2Ph), 4.75 (1 H, d, *J* = 11.9 Hz, CH_2Ph), 4.70–4.65 (5 H, m, 5 x CH_2Ph), 4.54 (1 H, d, *J* = 10.6 Hz, CH_2Ph), 4.53 (1 H, d, *J* = 12.1 Hz, CH_2Ph), 4.22–4.15 (2 H, m, H-5_{2DGal}, H-6_{2DGal}), 4.09 (1 H, ddd, *J* = 12.0, 4.3, 2.4 Hz, H-3_{2DGal}), 4.03 (1 H, dd, *J* = 9.5, 4.9 Hz, H-6_{2DGal}), 3.99 (1 H, t, *J* = 9.3 Hz, H-3_{Glc}), 3.85 (1 H, br s, H-4_{2DGal}), 3.83–3.68 (4 H, m, H-4_{Glc}, H-5_{Glc}, 2 x H-6_{Glc}), 3.65 (1 H, dd, *J* = 9.7, 3.6 Hz, H-2_{Glc}), 2.34 (1 H, td, *J* = 12.4, 3.5 Hz, H-$2_{2DGal\ ax}$), 2.00–1.96 (1 H, m, H-$2_{2DGal\ eq}$), 1.90 (3 H, s, C(O)CH_3); ^{13}C NMR (101 MHz, Chloroform-*d*) δ 170.3 (C=O), 138.9 (Ar C), 138.5 (Ar C), 138.3 (Ar C), 138.14 (Ar C), 138.08 (Ar C), 137.9 (Ar C), 128.49 (Ar CH), 128.46 (Ar CH), 128.42 (Ar CH), 128.39 (Ar CH), 128.3 (Ar CH), 128.1 (Ar CH), 127.9 (Ar CH), 127.85 (Ar CH), 127.78 (Ar CH), 127.74 (Ar CH), 127.70 (Ar CH), 127.687(Ar CH), 127.6 (Ar CH), 127.50 (Ar CH), 127.45 (Ar CH), 93.6 (C-1_{2DGal}), 92.1 (C-1_{Glc}), 81.7 (C-3_{Glc}), 79.4 (C-2_{Glc}), 77.7 (C-4_{Glc}), 75.5 (*C*H_2Ph), 75.3 (*C*H_2Ph), 74.3 (C-3_{2DGal}), 74.1 (*C*H_2Ph), 73.6 (*C*H_2Ph), 72.6 (*C*H_2Ph), 72.5 (C-4_{2DGal}), 71.0 (C-5_{Glc}), 70.7 (*C*H_2Ph), 69.2 (C-5_{Glc}), 68.4 (C-6_{Glc}), 63.7 (C-6_{2DGal}), 30.7 (C-2_{2DGal}), 20.8 (C(O)*C*H_3).

3,4,6-Tri-*O*-benzyl-2-deoxy-α-D-glucopyranosyl 2',3',4',6'-tetra-*O*-benzyl-α- D-glucopyranoside 17a

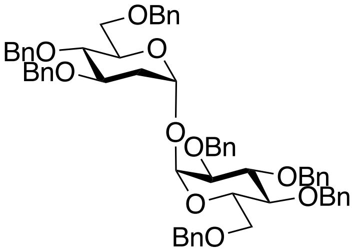

Prepared according to general procedure A from donor **3a** (86 mg, 0.20 mmol), acceptor **13a** (216 mg, 0.40 mmol), $AuCl_3$ (1.5 mg, 0.005 mmol, 2.5 mol%) and toluene (2 mL). After 3 hours and following work up and purification via silica gel flash column chromatography (2 columns. Column 1: 4:1 Hexane/EtOAc, Column 2: 5:1 Hexane/EtOAc) to give anomeric benzyl **7f** (4 mg, 0.008 mmol, 4%), dimer **11** (16 mg, 0.019 mmol, 5%), *the title compound* **17a** as a clear oil (138 mg, 0.14 mmol, 72%) and recovered acceptor (107 mg, 50% of amount used); R_f = 0.19 (Hexane/EtOAc 2:1); MS (ES$^+$) found *m/z* 974.5 $[M+NH_4]^+$, HRMS (ESI) m/z: Calcd $C_{61}H_{68}O_{10}N$ $[M+ NH_4]^+$ 974.4838; Found 974.4839; ^{1}H NMR (400 MHz, Chloroform-*d*) δ 7.43–7.25 (35 H, m, Ar*H*), 5.35 (2 H, app d, *J* = 3.5 Hz, H-1_{2DGlc}, H-1_{Glc}), 5.05 (1 H, d, *J* = 10.9 Hz, CH_2Ph), 5.00–4.90 (3 H, m, 3 x CH_2Ph), 4.75–4.56 (9 H, m, 9 x CH_2Ph), 4.53 (1 H, d, *J* = 12.1 Hz, CH_2Ph), 4.19 (1 H, ddd, *J* = 10.0, 3.5, 1.8 Hz, H-5_{2DGlc}), 4.13 (1 H, ddd, *J* = 11.4, 8.9, 5.0 Hz, H-3_{2DGlc}), 4.05 (1 H, t, *J* = 9.3 Hz, H-3_{Glc}), 3.88–3.67 (6 H, m, H-4_{2DGlc}, H-4_{Glc}, H-5_{Glc}, H-6_{2DGlc}, 2 x H-6_{Glc}), 3.66 (1 H, dd, *J* = 9.7, 3.6 Hz, H-2_{Glc}), 3.58 (1 H, dd, *J* = 10.5, 1.9 Hz, H-6_{2DGlc}), 2.27 (1 H, ddd, *J* = 12.9, 5.0, 0.9 Hz, H-$2_{2DGlc\ eq}$), 1.86 (1 H, ddd, *J* = 13.0, 11.6, 3.6 Hz, H-$2_{2DGlc\ ax}$); ^{13}C NMR (101 MHz, Chloroform-*d*) δ 138.9 (Ar C), 138.7 (Ar C), 138.21 (Ar C), 138.19 (Ar C), 138.1 (Ar C), 138.0 (Ar C), 128.50 (Ar CH), 128.46 (Ar CH), 128.43 (Ar CH), 128.39 (Ar CH), 128.37 (Ar CH), 128.33 (Ar CH), 128.1 (Ar CH), 128.0 (Ar CH), 127.9 (Ar CH), 127.8 (Ar CH), 127.64 (Ar CH), 127.60 (Ar CH), 127.59 (Ar CH), 127.56 (Ar CH), 93.8 (C-1_{2DGlc} or C-1_{Glc}), 92.9 (C-1_{2DGlc} or C-1_{Glc}), 81.8 (C-3_{Glc}), 79.3 (C-2_{Glc}), 78.4 (CH), 77.8 (CH), 77.3 (C-3_{2DGlc}), 75.6 (*C*H$_2$Ph), 75.4 (*C*H$_2$Ph), 75.0 (*C*H$_2$Ph), 73.6 (2 x *C*H$_2$Ph), 72.8 (*C*H$_2$Ph), 72.1 (*C*H$_2$Ph), 71.3 (C-5_{2DGlc}), 71.0 (CH), 68.7 (C-6_{2DGlc}), 68.5 (C-6_{Glc}), 35.5 (C-2_{2DGlc}).

3,4,6-Tri-*O*-benzyl-2-deoxy-α-D-glucopyranosyl 2',3',4',6'-tetra-*O*-acetyl-α-D-glucopyranoside 17b

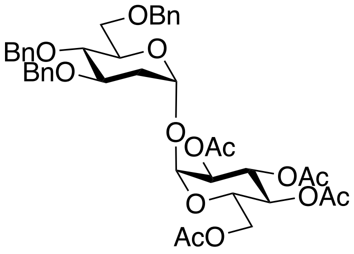

Prepared according to general procedure A from donor **3a** (642 mg, 1.48 mmol), acceptor **13b** (1.03 g, 2.99 mmol), $AuCl_3$ (11 mg, 0.037 mmol) and toluene (22 mL). After 6 hours and following work up and purification via silica gel flash column chromatography (11:1 to 7:1 Toluene/EtOAc) to give *the title compound* **17b** (406 mg, 0.53 mmol, 36%) plus recovered acceptor **13b** (580 mg, 58% of amount used); R_f = 0.21 (Hexane/EtOAc 1:1); MS (ES$^+$) found *m/z* 787.3 [M+Na]$^+$, HRMS (ESI) m/z: Calcd $C_{41}H_{48}O_{14}Na$ [M+Na]$^+$ 787.2936; Found 787.2928; ^{1}H NMR (400 MHz, Chloroform-*d*) δ 7.42–7.22 (15 H, m, Ar*H*), 5.52 (1 H, dd, *J* = 10.1, 9.6 Hz, H-3$_{Glc}$), 5.38 (1 H, d, *J* = 3.8 Hz, H-1$_{Glc}$), 5.25 (1 H, d, *J* = 2.6 Hz, H-1$_{2DGlc}$), 5.12 (1 H, t, *J* = 9.8 Hz, H-4$_{Glc}$), 5.04 (1 H, dd, *J* = 10.3, 3.8 Hz, H-2$_{Glc}$), 4.93 (1 H, d, *J* = 11.0 Hz, C*H*$_2$Ph), 4.76–4.71 (2 H, m, 2 x C*H*$_2$Ph), 4.64–4.50 (**3** H, m, **3** x C*H*$_2$Ph), 4.30 (1 H, dd, *J* = 12.3, 4.6 Hz, H-6$_{Glc}$), 4.12–3.99 (3 H, m, H-3$_{2DGlc}$, H-5$_{Glc}$, H-6$_{Glc}$), 3.78–3.71 (2 H, m, H-5$_{2DGlc}$, H-6$_{2DGlc}$), 3.65 (2 H, app t, *J* = 9.0 Hz, H-4$_{2DGlc}$, H-6$_{2DGlc}$), 2.22–2.17 (1 H, m, H-2$_{2DGlc\ eq}$), 2.12 (3 H, s, C(O)C*H*$_3$), 2.10 (3 H, s, C(O)C*H*$_3$), 2.06 (3 H, s, C(O)C*H*$_3$), 2.02 (3 H, s, C(O)C*H*$_3$), 1.84 (1 H, ddd, *J* = 13.2, 11.5, 3.7 Hz, H-2$_{2DGlc\ ax}$); ^{13}C NMR (101 MHz, Chloroform-*d*) δ 170.6 (C=O), 170.2 (C=O), 169.9 (C=O), 169.5 (C=O), 138.47 (Ar C), 138.45 (Ar C), 138.0 (Ar C), 129.0 (Ar CH), 128.5 (Ar CH), 128.4 (Ar CH), 128.3 (Ar CH), 128.2 (Ar CH), 128.0 (Ar CH), 127.83 (Ar CH), 127.81 (Ar CH), 127.69 (Ar CH), 127.67 (Ar CH), 127.62 (Ar CH), 125.3 (Ar CH), 93.6 (C-1$_{2DGlc}$), 91.4 (C-1$_{Glc}$), 77.9 (C-4$_{2DGlc}$), 77.0 (C-5$_{Glc}$), 75.0 (*C*H$_2$Ph), 73.6 (*C*H$_2$Ph), 72.4 (*C*H$_2$Ph), 72.1 (C-5$_{2DGlc}$), 70.1 (C-3$_{Glc}$), 69.9 (C-2$_{Glc}$), 68.7 (C-4$_{Glc}$), 68.6 (C-6$_{2DGlc}$), 67.8 (C-3$_{2DGlc}$), 61.8 (C-6$_{Glc}$), 35.2 (C-2$_{2DGlc}$), 20.9 (C(O)*C*H$_3$), 20.7 (C(O)*C*H$_3$), 20.64 (C(O)*C*H$_3$), 20.61 (C(O)*C*H$_3$).

3,4,6-Tri-*O*-benzyl-2-deoxy-α-D-glucopyranosyl 2',3',4',6'-tetra-*O*-benzoyl-α-D-glucopyranoside 17c

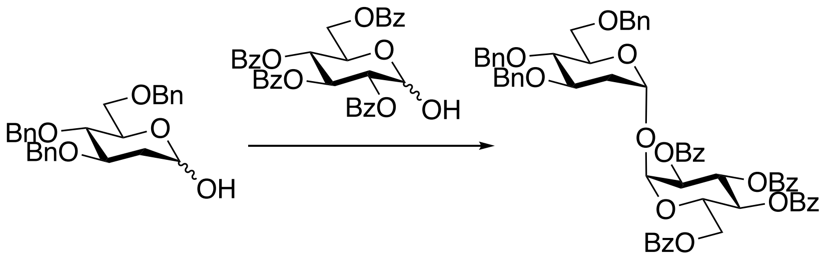

Prepared according to general procedure A from donor **3a** (795 mg, 1.83 mmol), acceptor **13c** (2.2 g, 3.67 mmol), $AuCl_3$ (14 mg, 0.047 mmol) and toluene (28 mL). After 4.5 hours and following work up and purification via silica gel flash column chromatography (2 columns Column 1: 15:1 Toluene/EtOAc, Column 2: 20:1 Toluene/EtOAc) to give anomeric benzyl **7f** (230mg, 0.44 mmol, 24%), the *title compound* **17c** as a clear oil (597 mg, 0.59 mmol, 32%) plus recovered acceptor **13c** (1.72 g, 78% of amount used; R_f = 0.33 (Hexane/EtOAc 2:1); MS (ES$^+$) found *m/z* 1035.4 [M+Na]$^+$, HRMS (ESI) m/z: Calcd $C_{61}H_{56}O_{14}Na$ [M+Na]$^+$ 1035.3562; Found 1035.3559; ^{1}H NMR (400 MHz, Chloroform-*d*) δ 8.07–7.90 (8 H, m, Ar*H*), 7.61–7.53 (2 H, m, Ar*H*), 7.49–7.18 (23 H, m, Ar*H*), 7.08–7.05 (2 H, m, Ar*H*), 6.22 (1 H, t, *J* = 10.0 Hz, H-3_{Glc}), 5.73 (1 H, t, *J* = 9.8 Hz, H-4_{Glc}), 5.65 (1 H, d, *J* = 3.9 Hz, H-1_{Glc}), 5.52 (1 H, dd, *J* = 10.3, 3.9 Hz, H-2_{Glc}), 5.35 (1 H, d, *J* = 2.6 Hz, H-1_{Glc2D}), 4.85–4.74 (3 H, m, 3 x C*H*$_2$Ph), 4.63 (1 H, dd, *J* = 11.8, 2.7 Hz, H-6_{Glc}), 4.54–4.45 (3 H, m, H-5_{Glc}, H-6_{Glc}, C*H*$_2$Ph), 4.43 (1 H, d, *J* = 11.1 Hz, C*H*$_2$Ph), 4.26 (1 H, d, *J* = 12.2 Hz, C*H*$_2$Ph), 4.14 (1 H, ddd, *J* = 11.5, 8.2, 5.0 Hz, H-3_{Glc2D}), 3.67 (1 H, dt, *J* = 10.0, 2.1 Hz, H-5_{Glc2D}), 3.63 (1 H, dd, *J* = 9.9, 8.3 Hz, H-4_{Glc2D}), 3.19 (1 H, *J* = 10.8, 2.8 Hz, H-6_{Glc2D}), 3.11 (1 H, d, *J* = 10.8, 1.7 Hz, H-6_{Glc2D}), 2.31 (1 H, ddd, 13.1, 5.1, 1.0 Hz, H-$2_{Glc2D\ eq}$), 1.85 (1 H, ddd, 13.2, 11.16, 3.6 Hz, H-$2_{Glc2D\ ax}$); ^{13}C NMR (101 MHz, Chloroform-*d*) δ 166.1 (C=O), 165.8 (C=O), 165.4 (C=O), 165.3 (C=O), 138.8 (Ar C), 138.6 (Ar C), 137.9 (Ar C), 133.5 (Ar CH), 133.4 (Ar CH), 133.2 (Ar CH), 129.9 (Ar CH), 129.8 (Ar CH), 129.73 (Ar CH), 129.68 (Ar CH), 129.6 (Ar CH), 129.1 (Ar CH), 128.9 (Ar CH), 128.55 (Ar CH), 128.47 (Ar CH), 128.46 (Ar CH), 128.3 (Ar CH), 128.2 (Ar CH), 128.1 (Ar CH), 128.0 (Ar CH), 127.8 (Ar CH), 127.7 (Ar CH), 127.6 (Ar CH), 127.5 (Ar CH), 127.3 (Ar CH), 94.1 (C-1_{Glc2D}), 91.7 (C-1_{Glc}), 77.7 (C-4_{Glc2D}), 77.1 (C-3_{Glc2D}), 74.5 (C*H*$_2$Ar), 73.3 (C*H*$_2$Ar), 72.5 (C*H*$_2$Ar), 71.5 (C-5_{Glc2D}), 71.0 (C-2_{Glc}), 70.3 (C-3_{Glc}), 69.7 (C-4_{Glc}), 68.4 (C-5_{Glc}), 67.8 (C-6_{Glc2D}), 63.1 (C-6_{Glc}), 35.2 (C-2_{Glc2D}).

Synthesis of 6-azido and 6' azido 2-deoxy trehalose derivatives 21 and 25

2-Deoxy-α-D-glucopyranosyl 2',3',4',6'-tetra-*O*-benzoyl-α-D-glucopyranoside 18

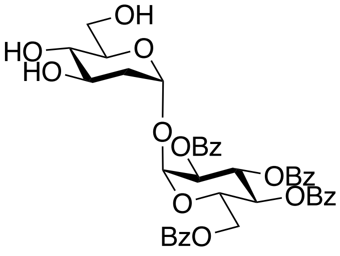

A solution of **17c** (380 mg, 0.375 mmol) was dissolved in MeOH/EtOAc (4 mL/2 mL). Pd/C (40 mg) was added and the reaction was placed under an atmosphere of H_2 (balloon). The reaction was stirred at room temperature for 16 h, filtered through Celite (washing MeOH/EtOAc) and concentrated *in vacuo*. Benzyl peaks were still visible in the crude NMR therefore the crude mixture was resubjected to identical reaction conditions. After stirring for a further 18h the reaction was filtered through Celite (washing MeOH/EtOAc) and concentrated *in vacuo* to give the title compound **18** as a white amorphous solid (265 mg, 0.357 mmol, 95%); R_f = 0.81 (CH_2Cl_2/MeOH 8:1); MS (ES^+) found *m/z* 760.3 $[M+ NH_4]^+$, HRMS (ESI) m/z: Calcd $C_{40}H_{42}O_{14}N$ $[M+ NH_4]^+$ 760.2600; Found 760.2588; ^{1}H NMR (400 MHz, Chloroform-*d*) δ 8.05–7.97 (6 H, m, Ar*H*), 7.92–7.90 (2 H, m, Ar*H*), 7.60–7.31 (12 H, m, Ar*H*), 6.21 (1 H, t, *J* = 10.0 Hz, H-3_{Glc}), 5.75 (1 H, t, *J* = 9.5 Hz, H-4_{Glc}), 5.66 (1 H, d, *J* = 3.8 Hz, H-1_{Glc}), 5.40 (1 H, dd, *J* = 10.3, 3.8 Hz, H-2_{Glc}), 5.30 (1 H, d, *J* = 3.1 Hz, H-1_{2DGlc}), 4.66–4.48 (3 H, m, H-5_{Glc}, 2 x H-6_{Glc}), 4.15 (1 H, ddd, *J* = 11.9, 8.1, 4.9 Hz, H-3_{2DGlc}), 3.51–3.42 (2 H, m, H-4_{2DGlc}, H-5_{2DGlc}), 3.31 (2 H, d, *J* = 3.2 Hz, 2 x H-6_{2DGlc}), 2.30 (1 H, ddd, *J* = 13.2, 5.0, 0.8 Hz, H-$2_{2DGlc\ eq}$), 1.80 (1 H, ddd, *J* = 13.2, 11.7, 3.7 Hz, H-$2_{2DGlc\ ax}$); ^{13}C NMR (101 MHz, Chloroform-*d*) δ 166.1 (C=O), 165.9 (C=O), 165.4 (C=O), 165.3 (C=O), 133.7 (Ar C), 133.5 (Ar C), 133.3 (Ar C), 133.2 (Ar C), 129.90 (Ar CH), 129.86 (Ar CH), 129.72 (Ar CH), 129.67 (Ar CH), 129.0 (Ar CH), 128.75 (Ar CH), 128.66 (Ar CH), 128.6 (Ar CH), 128.4 (Ar CH), 128.3 (Ar CH), 94.3 (C-1_{2DGlc}), 92.0 (C-1_{Glc}), 72.5 (C-4_{2DGlc}), 71.9 (C-5_{2DGlc}), 71.5 (C-2_{Glc}), 70.1 (C-3_{Glc}), 69.4 (C-4_{Glc}), 68.7 (C-3_{2DGlc}), 68.5 (C-5_{Glc}), 62.9 (C-6_{Glc}), 61.7 (C-6_{2DGlc}), 36.8 (C-2_{2DGlc}).

6-*O*-Tosyl-2-deoxy-α-D-glucopyranosyl 2',3',4',6'-tetra-*O*-benzoyl-α-D-glucopyranoside 19

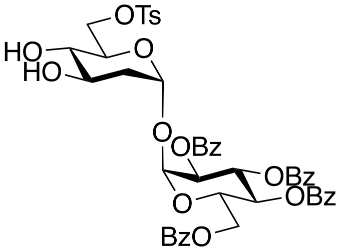

To a solution of **18** (220 mg, 0.296 mmol) in pyridine/DCM (0.6 mL/0.6 mL) was added TsCl (75 mg, 0.395 mmol). The reaction was stirred at Rt for 17h, sat. aq. $NaHCO_3$ was added and the reaction concentrated *in vacuo*. Purification by silica gel flash column chromatography (1:1:1 Hex/EA/CH_2Cl_2) gave the *title compound* **19** as a white amorphous solid (210 mg, 0.234 mmol, 79%); R_f = 0.28 (1:2 Hexane/EtOAc); ^{1}H NMR (400 MHz, Chloroform-*d*) δ 8.05–7.88 (8 H, m, Ar*H*), 7.69–7.66 (2 H, m, Ar*H*), 7.60–7.51 (3 H, m, Ar*H*), 7.48–7.30 (11 H, m, Ar*H*), 6.16 (1 H, t, *J* = 10.0 Hz, H-3_{Glc}), 5.73 (1 H, t, *J* = 10.0 Hz, H-4_{Glc}), 5.50 (1 H, d, *J* = 3.8 Hz, H-1_{Glc}), 5.37 (1 H, dd, *J* = 10.3, 3.8 Hz, H-2_{Glc}), 5.21 (1 H, d, *J* = 3.0 Hz, H-1_{2DGlc}), 4.63–4.59 (1 H, m, H-6_{Glc}), 4.51–4.46 (2 H, m, H-5_{Glc}, H-6_{Glc}), 4.19 –4.12 (1 H, m, H-3_{2DGlc}), 3.77 (1 H, m, *J* = 11.6, 2.8 Hz, H-6_{2DGlc}), 3.56 (1 H, dt, *J* = 9.7, 2.0 Hz, H-5_{2DGlc}), 3.49 (1 H, t, *J* = 9.3 Hz, H-4_{2DGlc}), 3.43 (1 H, *J* = 11.5, 1.7 Hz, H-6_{2DGlc}), 2.48 (3 H, s, ArC*H*$_3$), 2.29 (1 H, dd, *J* = 12.8, 5.5 Hz, H-$2_{2DGlc\ eq}$), 1.80 (1 H, ddd, *J* = 13.21 11.9, 3.6 Hz, H-$2_{2DGlc\ ax}$); ^{13}C NMR (101 MHz, Chloroform-*d*) δ 166.1 (C=O), 165.8 (C=O), 165.24 (C=O), 165.2 (C=O), 145.1 (Ar C), 133.7 (Ar C), 133.5 (Ar C), 133.3 (Ar C), 133.2 (Ar C), 129.9 (Ar CH), 129.8 (Ar CH), 129.71 (Ar CH), 129.66 (Ar CH), 129.5 (Ar CH), 129.0 (Ar CH), 128.72 (Ar CH), 128.66 (Ar CH), 128.4 (Ar CH), 128.4 (Ar CH), 128.3 (Ar CH), 127.9 (Ar CH), 94.5 (C-1_{2DGlc}), 92.2 (C-1_{Glc}), 71.3 (C-2_{Glc}), 71.0 (C-4_{2DGlc}), 70.6 (C-5_{2DGlc}), 70.1 (C-3_{Glc}), 69.3 (C-4_{Glc}), 68.5 (C-5_{Glc}), 68.2 (C-3_{2DGlc}), 67.7 (C-6_{2DGlc}), 62.9 (C-6_{Glc}), 36.5 (C-2_{2DGlc}), 21.7 (Ar*C*H$_3$).

N.B. Compound was not observed by mass spectrometry.

6-Deoxy-6-azido-2-deoxy-α-D-glucopyranosyl 2',3',4',6'-tetra-*O*-benzoyl-α-D-glucopyranoside 20

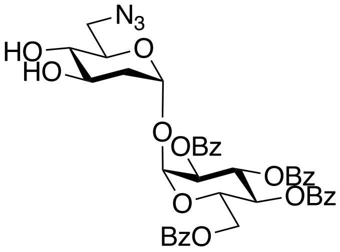

To a solution of **19** (85 mg, 0.095 mmol) in DMF (2 mL) was added NaN_3 (60 mg, 0.92 mmol). The reaction mixture was stirred for 18 h at room temperature then at 50 °C for 18 h. Sat. aq. $NaHCO_3$ was added, the product was extracted with EtOAc, dried and filtered to give a mixture of **19** and **20**. The crude mixture was resubjected to identical reaction conditions and stirred at 50 °C for 60 h. Sat. aq. $NaHCO_3$ was added, the product was extracted with EtOAc dried and filtered. The crude product was purified by flash column chromatography (1:1:1 Hexane/EtOAc/CH_2Cl_2) to give a mixture of the *title compound* **20** and DMF (2 eq.) (70 mg, calc. 0.77 mmol, 81%) which was used in the next step without further purification; R_f = 0.29 (1:2 Hexane/EtOAc); MS (ES^+) found *m/z* 790.3 $[M+ Na]^+$, HRMS (ESI) m/z: Calcd $C_{40}H_{37}O_{13}N_3Na$ $[M+Na]^+$ 790.2219; Found 790.2253; ^1H NMR (400 MHz, Chloroform-*d*) δ 8.05–7.96 (6 H*, m, Ar*H*), 7.90–7.88 (2 H, m, Ar*H*), 7.59–7.29 (12 H, m, Ar*H*), 6.19 (1 H, t, *J* = 10.0 Hz, H-3_{Glc}), 5.74 (1 H, t, *J* = 9.7 Hz, H-4_{Glc}), 5.65 (1 H, d, *J* = 3.8 Hz, H-1_{Glc}), 5.46 (1 H, dd, *J* = 10.3, 3.8 Hz, H-2_{Glc}), 5.32 (1 H, d, *J* = 2.9 Hz, H-1_{2DGlc}), 4.65–4.60 (1 H, m, H-6_{Glc}), 4.53–4.47 (2 H, m, H-5_{Glc}, H-6_{Glc}), 4.14 (1 H, ddd, *J* = 11.6, 8.9, 5.0 Hz, H-3_{2DGlc}), 3.63 (1 H, dt, *J* = 9.6, 3.7 Hz, H-5_{2DGlc}), 3.40 (1 H, t, *J* = 9.3 Hz, H-4_{2DGlc}), 2.99 (2 H, t, *J* = 3.3 Hz, 2 x H-6_{2DGlc}), 2.30 (1 H, ddd, *J* = 13.2, 5.0, 0.9 Hz, H-$2_{2DGlc\ eq}$), 1.83 (1 H, ddd, 13.2, 11.8, 3.6 Hz, H-$2_{2DGlc\ ax}$);^{13}C NMR (101 MHz, Chloroform-*d*) δ 166.1 (C=O), 165.9 (C=O), 165.4 (C=O), 165.3 (C=O), 133.6 (Ar C), 133.4 (Ar C), 133.3 (Ar C), 133.2 (Ar C), 129.9 (Ar CH), 129.8 (Ar CH), 129.69 (Ar CH), 129.68 (Ar CH), 129.5 (Ar CH), 129.0 (Ar CH), 128.8 (Ar CH), 128.60 (Ar CH), 128.58 (Ar CH), 128.4 (Ar CH), 128.3 (Ar CH), 94.2 (C-1_{2DGlc}), 92.2 (C-1_{Glc}), 72.5 (C-4_{2DGlc}), 71.7 (C-5_{2DGlc}), 71.2 (C-2_{Glc}), 70.2 (C-3_{Glc}), 69.5 (C-4_{Glc}), 68.8 (C-3_{2DGlc}), 68.5 (C-5_{Glc}), 62.9 (C-6_{Glc}), 50.6 (C-6_{2DGlc}), 36.9 (C-2_{2DGlc}), 21.7 (Ar*C*H_3).

*Peak overlayed with DMF signal.

6-Deoxy-6-azido-2-deoxy-α-D-glucopyranosyl α-D-glucopyranoside 21

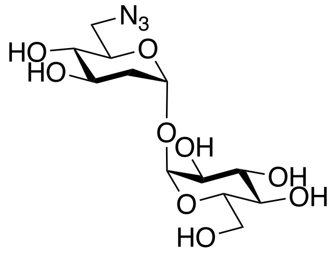

To a solution of **20** (40 mg, calc. 0.44 mmol contaminated with 2 eq. DMF) in THF (0.4 mL) was added H_2O (0.2 mL) and LiOH.H_2O (11 mg, 0.26 mmol). After stirring at RT for 22 h the reaction mixture was concentrated *in vacuo* and purified by silica gel flash column chromatography (CH_2Cl_2/MeOH 8:1 to remove BzOH then 3:1) to give the *title compound* **21** was a white amorphous solid (9 mg, 0.025 mmol, 57% over two steps; R_f = 0.41 (12:1 CH_2Cl_2/MeOH); MS (ES$^+$) found *m/z* 374.1 [M+ Na]$^+$, HRMS (ESI) m/z: Calcd $C_{12}H_{21}O_9N_3Na$ [M+Na]$^+$ 374.1170; Found 374.1173; ^{1}H NMR (400 MHz, MeOD-*d₄*) δ 5.30 (1 H, d, *J* = 2.6 Hz, H-1_{2DGlc}), 5.13 (1 H, d, *J* = 3.8 Hz, H-1_{Glc}), 4.00–3.94 (2 H, m, H-3_{2DGlc}, H-5_{2DGlc}), 3.83 (1 H, dd, *J* = 11.7, 2.2 Hz, H-6_{Glc}), 3.69 (1 H, dd, *J* = 9.8, 8.9 Hz, H-3_{Glc}), 3.67–3.58 (2 H, m, H-5_{Glc}, H-6_{Glc}), 3.54–3.40 (1 H, m, H-6_{2DGlc}), 3.49 (1 H, dd, *J* = 9.8, 3.8 Hz, H-2_{Glc}), 3.43 (1 H, dd, *J* = 13.1, 5.8 Hz, H-6_{2DGlc}), 3.32–3.30 (1 H, m, H-4_{Glc}), 3.27 (1 H, dd, *J* = 9.7, 9.1 Hz, H-4_{2DGlc}), 2.12 (1 H, ddd, *J* = 13.2, 5.2, 1.2 Hz, H-$2_{2DGlc\ eq}$), 1.72 (1 H, ddd, *J* = 13.2, 11.7, 3.7 Hz, H-$2_{2DGlc\ ax}$); ^{13}C NMR (101 MHz, MeOD-*d₄*) δ 93.9 (C-1_{Glc}), 92.3 (C-1_{2DGlc}), 73.4 (C-3_{Glc}), 72.9 (C-5_{Glc}), 72.6 (C-4_{2DGlc}), 72.0 (C-5_{2DGlc}), 71.6 (C-2_{Glc}), 70.5 (C-4_{Glc}), 67.9 (C-3_{2DGlc}), 61.3 (C-6_{Glc}), 51.4 (C-6_{2DGlc}), 37.1 (C-2_{2DGlc}).

3,4,6-Tri-*O*-benzyl-2-deoxy-α-D-glucopyranosyl α-D-glucopyranoside 22

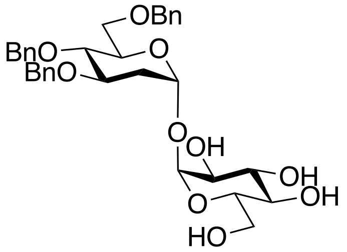

To a solution of **17c** (150 mg, 0.15 mmol) in MeOH/CH_2Cl_2 2.4 mL/0.6 mL) was added LiOH.H_2O (24 mg, 0.57 mmol). After stirring at RT for 2 h AcOH (40 µL, ~1 eq. w.r.t LiOH) was added and the reaction concentrated *in vacuo*. Purification by silica gel flash column chromatography (1:1 Hex/EtOAc to remove BzOH followed by 4:1 CH_2Cl_2/MeOH) gave the *title compound* **22** as a white amorphous solid (88 mg, 0.147 mmol, 98%); R_f = 0.77 (8:1 CH_2Cl_2/MeOH); MS (ES$^+$) found *m/z* 619.3 [M+Na]$^+$, HRMS (ESI) m/z: Calcd $C_{33}H_{40}O_{10}Na$ [M+Na]$^+$ 619.2514; Found 619.2515; ^{1}H NMR (400 MHz, Chloroform-*d*) δ 7.35–7.17 (15 H, m, Ar*H*), 5.22 (1 H, d, *J* = 1.9 Hz, H-1_{2DGlc}), 5.14 (1 H, d, *J* = 3.5 Hz, H-1_{Glc}), 4.89 (1 H, d, *J* = 10.9 Hz, C*H*$_2$Ph), 4.65–4.58 (3 H, m, 3 x C*H*$_2$Ph), 4.54 (1 H, d, *J* = 11.0 Hz, C*H*$_2$Ph), 4.46 (1 H, d, *J* = 12.2 Hz, C*H*$_2$Ph), 4.08 (1 H, ddd, *J* = 10.9, 8.9, 5.0 Hz, H-3_{2DGlc}), 4.01–3.98 (1 H, m, H-5_{2DGlc}), 3.82–3.50 (8 H, m, H-2_{Glc}, H-3_{Glc}, H-4_{Glc}, H-5_{Glc}, H-4_{2DGlc}, 2 x H-6_{Glc}, H-6_{2DGlc}), 3.72 (1 H, dd, *J* = 10.4, 3.4 Hz, H-6_{2DGlc}), 2.19 (1 H, dd, *J* = 12.4, 4.8, 0.3 Hz, H-$2_{2DGlc\ eq}$), 1.79–1.72 (1 H, m, H-$2_{2DGlc\ ax}$); ^{13}C NMR (101 MHz, Chloroform-*d*) δ 138.6 (Ar C), 138.1 (Ar C), 137.9 (Ar C), 128.42 (Ar CH), 128.39 (Ar CH), 128.3 (Ar CH), 128.2 (Ar CH), 128.0 (Ar CH), 127.8 (Ar CH), 127.7 (Ar CH), 127.6 (Ar CH), 94.1 (C-1_{Glc}), 93.0 (C-1_{2DGlc}), 78.1 (CH), 77.1 (C-3_{2DGlc}), 75.2 (*C*H$_2$Ph), 73.9 (CH), 73.4 (*C*H$_2$Ph), 72.0 (CH), 71.8 (*C*H$_2$Ph), 71.5 (CH), 71.2 (C-5_{2DGlc}), 69.4 (CH), 68.8 (C-6_{2DGlc}), 61.0 (C-6_{Glc}), 35.1 (C-2_{2DGlc}).

3,4,6-Tri-*O*-benzyl-2-deoxy-α-D-glucopyranosyl 6-*O*-tosyl-α-D-glucopyranoside 23

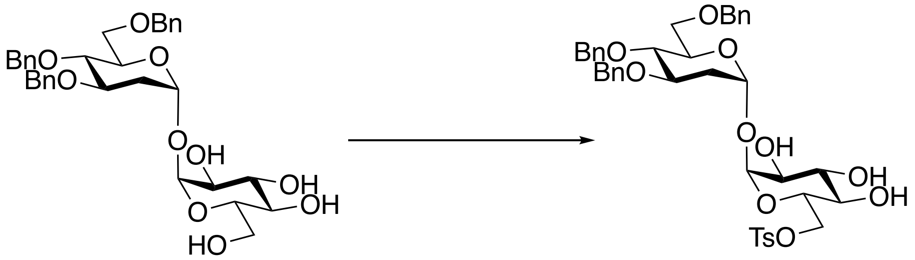

To a solution of **22** (170 mg, 0.29 mmol) in a mixture of pyridine and CH_2Cl_2 (1.2 ml, 1:1 *v:v*) was added TsCl (60 mg, 0.32 mmol). The reaction was stirred at RT for 18h at which point TLC analysis indicated no starting material was remaining. The reaction mxiture was concentrated *in vacuo,* and the reaction mixture purified by flash column chromatography (2:1:1 CH_2Cl_2/Ethyl acetate/Hexane to 1:1 CH_2Cl_2/Ethyl acetate) to give the *title compound* **23** as a white amorphous solid (45 mg, 0.06 mmol, 21%); R_f= 0.26 (3:7 Hexane/EtOAc); ^{1}H NMR (400 MHz, Chloroform-*d*) δ 7.93 (2 H, m, Ar*H*), 7.36–7.28 (15 H, m, Ar*H*),7.21–7.18 (2 H, m, Ar*H*), 5.15 (1 H, d, *J* = 2.4 Hz, H-1_{2DGlc}), 5.04 (1 H, d, *J* = 3.8 Hz, H-1_{Glc}), 4.87 (1 H, d, *J* = 10.8 Hz, C*H*$_2$Ph), 4.67–4.60 (3 H, m, 3 x C*H*$_2$Ph), 4.54 (1 H, d, *J* = 10.8 Hz, C*H*$_2$Ph), 4.50 (1 H, d, *J* = 12.1 Hz, C*H*$_2$Ph), 4.26 (1 H, dd, *J* = 11.0, 4.9 Hz, H-6_{Glc}), 4.18 (1 H, d, *J* = 11.0 Hz, 2.0 Hz, H-6_{Glc}), 4.00 (1 H, ddd, *J* = 11.3, 8.7, 5.0 Hz, H-3_{2DGlc}), 3.93 (1 H, ddd, *J* = 9.8, 3.9, 2.0 Hz, H-5_{2DGlc}), 3.75–3.65 (4 H, m, H-3_{Glc}, H-5_{Glc}, 2 x H-6_{2DGlc}), 3.61 (1 H, dd, *J* = 9.7, 9.0 Hz, H-4_{2DGlc}), 3.44 (1 H, dd, *J* = 9.7, 3.8 Hz, H-2_{Glc}), 3.37 (1 H, t, *J* = 9.5 Hz, H-4_{Glc}), 3.44 (3 H, s, ArC*H*$_3$), 2.45 (3H, s, C*H*$_3$), 2.16 (1 H, ddd, *J* = 13.2, 5.0, 1.2 Hz, H-$2_{2DGlc\ eq}$), 1.76 (1 H, ddd, *J* = 13.2, 11.4, 3.6 Hz, H-$2_{2DGlc\ ax}$); ^{13}C NMR (101 MHz, Chloroform-*d*) δ 145.0 (Ar C), 138.5 (Ar C), 138.0 (Ar C), 137.9 (Ar C), 132.8 (Ar C), 129.8 (Ar CH), 128.43 (Ar CH), 128.41 (Ar CH), 128.37 (Ar CH), 128.2 (Ar CH), 127.94 (Ar CH), 127.86 (Ar CH), 127.77 (Ar CH), 127.75 (Ar CH), 127.7 (Ar CH), 94.1 (C-1_{Glc}), 93.4 (C-1_{2DGlc}), 78.0 (C-4_{2DGlc}), 76.9 (C-3_{2DGlc}), 75.1 (*C*H$_2$Ph), 73.9 (CH), 73.5 (*C*H$_2$Ph), 71.8 (*C*H$_2$Ph), 71.6 (C-2_{Glc}), 71.3 (C-5_{2DGlc}), 70.0 (CH), 69.6 (C-4_{Glc}), 68.89 (C-6), 68.88 (C-6), 35.0 (C-2_{2DGlc}), 21.6 (Ar*C*H$_3$).

N.B. Compound was not observed by mass spectrometry.

2-Deoxy-α-D-glucopyranosyl 6-deoxy-6-azido-α-D-glucopyranoside 25

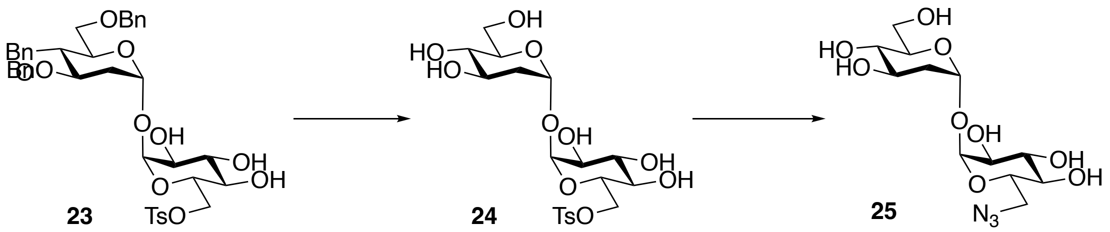

23 **24** **25**

To a solution of **23** (22 mg, 0.030 mmol) in MeOH/EtOAc (0.3 ml, 2:1) was added Pd/C (5 mg). The reaction was stirred under H_2 atmosphere for 18 h, filtered through celite (washing MeOH) and concentrated. NMR indicated the removal of benzyl peaks but the tosyl group was still present and crude **24** was used directly in the next step. Intermediate **24** was dissolved in DMF (0.5 mL) and sodium azide (19 mg, 0.29 mmol) was added. The reaction mixture was heated to 70 °C for 40 h, cooled to RT, diluted with H_2O and sat. aq. $NaHCO_3$. The reaction mixture was concentrated and purified by passage through a short plug of silica (3:1 EtOAc/MeOH0 then via size exclusion chromatography (Sephadex LH-20, 5:1 CH_2Cl_2/MeOH) to give the *title compound* **25** was a white amorphous solid (5 mg, 48% over 2 steps).

Data for **25**: R_f = 0.32 (12:1 CH_2Cl_2/MeOH); MS (ES^+) found *m/z* 374.1 $[M+Na]^+$, *m/z* HRMS (ESI) m/z: Calcd $C_{12}H_{21}O_9N_3Na$ $[M+Na]^+$ 374.1170; Found 374.1178; ^1H NMR (400 MHz, Chloroform-*d*) δ 5.27 (1 H, d, *J* = 2.5 Hz, H-1_{2DGlc}), 5.15 (1 H, d, *J* = 3.8 Hz, H-1_{Glc}), 3.99 (1 H, ddd, *J* = 11.6, 9.0, 5.1 Hz, H-3_{2DGlc}), 3.85–3.69 (4 H, m, H-5_{Glc}, H-5_{2DGlc}, 2 x H-6_{Glc}), 3.69 (1 H, dd, *J* = 9.7, 8.8 Hz, C-3_{Glc}), 3.55–3.48 (2 H, m, H-2_{Glc}, H-6_{2DGlc}), 3.39 (1 H, dd, *J* = 13.2, 6.5 Hz, H-6_{2DGlc}), 3.33–3.27 (2 H, m, H-4_{Glc}, H-4_{2DGlc}), 2.11 (1 H, ddd, *J* = 13.1, 5.1, 1.2 Hz, H-$2_{2DGlc\ eq}$), 1.72 (1 H, ddd, *J* = 13.1, 11.7, 3.7 Hz, H-$2_{2DGlc\ ax}$); ^{13}C NMR (101 MHz, Chloroform-*d*) δ 93.7 (C-1_{Glc}), 92.6 (C-1_{2DGlc}), 73.1 (C-3_{Glc}), 72.9 (C-5_{2DGlc}), 71.9 (C-5_{Glc}), 71.8 (C-4_{Glc} or C-4_{2DGlc}), 71.6 (C-2_{Glc}), 71.3 (C-4_{Glc} or C-4_{2DGlc}), 68.1 (C-3_{2DGlc}), 61.3 (C-6_{2DGlc}), 51.3 (C-6_{Glc}), 37.0 (C-2_{2DGlc}).

Crude ^1H NMR data for **24**: ^1H NMR (400 MHz, Chloroform-*d*) δ 7.81–7.79 (2 H, m, Ar*H*), 7.47–7.51 (2 H, m, Ar*H*), 5.11 (1 H, d, *J* = 2.9 Hz, H-1_{2DGlc}), 5.02 (1 H, d, *J* = 3.9 Hz, H-1_{Glc}), 4.34 (1 H, dd, *J* 10.9, 1.9 Hz, H-6_{Glc}), 4.17 (1 H, dd, *J* = 10.8, 6.2 Hz, H-6_{Glc}), 3.98–3.91 (1 H, m, H-3_{2DGlc}), 3.82 (1 H, dd, *J* = 11.2, 1.9 Hz, H-6_{2DGlc}), 3.76–3.70 (3 H, m, H-5_{Glc}, H-5_{2DGlc}, H-6_{2DGlc}), 3.63 (1 H, dd, *J* = 9.8, 8.8 Hz, H-3_{Glc}), 4.00 (1 H, dd, *J* = 9.8, 3.7 Hz, H-2_{Glc}), 3.28 (1 H, t, *J* = 9.3 Hz, H-4_{2DGlc}), 3.23 (1 H, dd, *J* = 10.2, 8.8 Hz, H-4_{Glc}), 3.23 (3 H, s, ArC*H*3), 2.03 (1 H, ddd, *J* = 13.1, 5.2, 1.3 Hz, H-$2_{2DGlc\ eq}$), 1.66 (1 H, ddd, *J* = 13.2, 11.7, 3.6 Hz, H-$2_{2DGlc\ ax}$).

Control Reactions and Mechanistic Studies

$AuCl_3$ catalysed anomerisation of 15b

In order to investigate the mechanism of the reaction an anomeric mixture of 2-deoxy trehalose **15b** was synthesised and subjected to the reaction conditions (2.5 mol% $AuCl_3$ in toluene at 50 °C). Samples were taken from the reaction mixture at 1 h, 4 h and 22 h and ^{1}H NMR spectra taken. The amount of **α,α'-15b** w.r.t **α,β'-15b** di not increase, in fact the opposite occurred with the amount of **α,β'-15b** increasing w.r.t **α,α'-15b** (Table S5) along with cleavage of the anomeric bond as evidenced by the appearance of hemiacetal **13a.**

Table S5 – Results of anomerisation of 15b

Time (h)	α,α'-**15b***	α,β'-**15b***	**13a***
0	4	1	0
1	1.2	1	0.6
4	1.3	1	0.7
22	1.3	1	0.7

*Calculated from crude ^{1}H NMR spectra

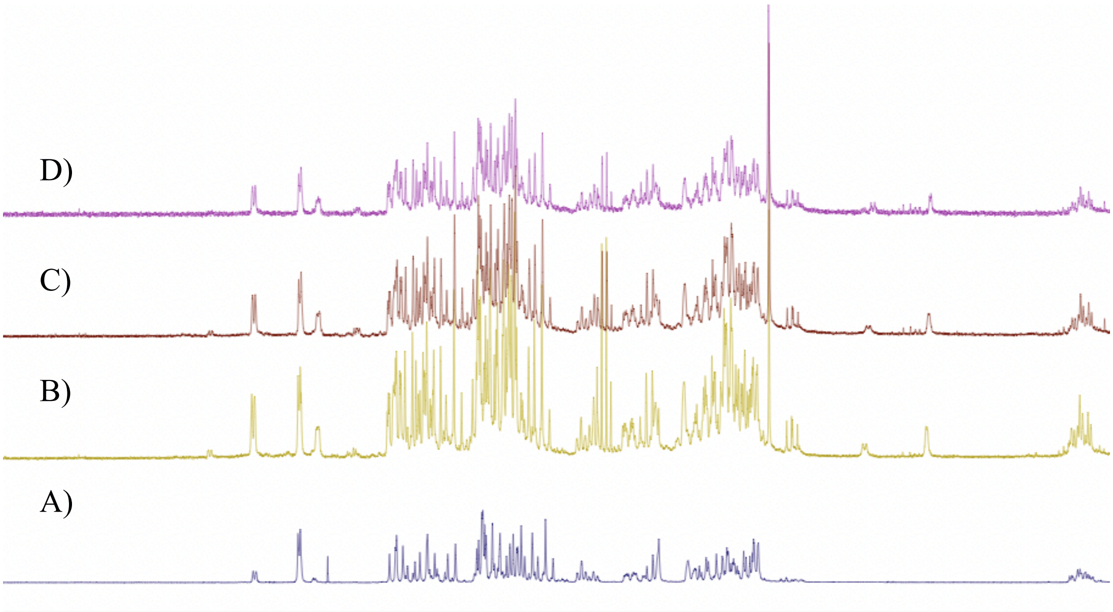

Figure S1 - ^{1}H NMR spectra in d_8-toluene of A) **α,α'-15b** and **α,β'-15b** B) after treatment with 2.5 mol% $AuCl_3$ at 50 °C after 1 hour C) after treatment with 2.5 mol% $AuCl_3$ at 50 °C after 4 hours, D) after treatment with 2.5 mol% $AuCl_3$ at 50 °C after 22 hours.

Synthesis of 3,4,6-Tri-*O*-benzyl-2-deoxy-α-D-galactopyranosyl 2',3',4',6'-tetra-*O*-benzyl-α/β-D-glucopyranoside 15b

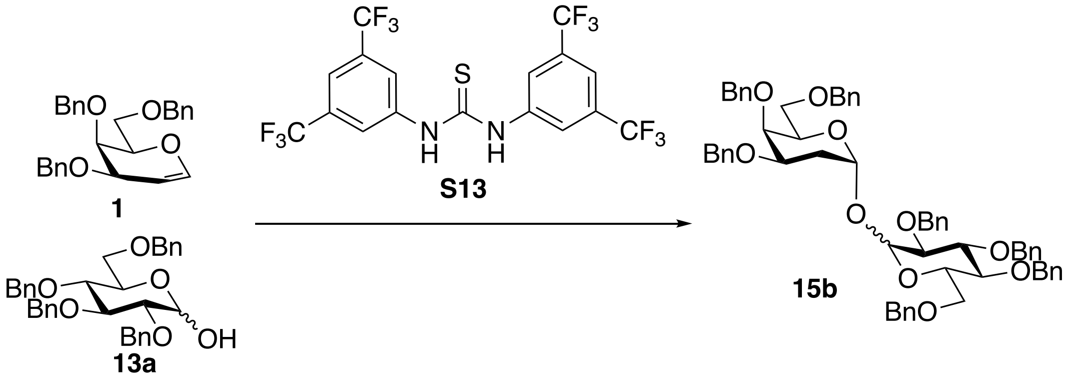

Synthesised according to a procedure modified from the method reported by *Bradshaw et al.*[26]

A solution of **13a** (292 mg, 0.54 mmol) and Schreiner's Thiourea Catalyst **S13** (2.5 mg, 0.005 mmol) in anhydrous CH_2Cl_2 (1.5 mL) was added to tri-benzylated galactal **1** (201 mg, 0.45 mmol). The reaction was refluxed for 4 h, concentrated *in vacuo* and purified by flash column chromatography (6:1 Hexane/EtOAc) to give **α,α'-14c** (188 mg, 0.20 mmol, 44%), a mixture of **α,α'-15b** and **α,β'-15b** (135 mg, 0.14, 31%, α,α'/α,β' = 4:1) and **α,β'-15b** (13 mg, 0.004 mmol, 3%).

Data for **α,α'-15b** and **α,β'-15b** in agreement with previous synthesis.[26]

Experimental procedure for attempted $AuCl_3$ catalysed anomerisation

A mixture of **α,α'-14c** and **α,β'-15b** (38 mg, 0.04 mmol, α,α'/α,β' = 4:1) was dissolved in toluene (0.2 mL) and $AuCl_3$ (0.3 mg, 0.001 mmol) was added. The reaction was heated to 50 °C and samples taken after 1h, 4 h and 22h. The samples were quenched with sat. aq. $NaHCO_3$ and sat. aq. NaS_2O_3, extracted with EtOAc, dried and concentrated.

NMR experiments

In order to investigate if the unreactive hemiacetal acceptors interacted with $AuCl_3$ hemiacetal, **13c** was mixed with equimolar amounts of $AuCl_3$ in d_8-toluene. No interaction was observed by ^{1}H NMR spectroscopy at RT of 50 °C

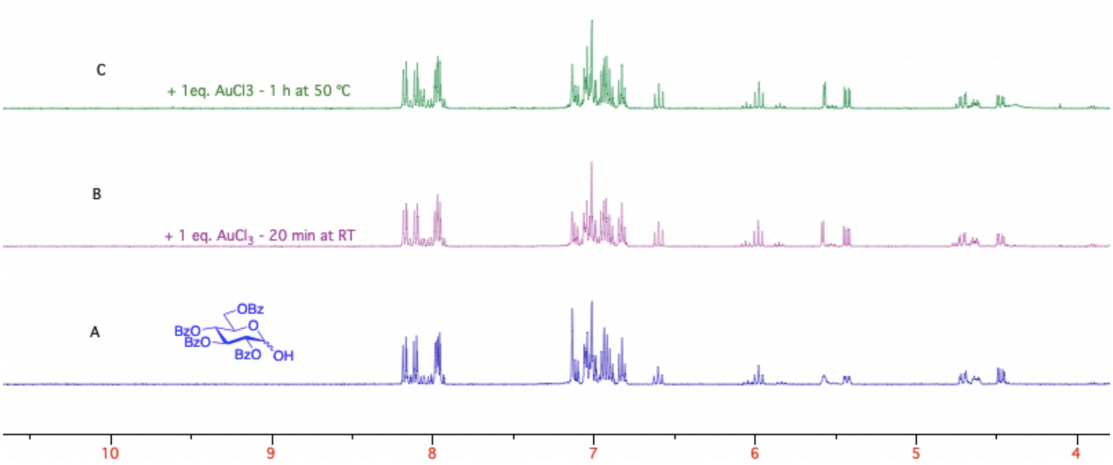

Figure S2 - ^{1}H NMR spectra in d8-toluene of A) Hemiacetal **13c**, B) Hemiacetal **13c** plus 1 eq. $AuCl_3$ after 20 min at RT, C) Hemiacetal **13c** plus 1 eq. $AuCl_3$ after 1 hour at 50 °C.

Control reactions using base

Dimerisation of 2-deoxy donor **3a** was not observed to occur in the presence of DIPEA. No reaction was observed between 2-deoxy donor **3a** and acceptor **4a** in the presence of K_2CO_3.

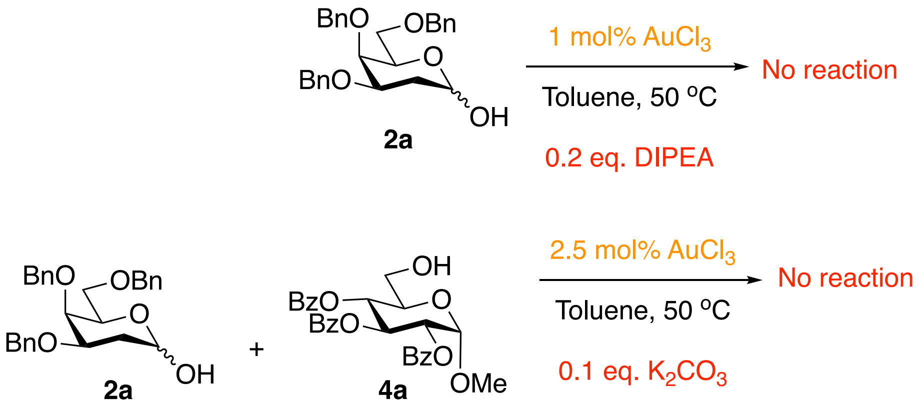

Scheme S1. **Attempted $AuCl_3$ catalysed reactions of 2a in the presence of organic or inorganic bases**

Synthesis of 2-deoxy trehalose derivatives using other acid-based activating promoters.

To probe he mechanism of the reaction dimerisation of 2-deoxy hemiacetal donor **2a** was tested using HCl. Dimerisation was shown to occur although in lower yields than when $AuCl_3$ was used (Table S6). However, formation of unsymmetrical trehalose derivative **15e** using benzoylated hemiacetal **2a** and acceptor **13c** was not observed using HCl as the catalyst (Scheme S2). A number of different acidic activation conditions were also tested for this reaction (**2a** + **13c**) but in all cases lower yields and/or less clean reaction profiles and the formation of by-products were observed compared to the use of $AuCl_3$ (Table S7).

Table S6 – Synthesis of Dimer 10 using HCl

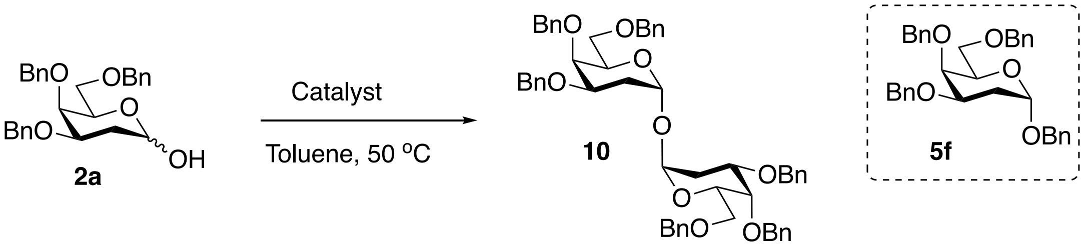

Entry	Conditions	Time (h)	Crude yield*	**2a** : **10** : **5f** : unknown
1	1 mol% $AuCl_3$	4	87% (55% isolated)	4 : 87 : 2 : 6
2	5 mol% HCl**	1	56%	22 : 56 : 0 : 22
3	5 mol% HCl** (at RT)	2	56%	18 : 56 : 0 : 25
4	5 mol% HCl**	3***	79%	8 : 79 : 5 : 8
5	5 mol% HCl**	20***	51%	5 : 51 : 44 : 0

*Estimated from crude ^{1}H NMR spectrum
**4 M HCl in dioxane used
***First 2 h at RT

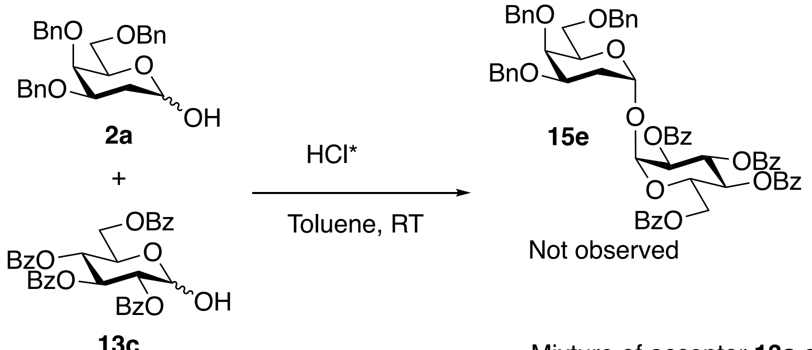

Mixture of acceptor **13a** and unknown impurity observed in crude ^{1}H NMR spectrum

*Solution of 2 M HCl in Et_2O diluted in toluene

0.2 eq, 1 eq, 2 eq tested

Scheme S2 – Attempted formation of asymmetric trehalose **15e** using HCl

Table S7 – Asymmetric trehalose formation using alternative activation systems

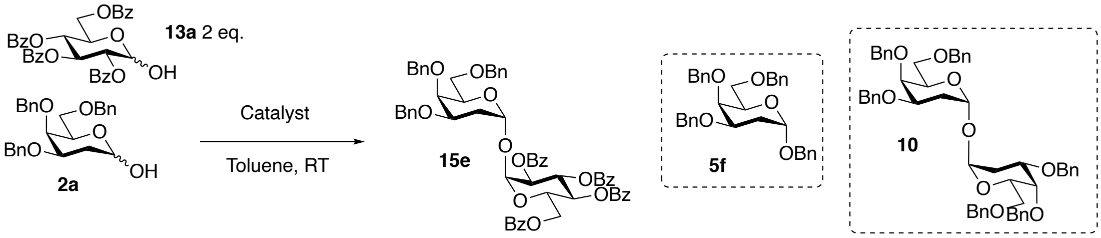

Entry	Conditions	Time (h)	Yield of **15e**	Impurities formed
1	0.2 eq. $AuCl_3$	0.5	8%	
2	0.2 eq. $AuCl_3$	2	14%	
3	0.2 eq. $AuCl_3$	7	27%	12% **10**
4	1 eq. $AuCl_3$	0.5	33%	
5	1 eq. $AuCl_3$	2	17%	
6	1 eq. $AuCl_3$	7	Trace	
7	1 eq. TfOH	0.5, 2, 7	0%	Donor consumed, only acceptor remaining
8	0.2 eq.TMSOTf	0.5	13%	13% **10**, ~15% unidentified impurity
9	0.2 eq.TMSOTf	2	18%	20% **10,** ~20% unidentified impurity
10	0.2 eq.TMSOTf	7	25%	15% **10,** 10 % **5f**, ~25% unidentified impurity
11	1.1 eq. TMSOTf	0.5, 2, 7	Trace	
12	2.2 eq. TMSOTf	0.5, 2, 7	Trace	
13	2 eq. BuBOTf	0.5	25%	~25% unidentified impurity
14	2 eq. BuBOTf	2	18%	~20% unidentified impurity
15	2 eq. BuBOTf	7	9%	~10% unidentified impurity
16	Amberlite IR120 H+	0.5, 2, 7	No reaction	

References

(1) Still, W. C.; Kahn, M.; Mitra, A. Rapid chromatographic technique for preparative separations with moderate resolution *J. Org. Chem.*, **1978**, *43*, 2923–2925.
(2) Barnes, N. J.; Probert, M. A.; Wightman, R. H. Synthesis of 2-deoxy-α- and -β-D-arabino-hexopyranosyl phosphonic acids and related compounds; analogues of early intermediates in the shikimate pathway. *J. Chem. Soc., Perkin Trans. 1.* **1996***, 5,* 431–438.
(3) Durantie, E.; Bucher, C.; Gilmour, R. Fluorine-Directed β-Galactosylation: Chemical Glycosylation Development by Molecular Editing *Chem. Eur. J.* **2012**, *18*, 8208–8215.
(4) Wagner, S.; Mersch, C.; and Hoffmann-Röder, A. Fluorinated Glycosyl Amino Acids for Mucin-Like Glycopeptide Antigen Analogues, *Chem. Eur. J.* **2010**, *16*, 7319–7330.
(5) Balmond, E. I.; Coe, D. M.; Galan, C. M.; McGarrigle, E. M. α-Selective Organocatalytic Synthesis of 2-Deoxygalactosides, *Angew. Chem. Int. Ed.,* **2012**, *51*, 9152–9155.
(6) Bucher, C.; Gilmour, R. Fluorine-Directed Glycosylation. *Angew. Chem., Int. Ed.* **2010**, *49***,** 8724–8728.
(7) Lecourt, T.; Herault, A.; Pearce, A. J.; Soologoub, M.; Sinaÿ, P. Triisobutylaluminium and Diisobutylaluminium Hydride as Molecular Scalpels: The Regioselective Stripping of Perbenzylated Sugars and Cyclodextrins *Chem. Eur. J.* **2004**, *10*, 2960–2971.
(8) Varga-Berenguel, A. V.; Meldal, M.; Paulsen, H.; Jensen, K. J.; Bock, K. Synthesis of Glycosyltyrosine Building Blocks for Sol id - Phase G lycopeptide Assembly: Use of Aryl tert-Butyl Ethers as Glycosyl Acceptors in Aromatic Glycosylations. *J. Chem. Soc. Perkin. Trans. 1.,* **1994,** 3287–3294.
(9) Zhang, Z.; Ollmann, I. R.; Ye, X-S.; Wischnat, R.; Baasov, T.; Wong, C-H. Programmable One-Pot Oligosaccharide Synthesis *J. Am. Chem. Soc.* **1999**, *121*, 734-753
(10) Fu, J., Laval, S. & Yu, B. Total Synthesis of Nucleoside Antibiotics Plicacetin and Streptcytosine A. *J. Org. Chem.* **83**, 7076–7084 (2018).
(11) Sau, A.; Williams, R.; Palo-Nieto, C.; Franconetti, A.; Medina, S.; Galan, M. C. Palladium-Catalysed Direct Stereoselective Synthesis of Deoxyglycosides from Glycals. *Angew. Chem. Int. Ed*. **2017**, *56*, 3640–3644.
(12) Sau, A.; Galan. M. C. Palladium-Catalyzed α-Stereoselective *O*-Glycosylation of O(3)-Acylated Glycals Org. Lett. 2017, 19, 2857–2860.
(13) Sau, A.; Palo-Nieto, C.; Galan, M. C. Substrate-controlled direct stereoselective synthesis of deoxyglycosides from glycals. *J. Org. Chem.* **2019**, *84*, 2415–2424.
(14) Palo-Nieto, C.; Sau, A.; Jeanneret, R. A.; Payard, P.-A.; Braga Martins-Teixeira, M.; Carvalho, I.; Grimaud, L.; Galan, M. C. Copper Reactivity Can be Tuned to Catalyse the Stereoselective Synthesis of 2-Deoxy Glycosides from Glycals *Org. Lett.*, **2020**, *22*, 1991–1996.
(15) B. D. Sherry, R. N. Loy, F. D. Toste, Rhenium(V)-Catalyzed Synthesis of 2-Deoxy α-glycosides, *Journal of the American Chemical Society*, **2004**, *126*, 4510–4511.
(16) Manhas, S.; Taylor, M. S. Taylor, Dehydrative glycosidations of 2-deoxysugar derivatives catalyzed by an arylboronic ester. *Carbohydr. Res.,* **2018***, 470,* 42–49.
(17) Cue, X-H.; Zhong, M.; Meng, X-B.; Li, Z-J. The synthesis of α-2-deoxyglycosides from glycals using TMSI-PPh_3. *Carbohydr. Res.,* **2012***, 1,* 19–22.
(18) Bai, J.; Li, B.; Li, T.; Liu, K-M.; Liu, M.; Qin, X.; Xiong, D.-C.; Ye, X-S.; Zhang, H.; Stereoselective Electro-2-deoxyglycosylation from Glycals. *Angew. Chem. Int. Ed.* **2020**, *59*, 15204–15208.
(19) Hsu, M-Y.; Lio, Y-P.; Lam, S.; Lin, S-C.; Wan, C-C. TMSBr-mediated solvent- and work-up-free synthesis of α-2-deoxyglycosides from glycals, *Beilstein J. Org. Chem.* **2016**, *12*, 1758–1764.
(20) Morris, W. J.; Shair, M. D. Stereoselective Synthesis of 2-Deoxy--glycosides Using Anomeric O-Alkylation/Arylation. *Org. Lett.,* **2009**, *11*, 9–12.

(21) Ghosh, T.; Mukherji, A.; Srivastava, H. K.; Kancharla, P. K. Secondary amine salt catalyzed controlled activation of 2-deoxy sugar lactols towards alpha-selective dehydrative glycosylation *Org. Biomol. Chem.*, **2018**, *16*, 2870–2875.
(22) Nogueira, J. M.; Nguyen, S. H.; Bennett, C. S. Cyclopropenium Cation Promoted Dehydrative Glycosylations Using 2-Deoxy- and 2,6-Dideoxy-Sugar Donors
Org. Lett., **2011***, 13,* 2814–2817.
(23) Kim, K. S.; Lee, Y. J.; Kim, H. Y.; Kang, S. S.; Kwon, S. Y. Glycosylation with glycosyl benzyl phthalates as a new type of glycosyl donor. *Org. Biomol. Chem.,* **2004**, *2*, 2408–2410.
(24) Komba, S.; Ishida, H.; Kiso, M.; Hasegawa, A. Synthesis and biological activities of three sulfated sialyl Le^x ganglioside analogues for clarifying the real carbohydrate ligand structure of L-selectin, *Bioorg. Med. Chem.,* **1996**, *4*, 1833–1847.
(25) Wiebe, C.; Schlemmer, C.; Weck, S.; Opatz, T. Sweet (hetero)aromatics: glycosylated templates for the construction of saccharide mimetics *Chem. Commun.,* **2011**, *47*, 9212–9214.
(26) Bradshaw, G. A.; Colgan, A. C.; Allen, N. P.; Pongener, I.; Boland, M. B.; McGarrigle, E. M. Stereoselective organocatalyzed glycosylations – thiouracil, thioureas and monothiophthalimide act as Brønsted acid catalysts at low loadings. *Chem. Sci.,* **2019**, *10*, 508–514.
(27) Kelson, I. K.; Fet, B-A. Reactions of Nucleophiles with Reactive Intermediates in the 3,4,6-Tri-*O*-benzyl-d-glucal–TfOH–*n*-Bu_4NI Reaction System. *J. Carbohydr. Chem.* **2003**, *22,* 827–841.
(28) Hadd, M.; Gervay, J. Glycosyl iodides are highly efficient donors under neutral conditions. *Carbohyr. Res.,* **1999**, *320*, 61–69.
(29) Cumpstey, I.; Frigell, J.; Pershagen, E.; Aktar, T.; Moreno-Calavijo, E.; Robina, I.; Alonzi, D. S.; Butter, T. D. Amine-linked diglycosides: Synthesis facilitated by the enhanced reactivity of allylic electrophiles, and glycosidase inhibition assays. *Beilstein J. Org. Chem.* **2011,** *7,* 1115–1123.
(30) Beaver, M.; Woerpol, K. A.; Erosion of Stereochemical Control with Increasing Nucleophilicity: *O*-Glycosylation at the Diffusion Limit. *J. Org. Chem.* **2010,** *75***,** 1107–1118.
(31) Rintelmann, C. L.; Grinnagq-Pulley, T.; Ross, K.; Kabotso, S. E> K.; Toepp, A.; Cowell, A.; Peterson, C.; Narasimham, B.; Pohl, N. Design and Synthesis of Multivalent a-1,2-Trimannose-Linked Bioerodible Microparticles for Applications in Immune Response Studies of Leishmania Major Infection, *Beilstein, J. Org. Chem.* **2019**, 15, 623–632.
(32) Andersen, S. M. et al. 3-(Dimethylamino)-1-propylamine: A Cheap and Versatile Reagent for Removal of Byproducts in Carbohydrate Chemistry, *Org. Lett.* **2015**, 17, 944–947.
(33) Li, X-D.; Kang, S-T.; Li, G-Y.; Li, X.; Wang, J-H. Synthesis of Some Phenylpropanoid Glycosides (PPGs) and their Acetylcholinesterase/Xanthine Oxidase Inhibitory Activities *Molecules,* **2011**, *16*, 3580–3596.

NMR spectra

^{1}H Spectrum of 2a (400 MHz, Chloroform-*d*)

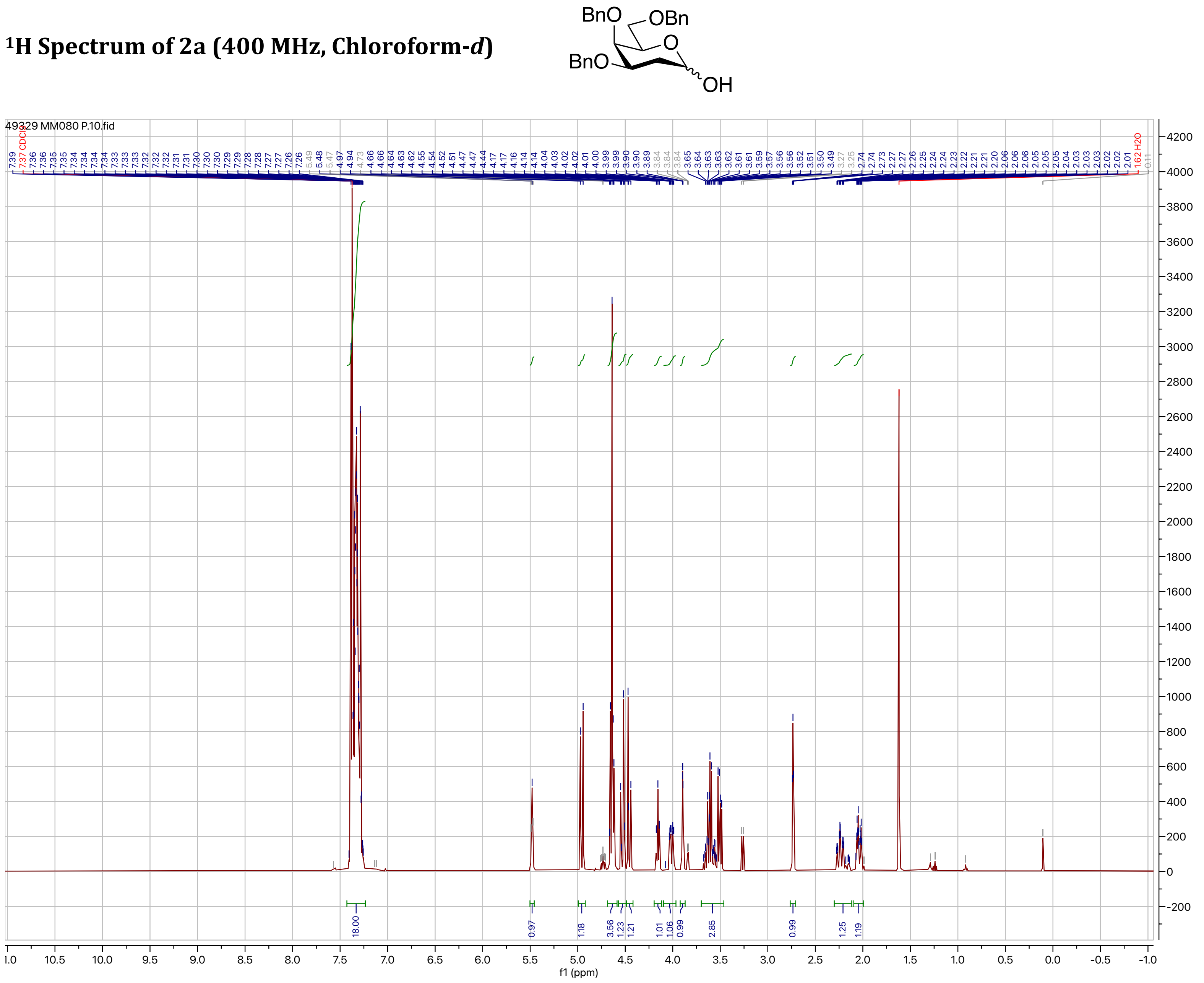

^{13}C Spectrum of 2a (101 MHz, Chloroform-*d*)

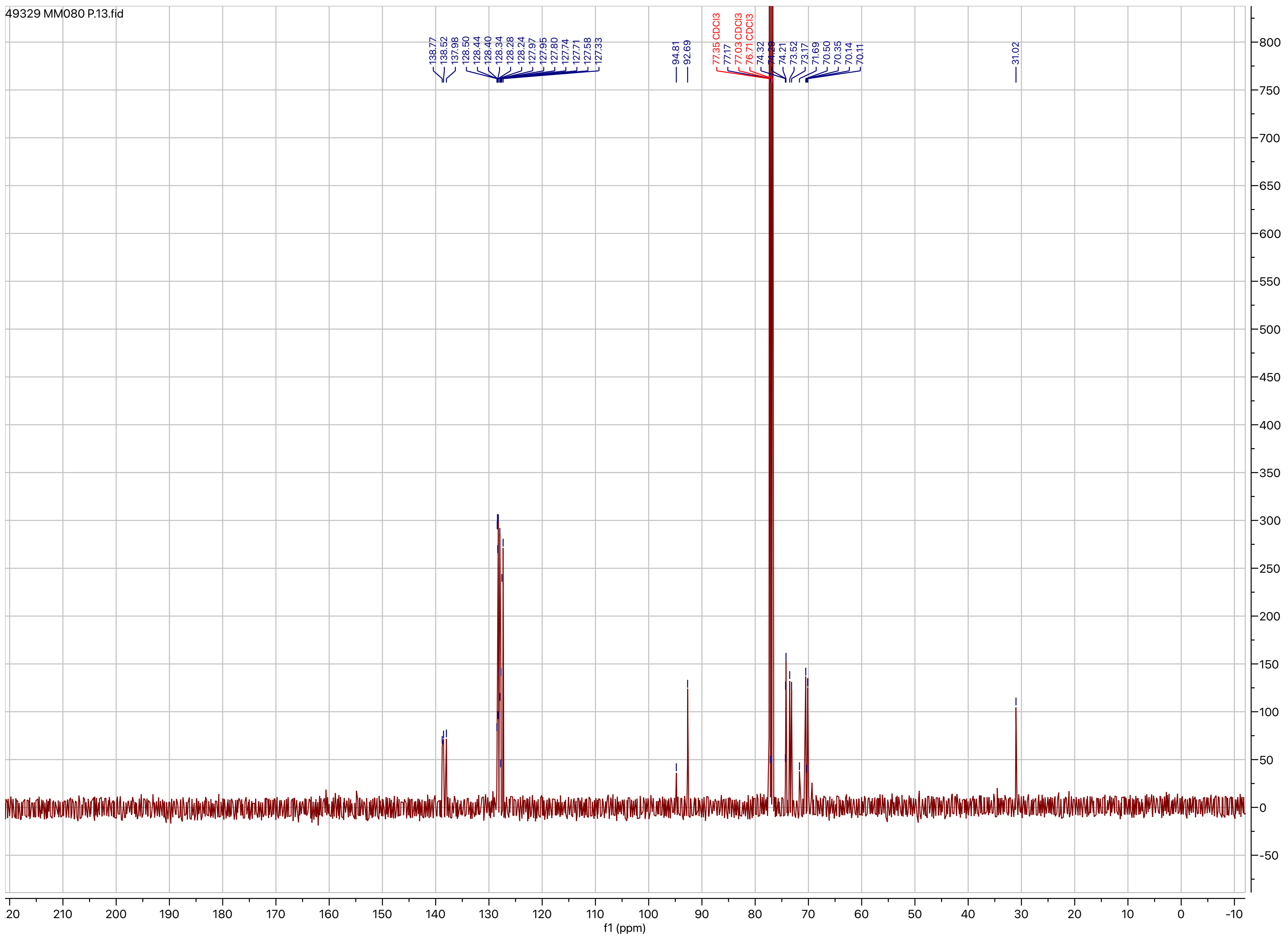

^{1}H Spectrum of 2b (400 MHz, Chloroform-*d*)

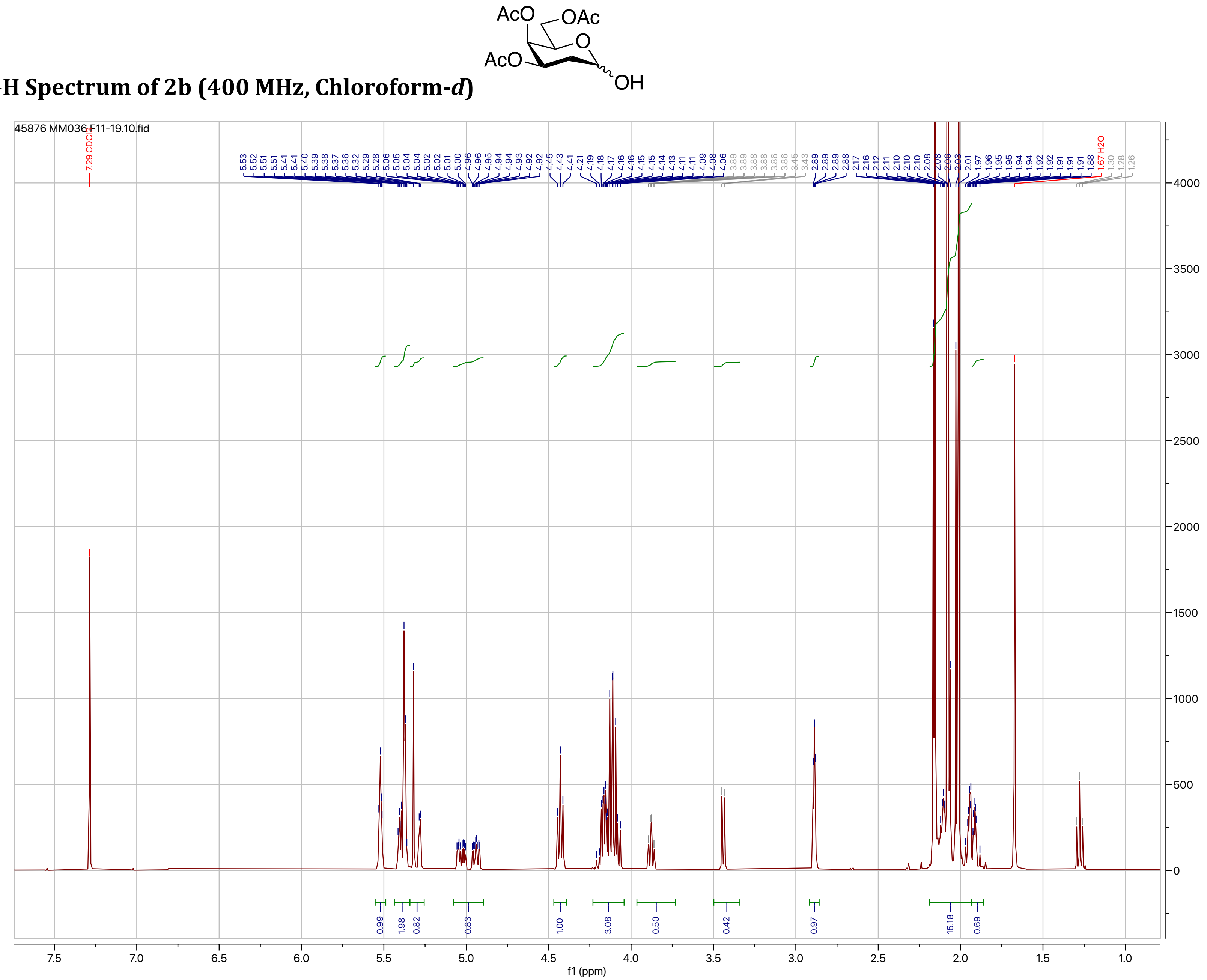

^{13}C Spectrum of 2b (101 MHz, Chloroform-*d*)

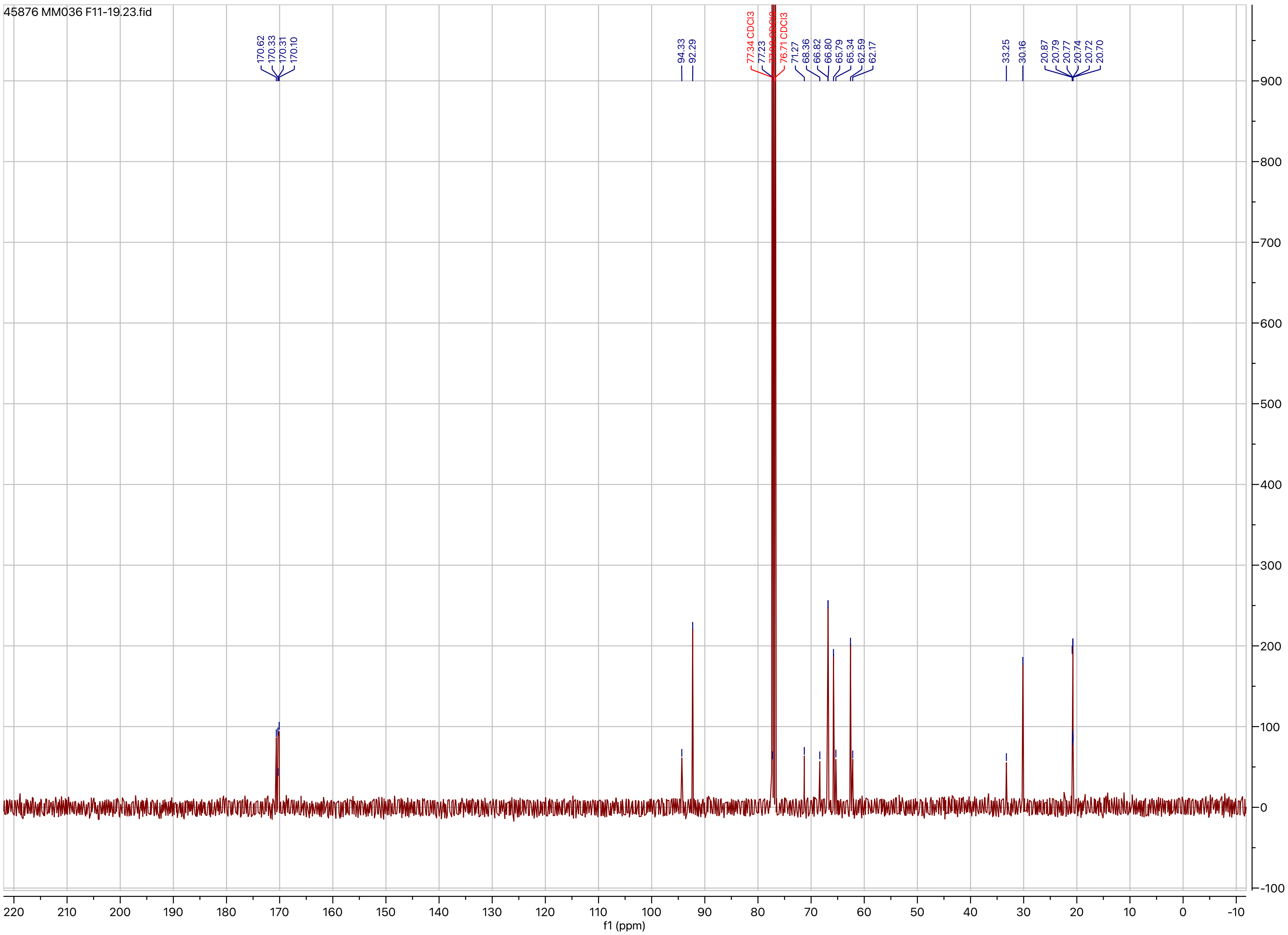

¹H Spectrum of 2c (400 MHz, Chloroform-*d*)

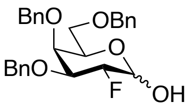

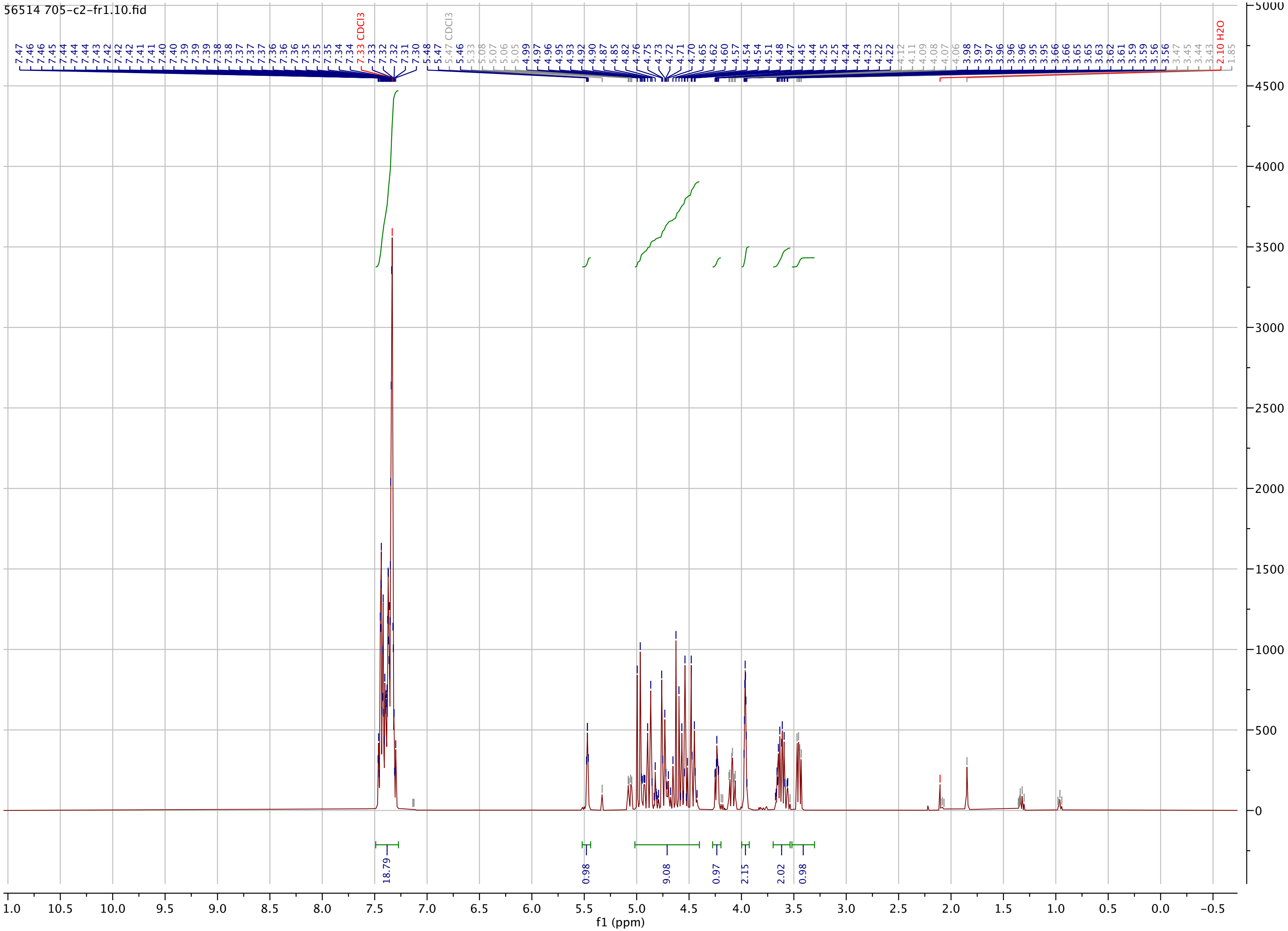

[13]C Spectrum of 2c (101 MHz, Chloroform-*d*)

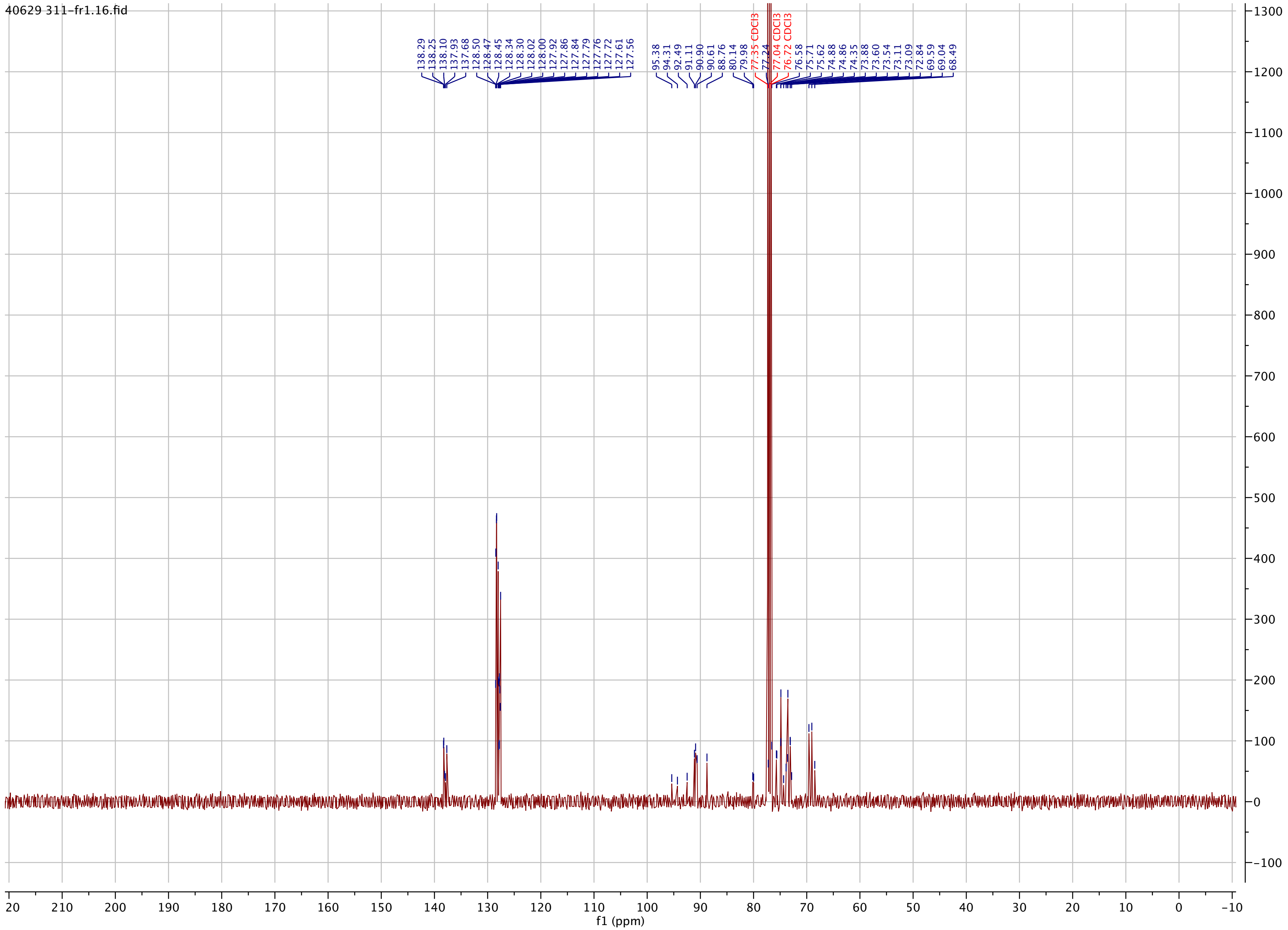

^{19}F Spectrum of 2c (376 MHz, Chloroform-*d*)

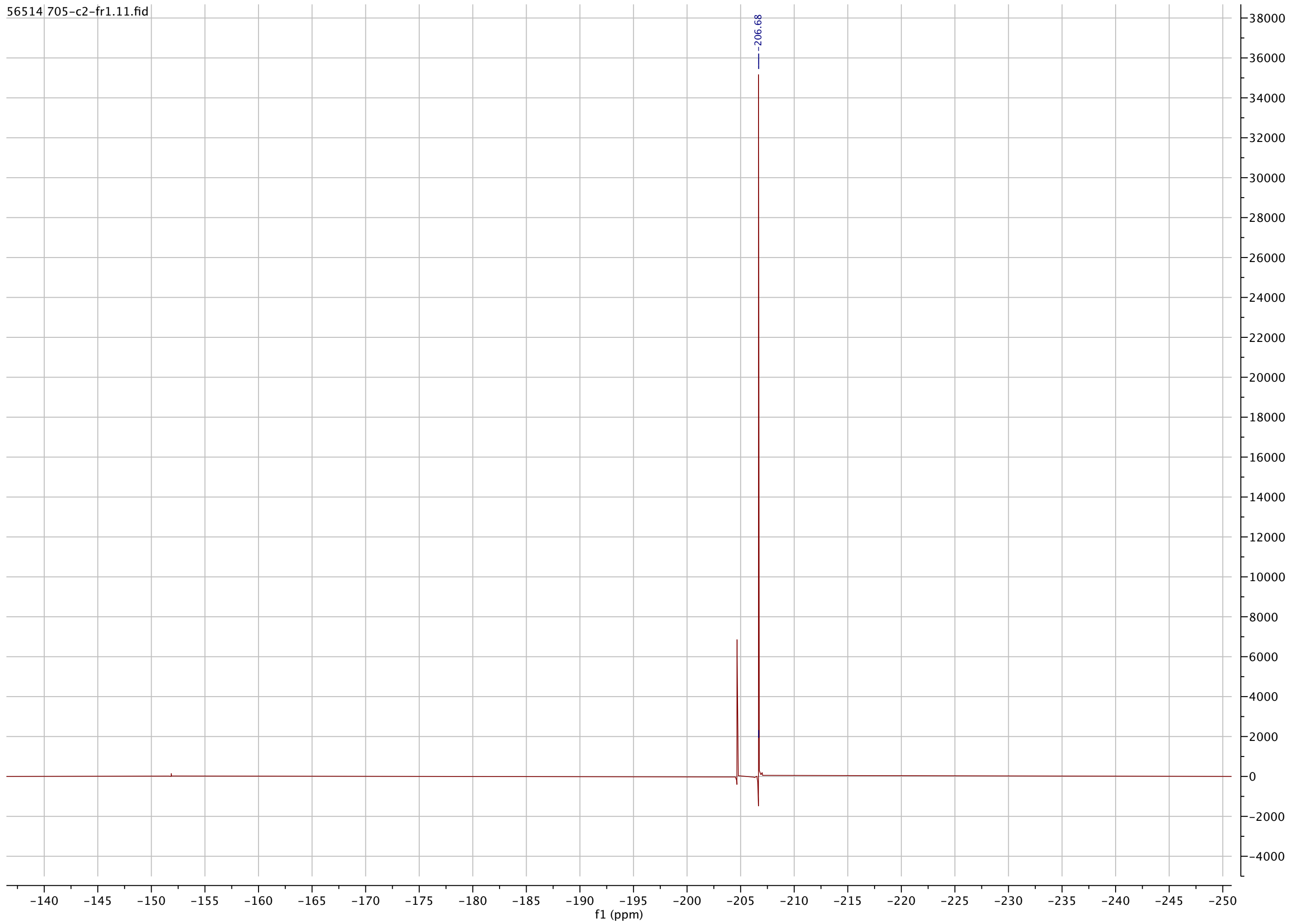

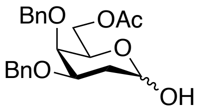

1H Spectrum of 2d (400 MHz, Chloroform-*d*)

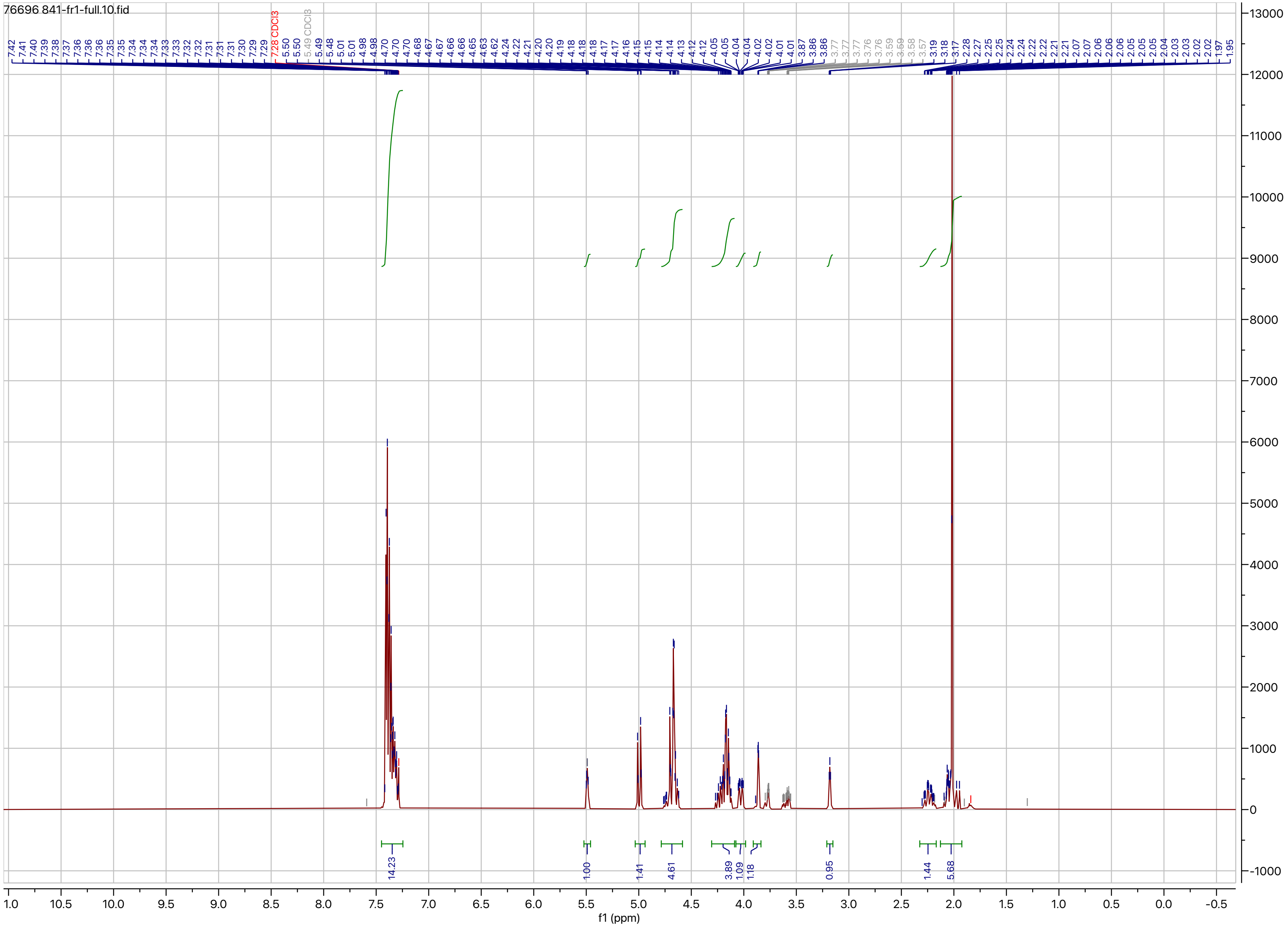

^{13}C Spectrum of 2d (101 MHz, Chloroform-*d*)

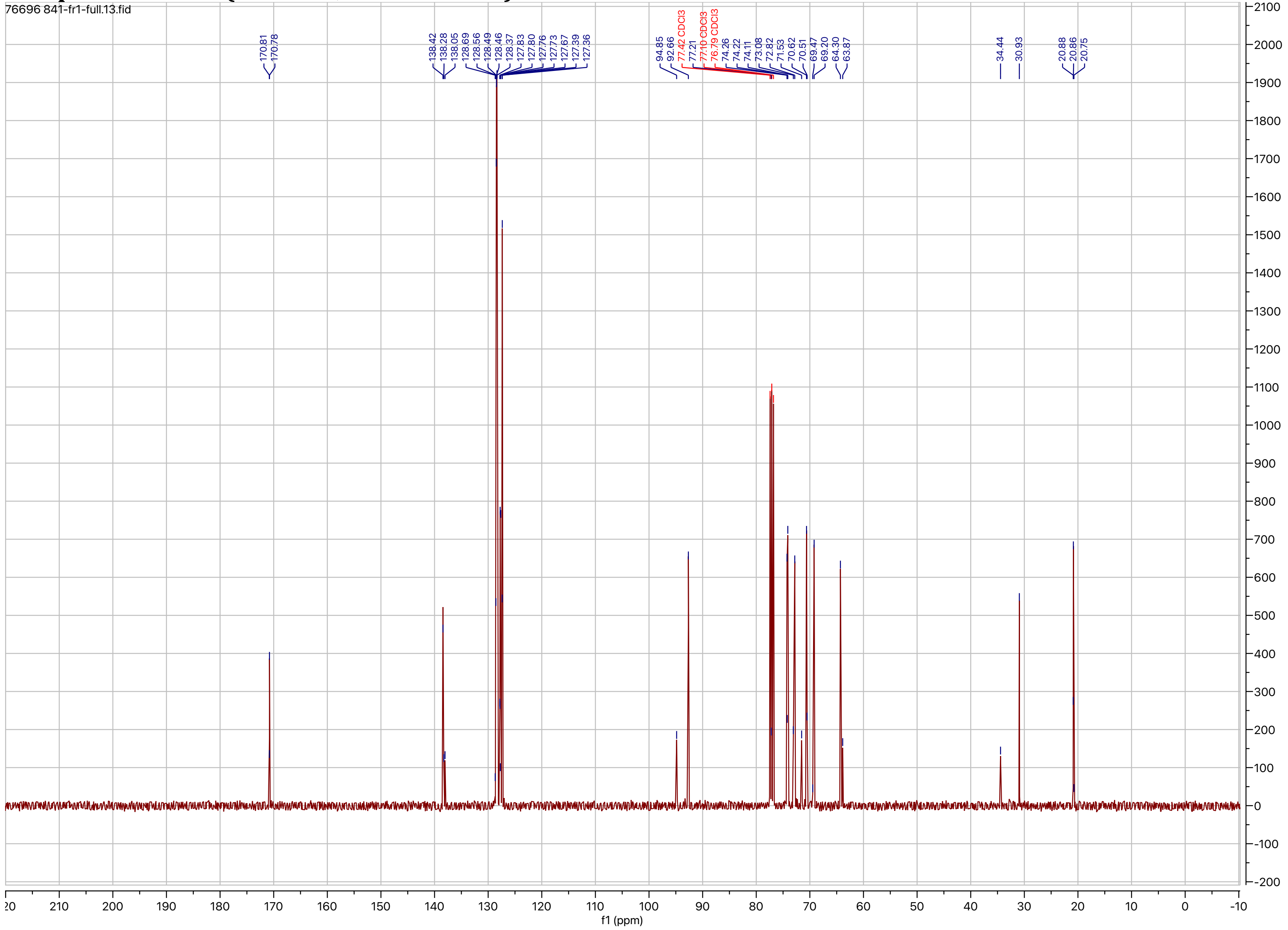

^{1}H Spectrum of S5 (400 MHz, Chloroform-*d*)

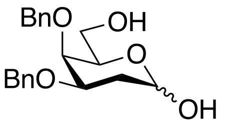

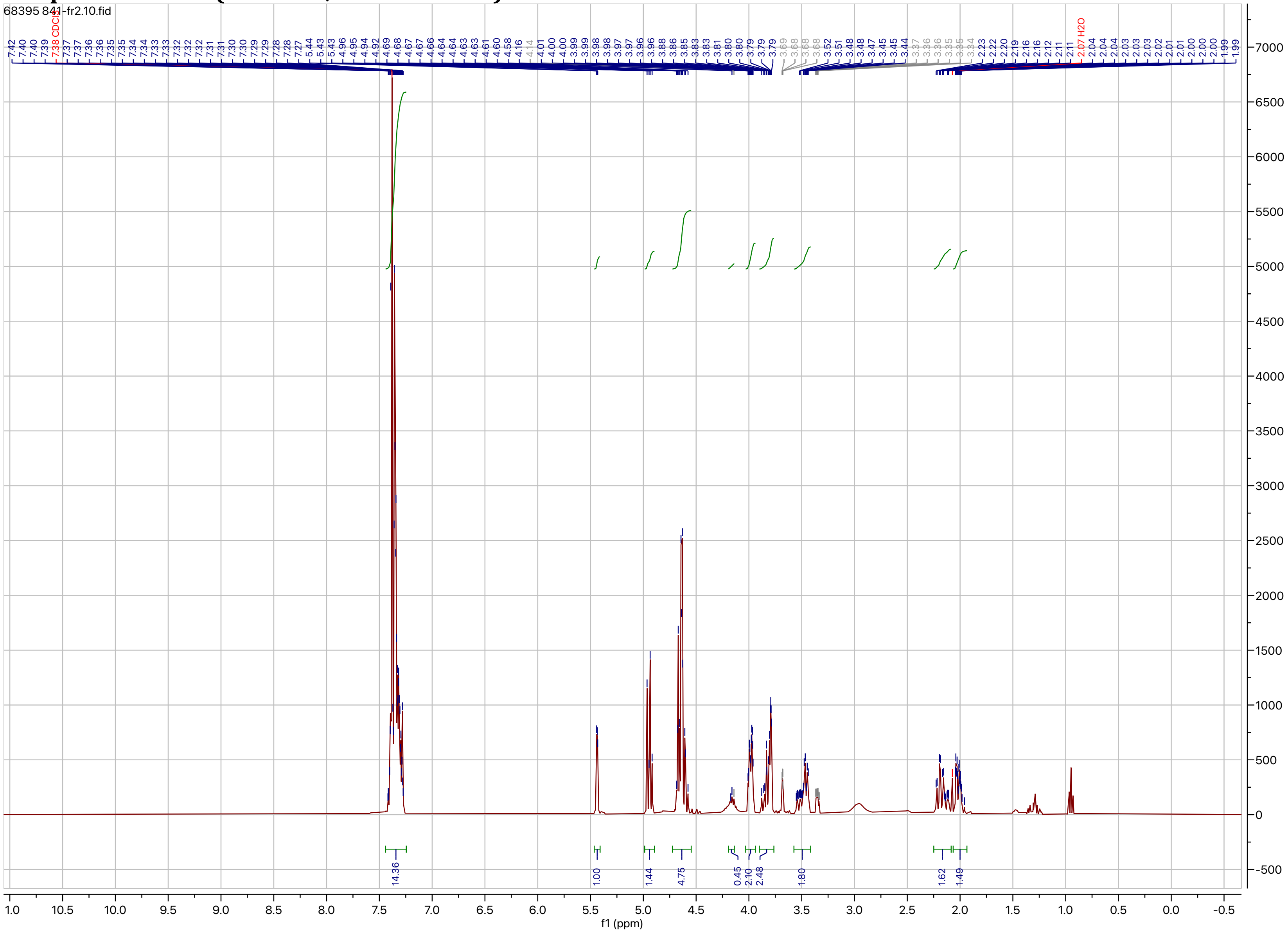

^{13}C Spectrum of S5 (101 MHz, Chloroform-*d*)

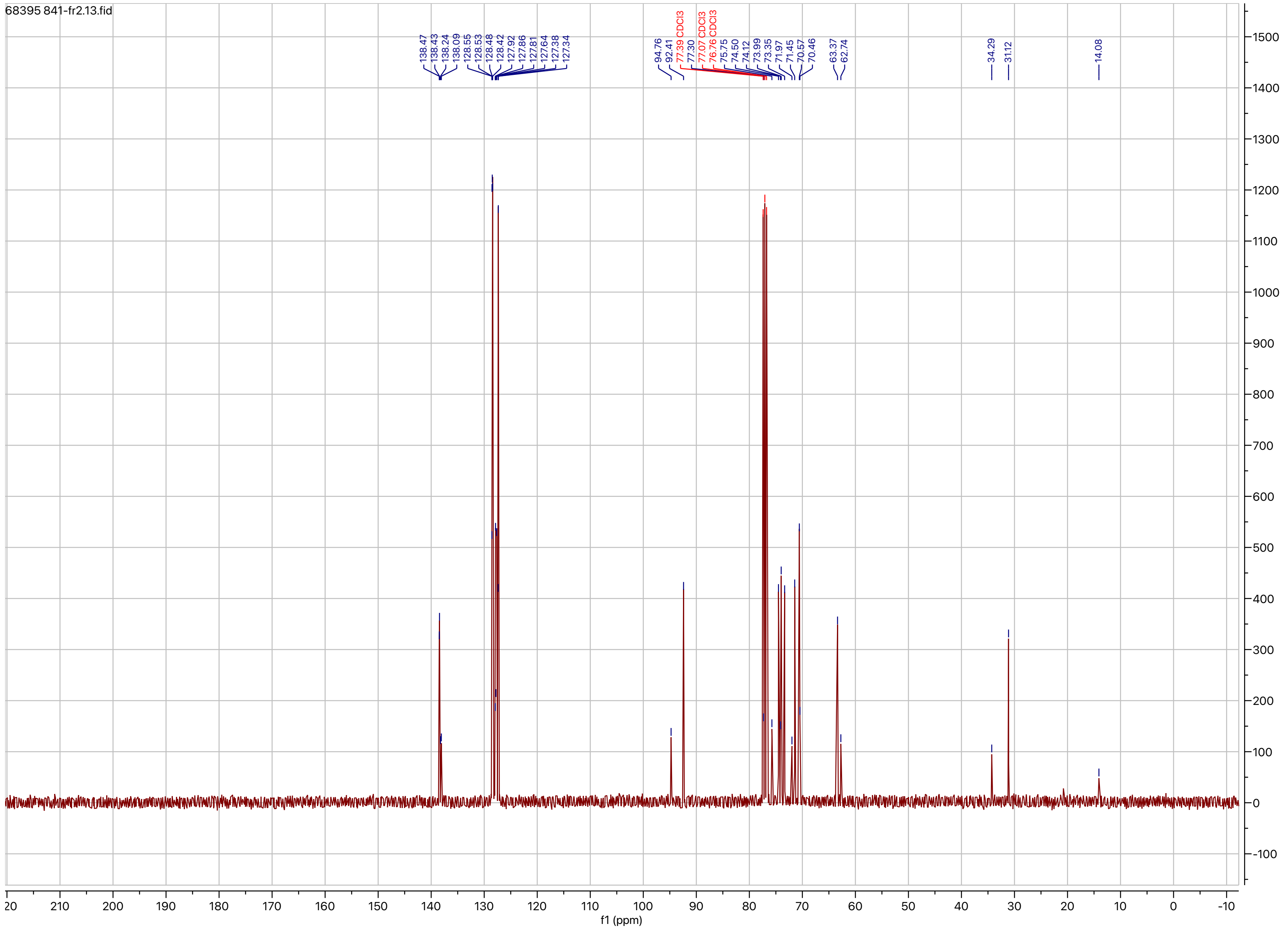

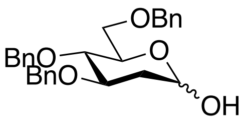

[1]H Spectrum of 3a (400 MHz, Chloroform-*d*)

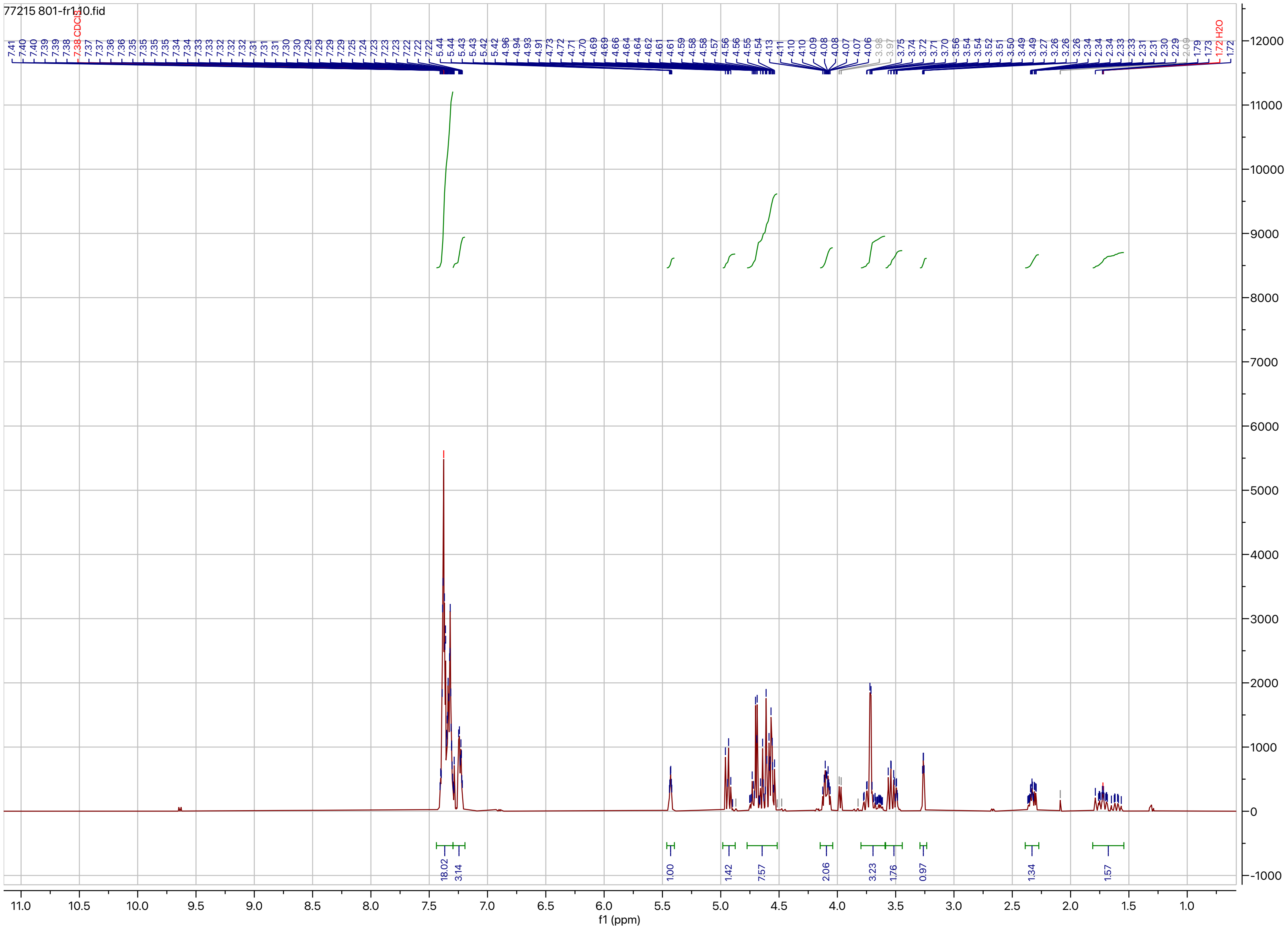

^{13}C Spectrum of 3a (101 MHz, Chloroform-*d*)

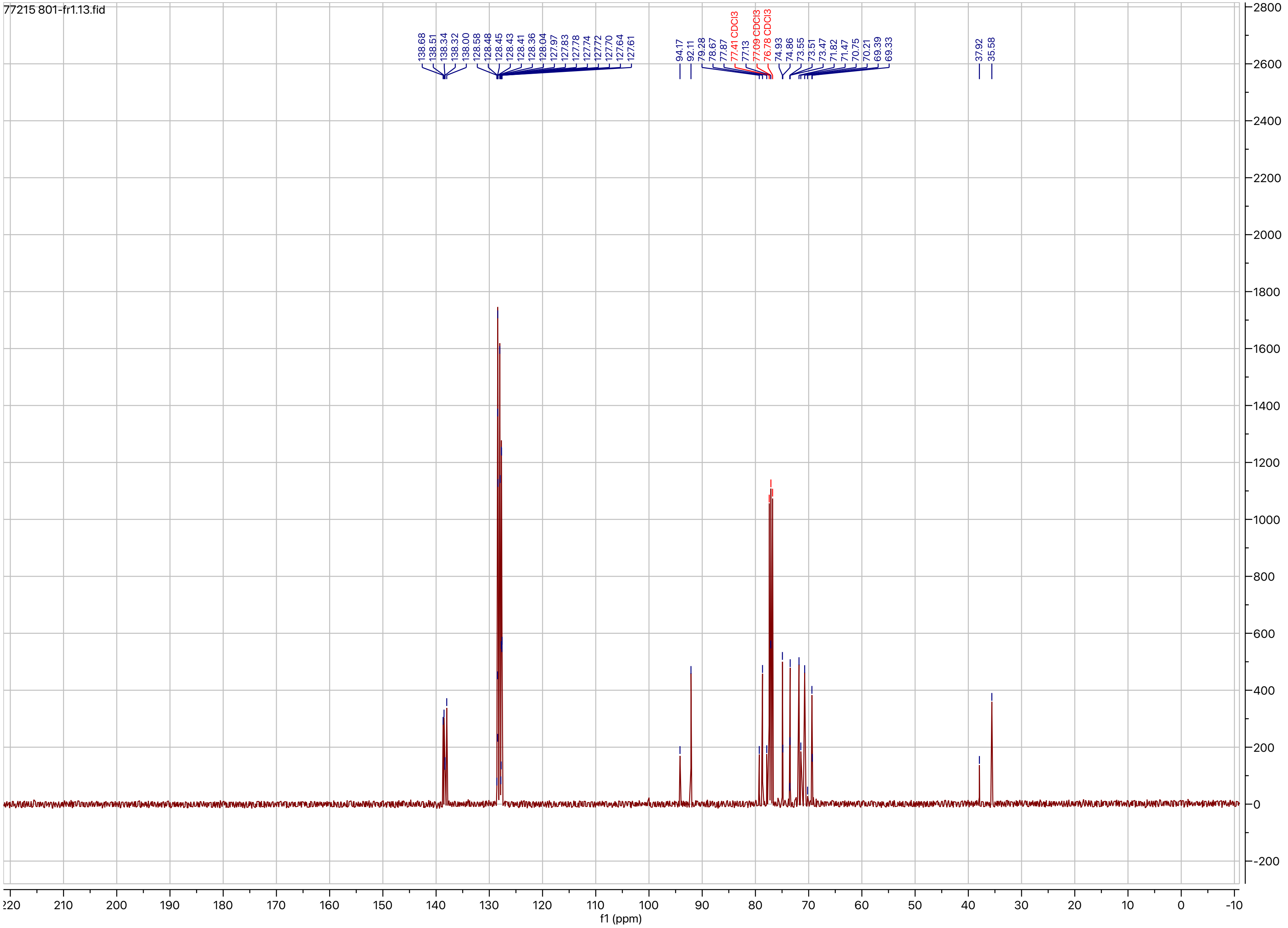

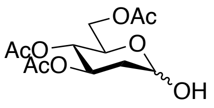

[1]H Spectrum of 3b (400 MHz, Chloroform-*d*)

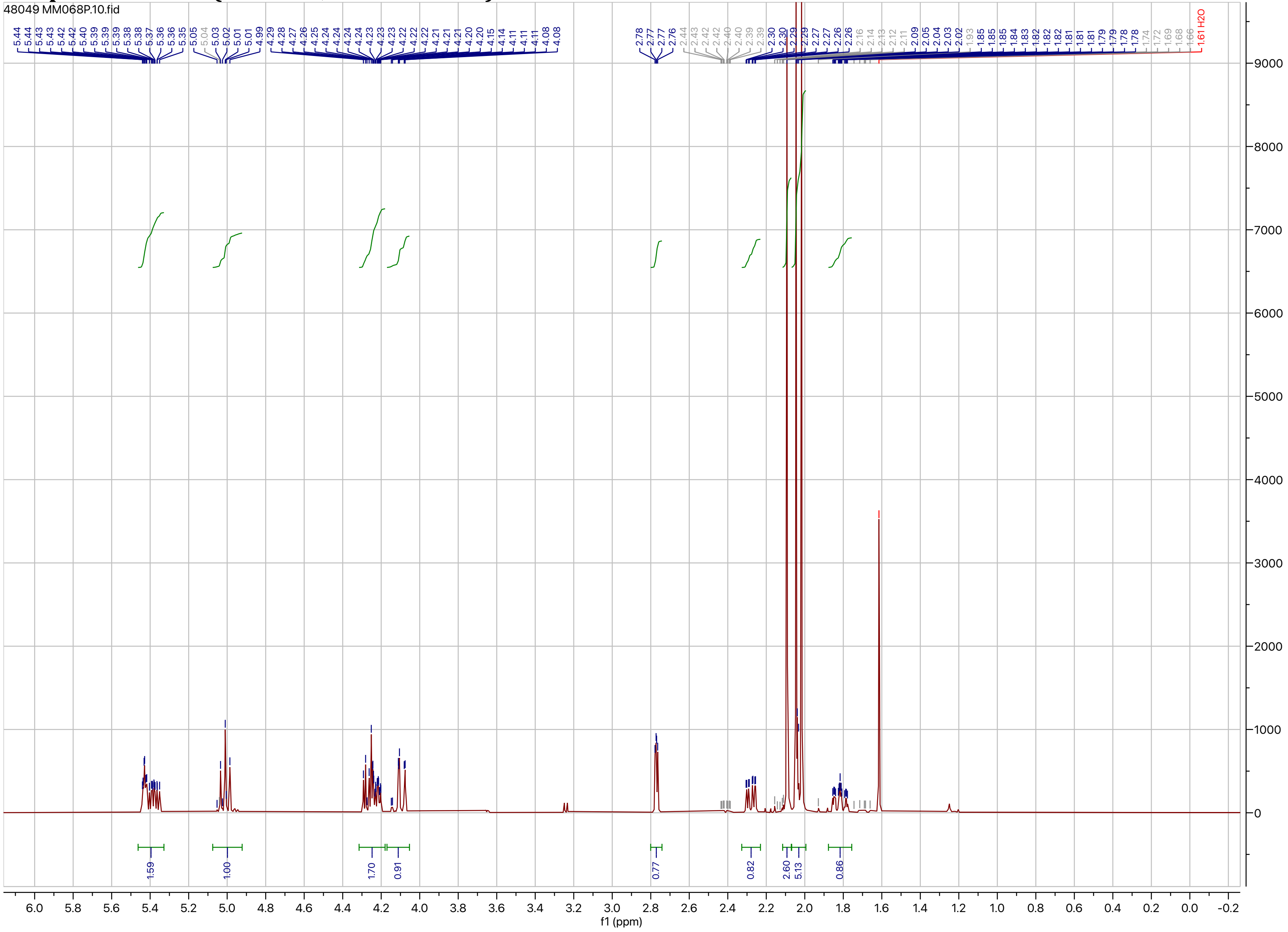

^{13}C Spectrum of 3b (101 MHz, Chloroform-*d*)

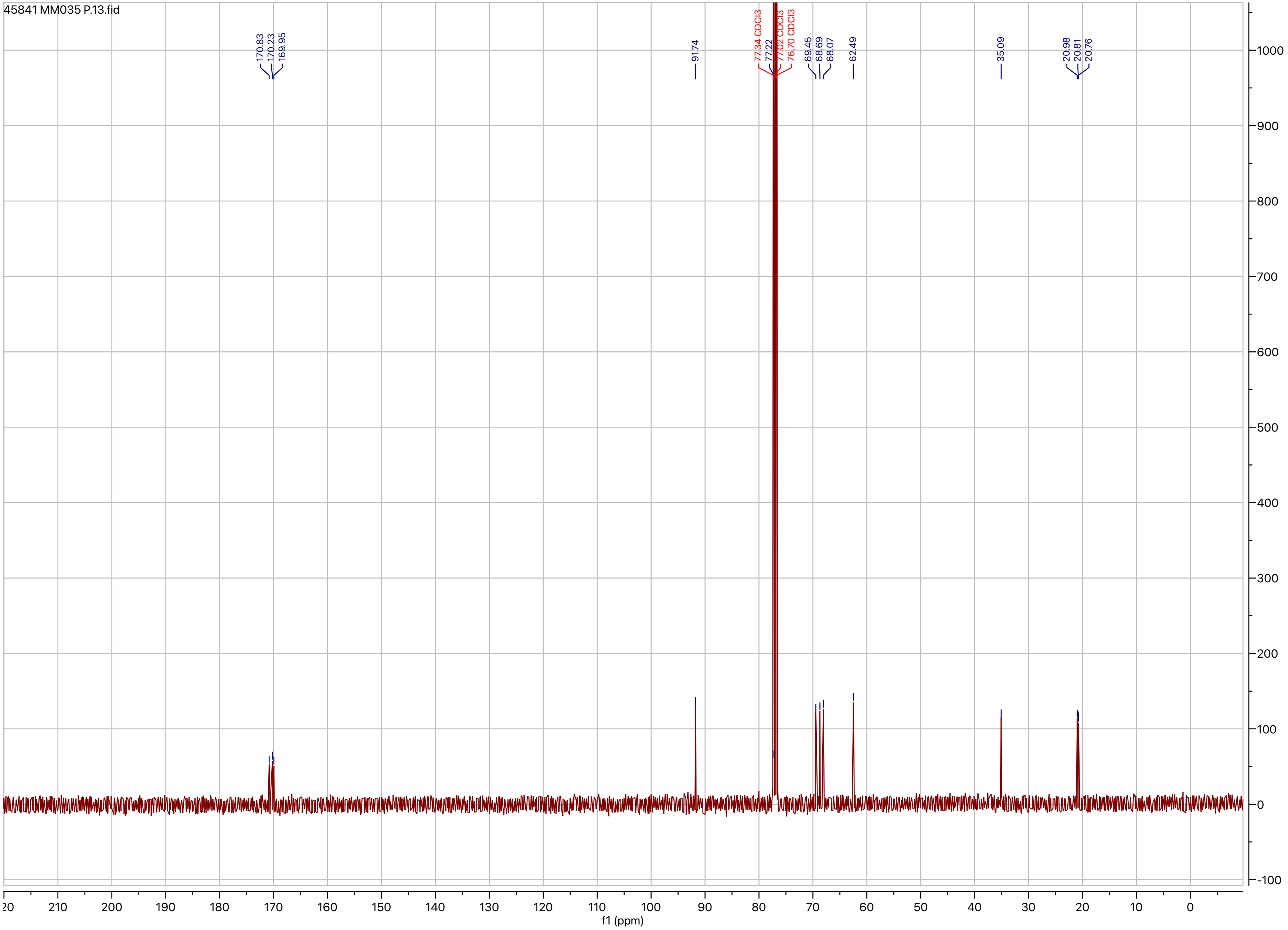

^{1}H Spectrum of 5a (400 MHz, Chloroform-*d*)

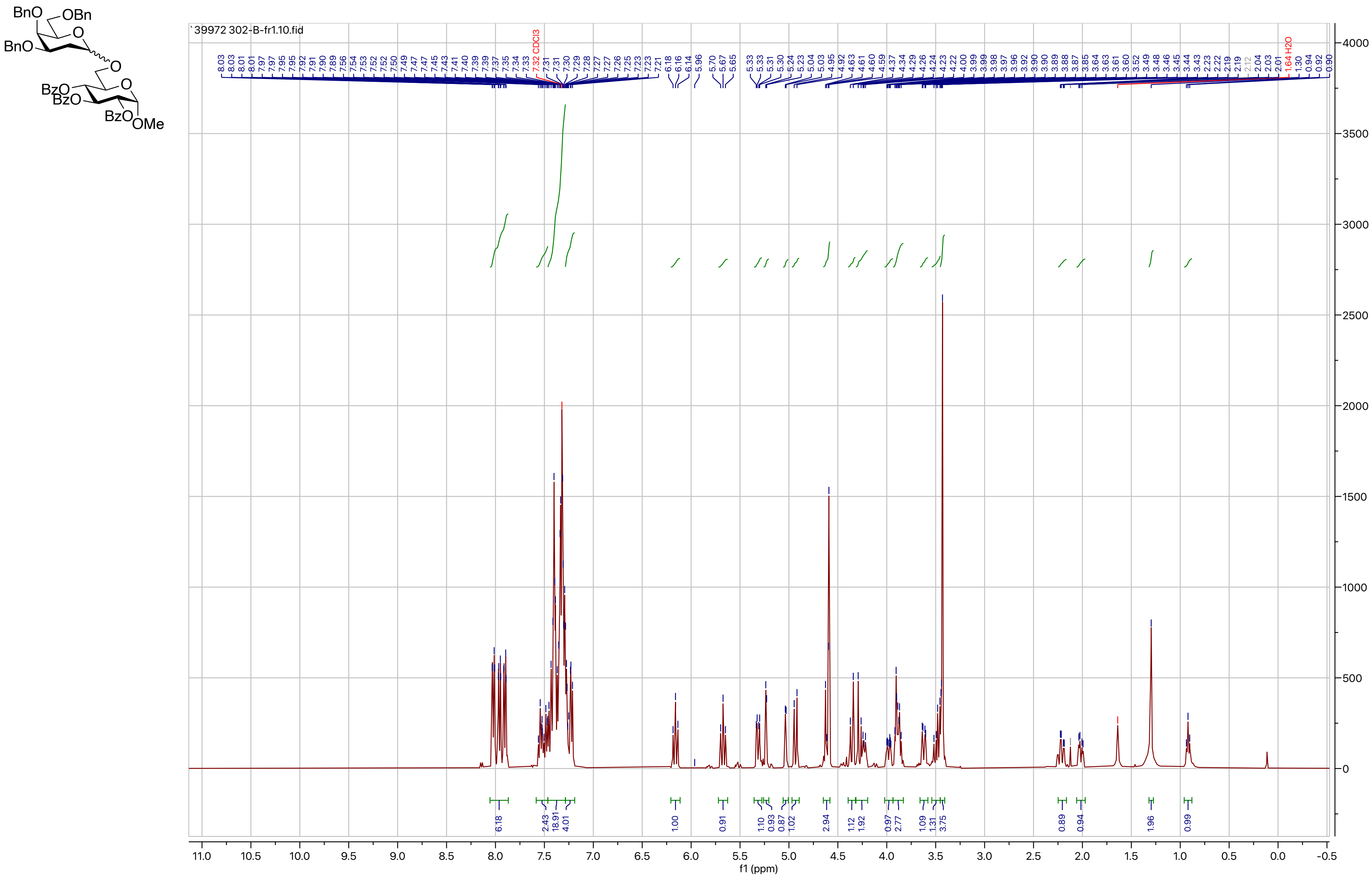

^{13}C Spectrum of 5a (101 MHz, Chloroform-*d*)

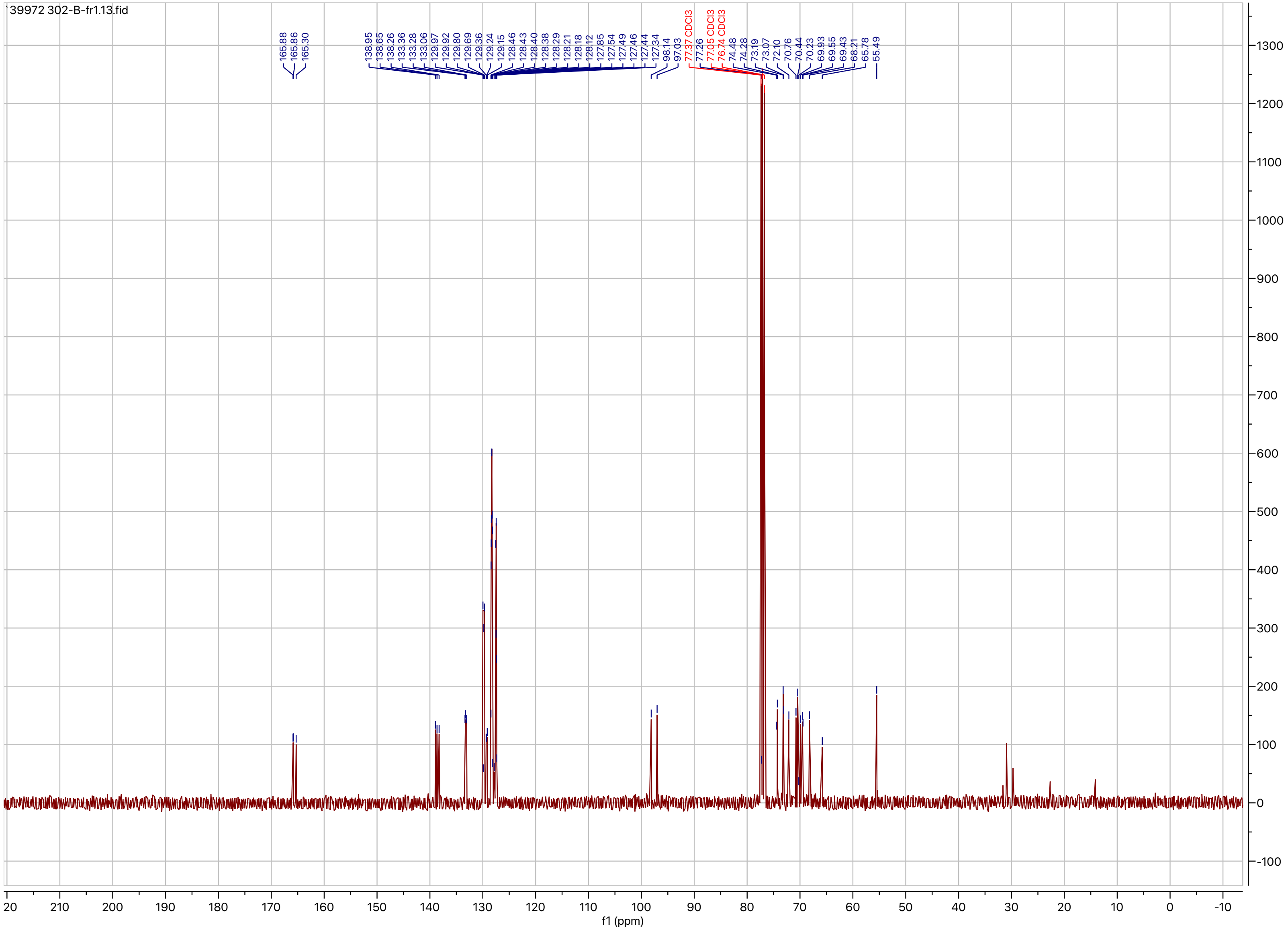

¹H Spectrum of 5b (400 MHz, Chloroform-d)

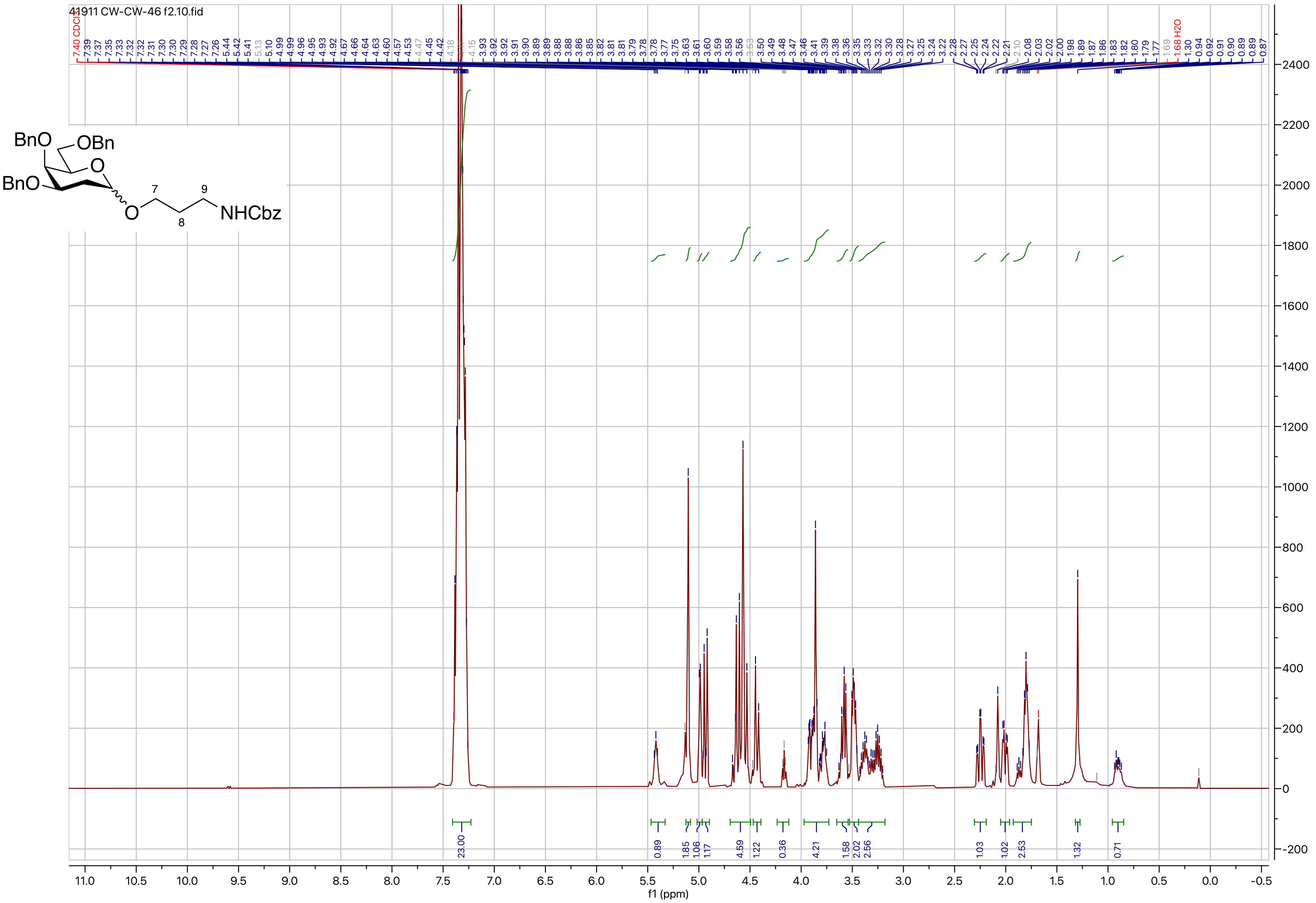

^{13}C Spectrum of 5b (101 MHz, Chloroform-*d*)

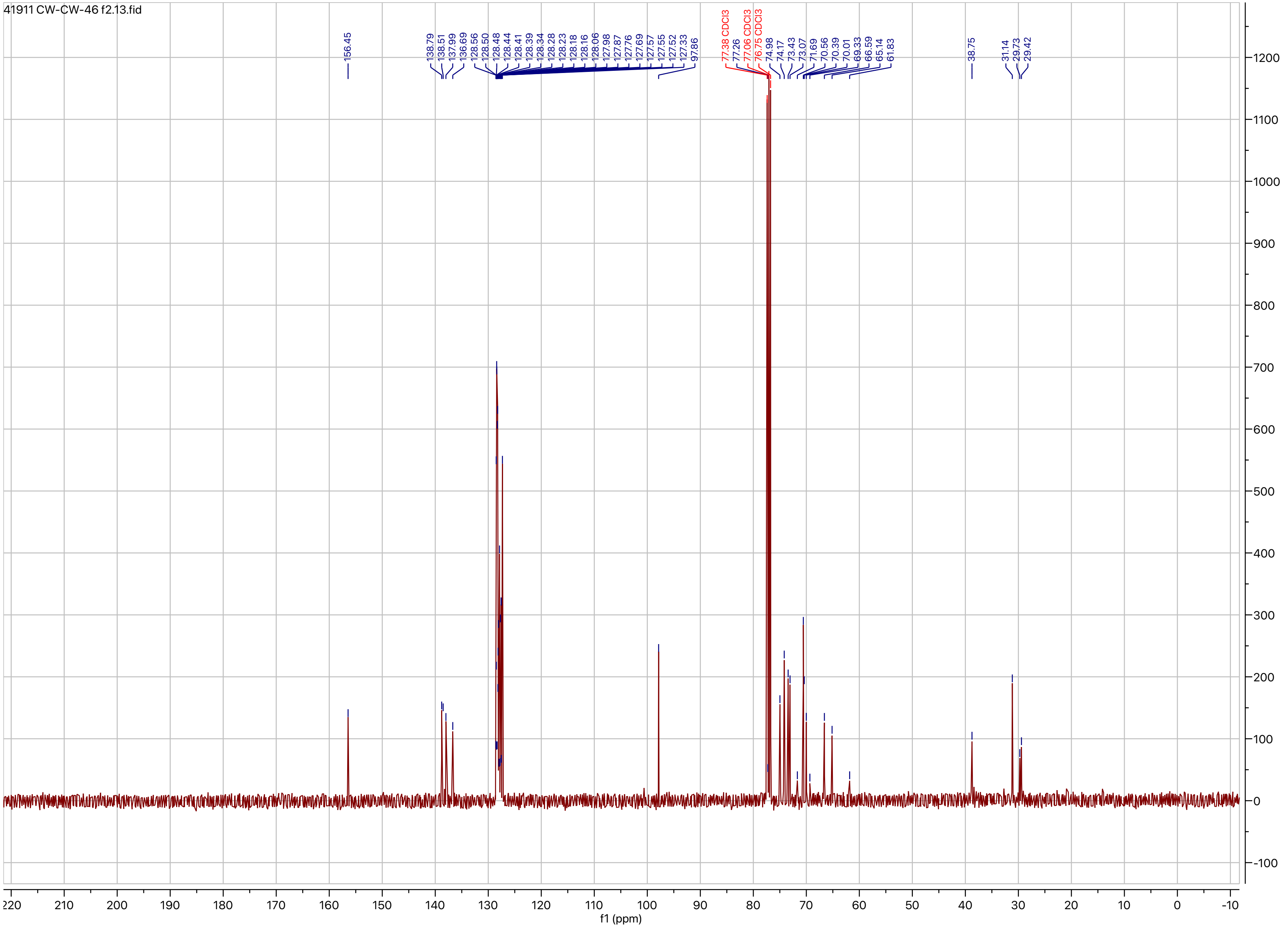

¹H Spectrum of 5c (400 MHz, Chloroform-*d*)

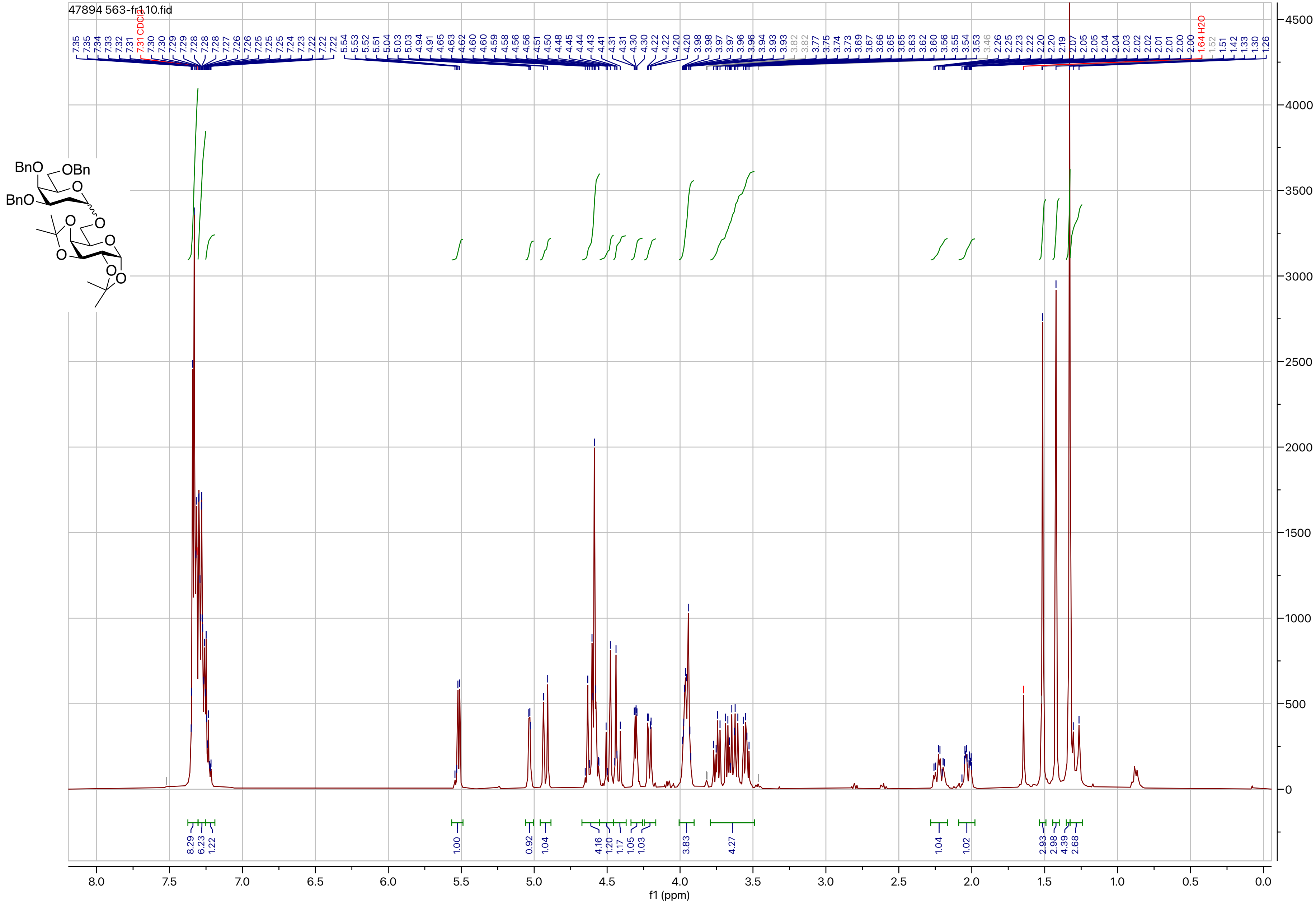

^{13}C Spectrum of 5c (101 MHz, Chloroform-*d*)

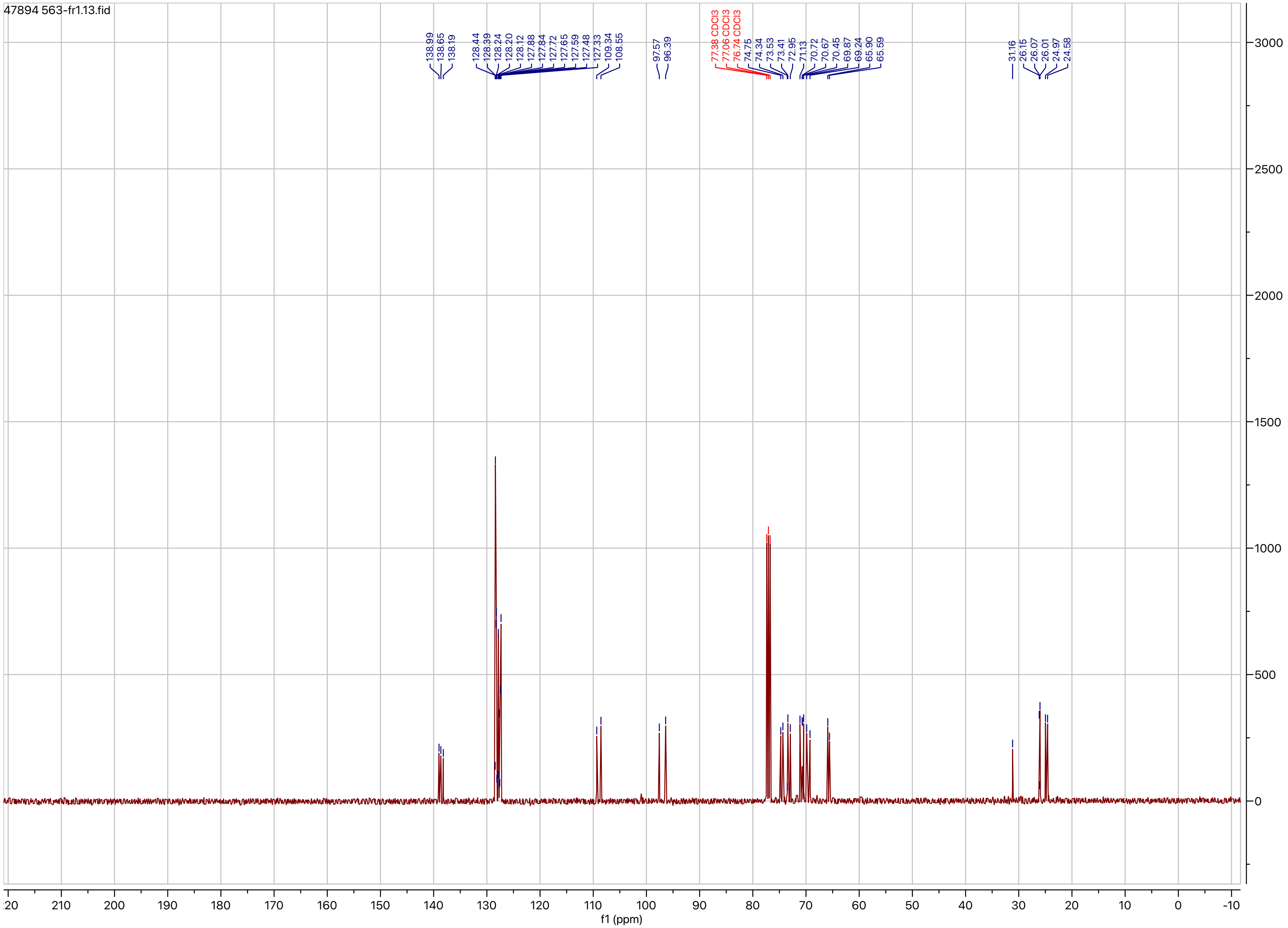

^{1}H Spectrum of 5d (400 MHz, Chloroform-*d*)

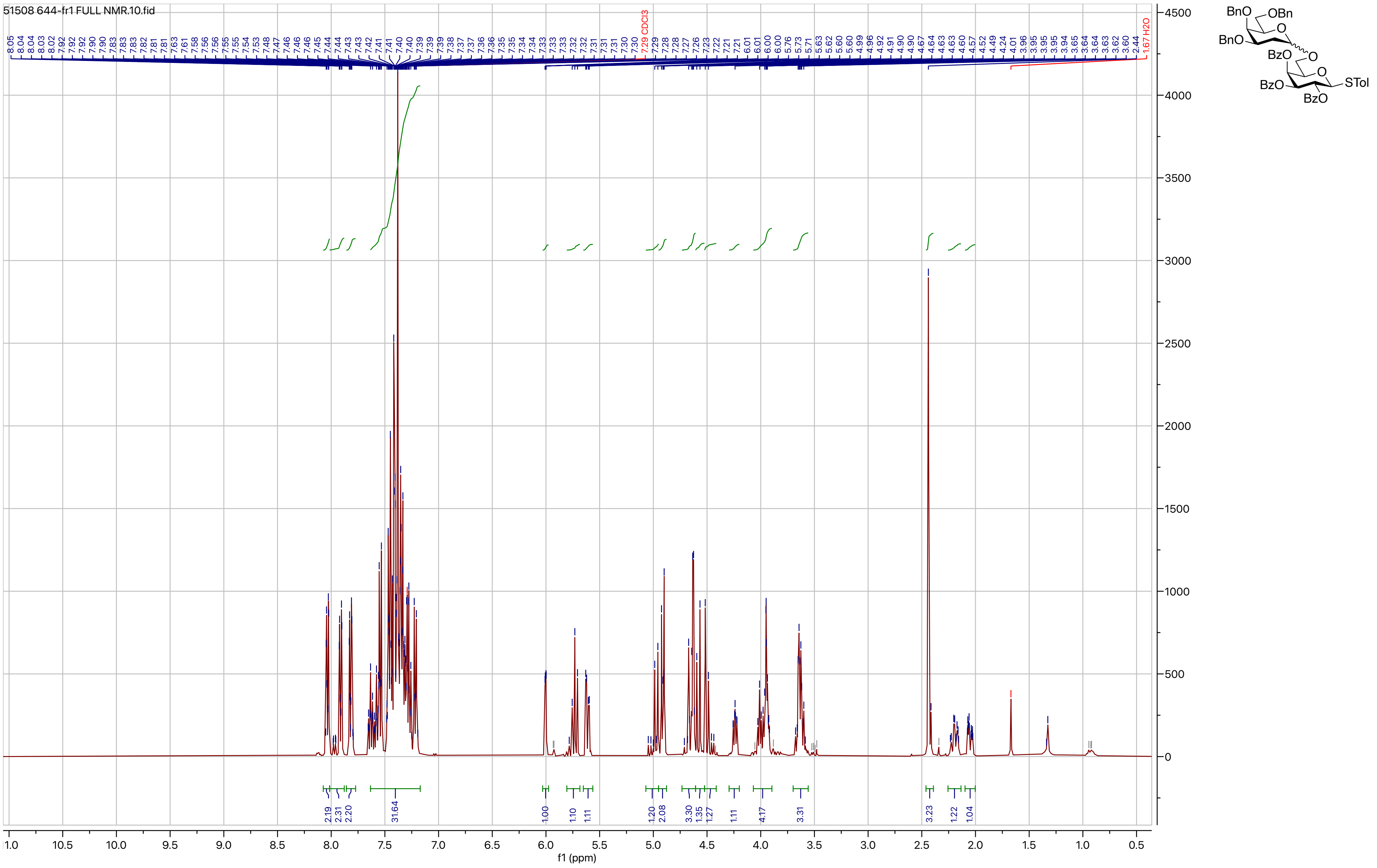

APT Spectrum of 5d (101 MHz, Chloroform-*d*)

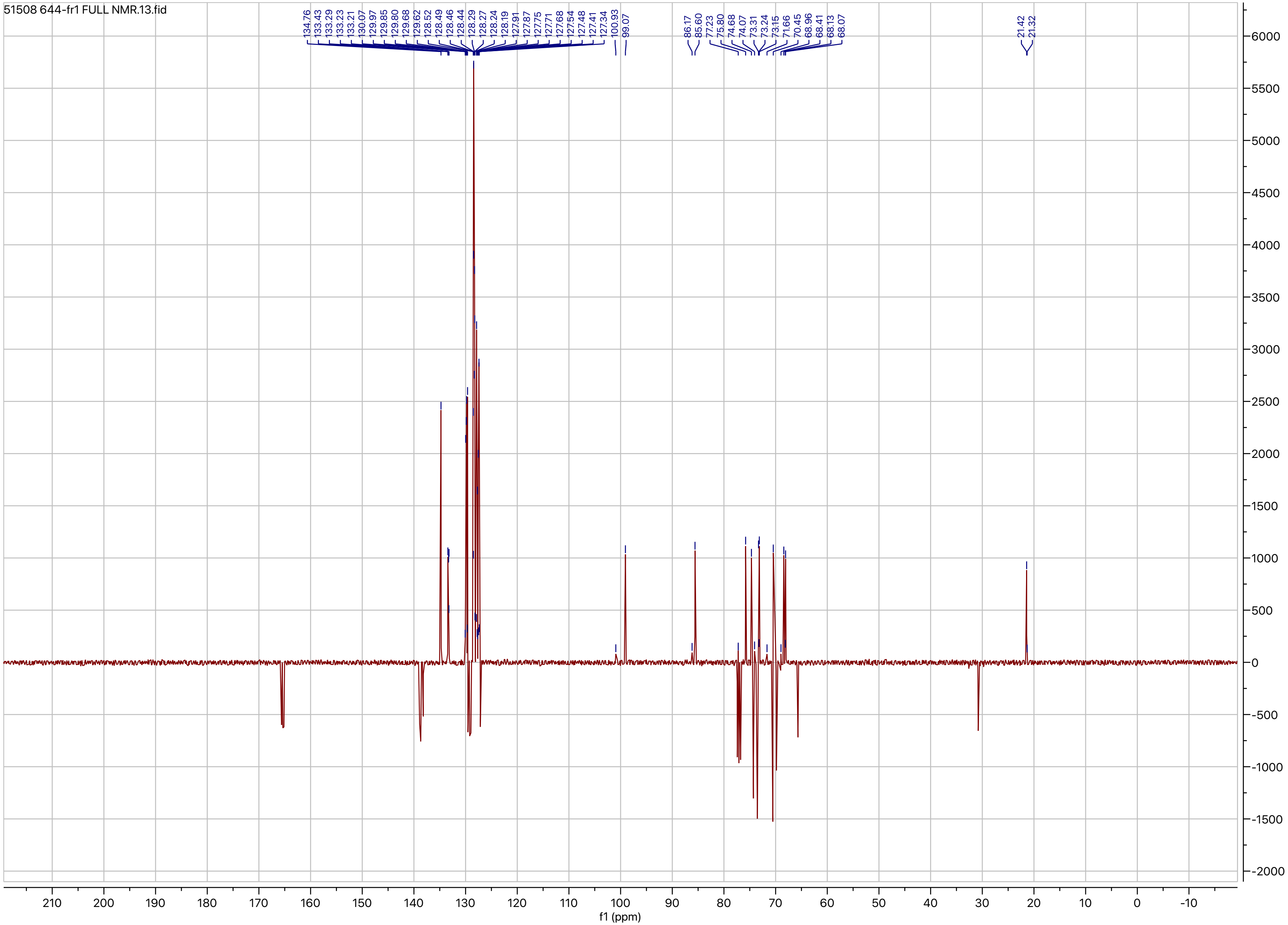

[1]H Spectrum of 5h (400 MHz, Chloroform-*d*)

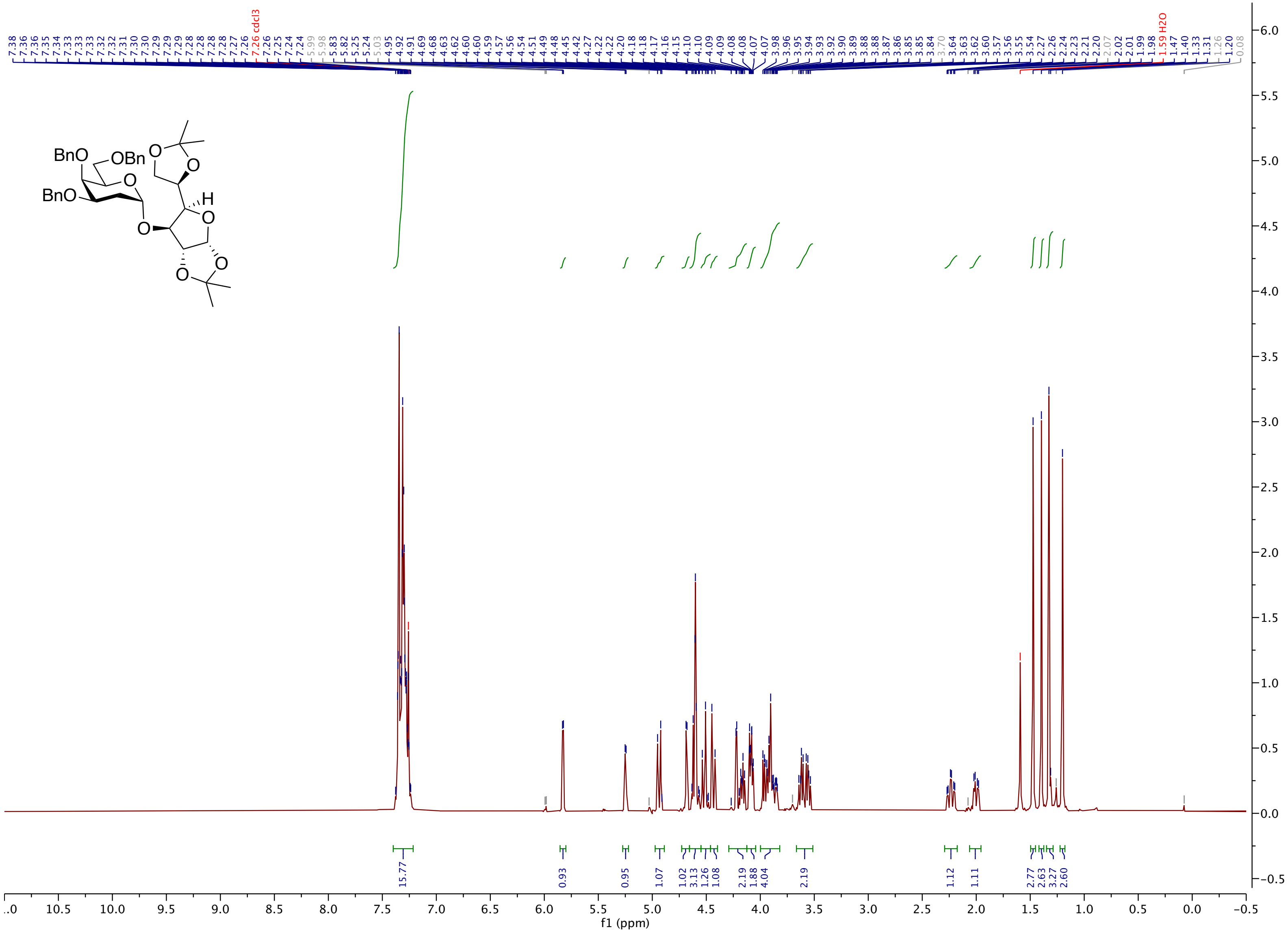

^{13}C Spectrum of 5h (101 MHz, Chloroform-*d*)

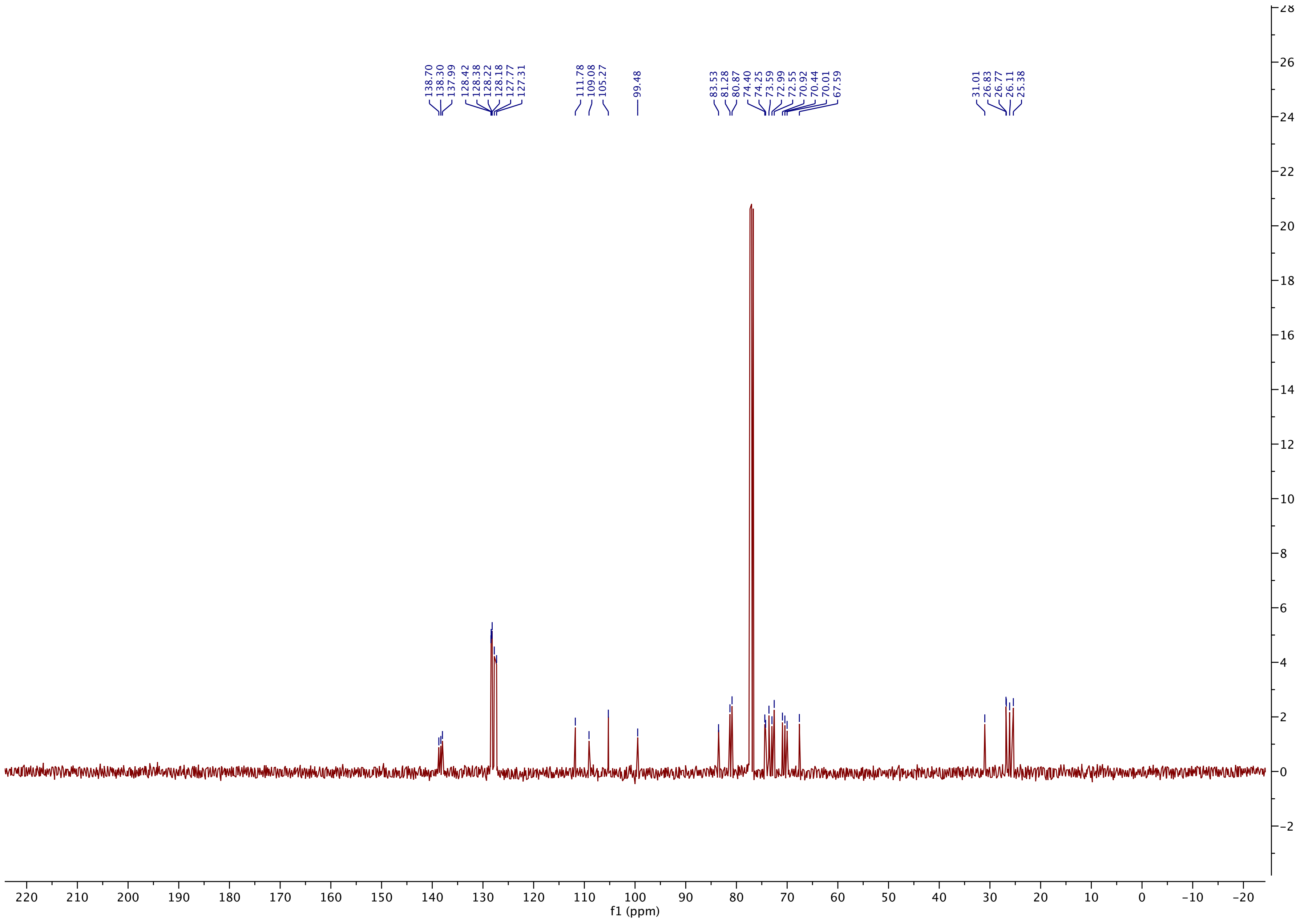

^{1}H Spectrum of 5i (400 MHz, Chloroform-*d*)

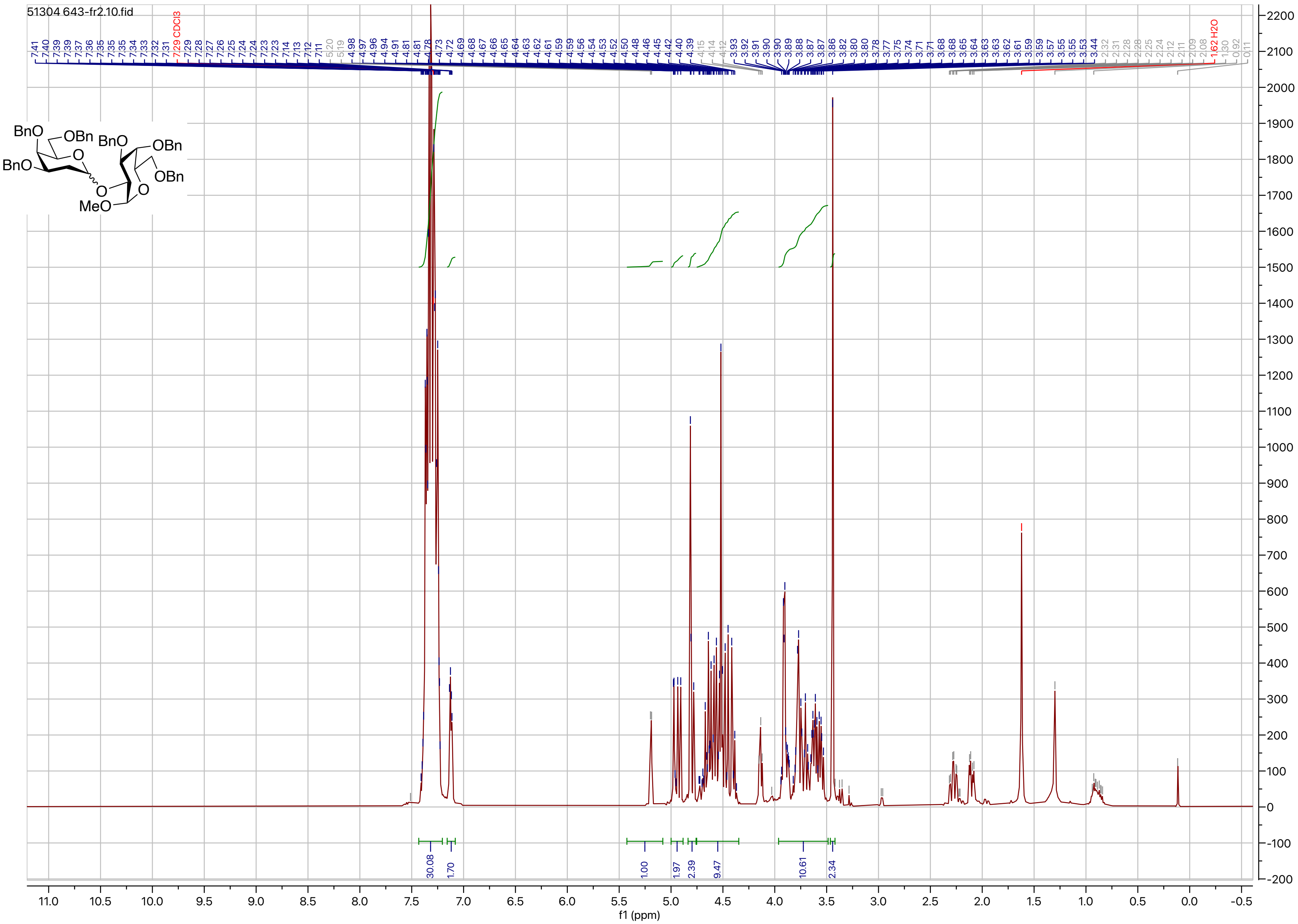

APT Spectrum of 5i (101 MHz, Chloroform-*d*)

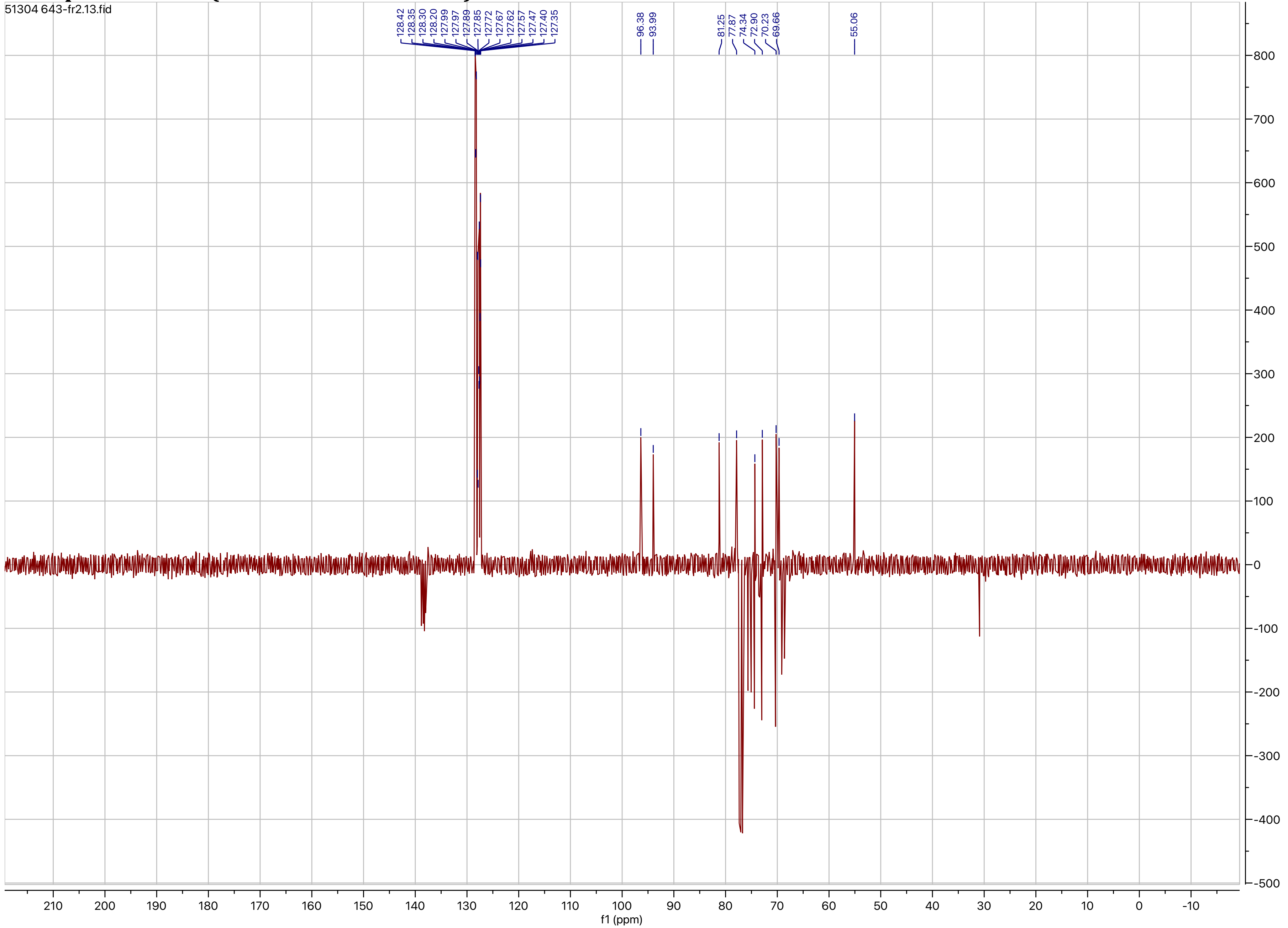

¹H Spectrum of 7b (400 MHz, Chloroform-*d*) c

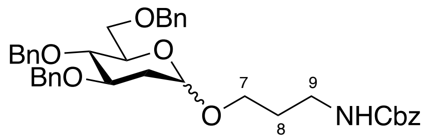

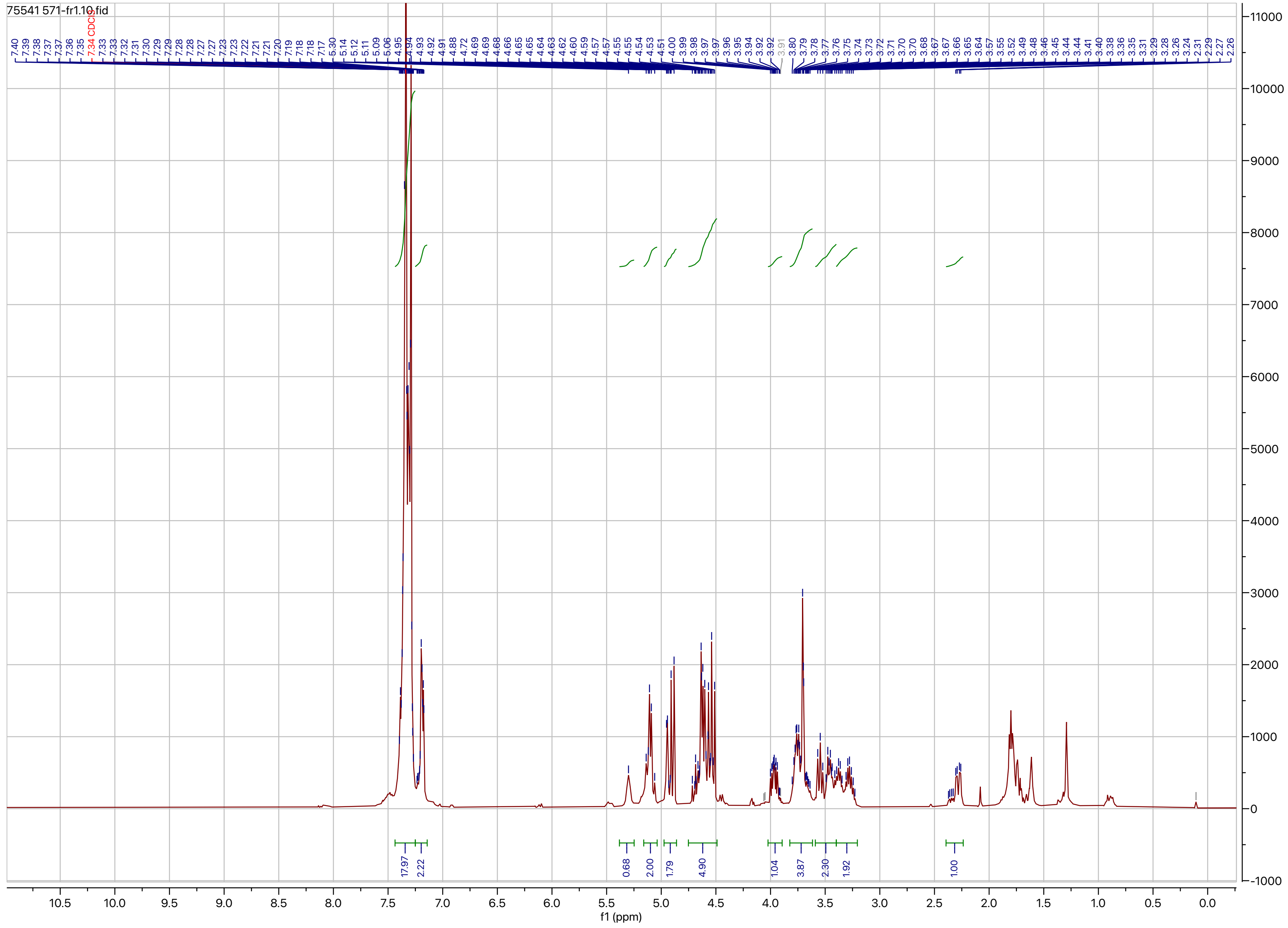

[13]C Spectrum of 7b (101 MHz, Chloroform-*d*)

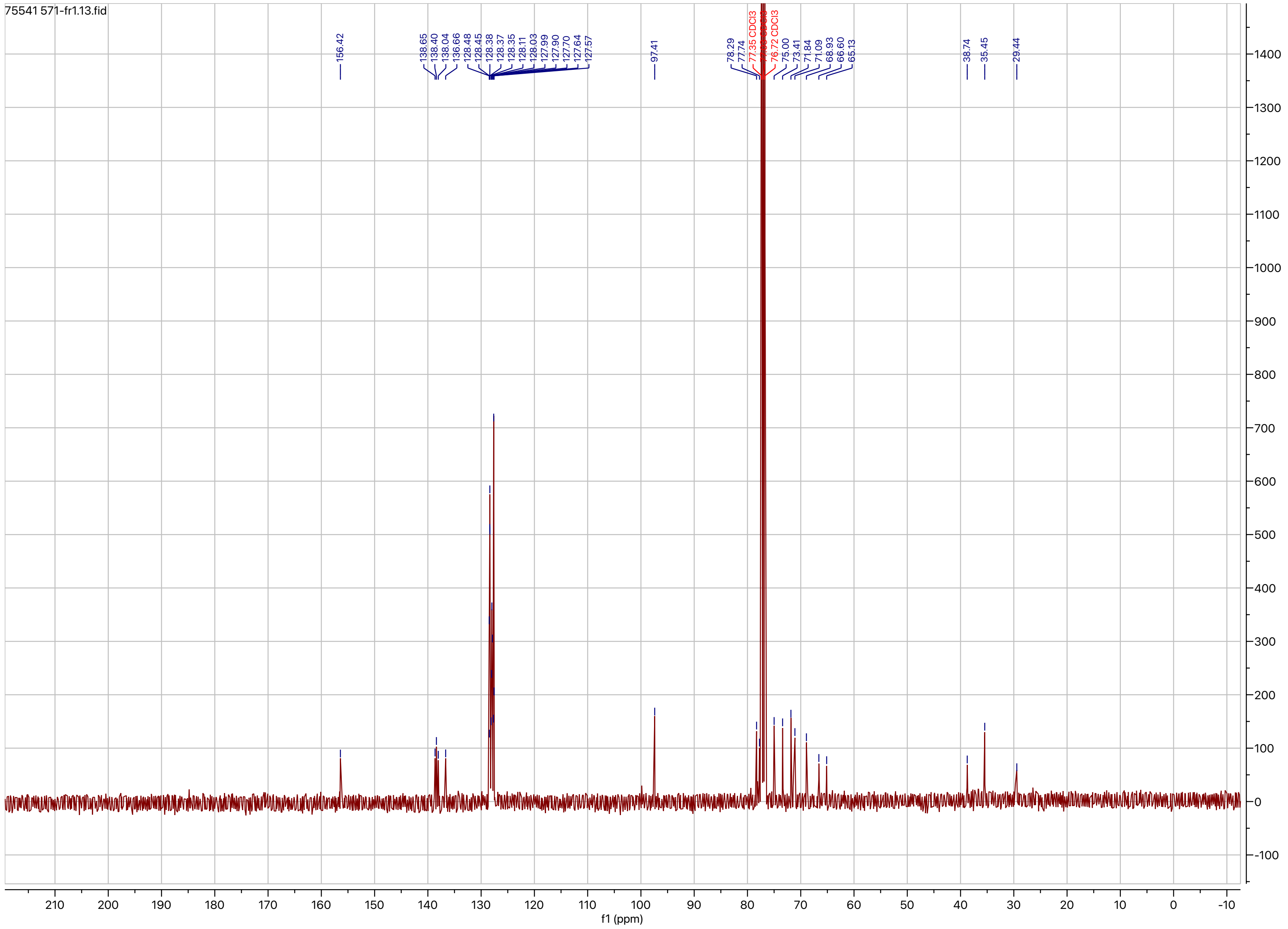

^{1}H Spectrum of 7c (400 MHz, Chloroform-*d*)

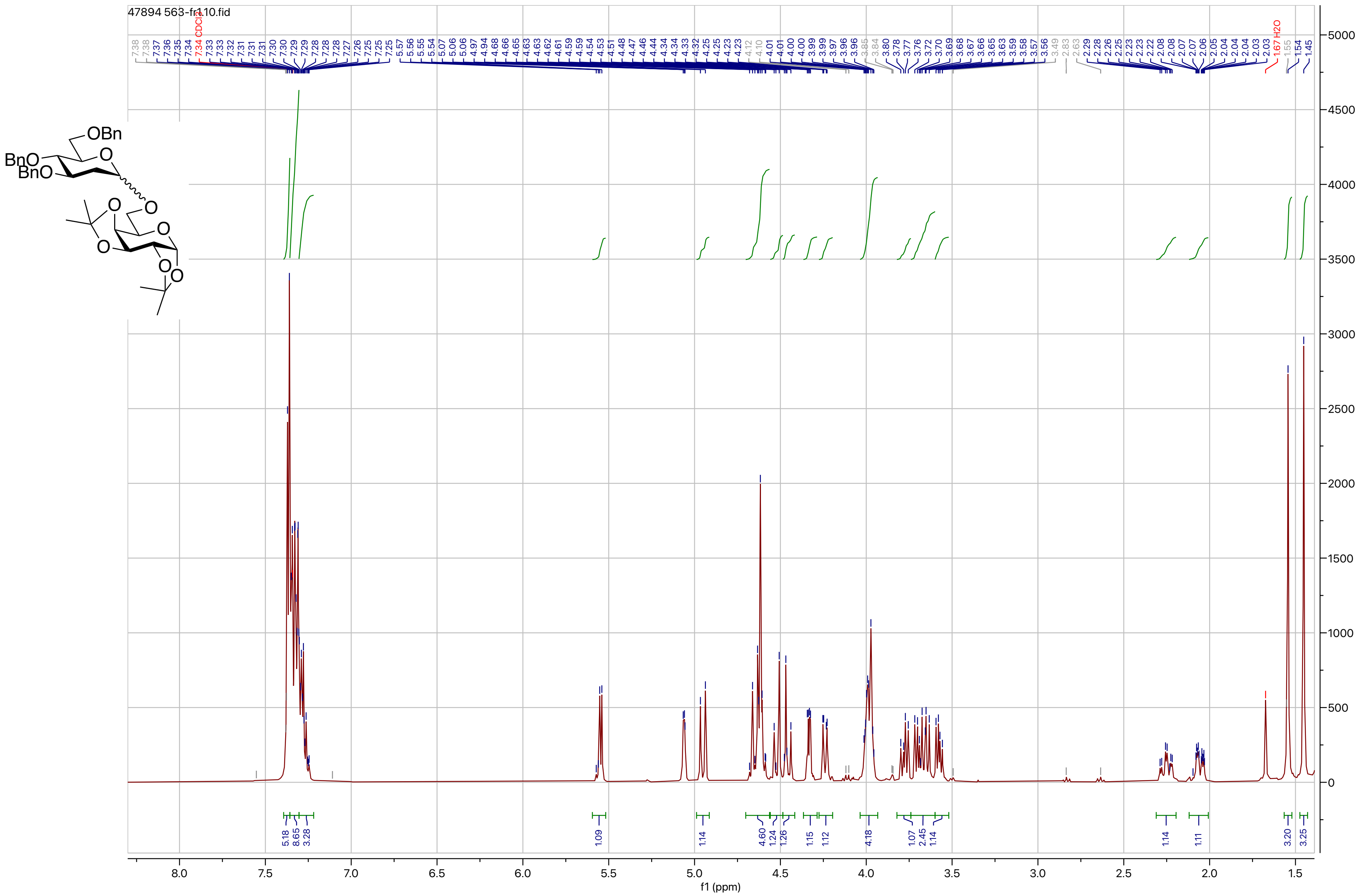

^{13}C Spectrum of 7c (101 MHz, Chloroform-*d*)

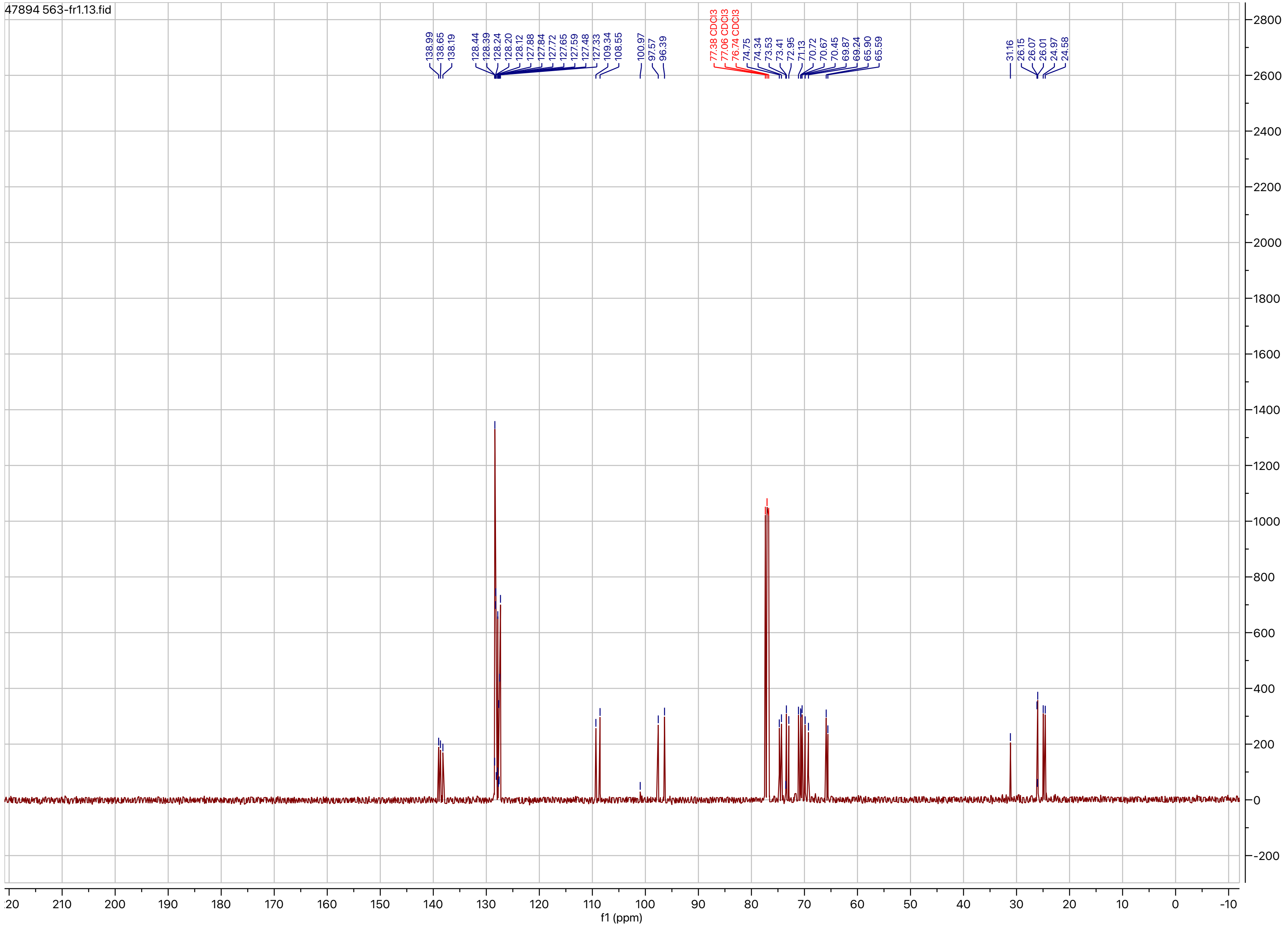

^{1}H Spectrum of 7e (400 MHz, Chloroform-*d*)

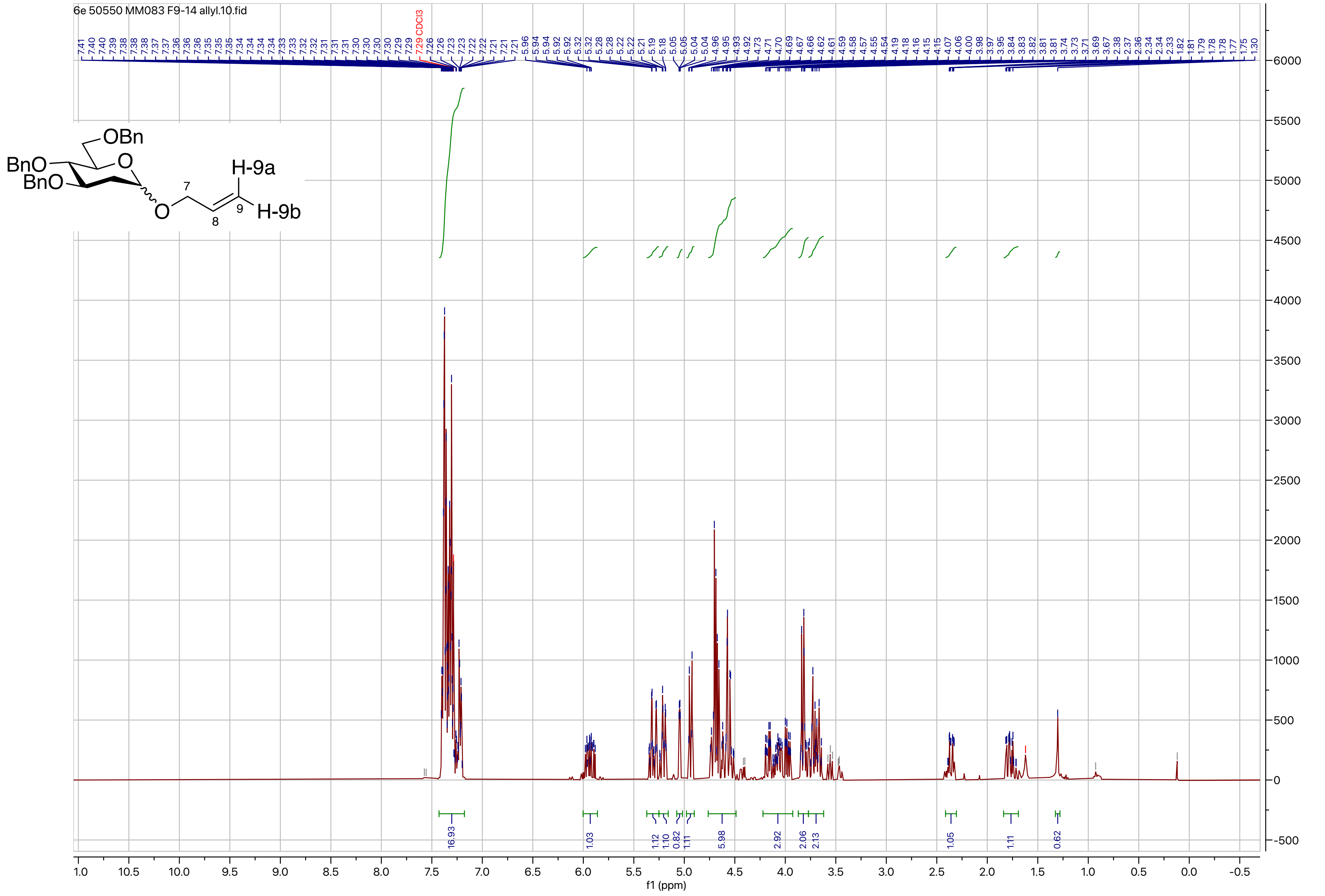

^{13}C Spectrum of 7e (101 MHz, Chloroform-*d*)

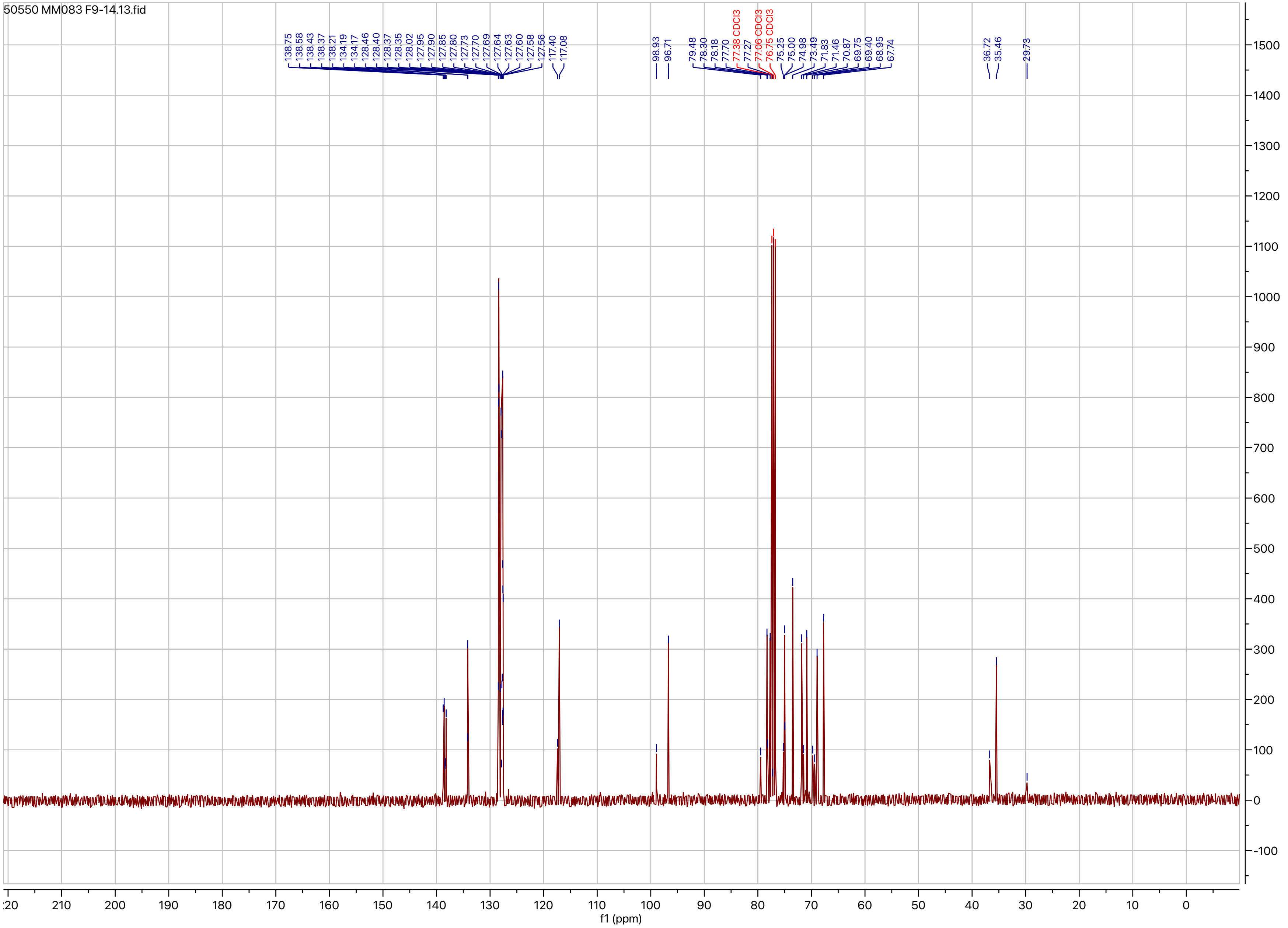

[1]H Spectrum of 7f (400 MHz, Chloroform-*d*)

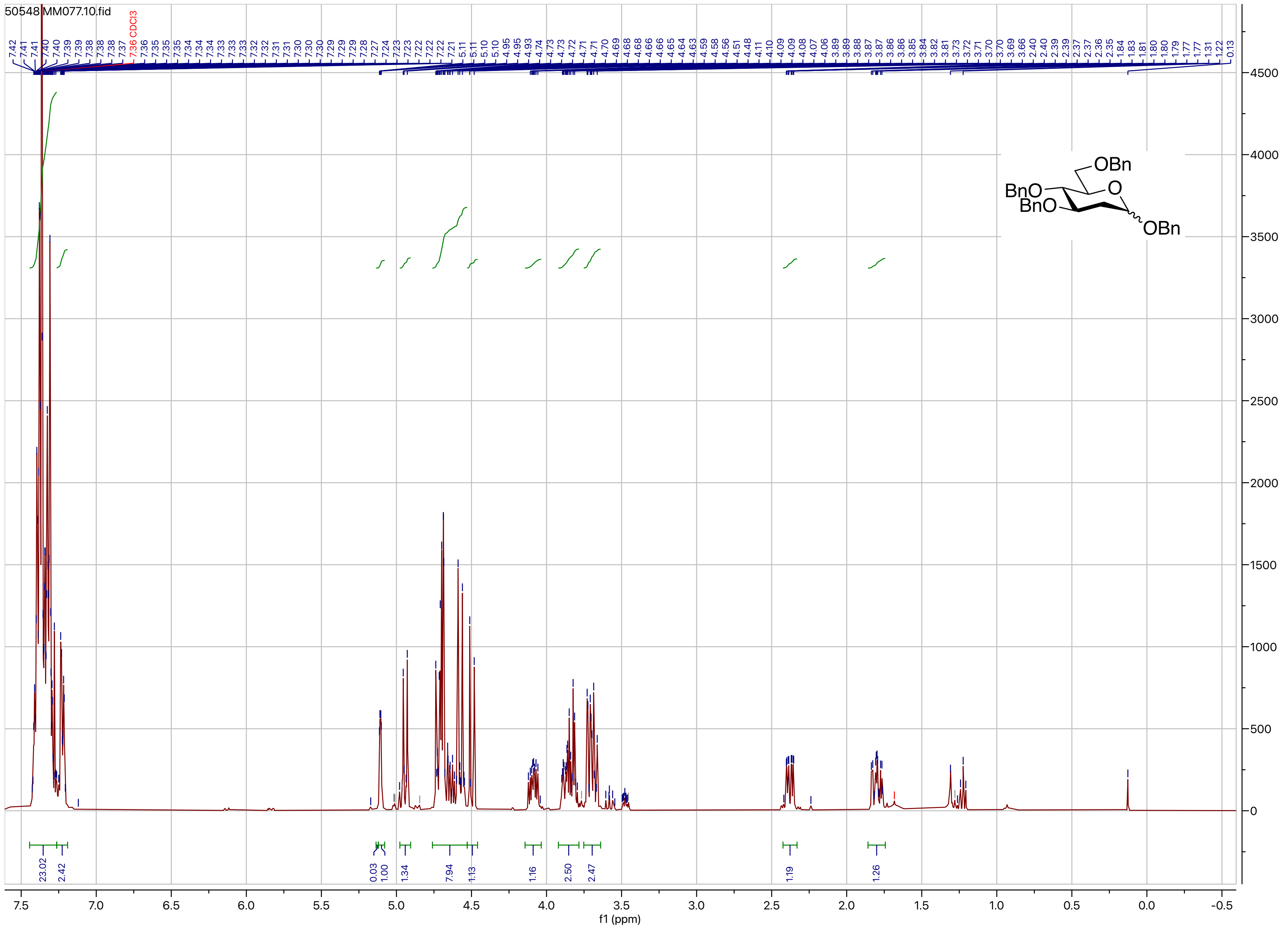

^{13}C Spectrum of 7f (101 MHz, Chloroform-*d*)

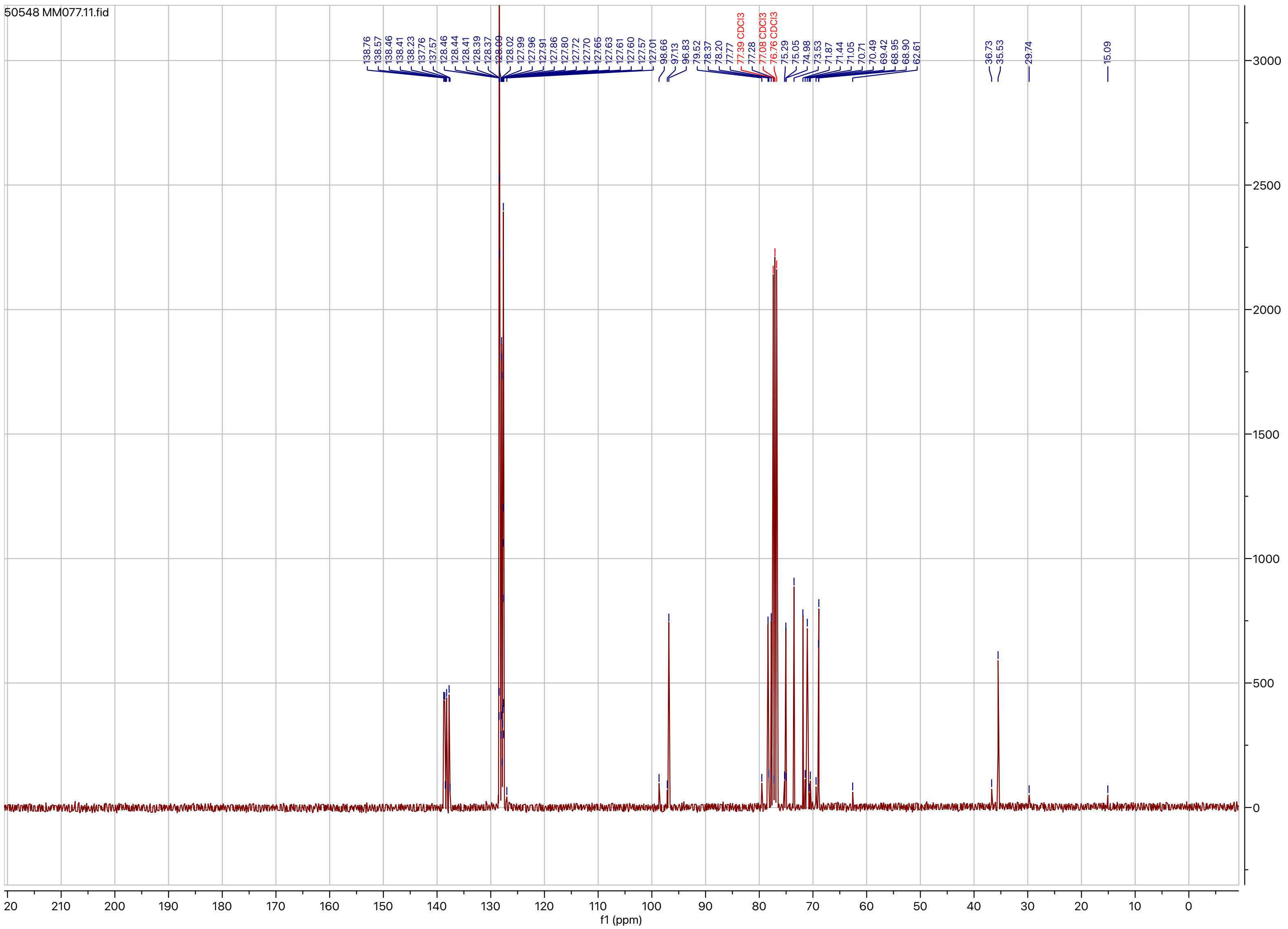

^{1}H Spectrum of 7g (400 MHz, Chloroform-*d*).

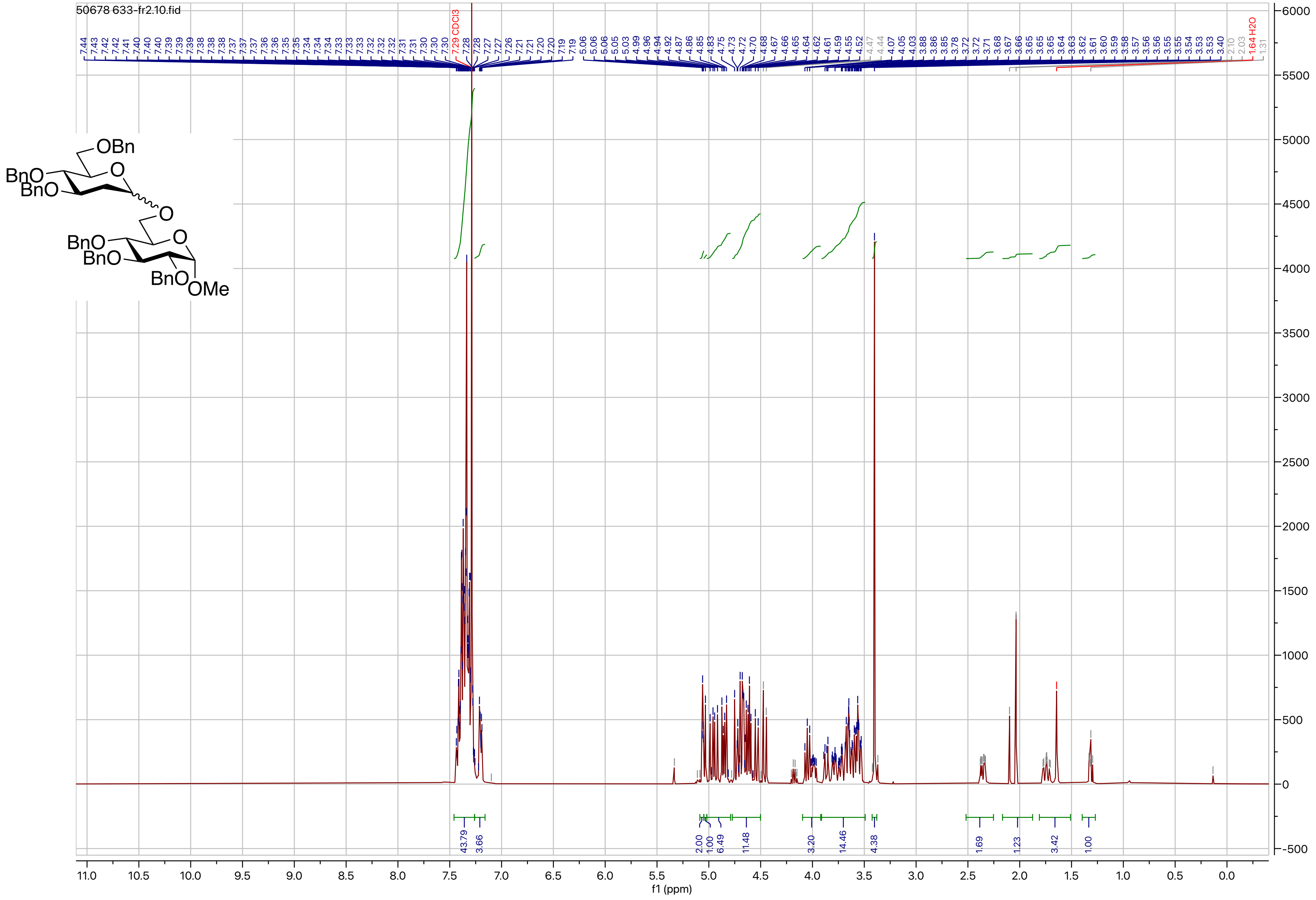

^{13}C Spectrum of 7g (101 MHz, Chloroform-*d*)

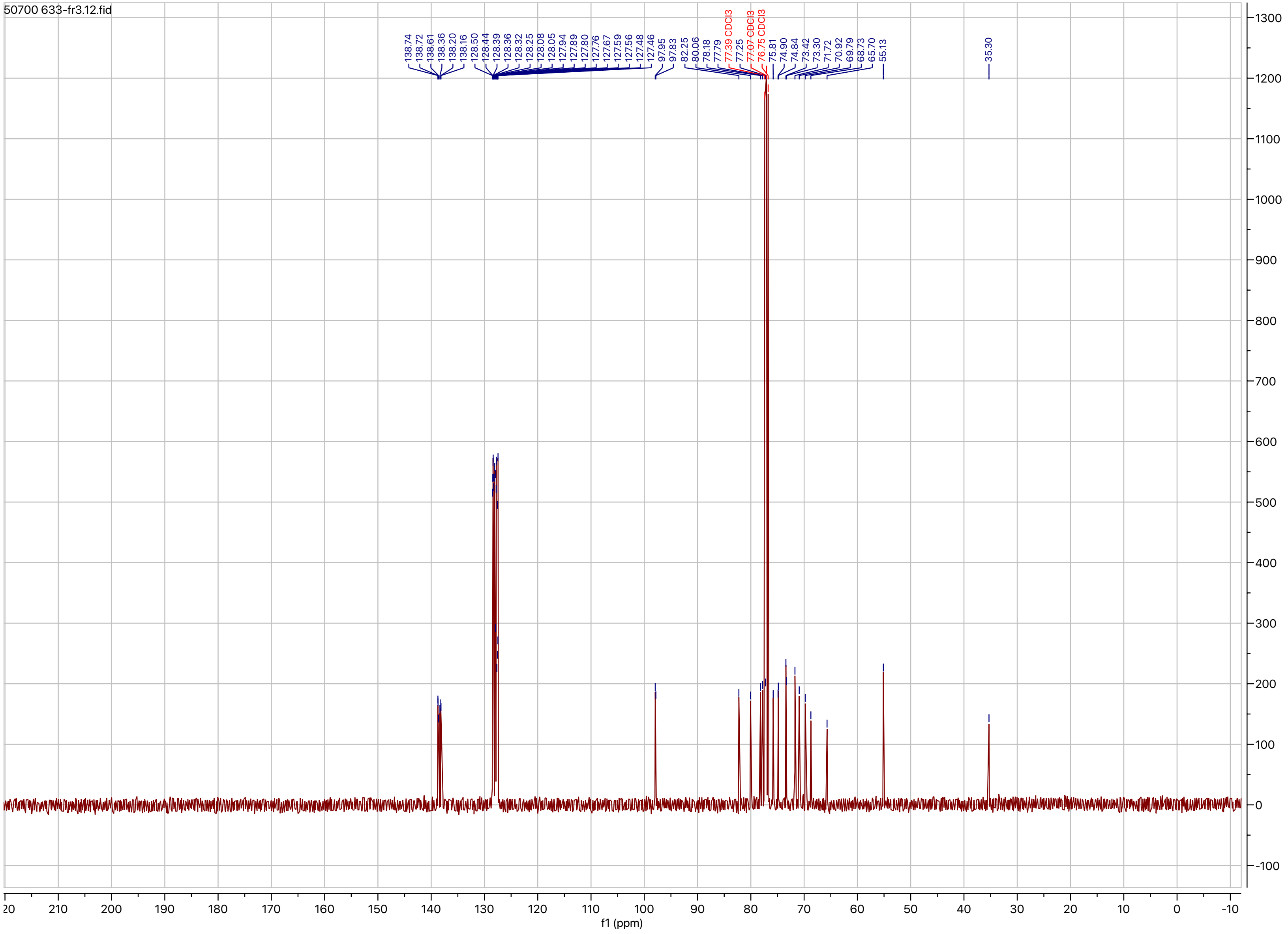

^{1}H Spectrum of 7h (400 MHz, Chloroform-*d*).

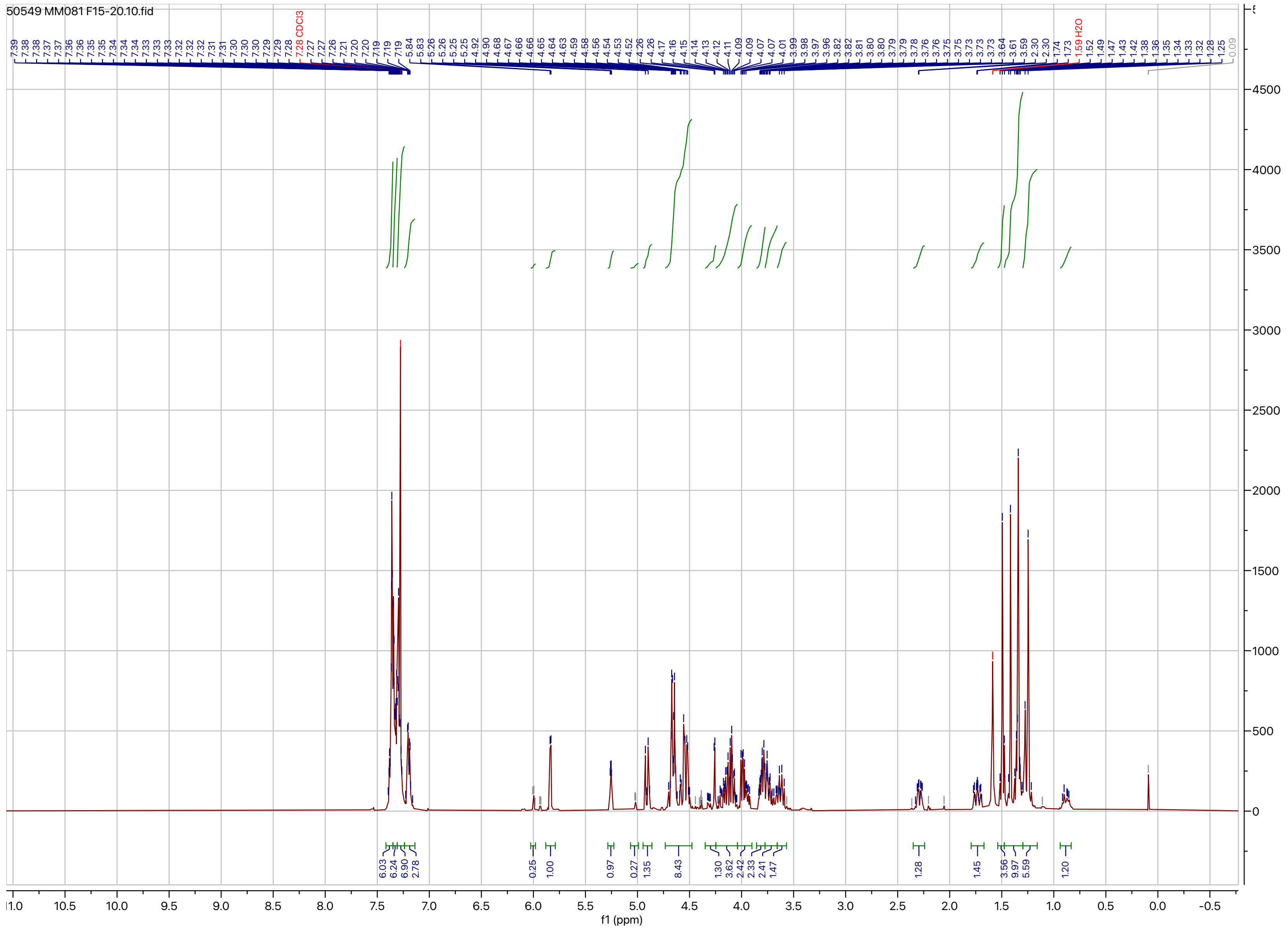

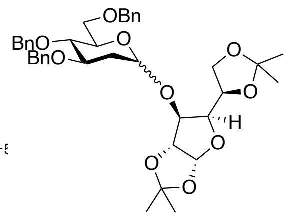

^{13}C Spectrum of 7h (101 MHz, Chloroform-*d*)

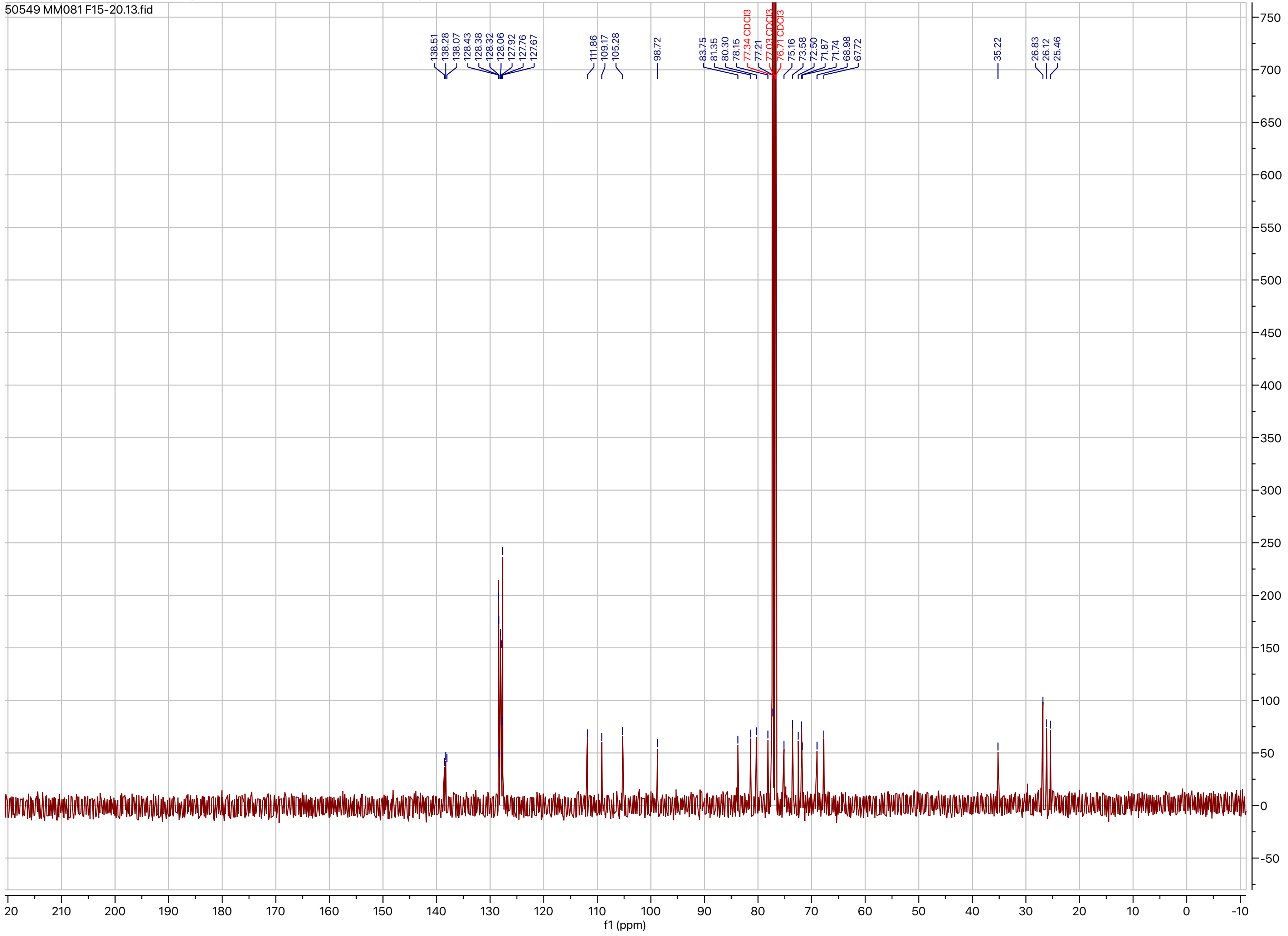

^{1}H Spectrum of 9 (400 MHz, Chloroform-*d*)

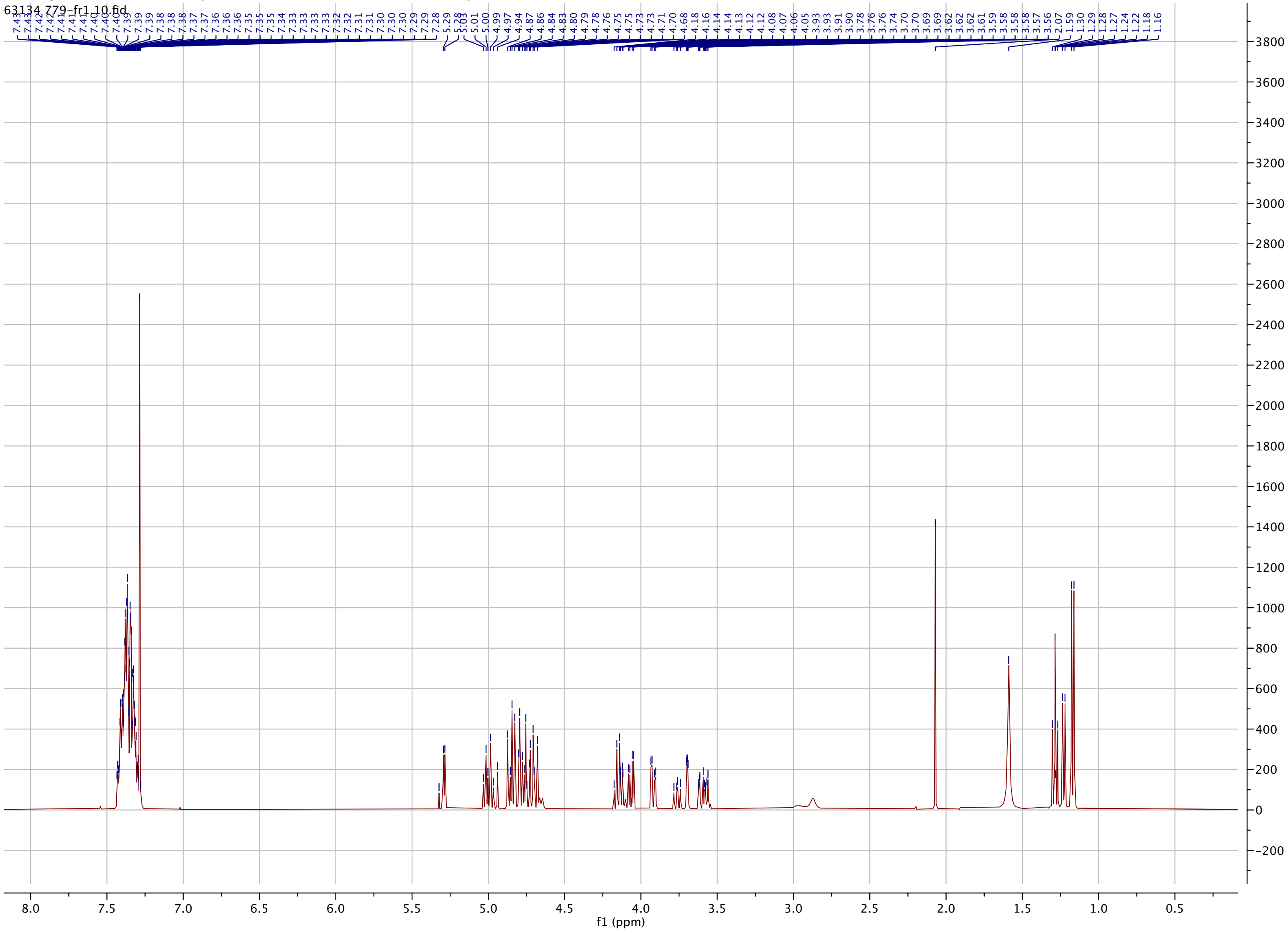

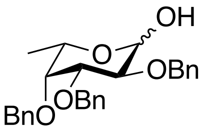

^{1}H Spectrum of 10 (400 MHz, Chloroform-*d*).

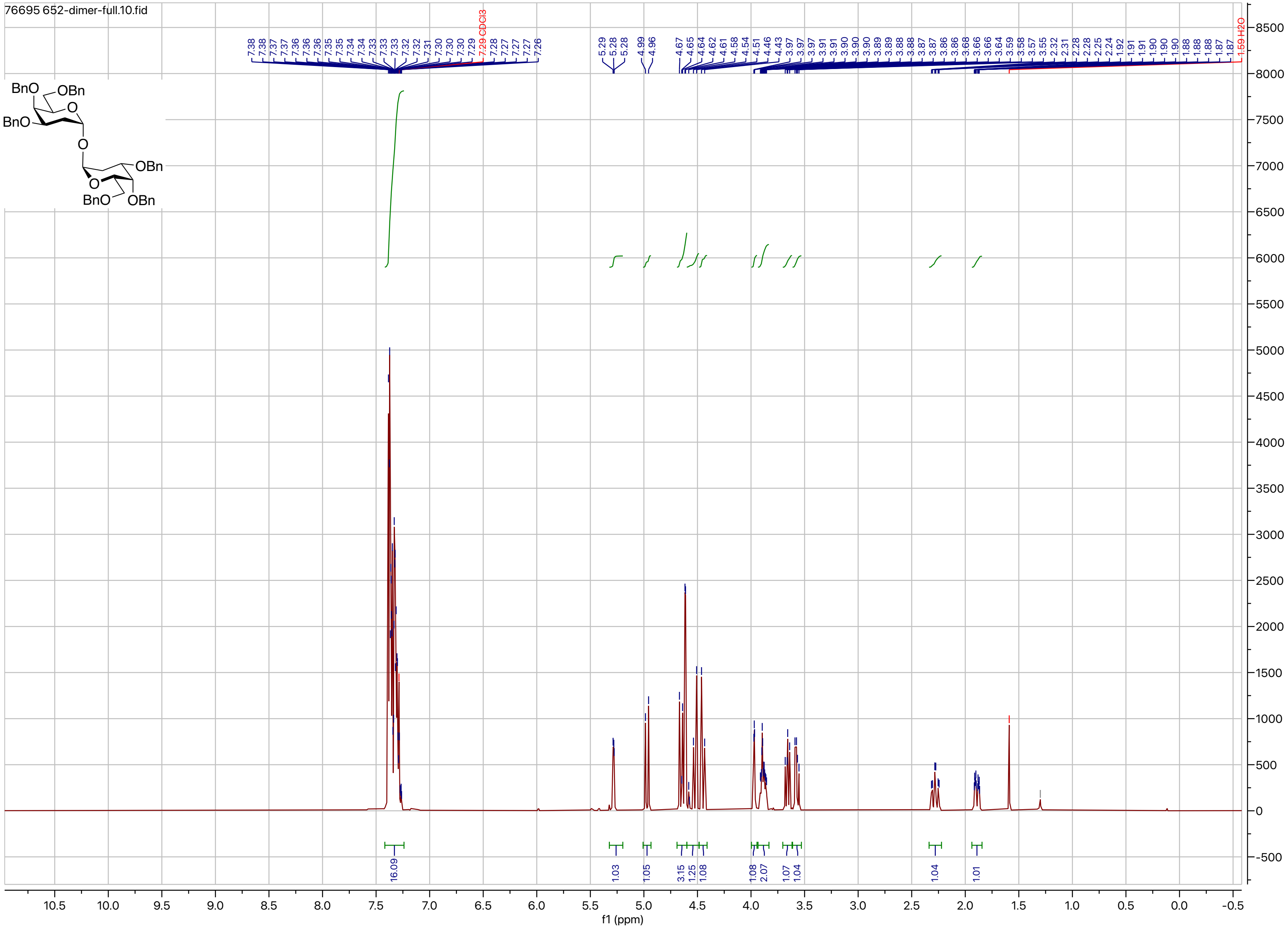

^{13}C Spectrum of 10 (101 MHz, Chloroform-*d*)

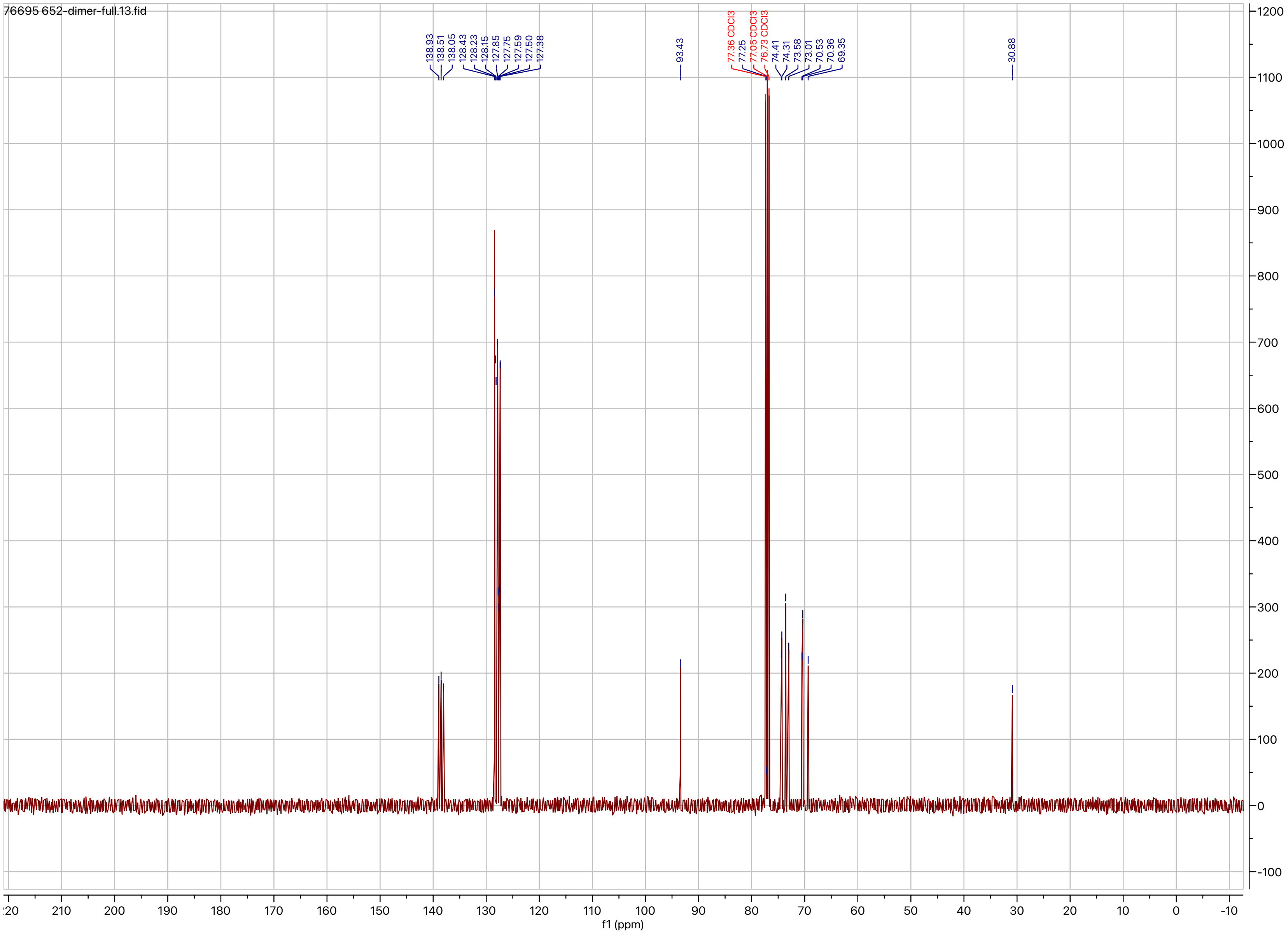

^{1}H Spectrum of 11 (400 MHz, Chloroform-*d*).

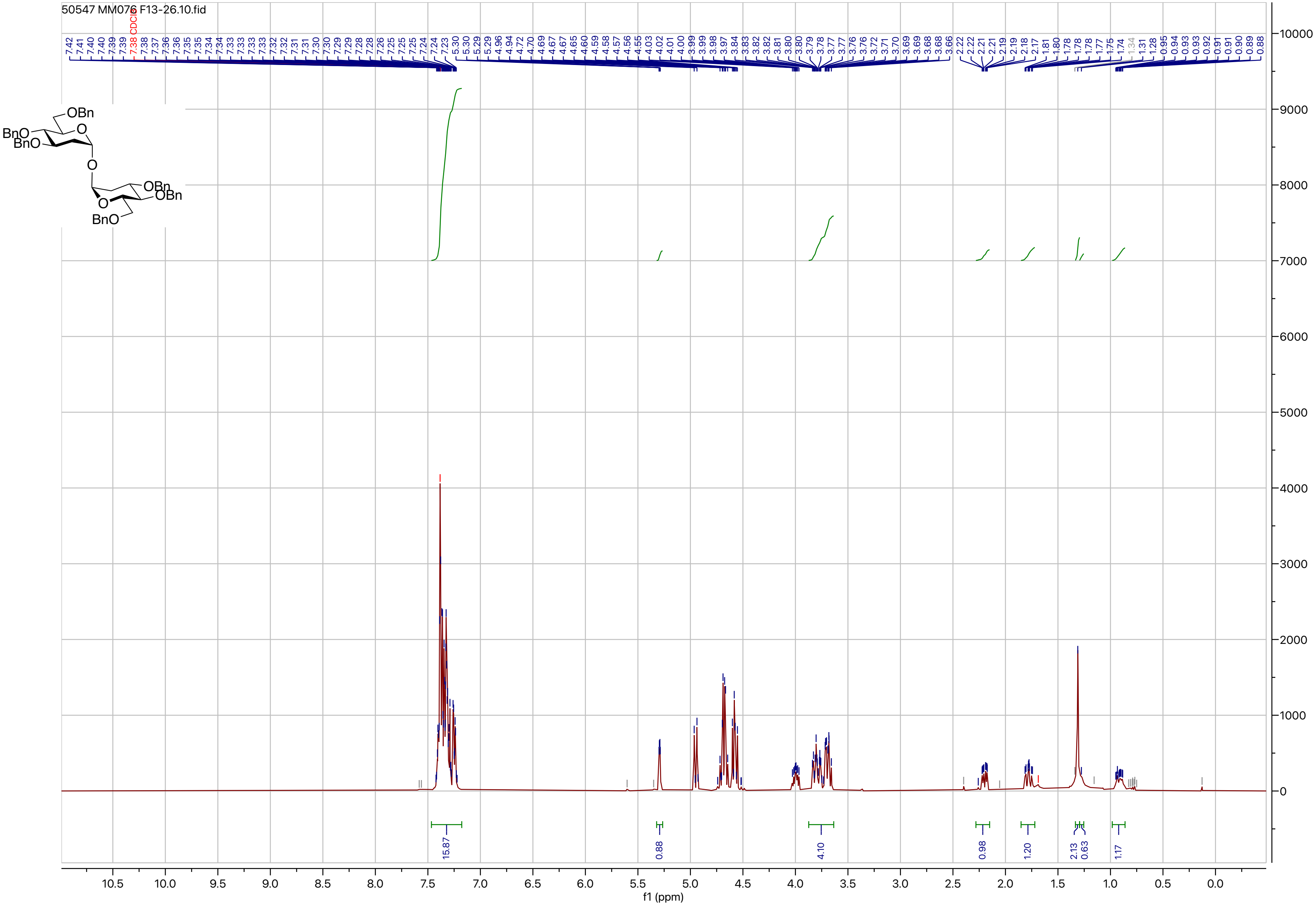

^{13}C Spectrum of 11 (101 MHz, Chloroform-*d*)

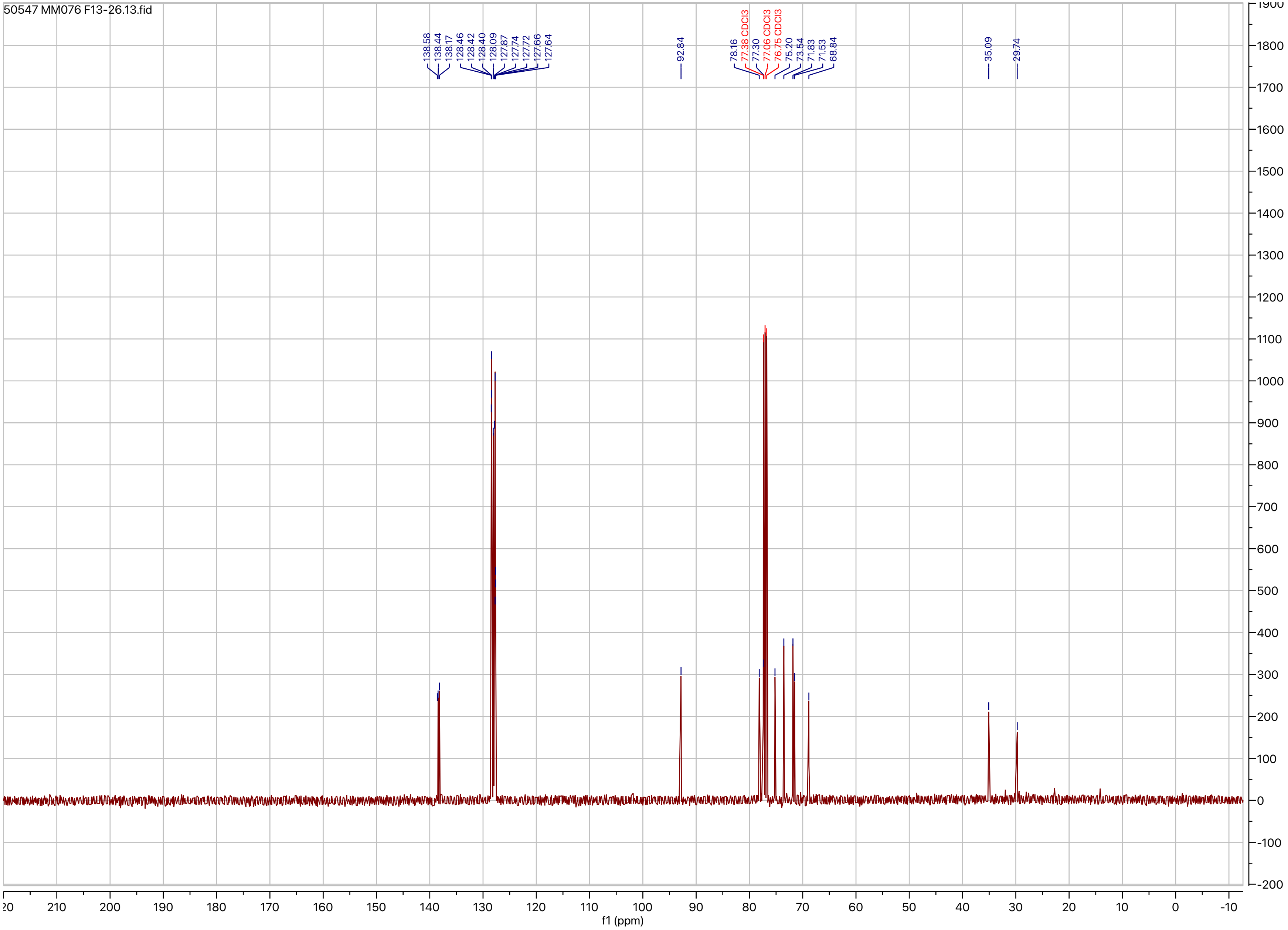

^{1}H Spectrum of 12 (400 MHz, Chloroform-*d*)

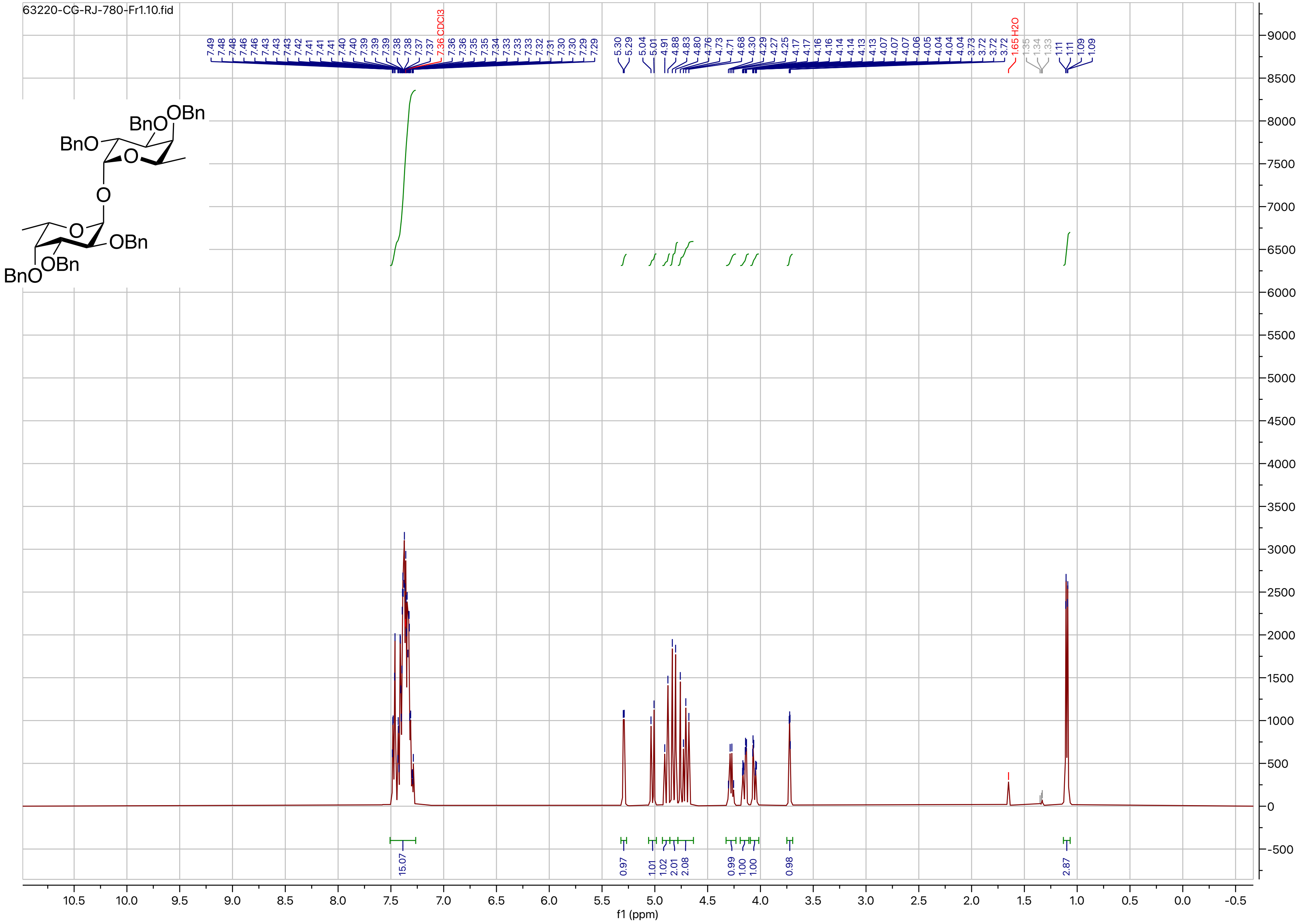

^{13}C Spectrum of 12 (101 MHz, Chloroform-*d*)

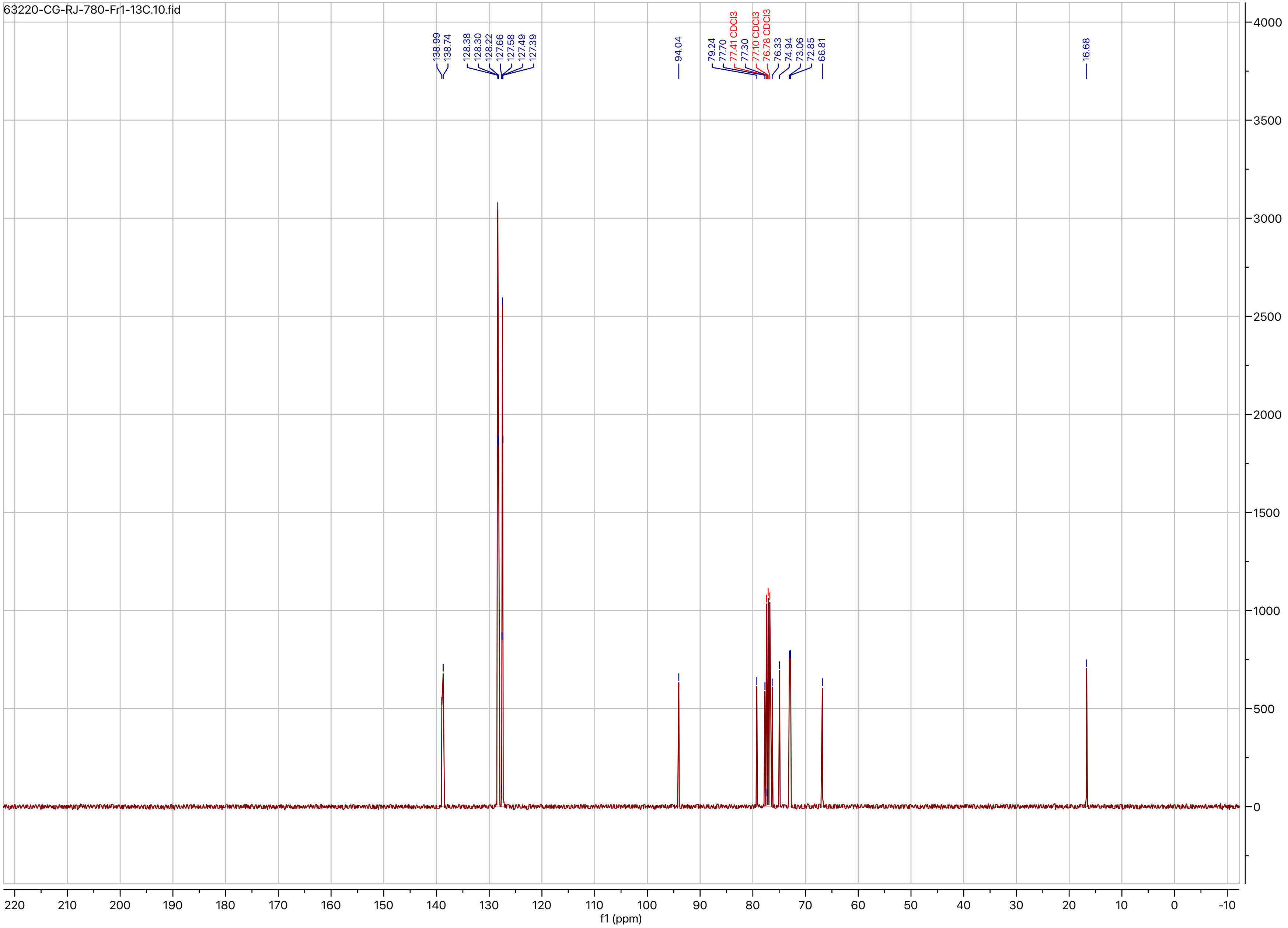

[1]H Spectrum of α-S11 (400 MHz, Chloroform-*d*)

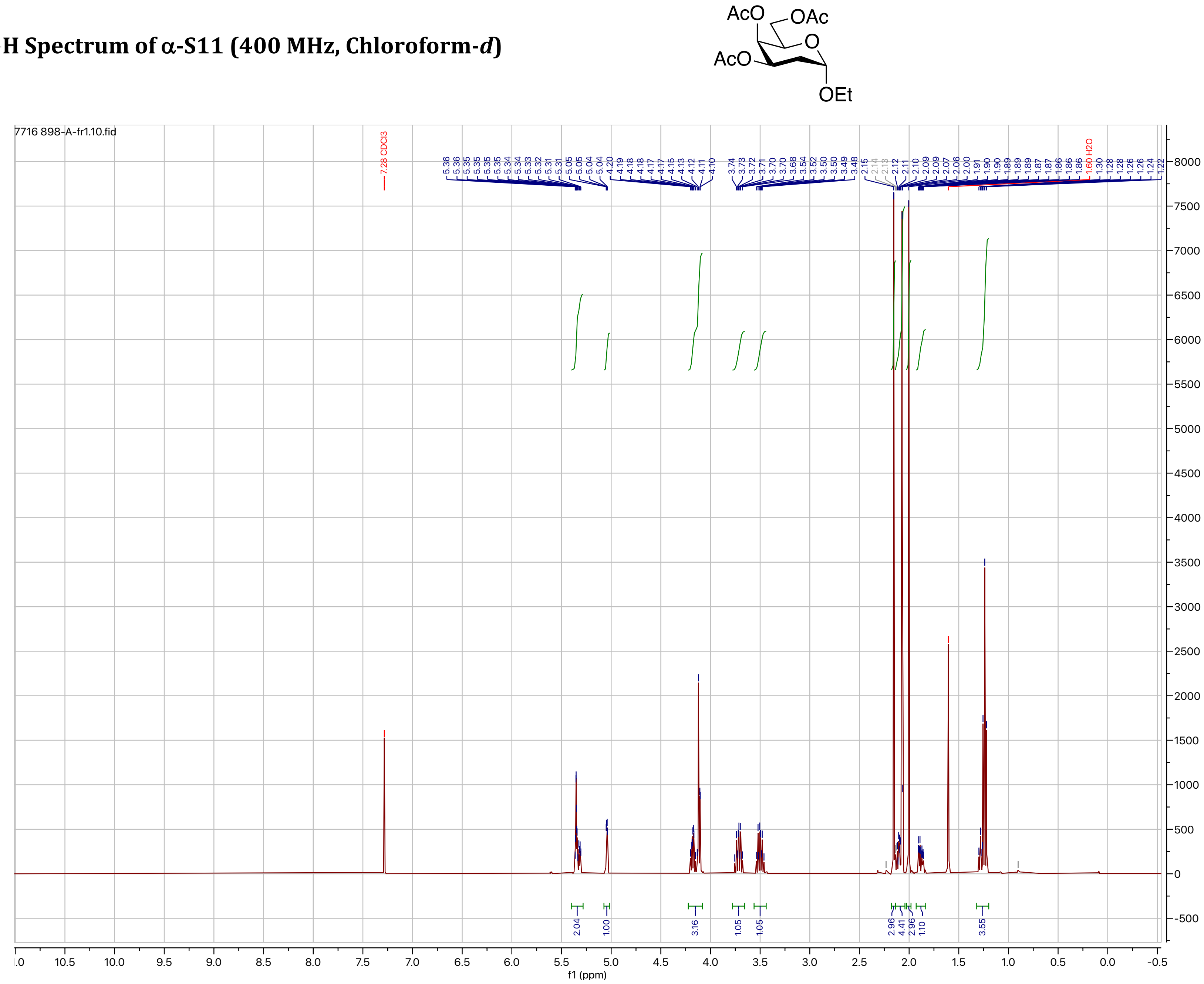

[13]C Spectrum of α-S11 (101 MHz, Chloroform-*d*)

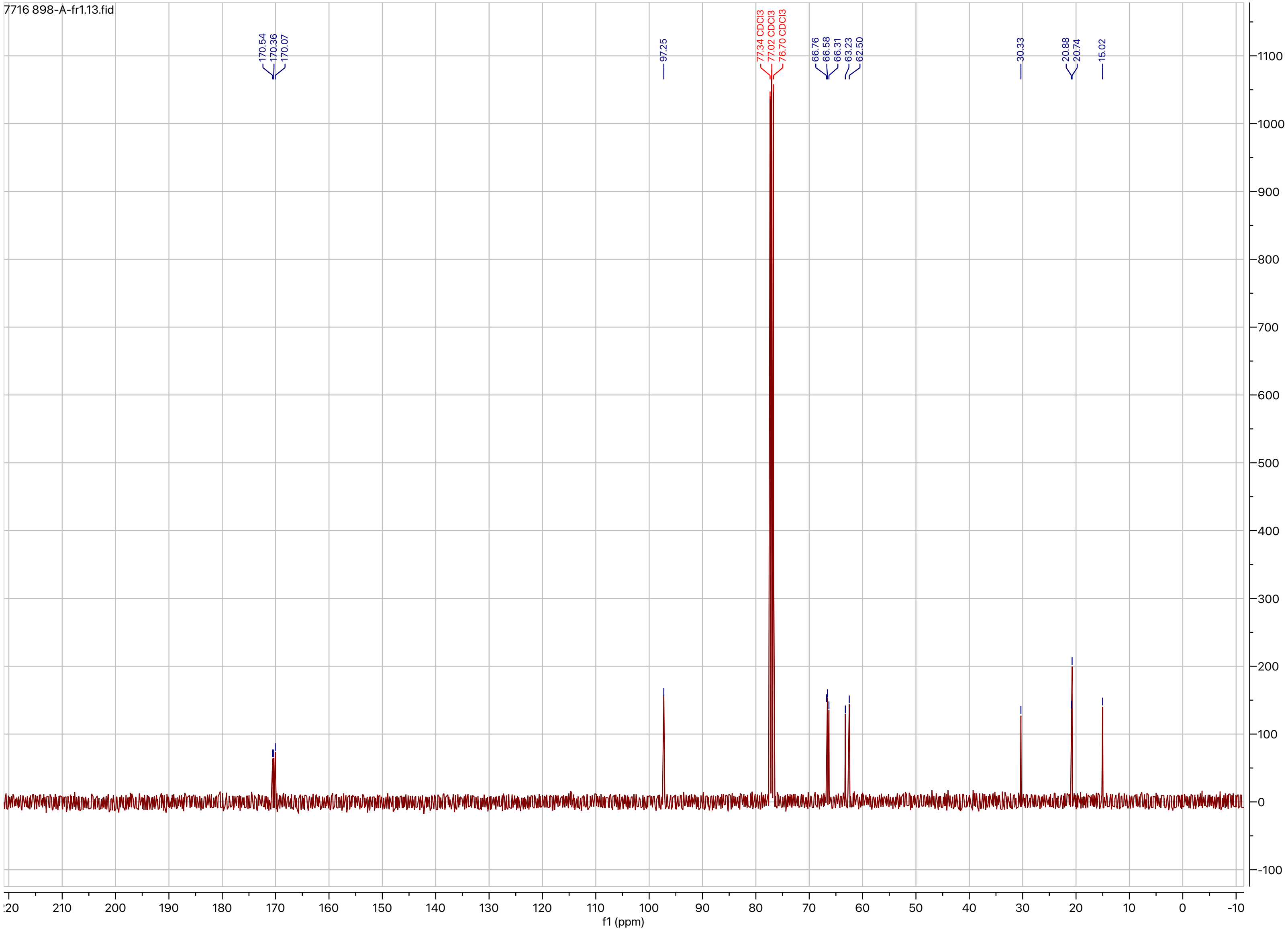

[1]H Spectrum of β-S11 (400 MHz, Chloroform-*d*).

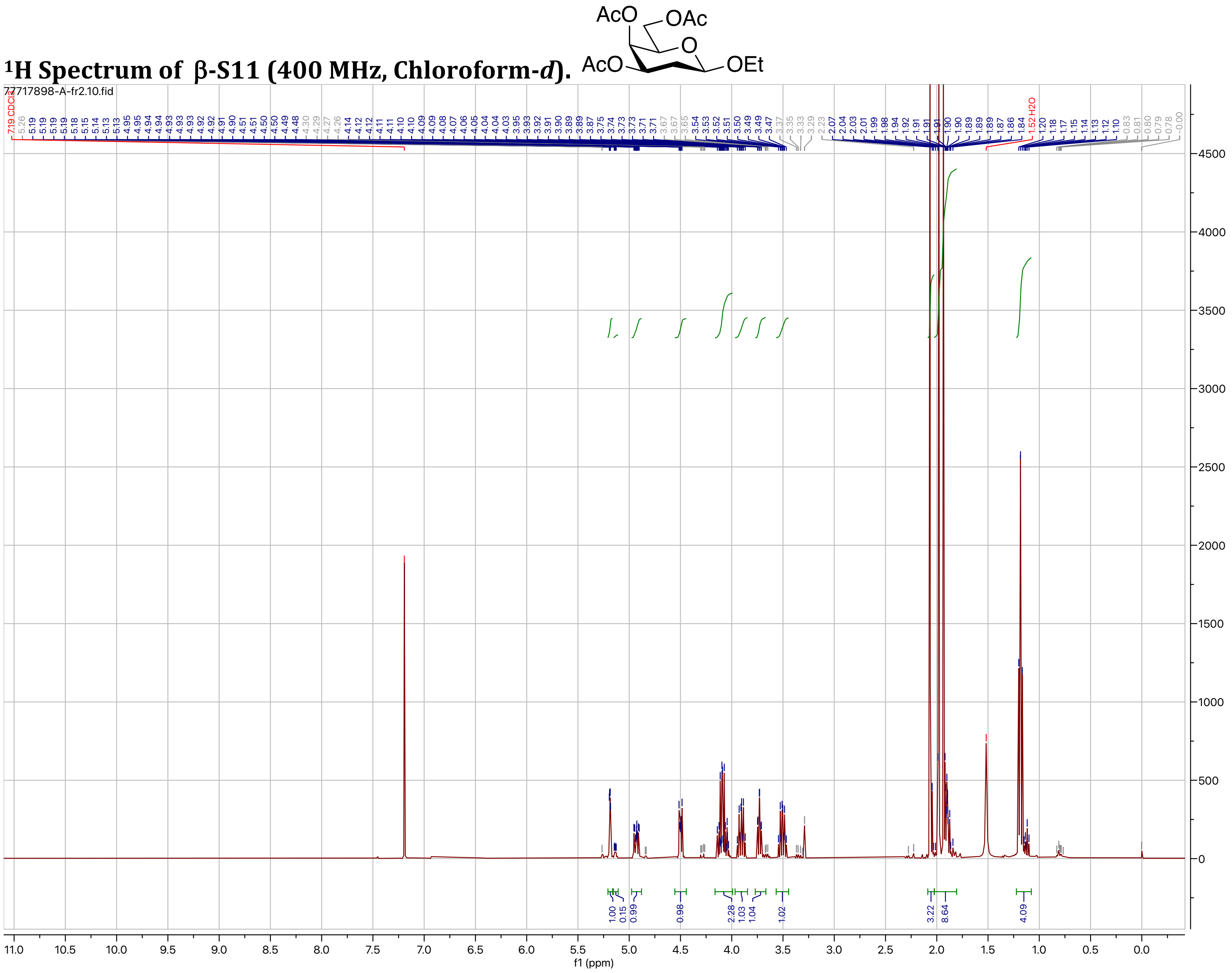

[13]C Spectrum of β-S11 (101 MHz, Chloroform-*d*)

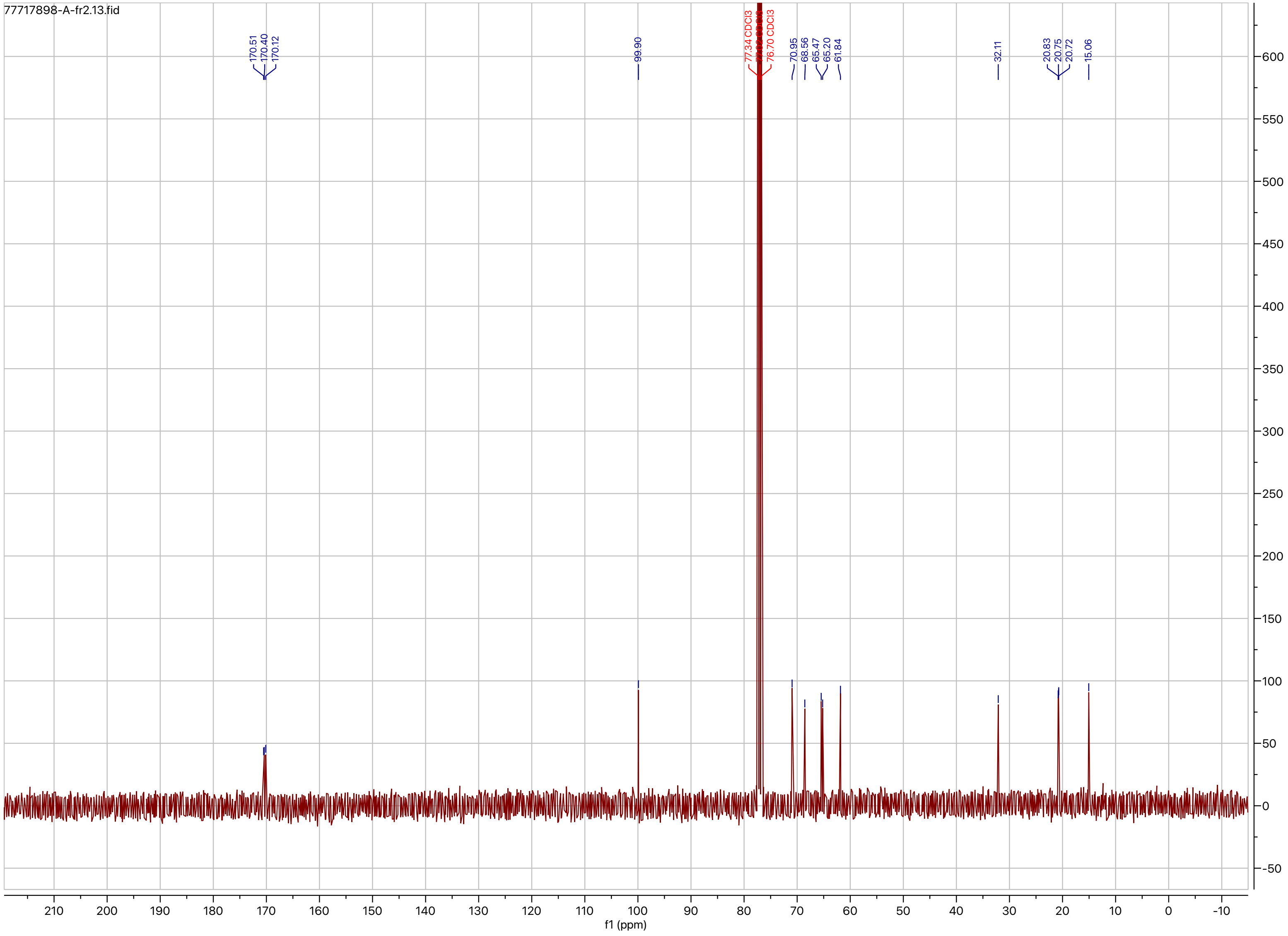

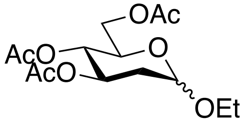

^{1}H Spectrum of S12 (400 MHz, Chloroform-*d*)

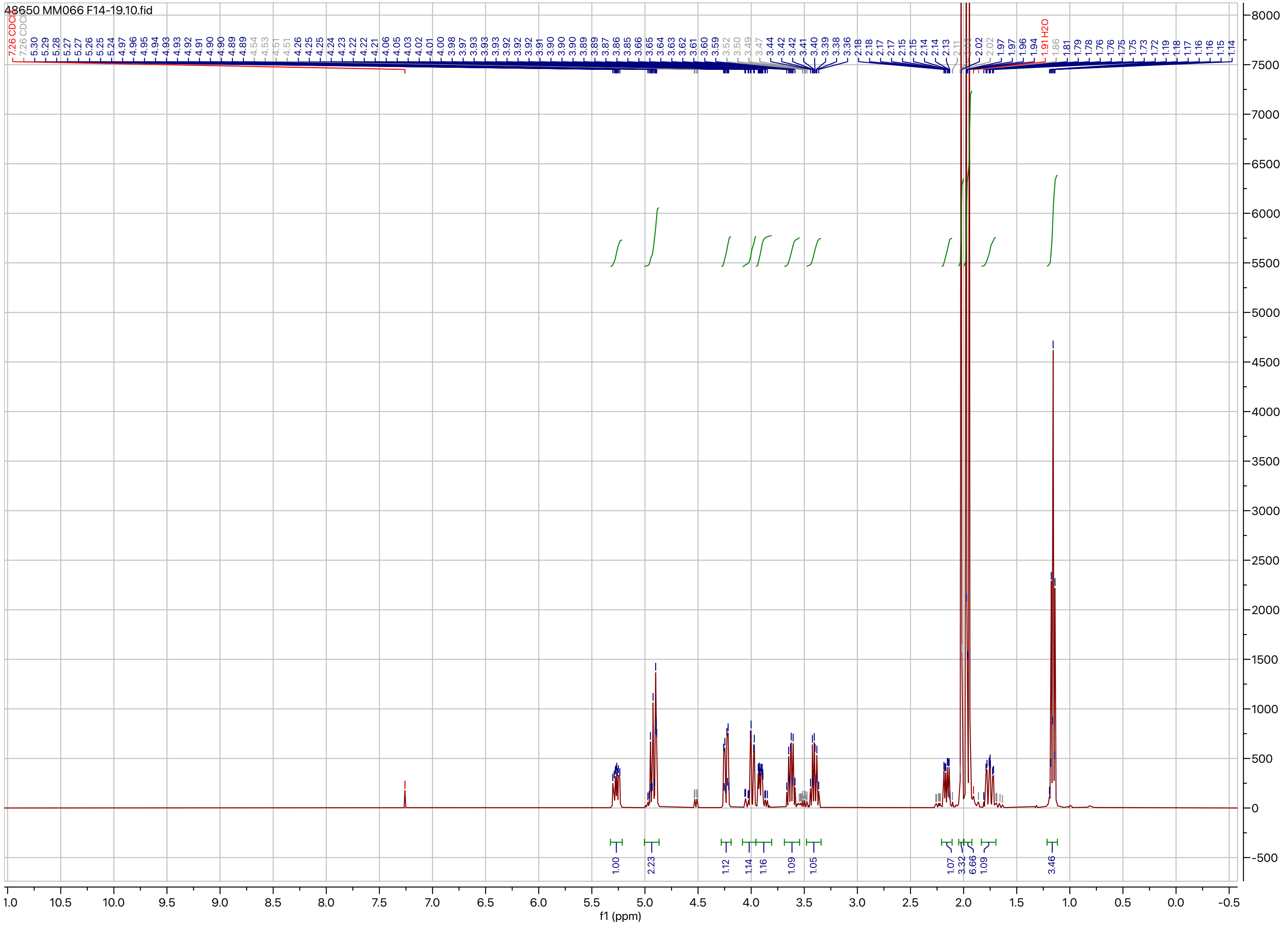

^{13}C Spectrum of S12 (101 MHz, Chloroform-*d*)

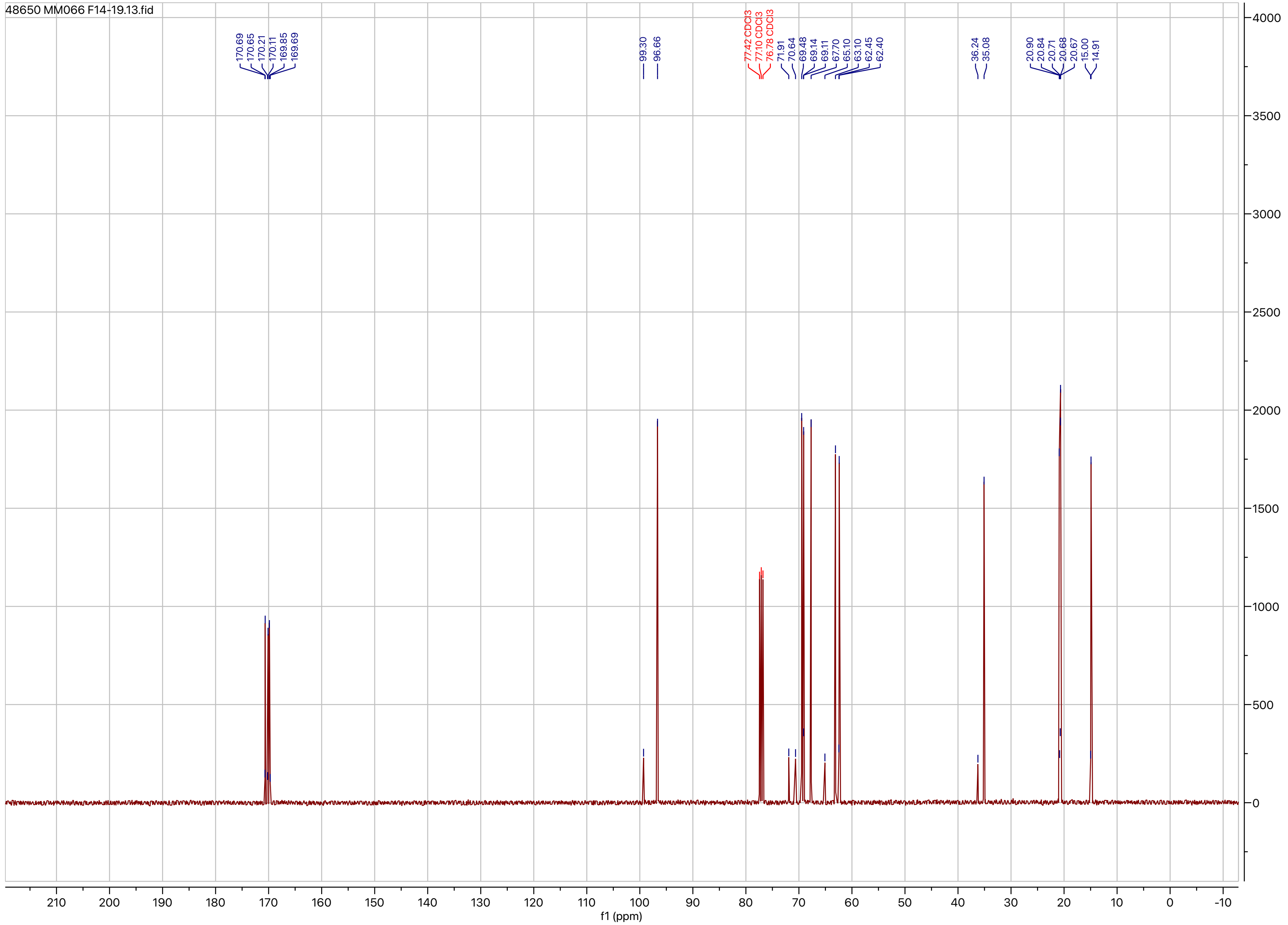

[1]H Spectrum of 15a (400 MHz, Chloroform-*d*).

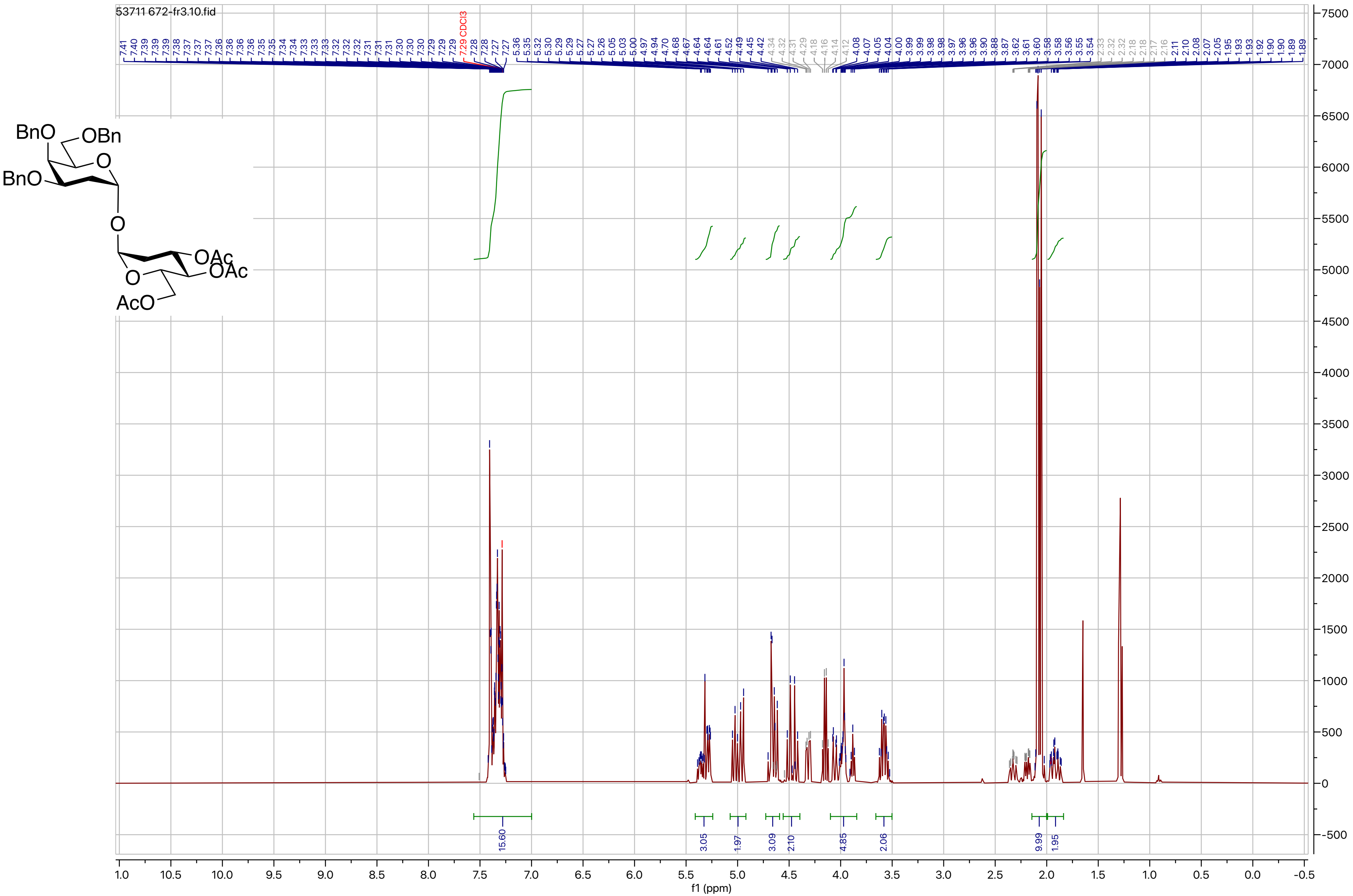

^{13}C Spectrum of 15a (101 MHz, Chloroform-*d*)

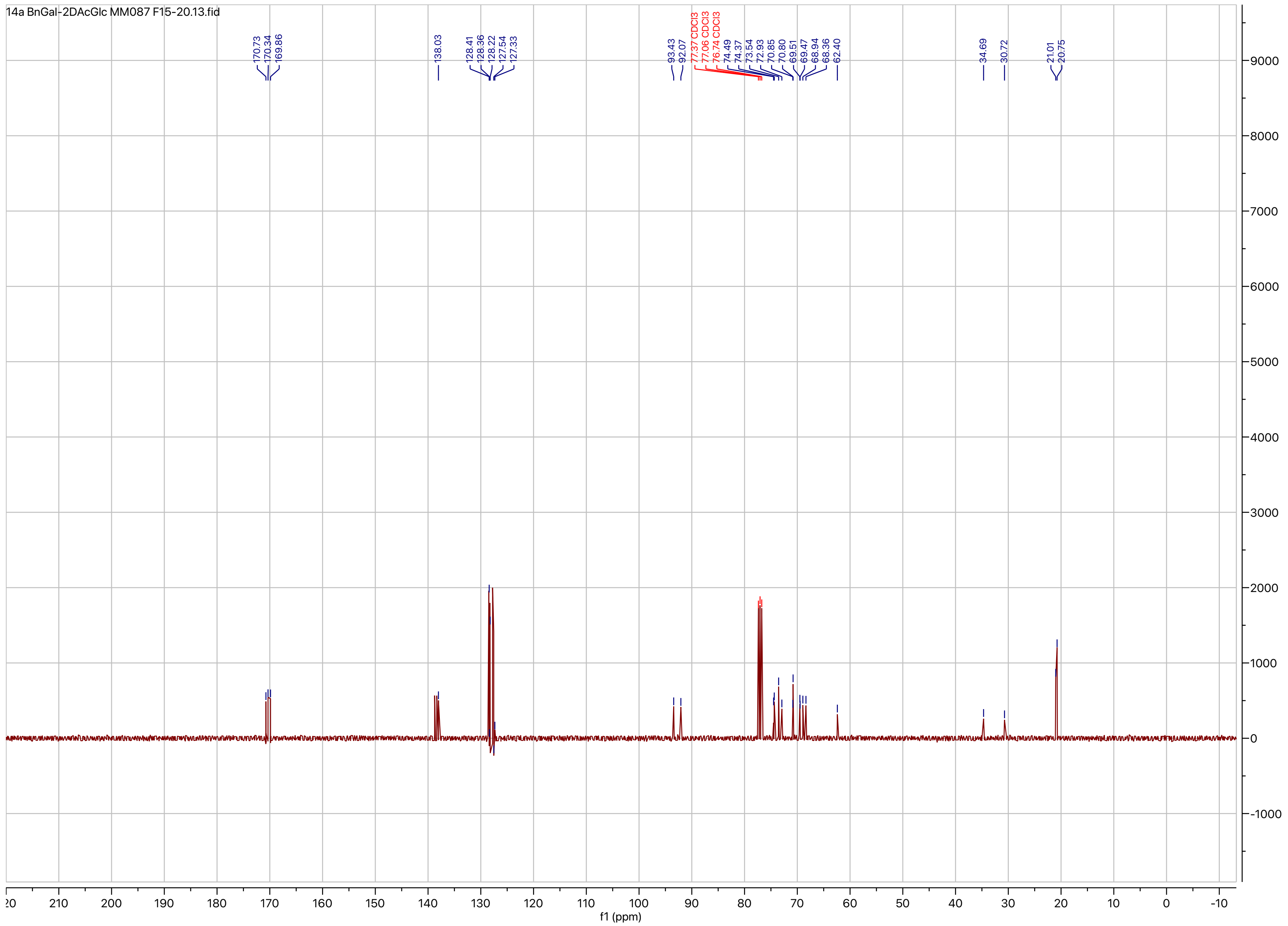

[1]H Spectrum of 15b (400 MHz, Chloroform-*d*).

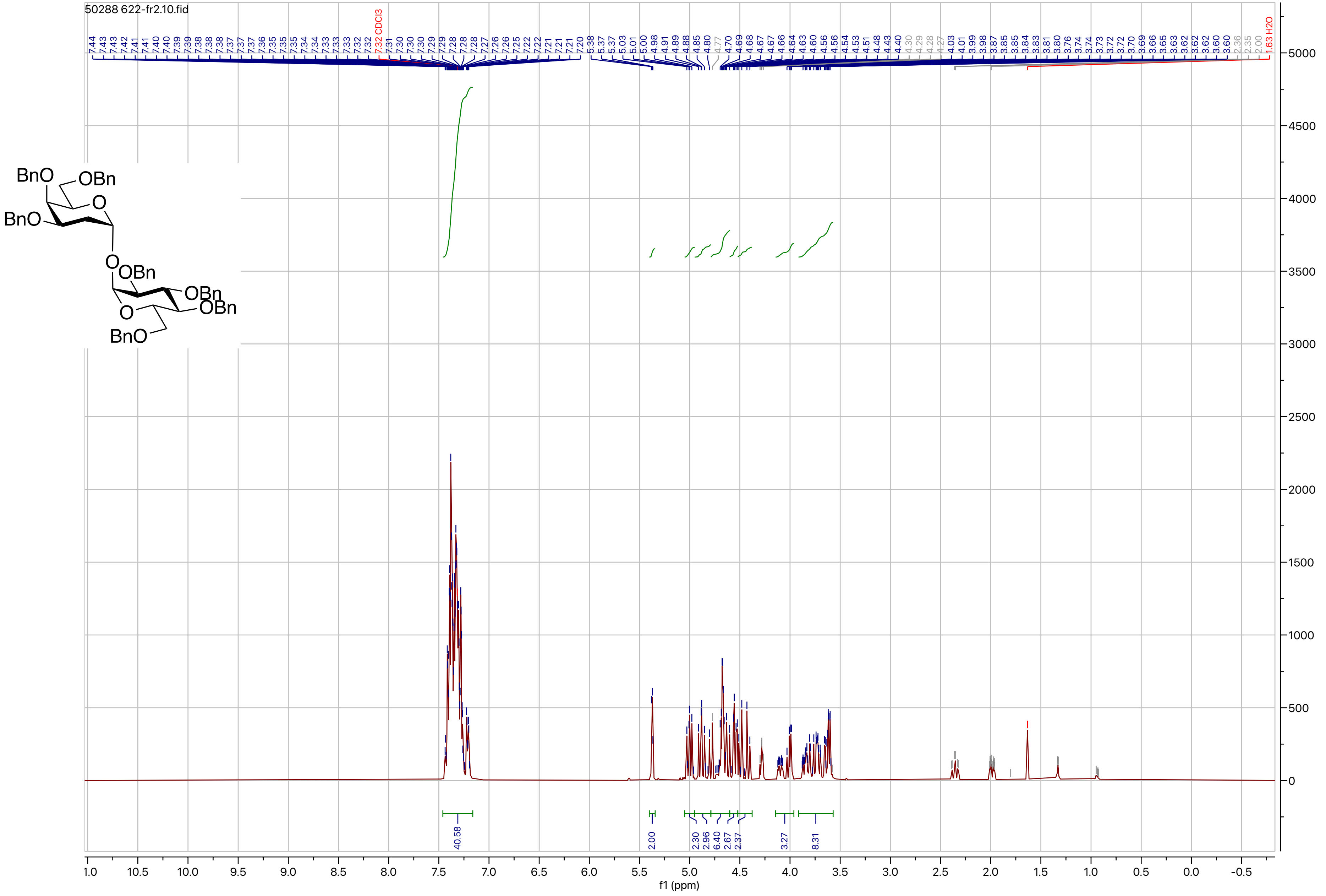

^{13}C Spectrum of 15b (101 MHz, Chloroform-*d*)

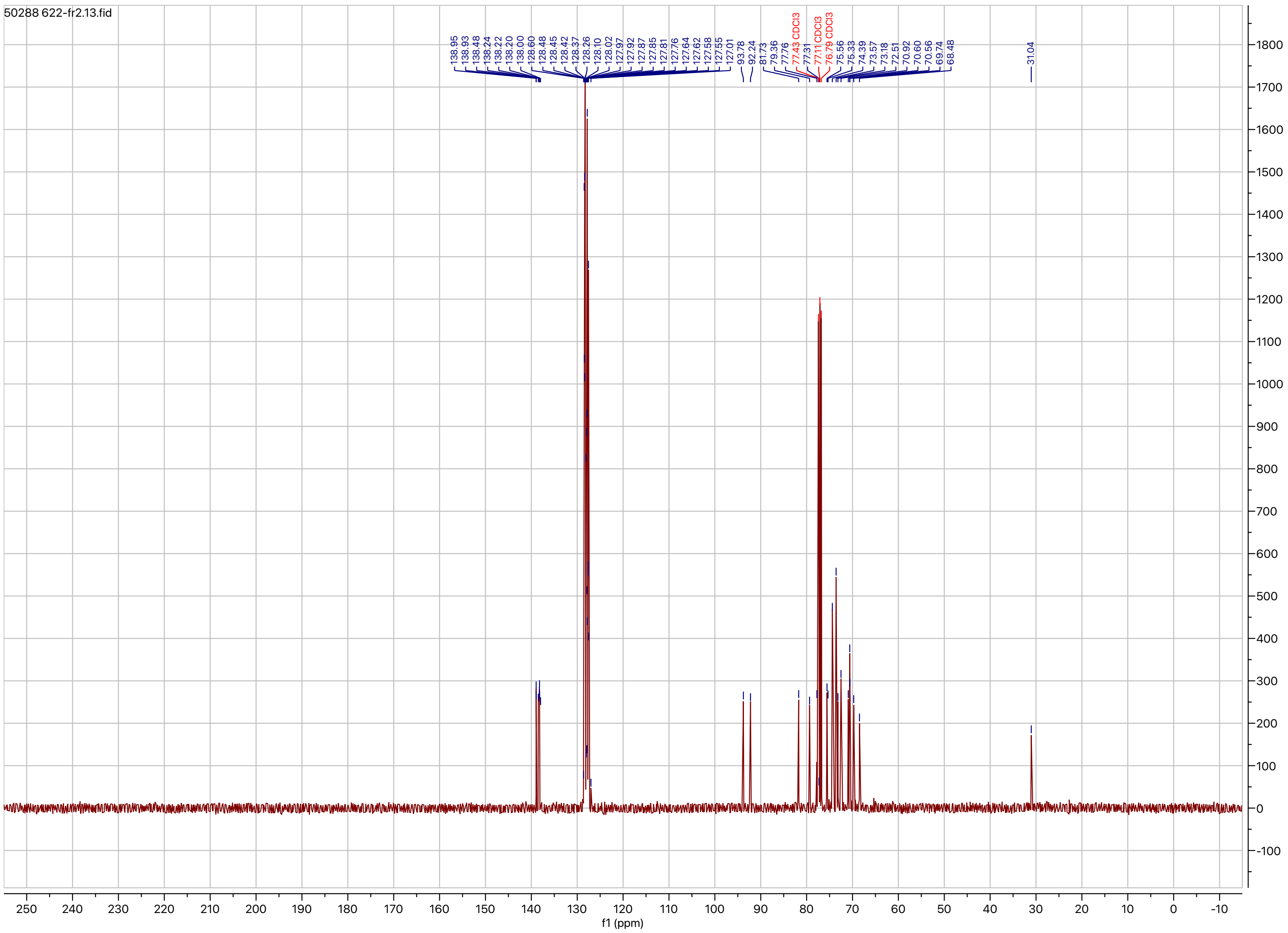

[1]H Spectrum of 15c (400 MHz, Chloroform-*d*)

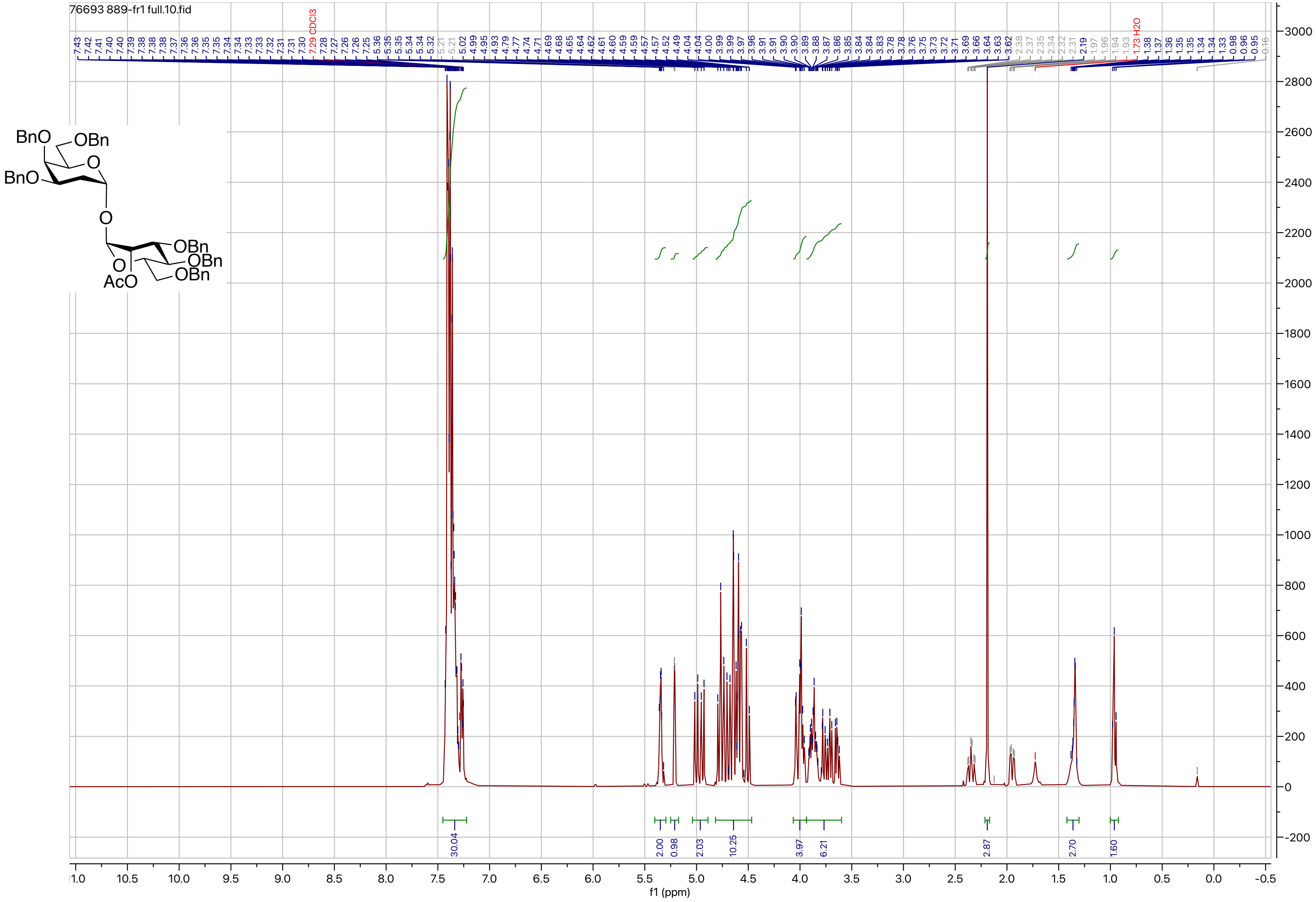

^{13}C Spectrum of 15c (101 MHz, Chloroform-*d*)

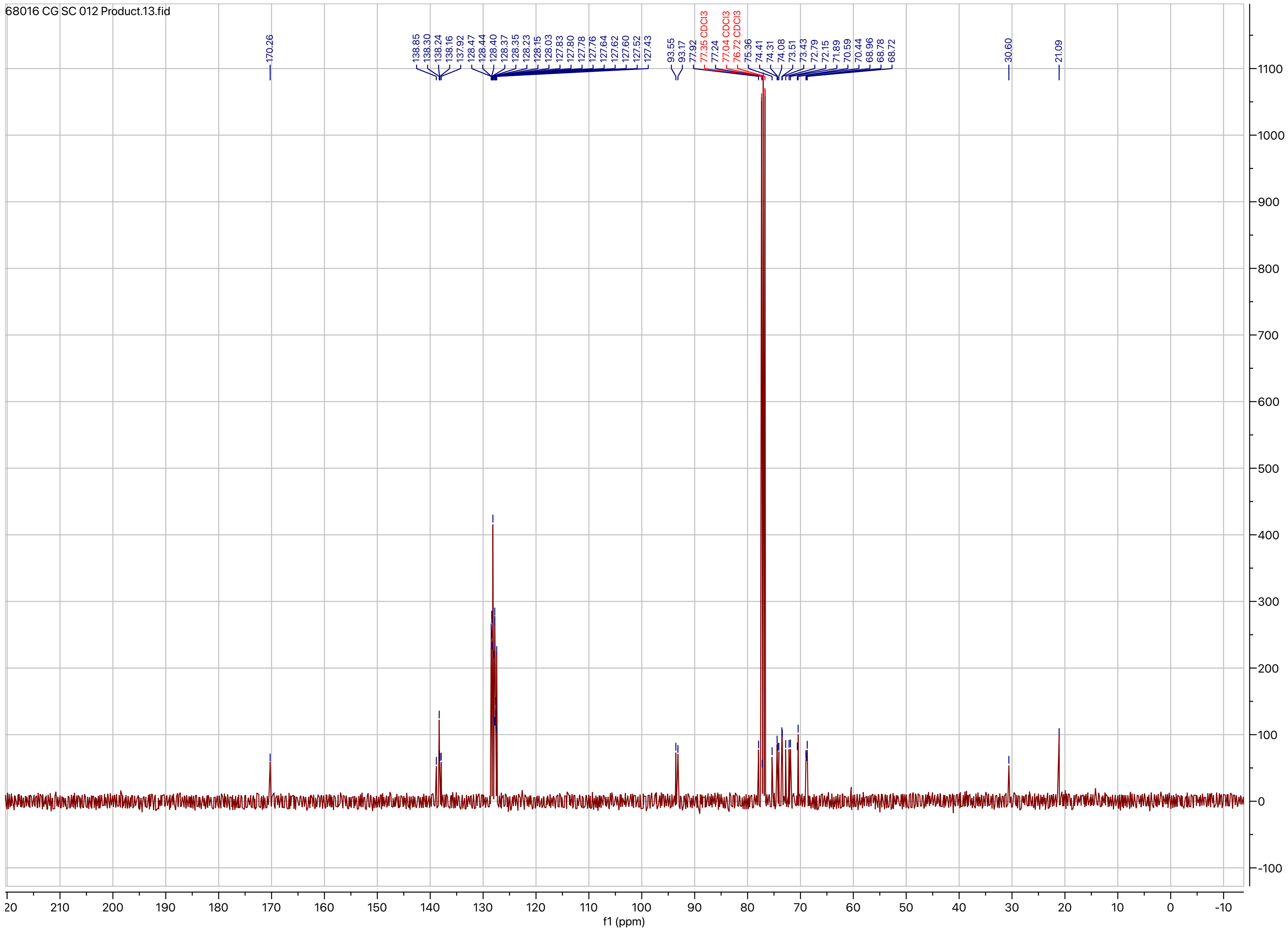

[1]H Spectrum of 15d (400 MHz, Chloroform-*d*)

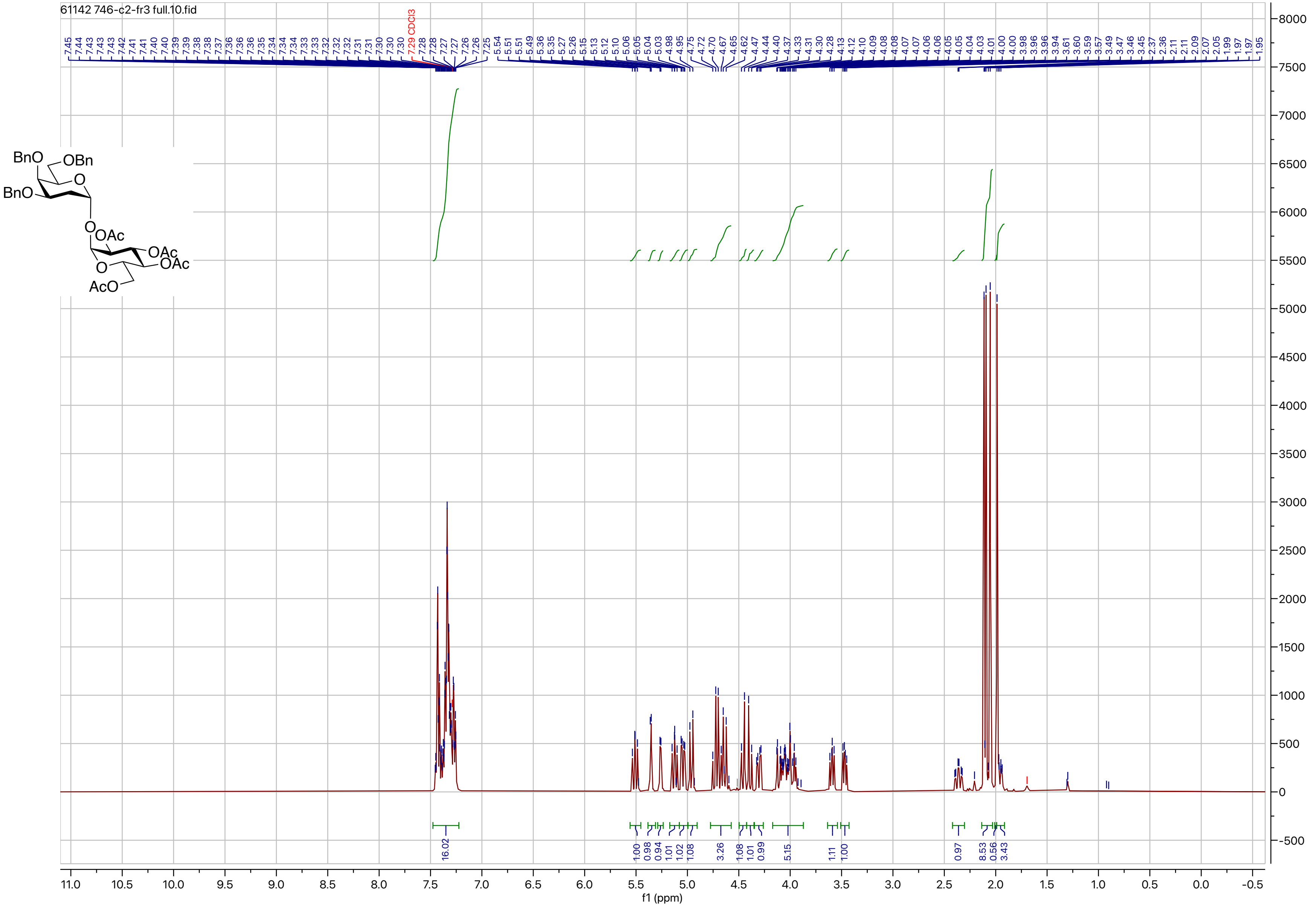

^{13}C Spectrum of 15d (101 MHz, Chloroform-*d*)

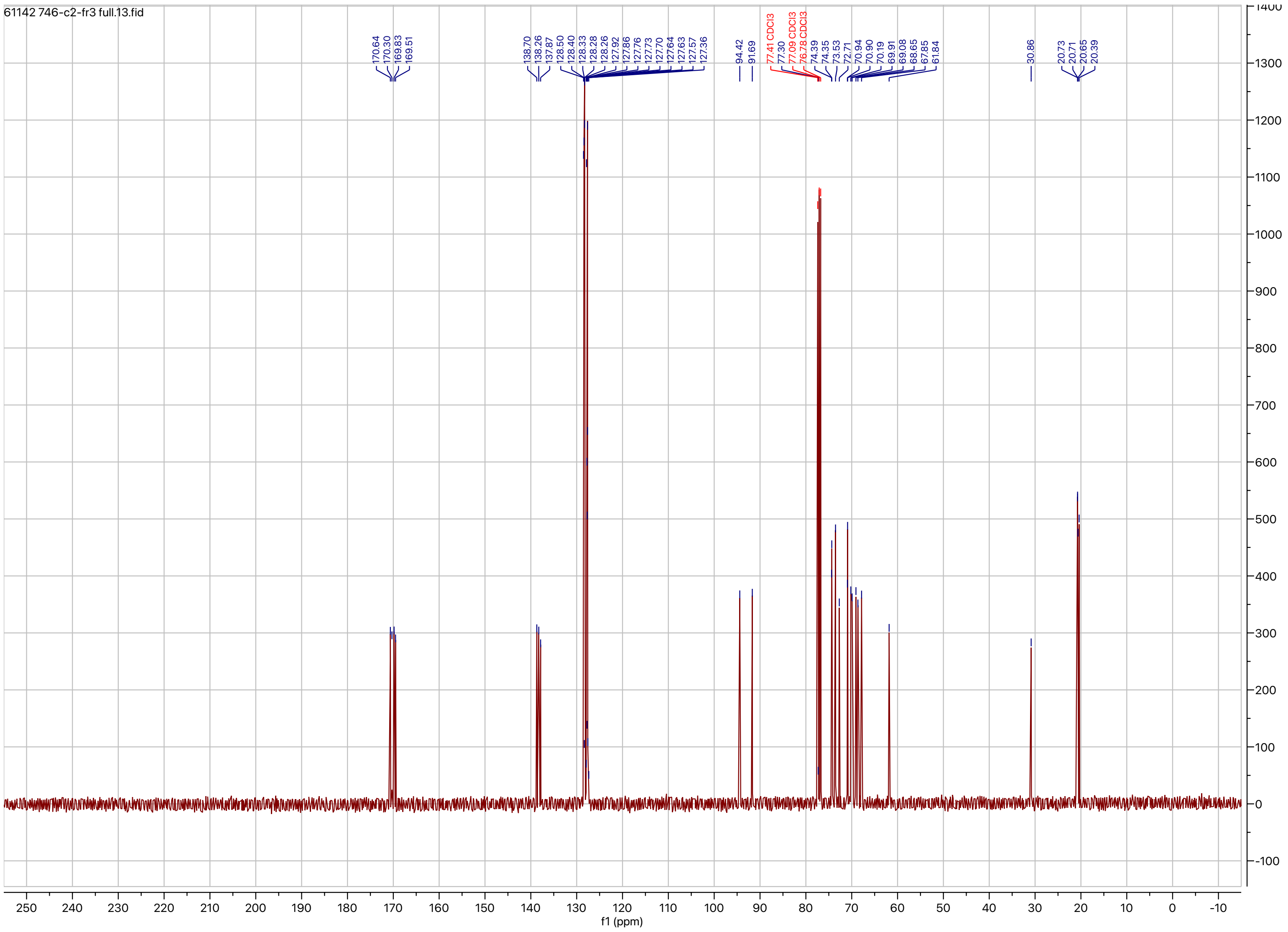

[1]H Spectrum of 15e (400 MHz, Chloroform-*d*).

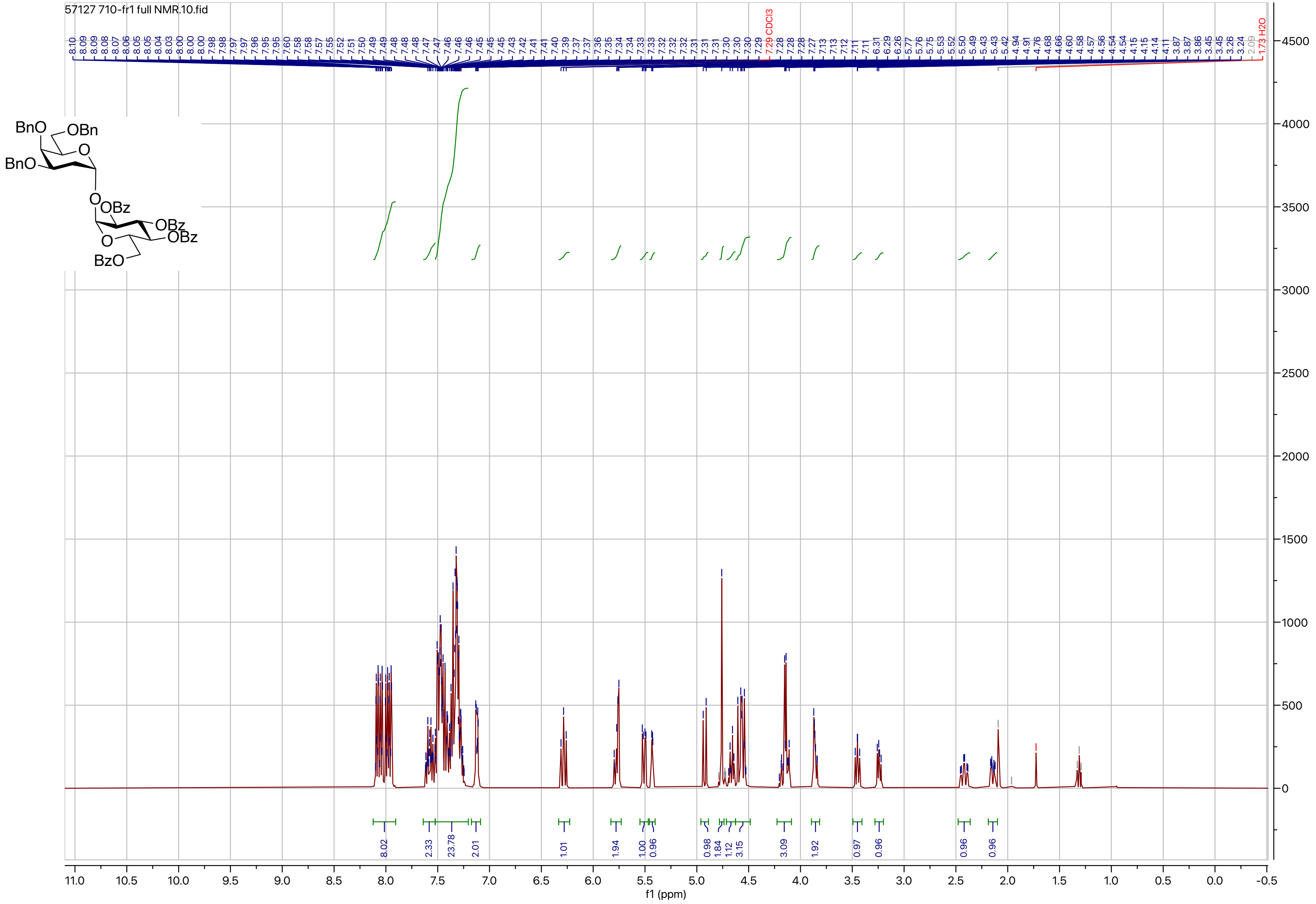

^{13}C Spectrum of 15e (101 MHz, Chloroform-*d*)

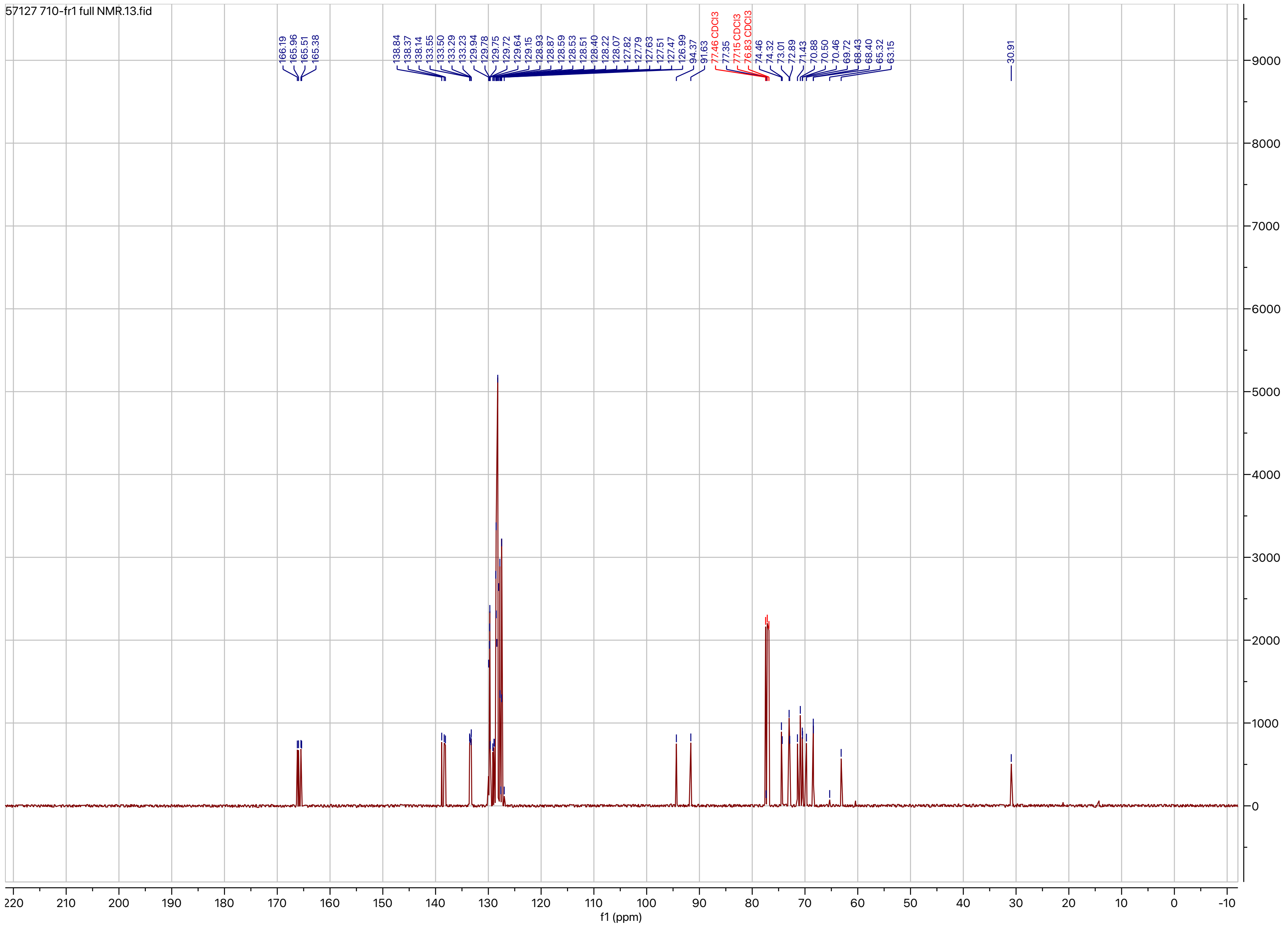

[1]H Spectrum of 15f (400 MHz, Chloroform-*d*)

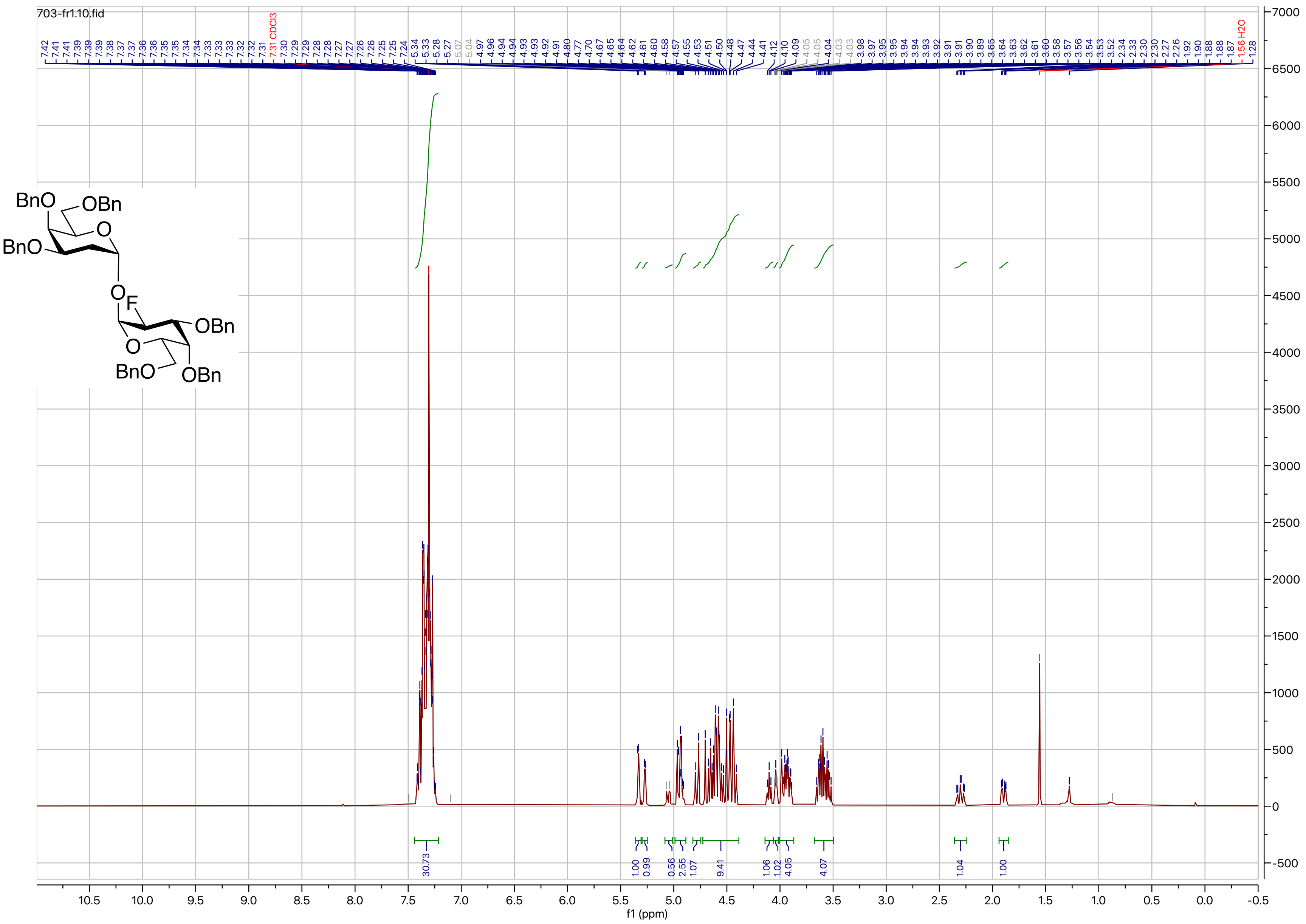

^{13}C Spectrum of 15f (101 MHz, Chloroform-*d*)

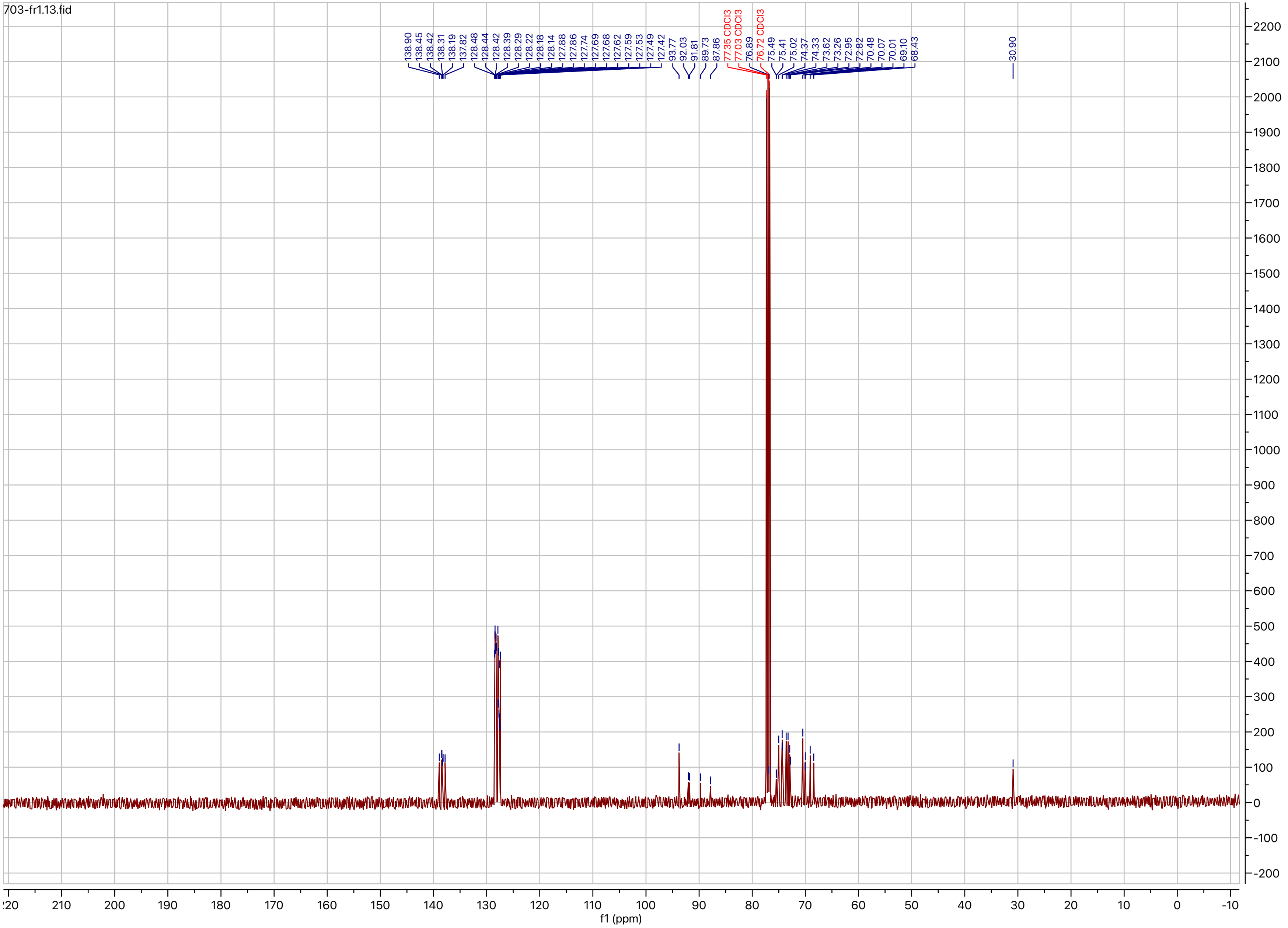

[19]F Spectrum of 15f (376 MHz, Chloroform-*d*)

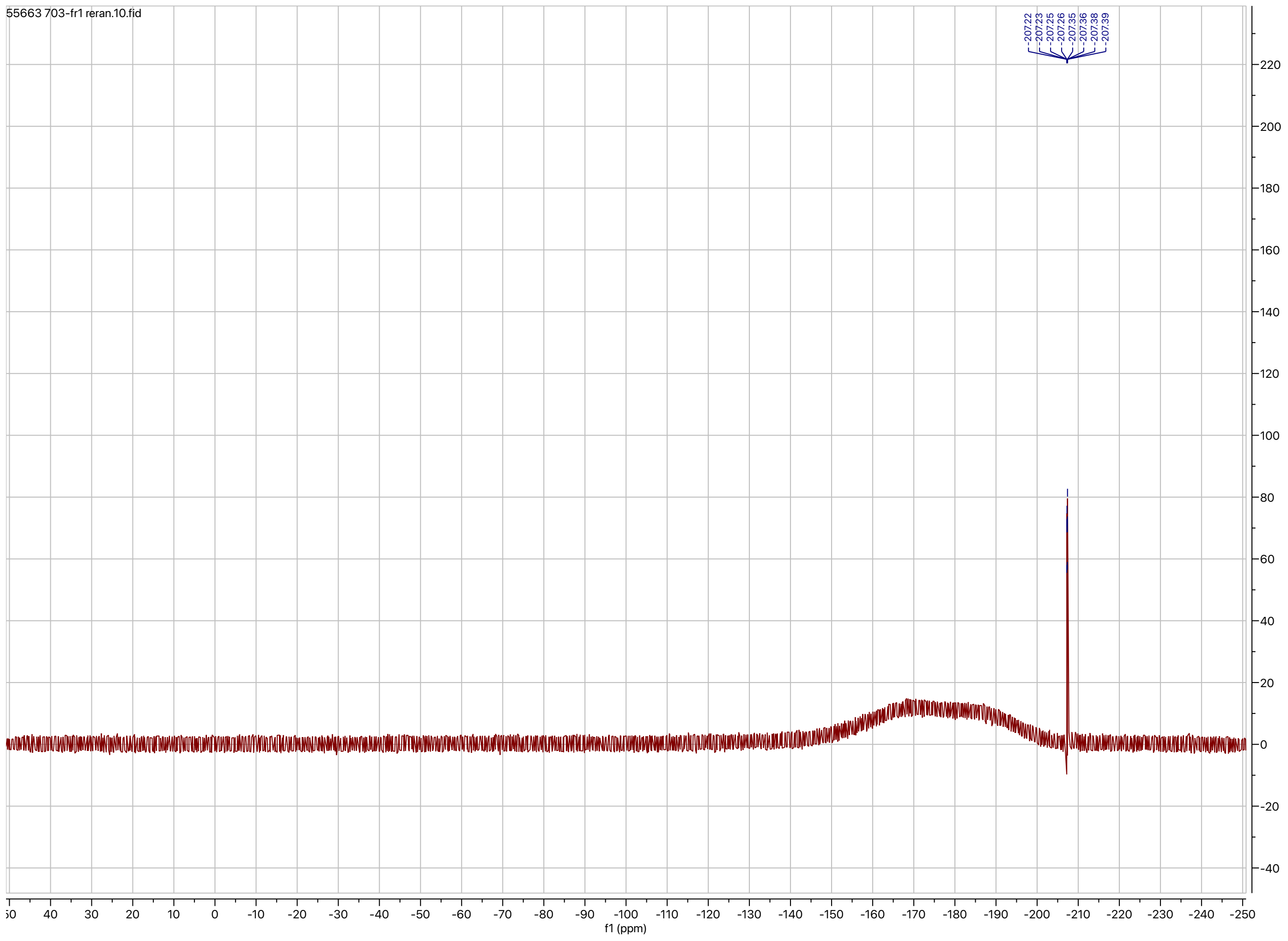

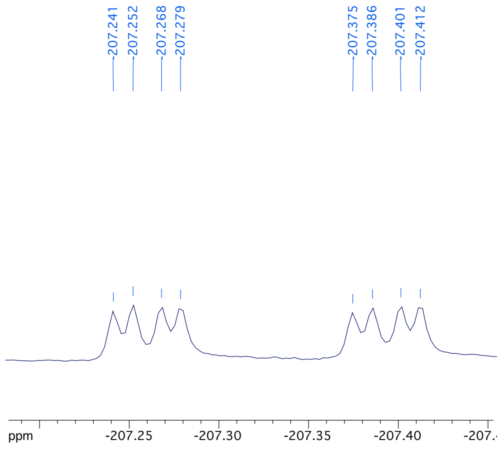

[1]H Spectrum of 16 (400 MHz, Chloroform-*d*)

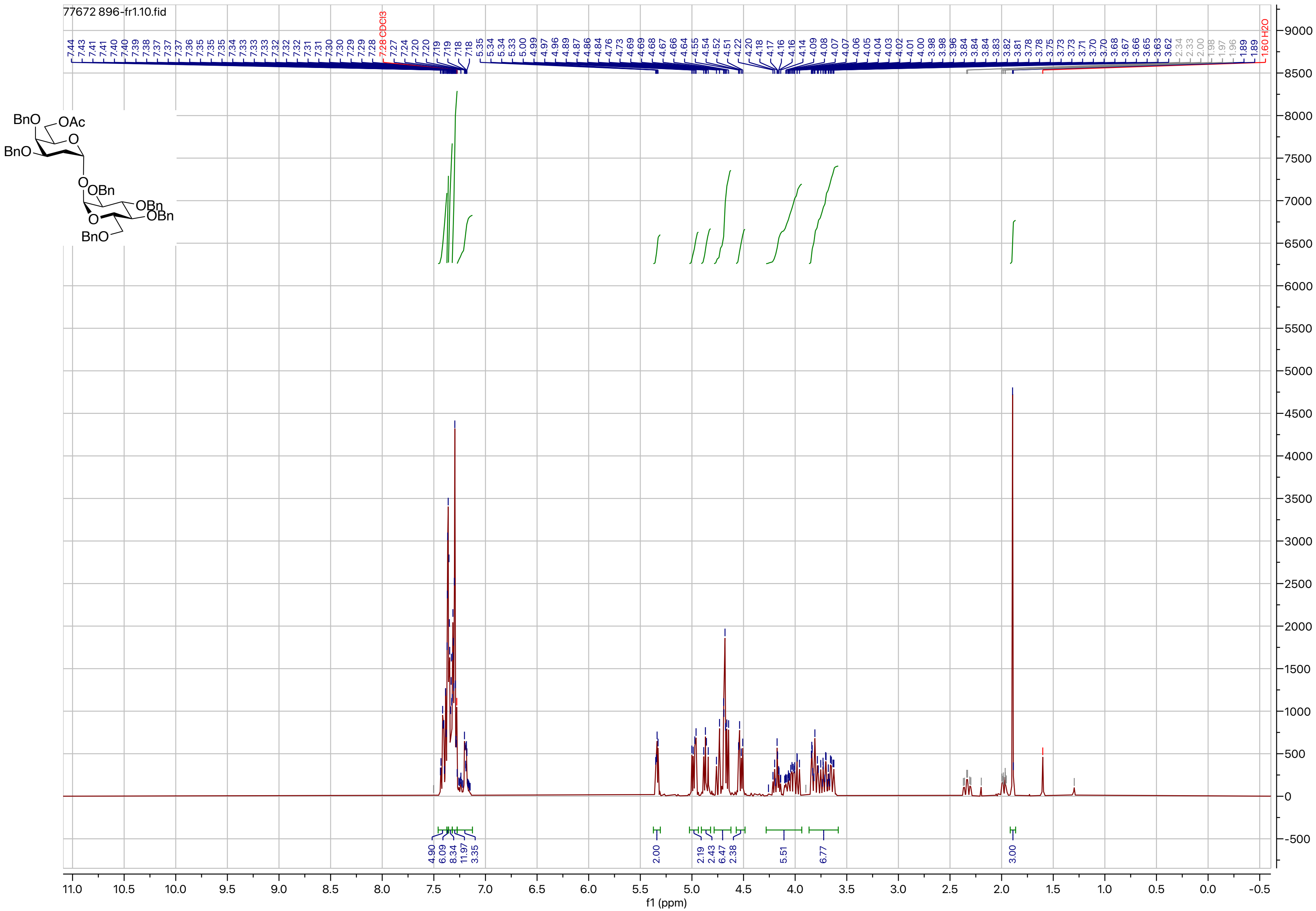

^{13}C Spectrum of 16 (101 MHz, Chloroform-*d*)

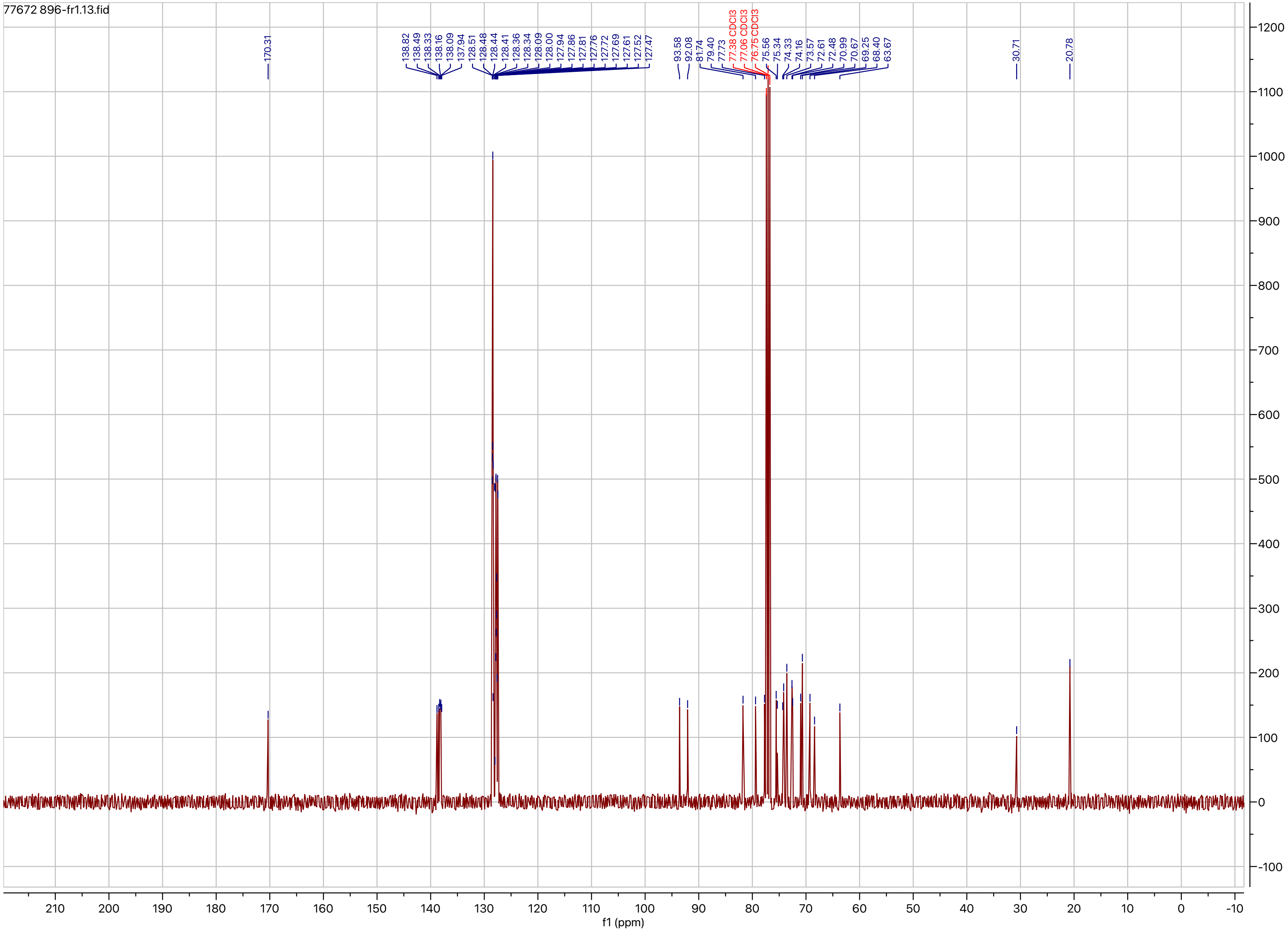

[1]H Spectrum of 17a (400 MHz, Chloroform-*d*)

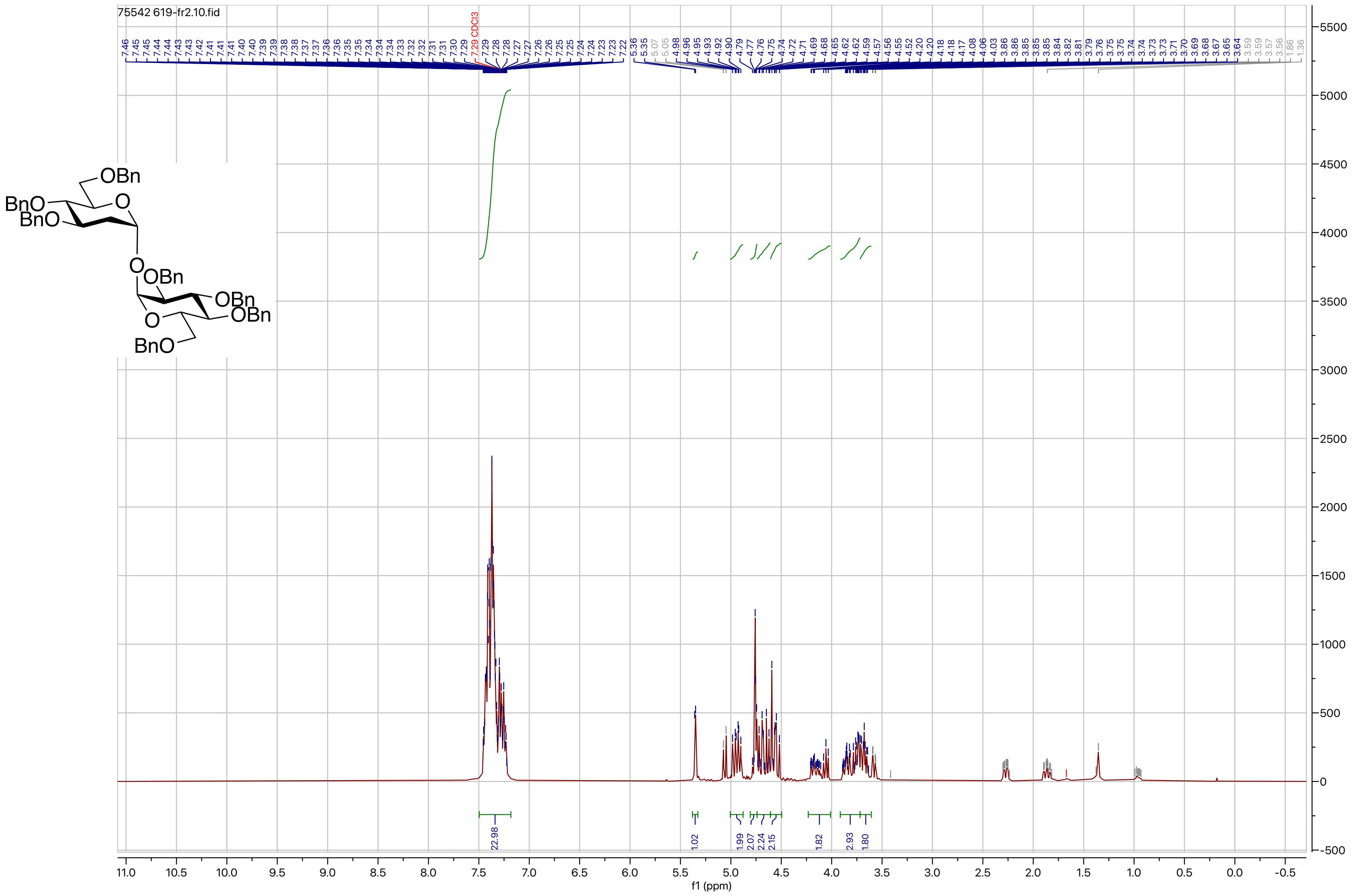

^{13}C Spectrum of 17a (101 MHz, Chloroform-*d*)

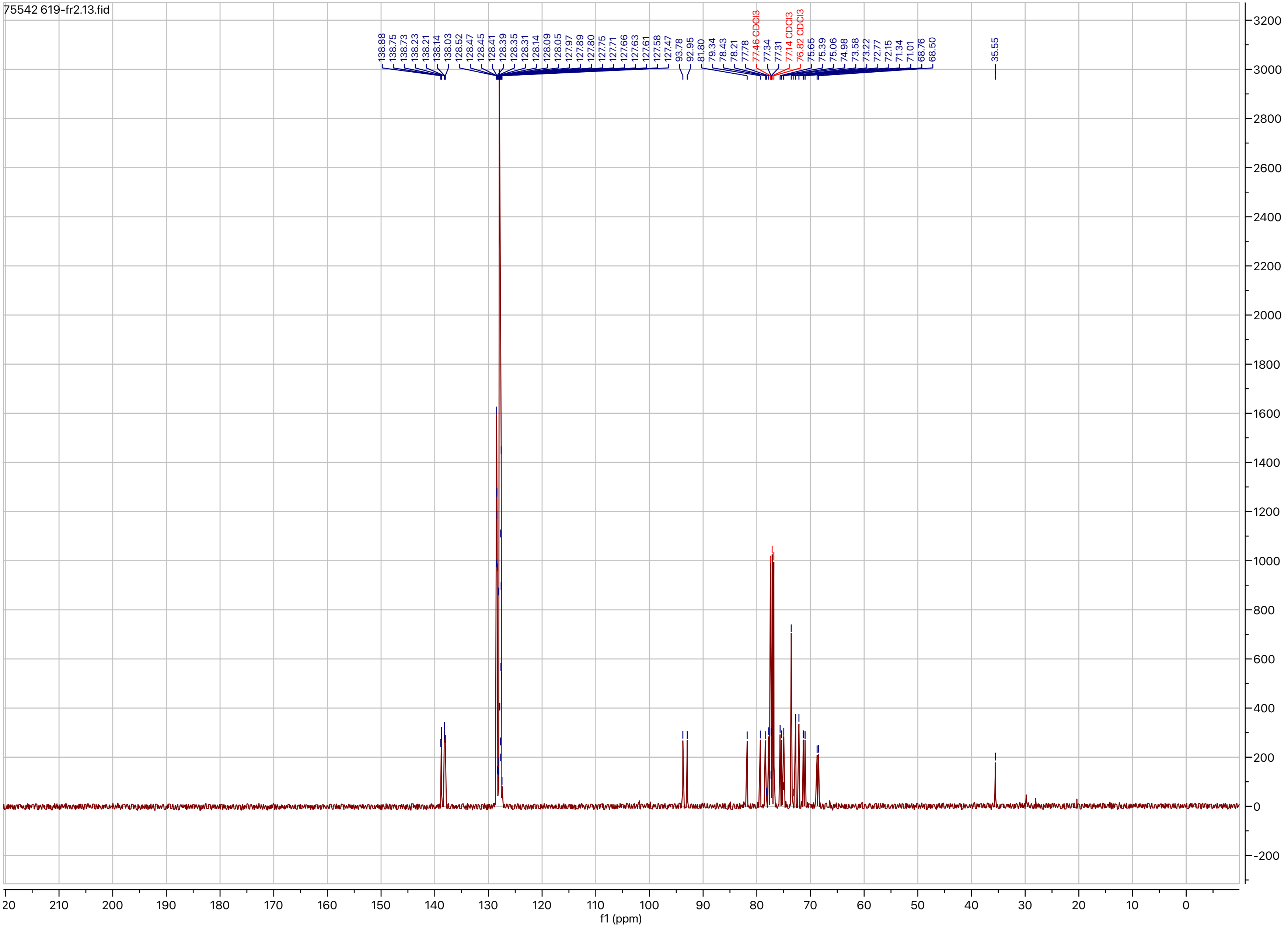

[1]H Spectrum of 17b (400 MHz, Chloroform-*d*)

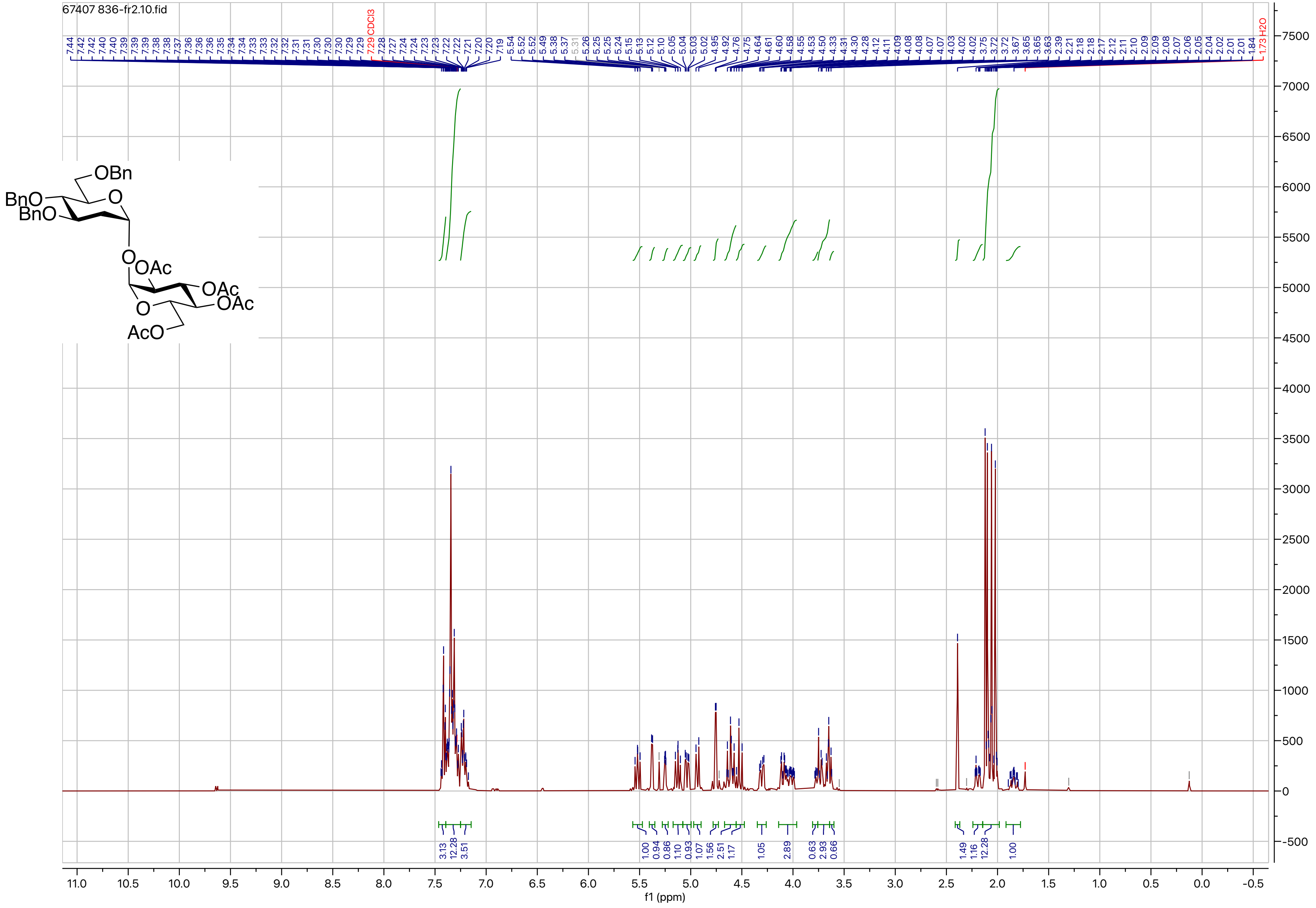

^{13}C Spectrum of 17b (101 MHz, Chloroform-*d*)

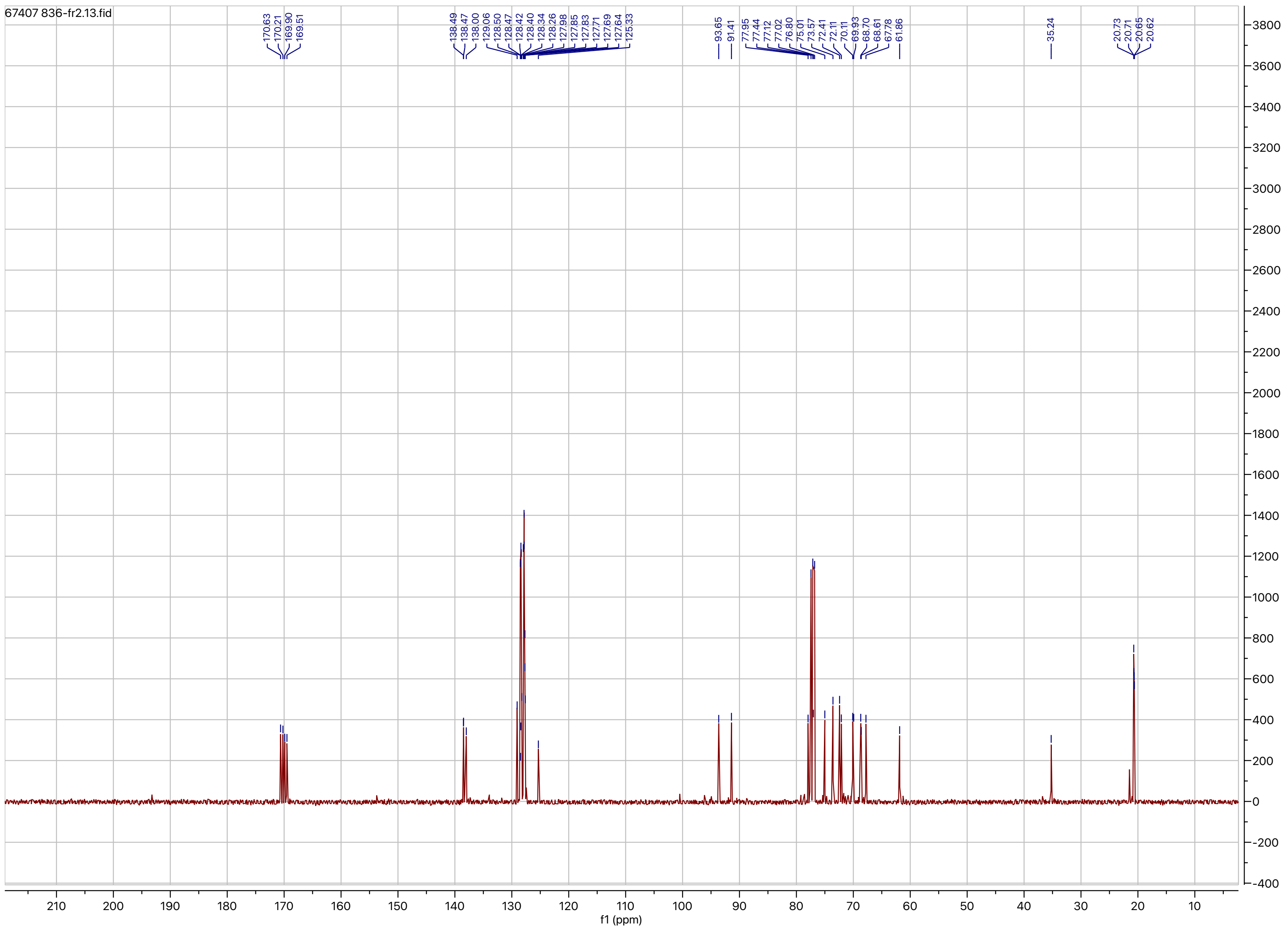

[1]H Spectrum of 17c (400 MHz, Chloroform-*d*)

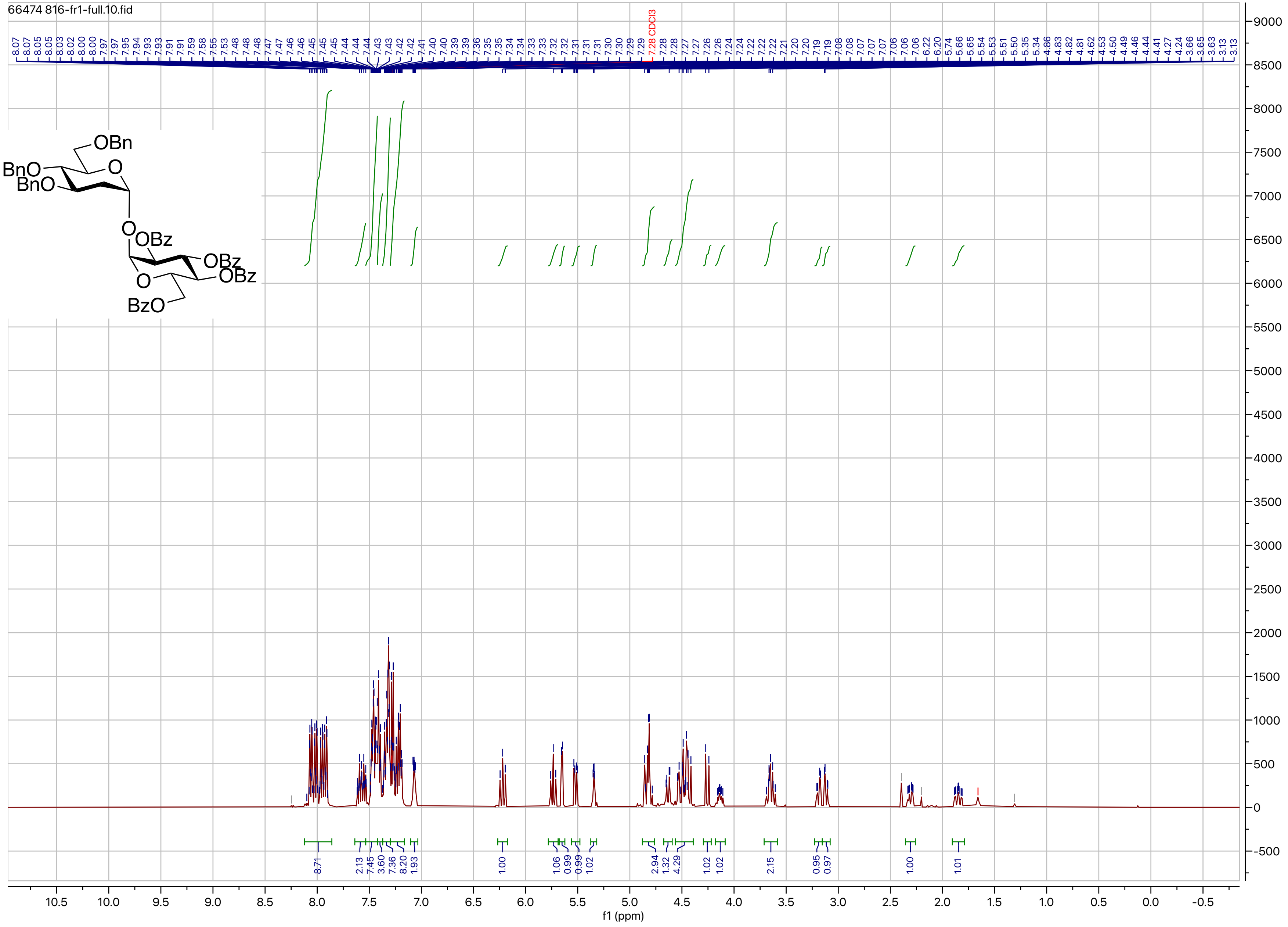

^{13}C Spectrum of 17c (101 MHz, Chloroform-*d*)

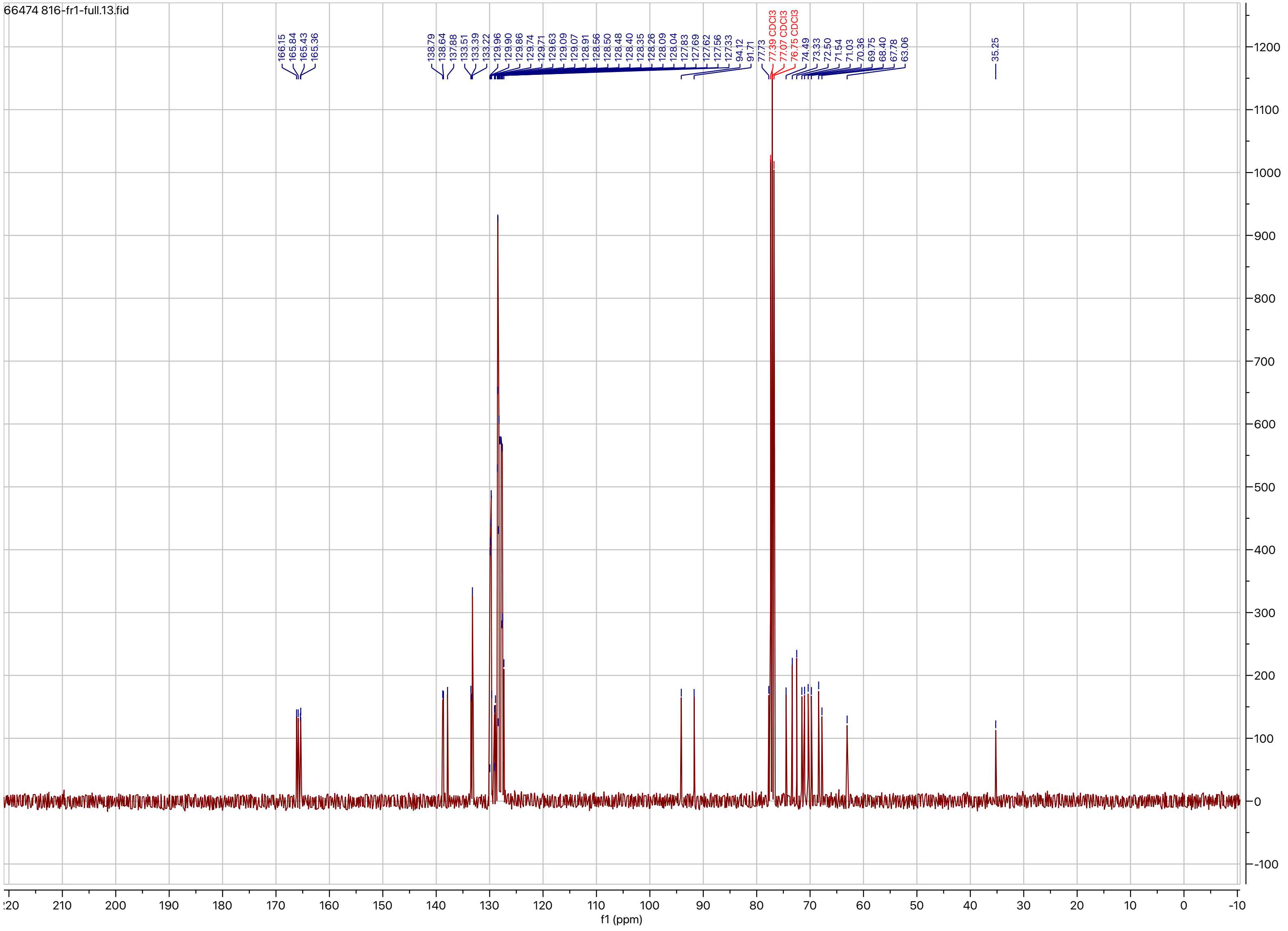

^{1}H Spectrum of 18 (400 MHz, Chloroform-*d*)

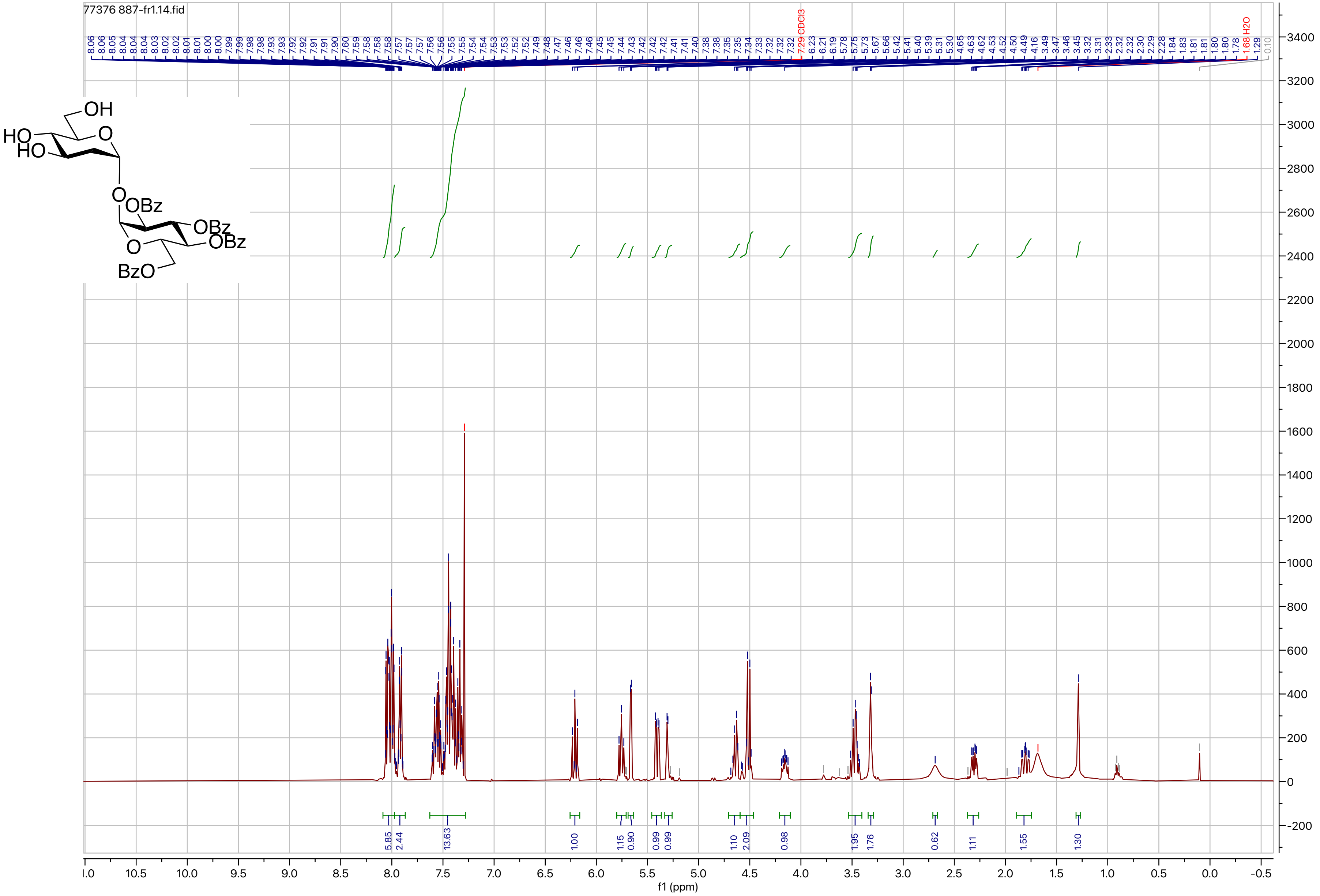

^{13}C Spectrum of 18 (101 MHz, Chloroform-*d*)

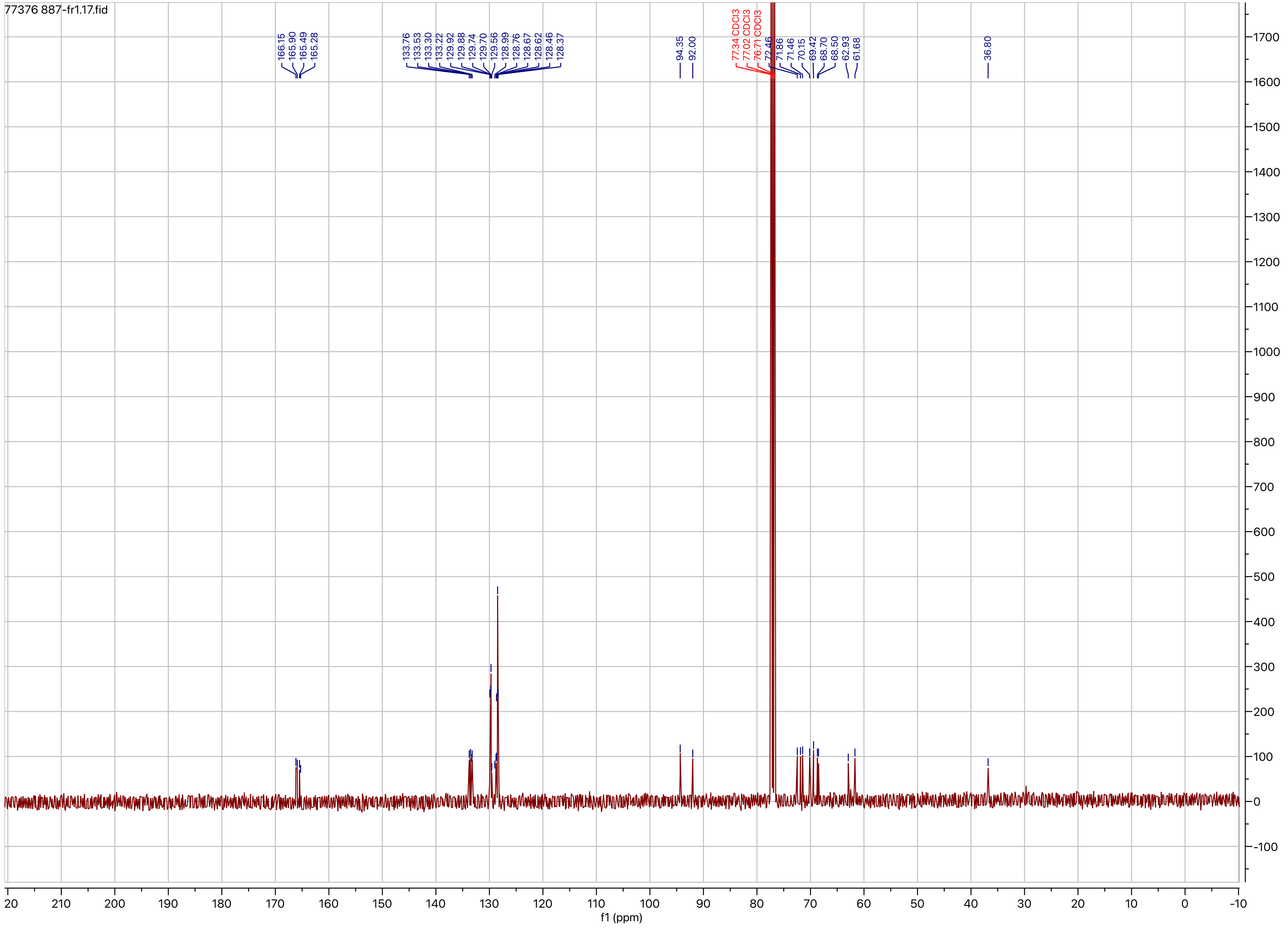

^{1}H Spectrum of 19 (400 MHz, Chloroform-*d*)

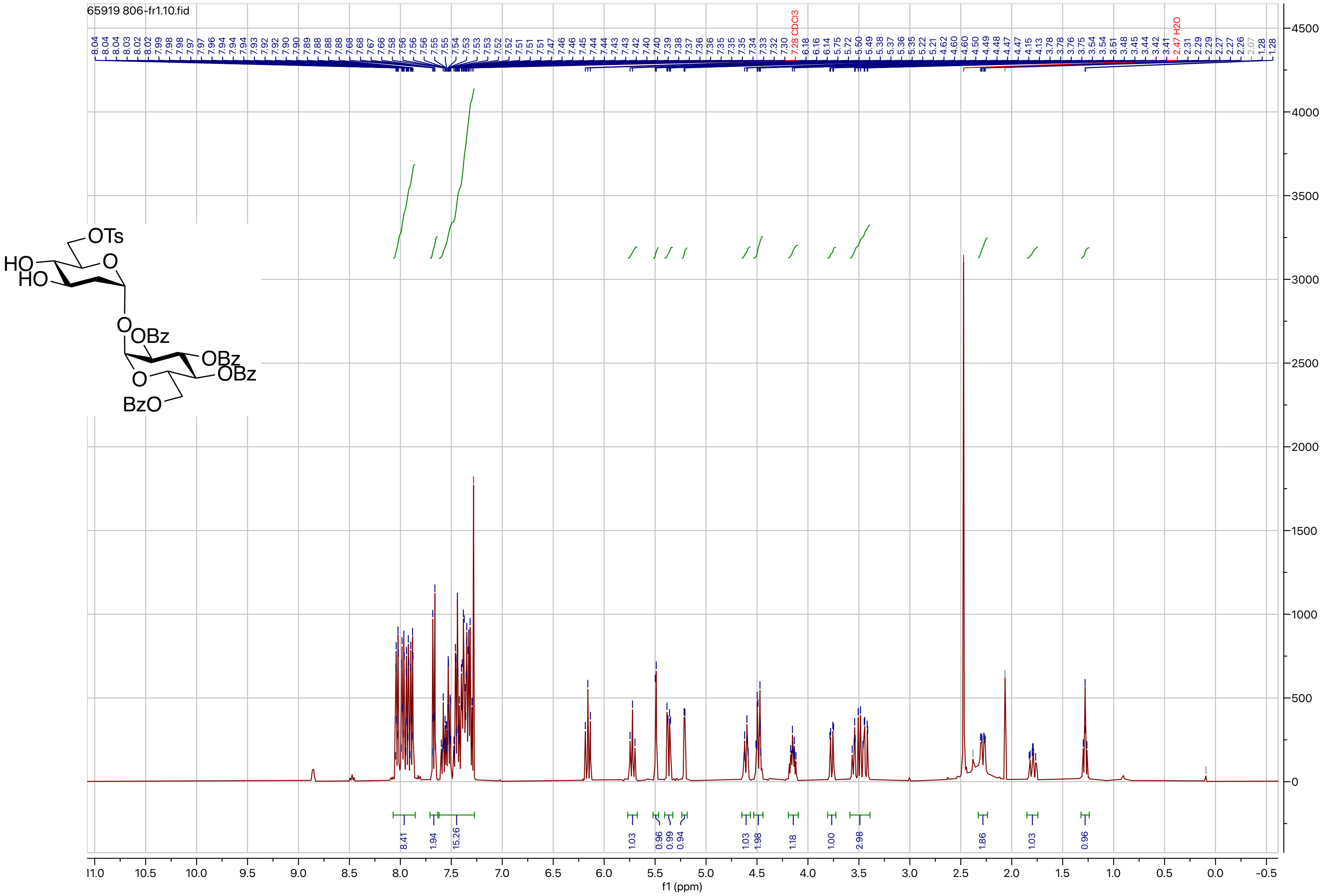

^{13}C Spectrum of 19 (101 MHz, Chloroform-*d*)

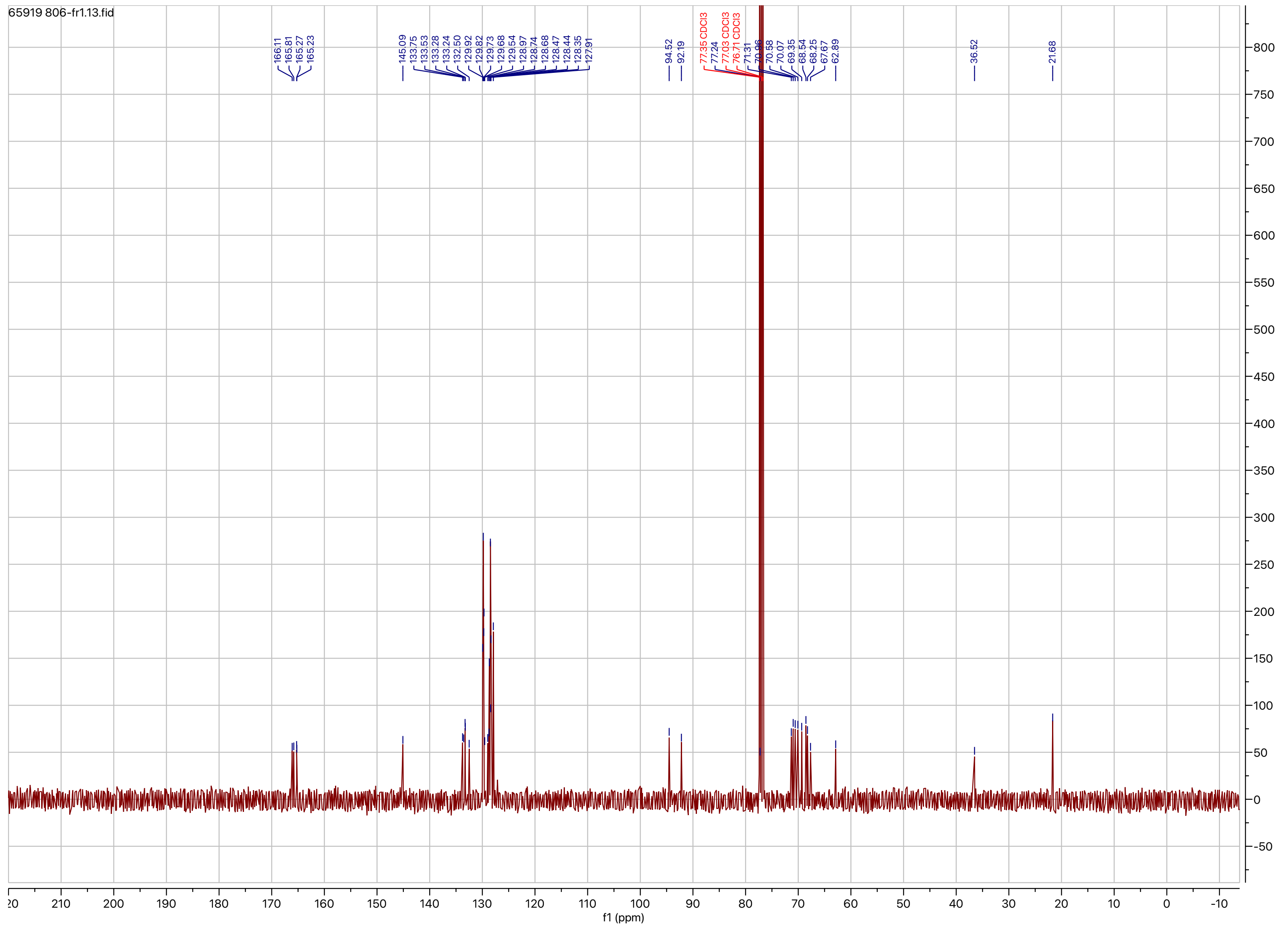

^{1}H Spectrum of 20 (400 MHz, Chloroform-*d*)

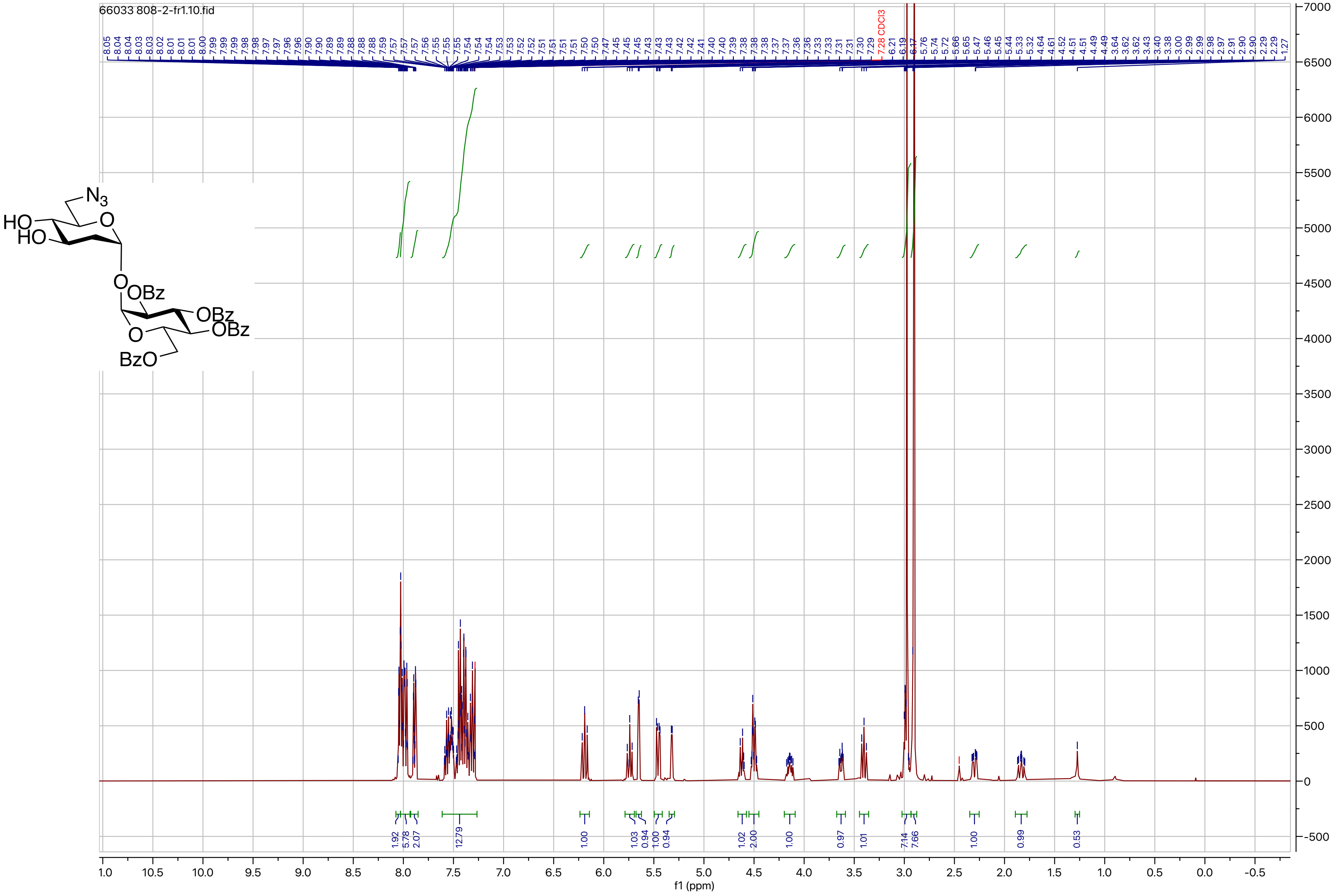

^{13}C Spectrum of 20 (101 MHz, Chloroform-*d*)

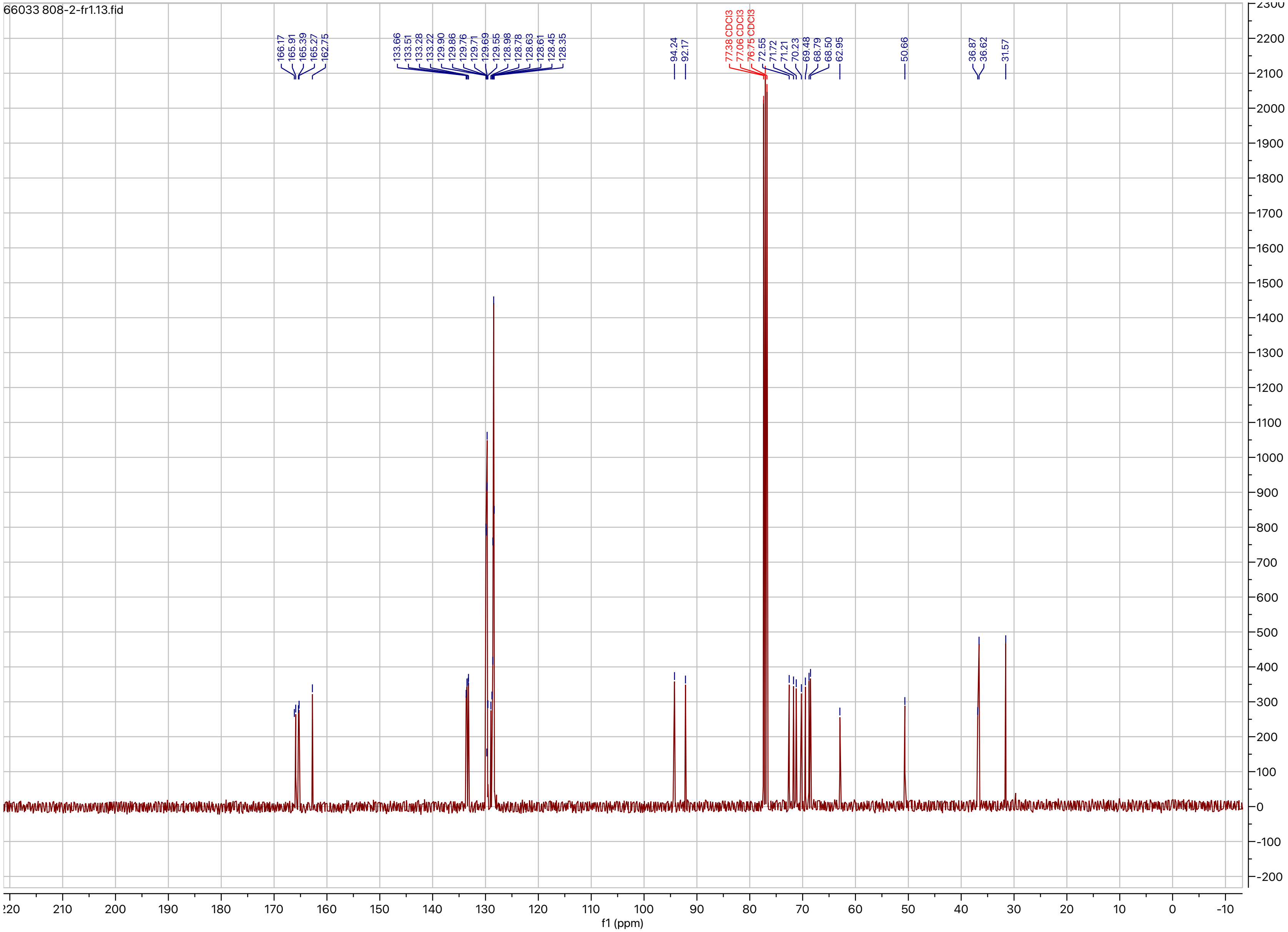

^{1}H Spectrum of 21 (400 MHz, MeOD-d_4)

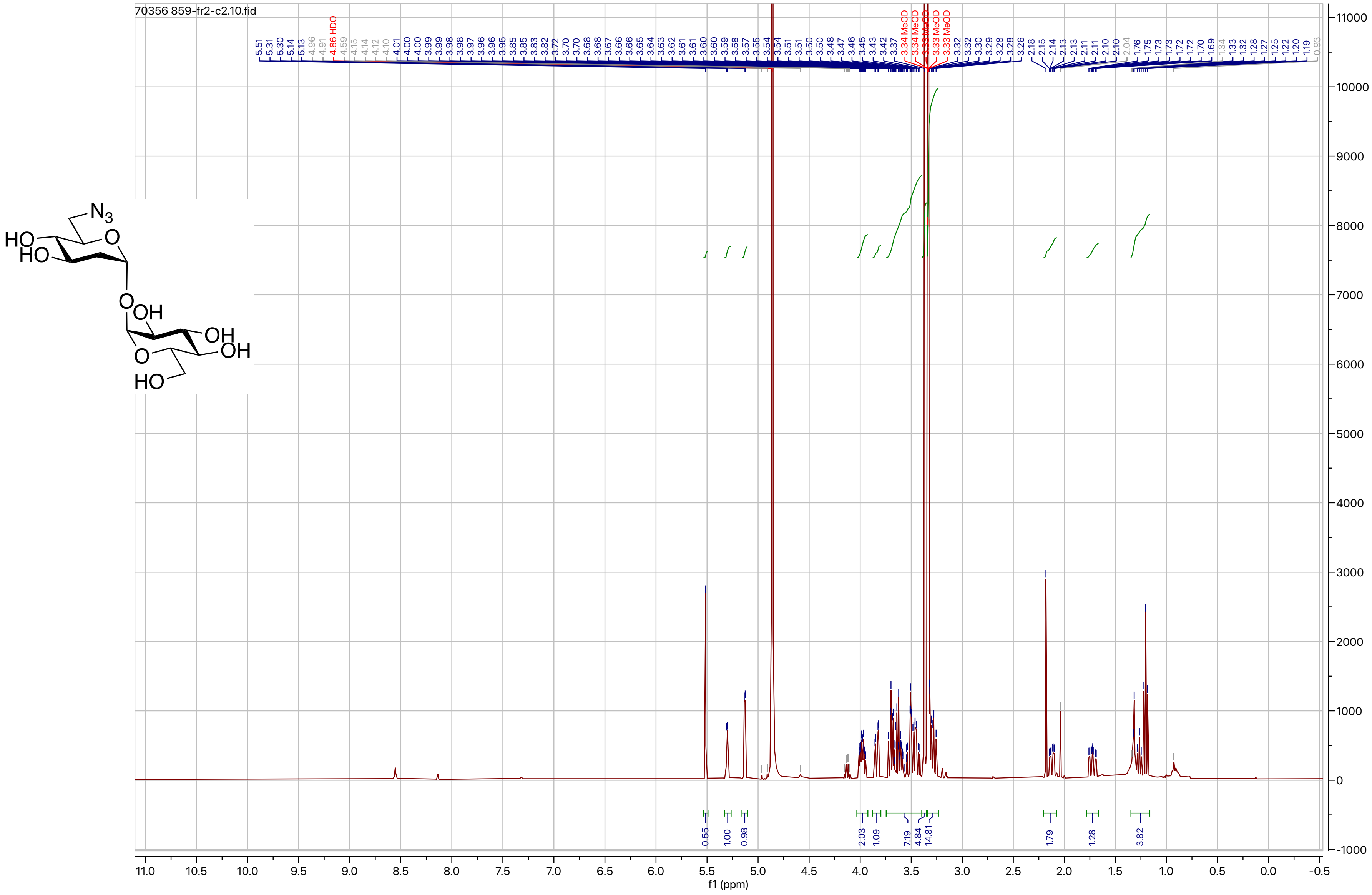

^{13}C Spectrum of 21 (101 MHz, MeOD-d_4)

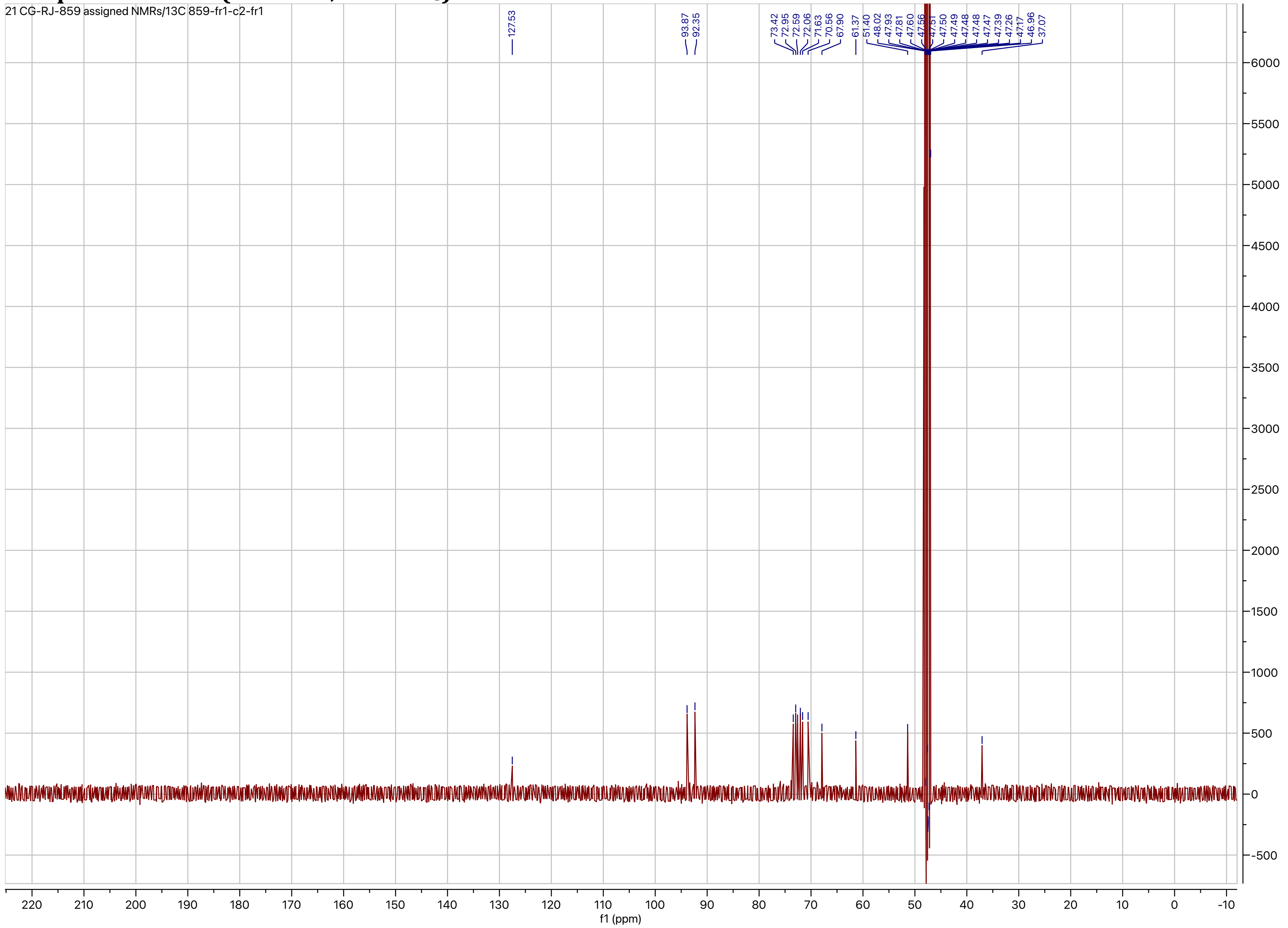

^{1}H Spectrum of 22 (400 MHz, Chloroform-*d*)

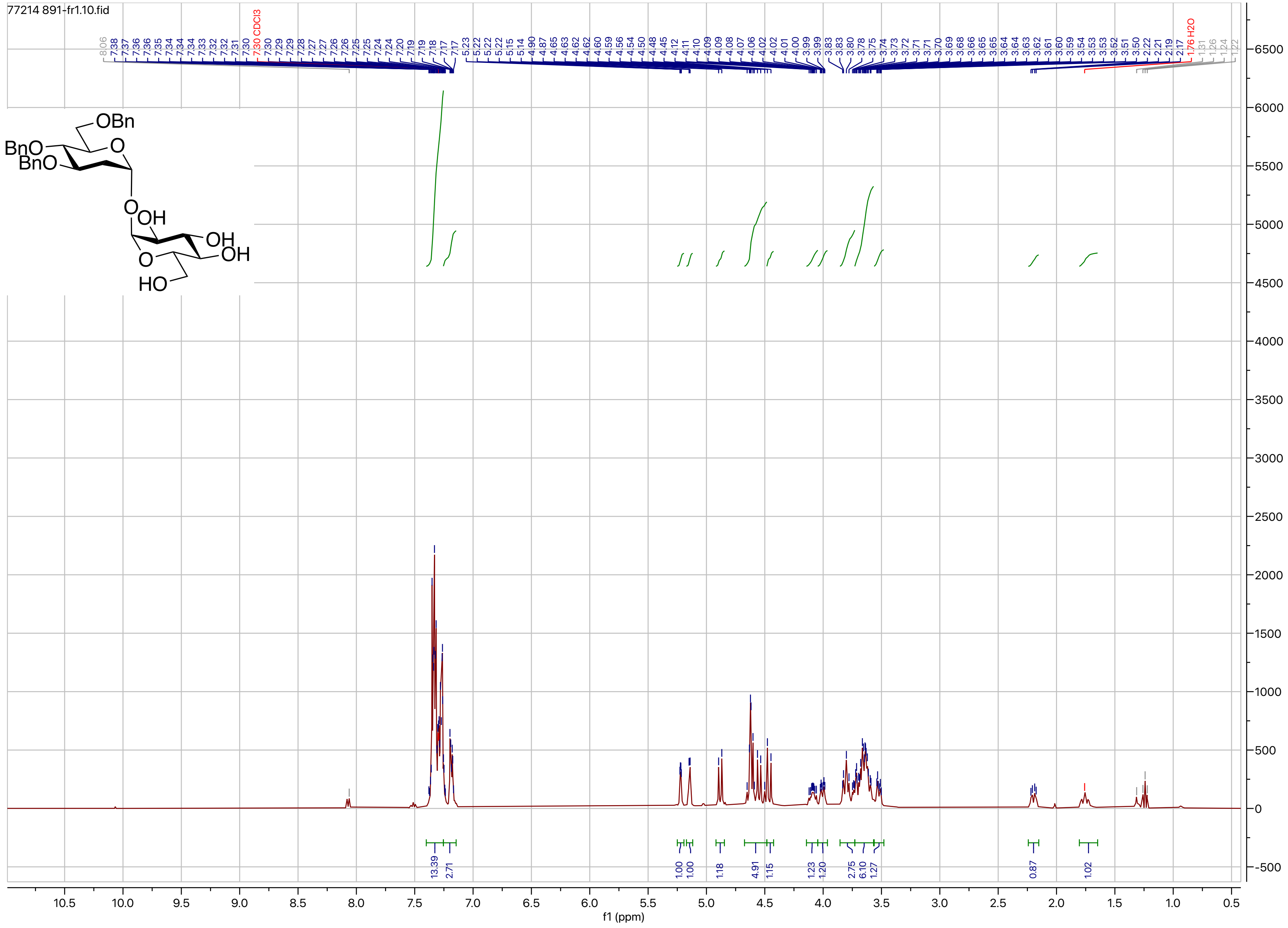

^{13}C Spectrum of 22 (101 MHz, Chloroform-*d*)

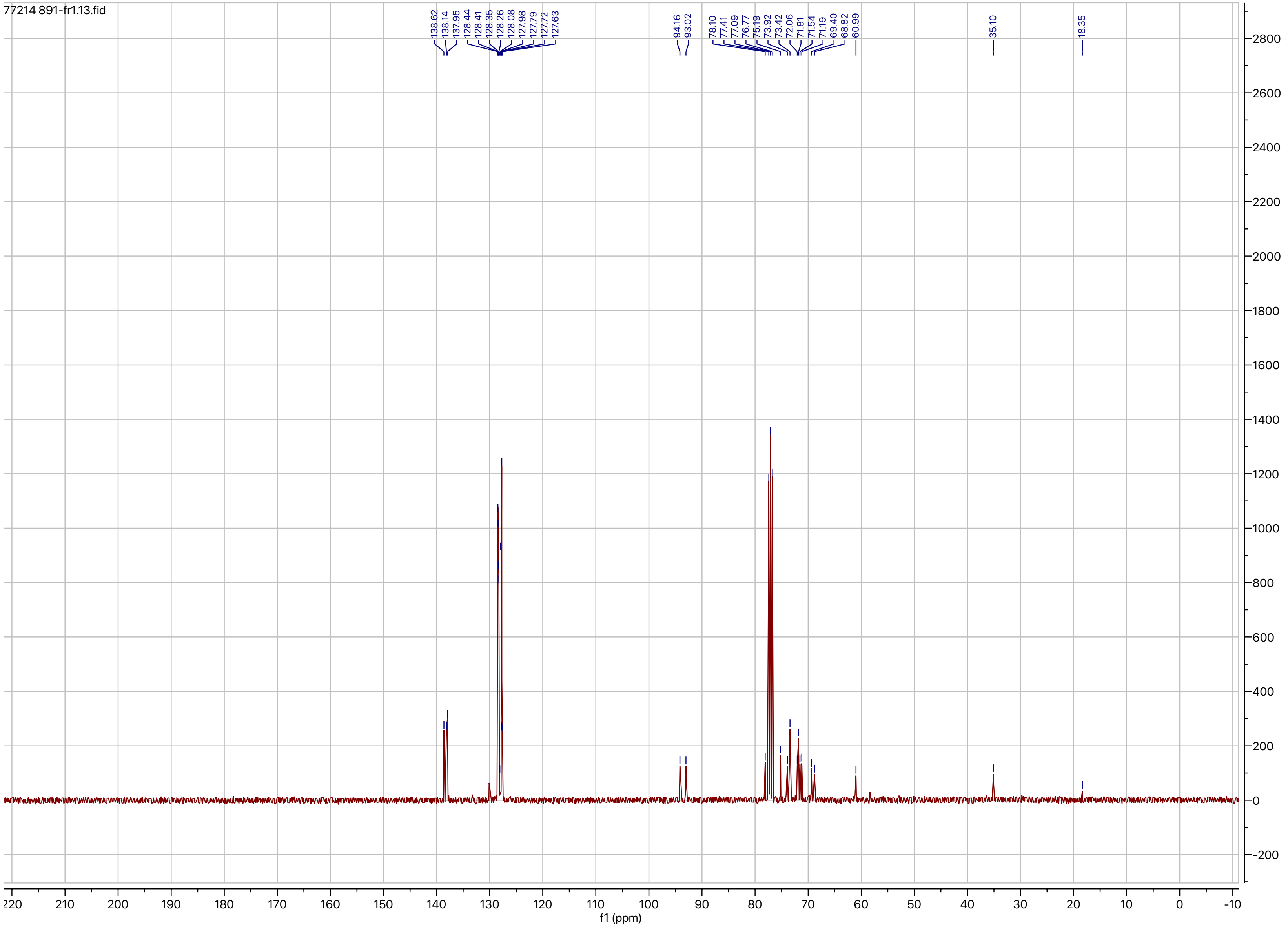

[1]H Spectrum of 23 (400 MHz, Chloroform-*d*)

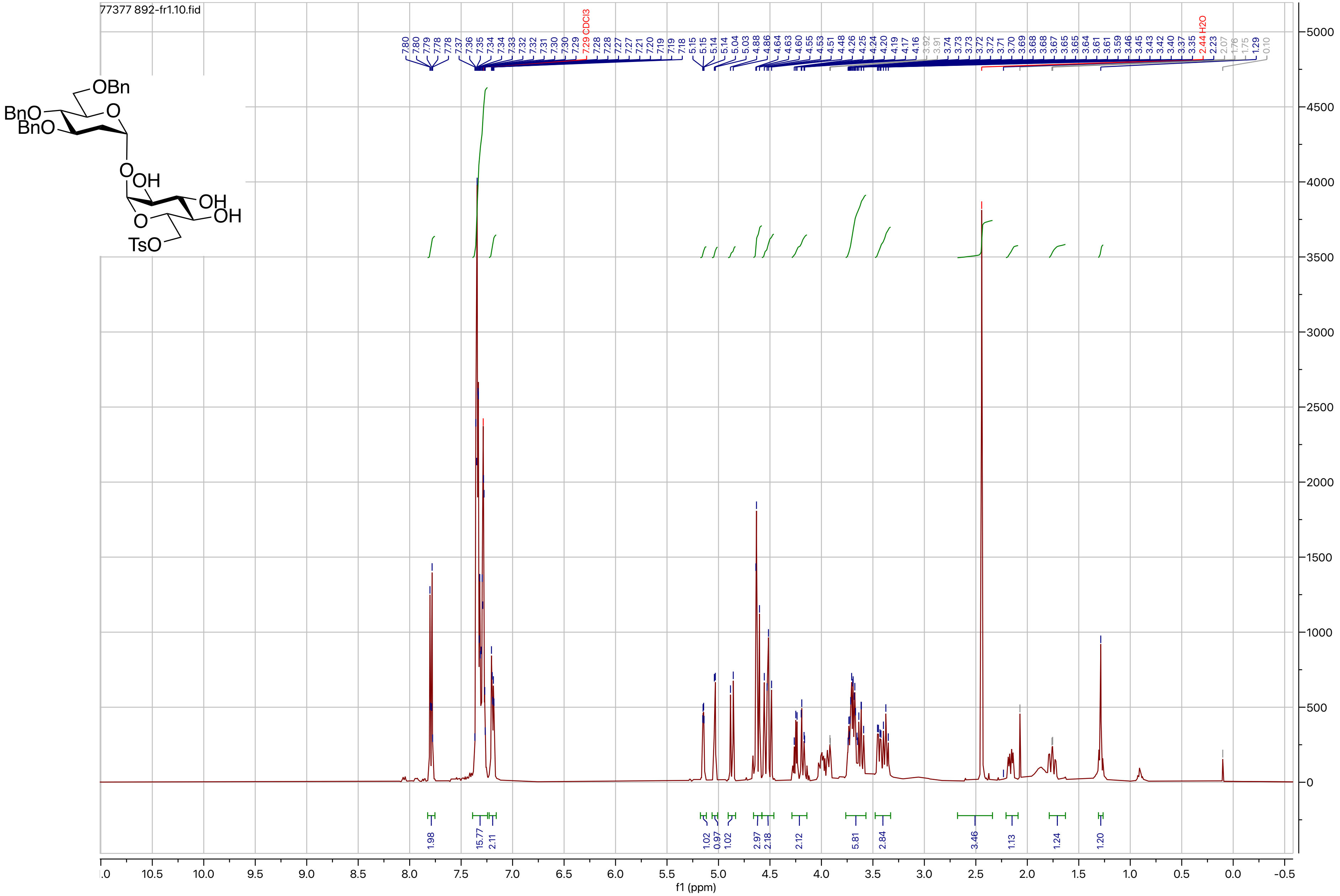

^{13}C Spectrum of 23 (101 MHz, Chloroform-*d*)

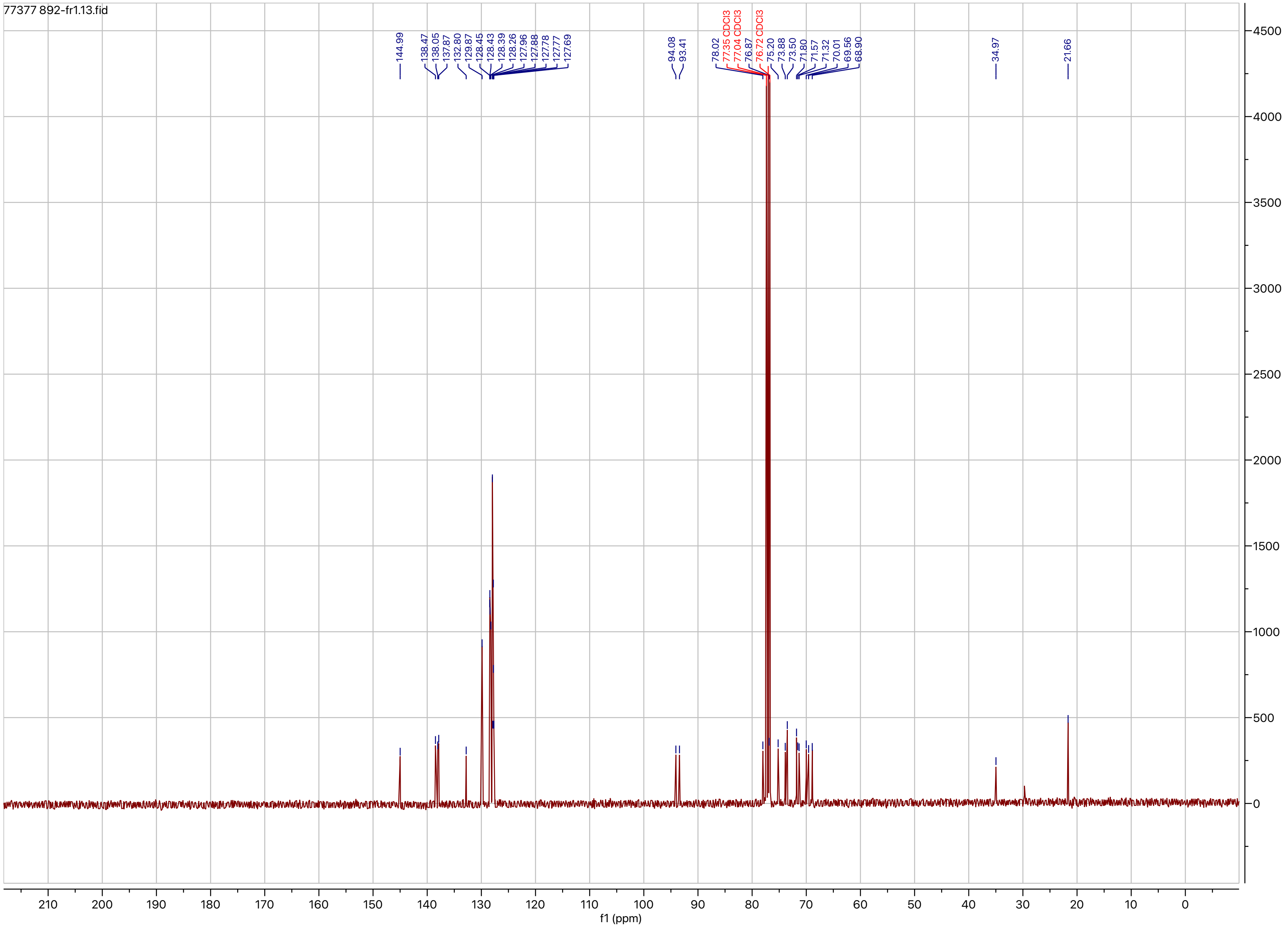

Crude ^{1}H Spectrum of 24 (400 MHz, D_2O)

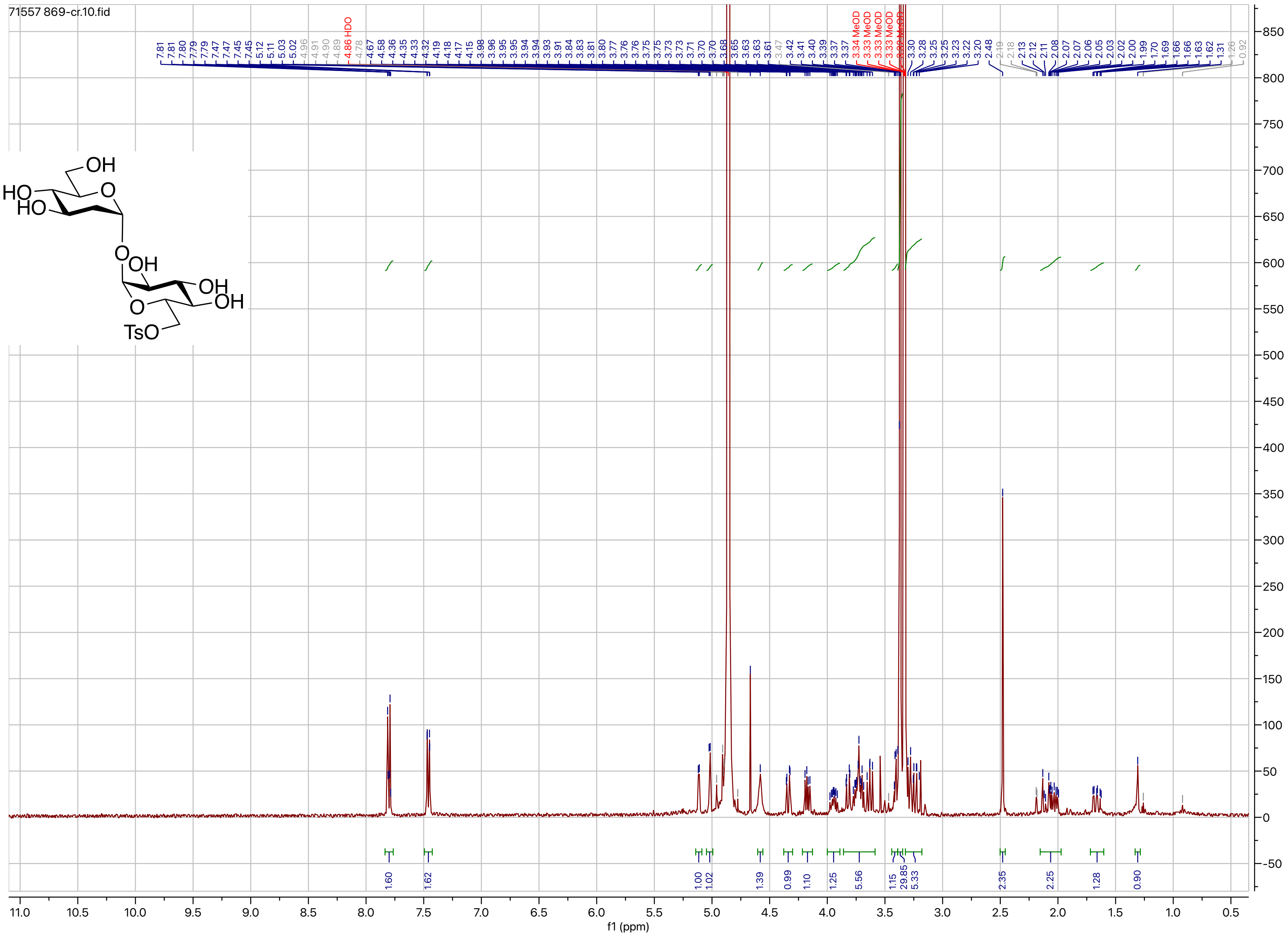

^{1}H Spectrum of 25 (400 MHz, D2O)

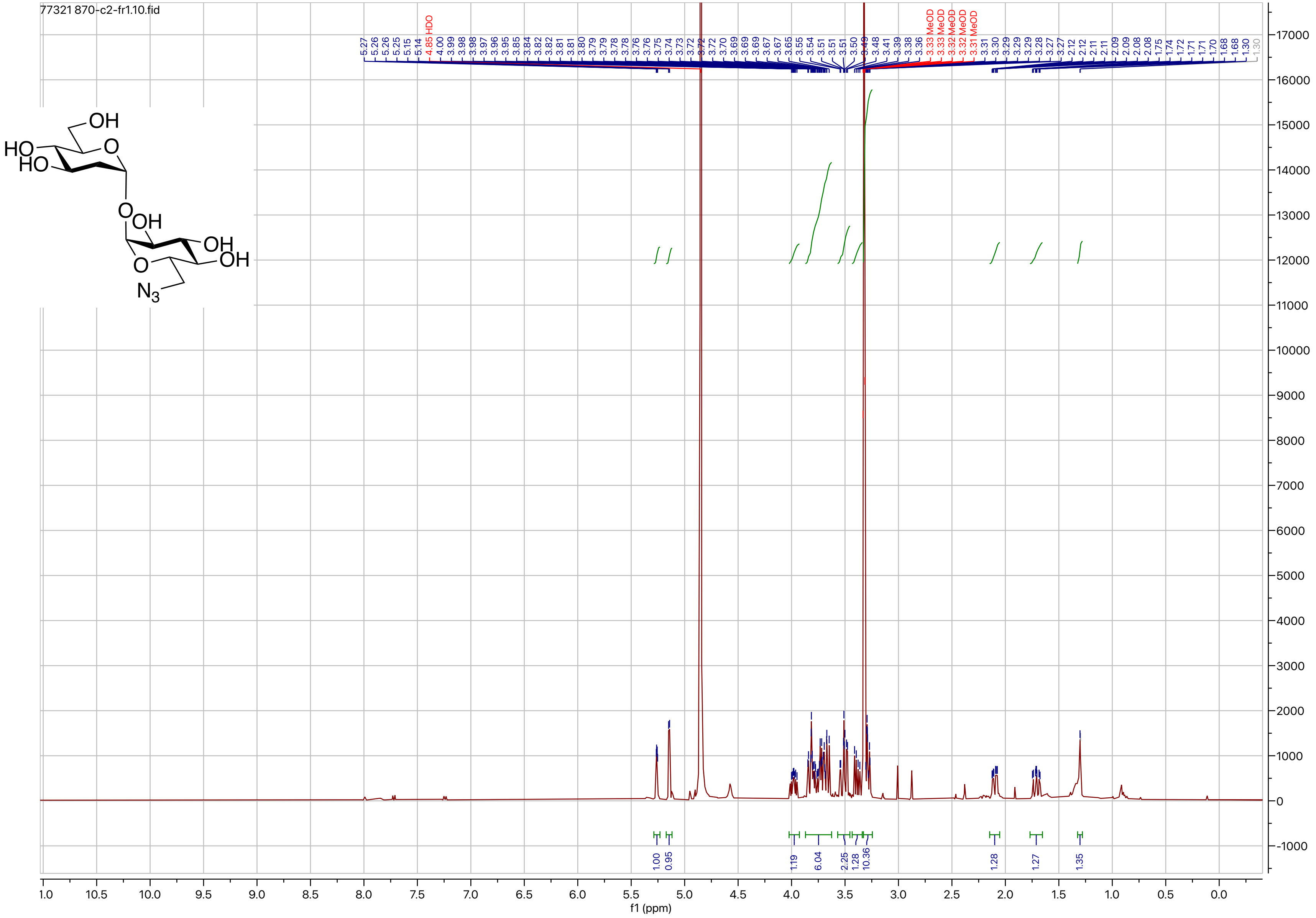

DEPT Spectrum of 25 (101 MHz, D2O-*d*)

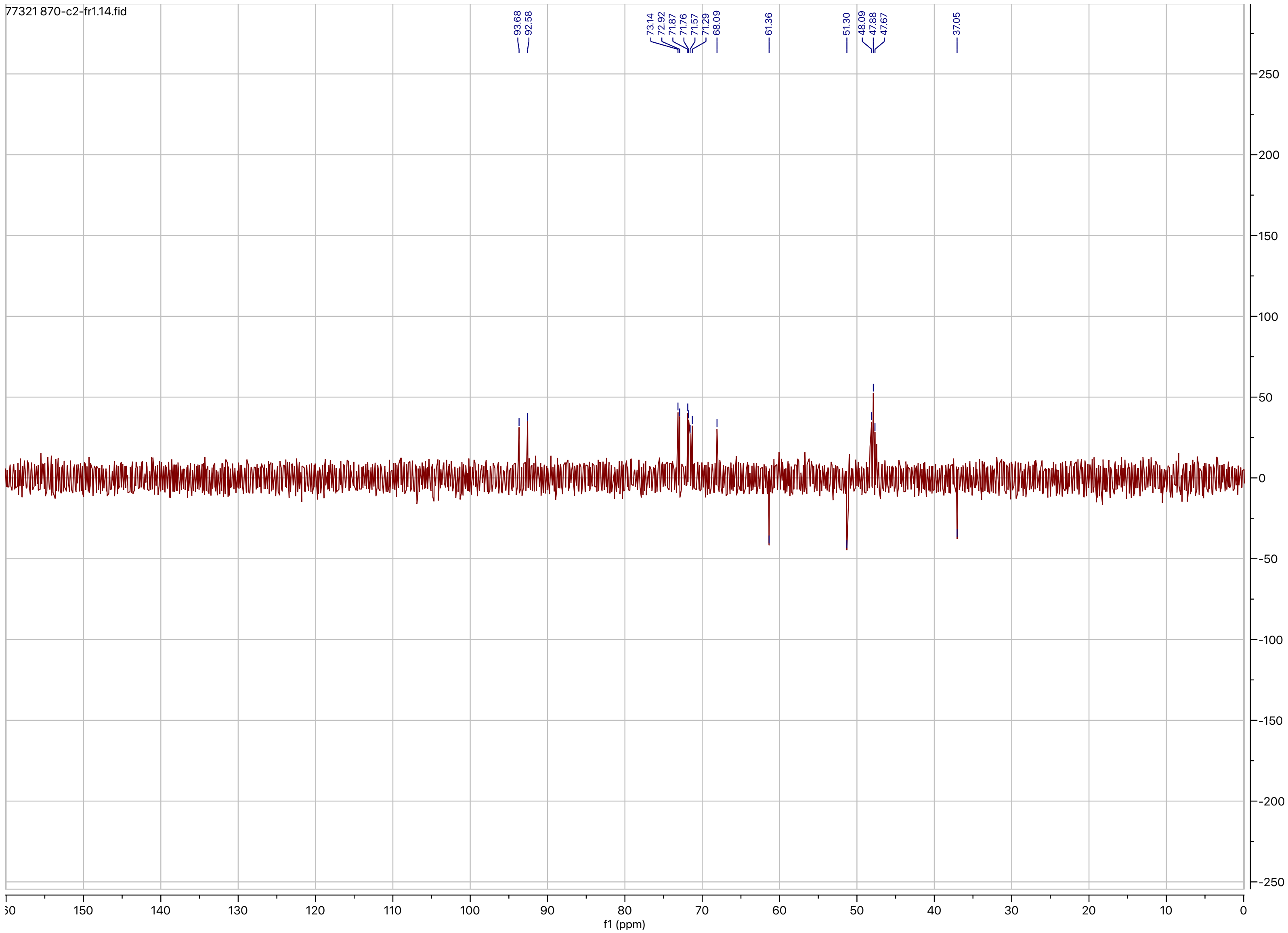

Supplement: Supplementary file 1 — ol2c02530_si_001.pdf [file ol2c02530_si_001.pdf]
